# Paeoniflorin alleviates CKD-associated constipation by modulating TPH1/AHR-related signaling and suppressing NLRP3/GSDMD-mediated pyroptosis

**DOI:** 10.3389/fphar.2026.1844043

**Published:** 2026-07-10

**Authors:** Jun Qiu, Zhimin Ao, Fenghua Yao, Jianing Zhang, Yong Wen, Yu Zhan

**Affiliations:** 1 Department of Nephrology, Fifth Medical Center of the Chinese PLA General Hospital, National Key Laboratory of Kidney Diseases, National Clinical Research Center for Kidney Diseases, Beijing, China; 2 Hospital of Chengdu University of TCM, Anorectal Department, Chengdu, Sichuan, China; 3 Department of Nephrology, First Medical Center of the Chinese PLA General Hospital, National Key Laboratory of Kidney Diseases, National Clinical Research Center for Kidney Diseases, Beijing, China; 4 Traditional Chinese Medicine Department, Fifth Medical Center of the Chinese PLA General Hospital, National Key Laboratory of Kidney Diseases, National Clinical Research Center for Kidney Diseases, Beijing, China; 5 Anorectal Department, Chengdu Integrated TCM & Western Medicine Hospital, Chengdu, China

**Keywords:** CKD-associated constipation, NLRP3/GSDMD pyroptosis, paeoniflorin, p-cresyl sulfate, TPH1/AHR-related signaling

## Abstract

**Introduction:**

Constipation is a common complication of chronic kidney disease (CKD) and contributes to a vicious cycle linking intestinal dysfunction and renal injury, yet effective targeted therapies remain limited. Paeoniflorin (PF), a major bioactive constituent of Paeonia lactiflora Pall., has reported renoprotective and gastrointestinal regulatory effects, but its role in CKD-associated constipation remains unclear.

**Methods:**

In this study, we investigated the therapeutic effects and underlying mechanisms of PF in an adenine-induced mouse model of CKD-associated constipation. Constipation-related phenotypes, renal function, renal and colonic histopathology, intestinal barrier markers, TPH1/AHR-related signaling, renal NLRP3/GSDMD-mediated pyroptosis, gut microbial composition, PCS-related metabolic alterations, and molecular docking with key enzymes involved in the tyrosine-p-cresol-PCS pathway were assessed.

**Results:**

PF dose-dependently improved constipation-related phenotypes and renal dysfunction, and the high-dose PF group was selected for subsequent mechanistic analyses. High-dose PF attenuated renal fibrosis, restored colonic mucosal architecture, and increased the expression of tight junction proteins. Mechanistically, PF upregulated TPH1 and AHR expression, suggesting modulation of TPH1/AHR-related signaling. In renal tissues, PF suppressed NLRP3/GSDMD-mediated pyroptosis and improved the expression of KCNK3 and OAT3. PF also selectively reshaped gut microbial composition and was associated with microbial functional changes related to amino acid metabolism and inflammatory signaling. In addition, PF was associated with PCS-related metabolic alterations and showed potential interactions with key enzymes involved in the tyrosine-p-cresol-PCS pathway.

**Discussion:**

These findings indicate that PF alleviated CKD-associated constipation and improved renal injury, possibly through integrated modulation of intestinal barrier integrity, gut microbial composition, TPH1/AHR-related signaling, and renal pyroptosis-related signaling. PF may therefore represent a potential pharmacological candidate for CKD-associated constipation.

## 1 Introduction

Chronic kidney disease (CKD) has evolved into a pressing global public health crisis, with its burden escalating dramatically over the past 3 decades. According to the 2023 Global Burden of Disease (GBD) Study, the global prevalence of CKD among adults aged 20 years and above has reached 788 million, representing a nearly twofold increase from 1990, and it currently ranks as the ninth leading cause of death worldwide (GBD 2023 Chronic Kidney Disease Collaborators, 2025). The World Health Organization (WHO) formally adopted a resolution on kidney health in 2025, urging member states to strengthen investments in CKD prevention and management, highlighting the urgent need for effective interventions (ERA’s ABCDE Framework for Kidney Disease Prevention, 2026). Beyond progressive renal dysfunction, CKD is frequently complicated by gastrointestinal disorders, among which constipation is a highly prevalent yet underaddressed comorbidity. Clinical data indicate that 30%–63% of CKD patients, particularly those receiving dialysis, suffer from constipation, which not only impairs quality of life but also forms a pathological vicious cycle with CKD (Lu et al., 2019). Constipation prolongs the intestinal transit of gut-derived uremic toxins, while impaired renal excretion exacerbates gut microbial dysbiosis and intestinal barrier dysfunction, ultimately accelerating renal fibrosis and cardiovascular complications (Wan et al., 2023).

The gut-kidney axis has emerged as a core regulatory network linking intestinal homeostasis to renal function, integrating insights from nephrology, microbiology, gastroenterology, and metabolomics. In CKD, the imbalance between saccharolytic and proteolytic gut bacteria leads to reduced production of short-chain fatty acids (SCFAs) and excessive accumulation of uremic toxins, including indoxyl sulfate (IS) and p-cresol sulfate (PCS) (Tsuji et al., 2024). Indoxyl sulfate, a key metabolite of tryptophan catabolism by intestinal flora, accumulates in CKD due to impaired renal clearance and directly induces renal tubular cell apoptosis and endothelial dysfunction (Yamamoto and Fukagawa, 2017). Concurrently, tryptophan metabolism plays a pivotal role in regulating intestinal motility: dietary tryptophan is metabolized to serotonin (5-HT), a critical neurotransmitter for gut peristalsis, via the rate-limiting enzyme tryptophan hydroxylase 1 (TPH1), with downstream signaling mediated by the aryl hydrocarbon receptor (AHR) (Wen et al., 2022). Dysregulation of the TPH1/AHR axis in CKD has been associated with reduced 5-HT synthesis and impaired gut motility.

Parallel to metabolic dysregulation, the NLRP3/GSDMD pyroptosis pathway contributes significantly to the pathological crosstalk between gut and kidney in CKD. Aberrant activation of this pathway induces intestinal epithelial cell death, disrupts barrier integrity, and promotes the release of proinflammatory cytokines such as IL-1β and IL-6, which further suppress colonic smooth muscle contractility and aggravate renal interstitial fibrosis. Despite growing evidence linking tryptophan metabolism and pyroptosis to CKD and constipation individually, their synergistic regulation in the context of CKD-related constipation remains incompletely understood, creating a critical gap in the development of targeted therapies.

Paeoniflorin (PF), a principal bioactive component isolated from Paeonia lactiflora Pall., has garnered attention for its multifaceted pharmacological properties, including renoprotective, anti-inflammatory, and gut microbiota-modulating effects. Previous studies demonstrated that PF alleviates renal fibrosis by inhibiting uremic toxin accumulation and improves intestinal barrier integrity through regulating TLR4/NF-κB signaling pathway (Jan et al., 2024). However, whether PF alleviates CKD-related constipation through concurrent regulation of tryptophan metabolism (TPH1/AHR axis) and the NLRP3/GSDMD pyroptosis pathway has not been systematically investigated.

In the current study, we first evaluated the therapeutic efficacy of PF on constipation symptoms and renal function in adenine-induced CKD mice. Subsequently, we investigated its regulatory effects on TPH1/AHR-mediated tryptophan metabolism and the activation of the NLRP3/GSDMD pathway in intestinal and renal tissues. Ultimately, we delineated the synergistic mechanisms underlying these interconnected signaling cascades, with a focus on their crosstalk in mediating gut-kidney axis homeostasis.

## 2 Materials and methods

### 2.1 Materials

**FIGURE 1:**
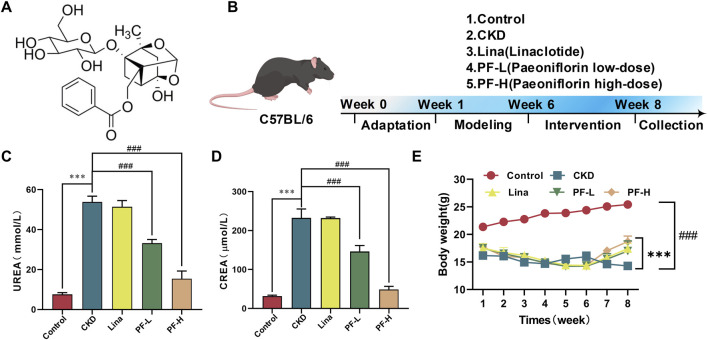
PF improved renal function in adenine-induced CKD mice. **(A)** Chemical structure of paeoniflorin. **(B)** Schematic diagram of the animal experimental design. **(C)** Serum blood urea nitrogen (BUN) levels. **(D)** Serum creatinine (Scr) levels. **(E)** Body weight changes during the experimental period; no significant intergroup difference was observed at baseline before intervention. Data are presented as mean ± SD (n = 6 per group). Statistical analysis was performed using one-way ANOVA followed by Bonferroni’s *post hoc* test for **(C)** and **(D)**. For **(E)**, statistical comparisons among groups were performed using one-way ANOVA followed by Bonferroni’s *post hoc* test at the indicated time point. Compared with the control group, ##P < 0.01 and ###P < 0.001; compared with the CKD group, *P < 0.05, **P < 0.01, and ***P < 0.001; &P < 0.05 and &&P < 0.01 indicate statistical significance between the connected groups.

Paeoniflorin (purity ≥98%) was purchased from Shanghai Macklin Biochemical Co., Ltd. Figure 1 displays the chemical structure of the PF. Linaclotide was obtained from AstraZeneca Pharmaceuticals Co., Ltd. The 0.2% adenine-containing diet was purchased from Jiangsu Xiehong Pharmaceutical Biotechnology Co., Ltd. Mouse Serum Urea Nitrogen (BUN) and Serum Creatinine (CREA) Detection Kits were acquired from Nanjing Jiancheng Bioengineering Institute, while the Mouse PCS ELISA Kit was obtained from Hefei Bomei Biotechnology Co., Ltd. Primary antibodies against AhR, NLRP3, GSDMD, Pro-Caspase-1, IL-1β, OAT3, KCNK3, claudin, occludin and ZO-1 were obtained from ABclonal Biotechnology Co., Ltd (Wuhan, China). The second antibody was purchased from Wuhan Sevier Biotechnology Co., Ltd (Wuhan, China).

### 2.2 Animal experiment

Six–seven-week-old specific pathogen-free (SPF) male C57BL/6 mice, weighing 20–22 g, were purchased from Sichuan Weitong Lihua Experimental Animal Technology Co., Ltd (Certificate of Quality for Experimental Animal Production: SCXK (Chuan) 2023–0,263). All animal housing conditions and experimental procedures strictly complied with the regulations and guidelines of the Animal Ethics Committee of Sichuan Provincial Animal Association, with the Ethics Approval Number: 202104A005. After 7 days of acclimatization, the mice were subjected to adenine-induced chronic kidney disease (CKD) model establishment. They had free access to food and water under a regular light/dark cycle, and the animal facility was maintained at a temperature of (22 ± 2) °C and humidity of 55% ± 5% (Certificate of Permission for Experimental Animal Use: SYXK (Chuan) 2021–246).

### 2.3 CKD induction and PF treatment

After 7 days of acclimatization, a total of 30 mice were randomly assigned to five groups (n = 6 per group) using a random number table, and each group received the corresponding intervention as follows: (1) Control group (Con), fed a basal diet and gavaged with distilled water for 14 consecutive days; (2) CKD model group (CKD), fed a 0.2% adenine-containing diet and gavaged with distilled water for 14 consecutive days; (3) Linaclotide treatment group (Lina), fed a 0.2% adenine-containing diet and gavaged with linaclotide suspension at 100 μg/(kgd) for 14 consecutive days; (4) Paeoniflorin low-dose group (PF-L), fed a 0.2% adenine-containing diet and gavaged with paeoniflorin at 100 mg/(kgd) for 14 consecutive days; and (5) Paeoniflorin high-dose group (PF-H), fed a 0.2% adenine-containing diet and gavaged with paeoniflorin at 200 mg/(kgd) for 14 consecutive days. The PF doses (100 and 200 mg/kg/day) were pre-specified based on our previous study. An initial dose-screening analysis was then conducted using constipation-related phenotypes and renal functional parameters. Based on the overall efficacy profile, the higher dose, which showed the most consistent improvement in constipation-related and renal functional indices, was selected as the representative effective dose for subsequent exploratory mechanistic analyses, including pathology, intestinal barrier integrity, tryptophan metabolism-related signaling, pyroptosis-related signaling, and gut microbiota assessment.

Successful establishment of the CKD-associated constipation model was confirmed by elevated serum blood urea nitrogen (BUN) and creatinine (CREA) levels, together with reduced fecal water content and intestinal transit rate. The overall experimental design is shown in Figure 1B.

### 2.4 Determination of constipation-related indicators

Fecal morphology of mice was observed, and fecal water content was measured. Mice in each group were individually housed in metabolic cages, and feces excreted within 24 h were collected and counted for each group. The wet weight (W1) of the collected feces was weighed using an electronic balance; subsequently, the feces were dried in a 60 °C incubator for 12 h and reweighed to obtain the dry weight (W2). The fecal water content was calculated using the following formula: Fecal water content (%) = [(Wet weight of feces − Dry weight of feces)/Wet weight of feces] × 100%.After the above measurements, intestinal transit time was determined in six mice per group. Mice were administered a charcoal powder solution via oral gavage, and the time from gavage to the excretion of the first black fecal pellet was recorded as the intestinal transit time. Prior to sacrifice, mice in all groups were fasted with free access to water withdrawn for 24 h, followed by oral gavage of charcoal powder solution. Thirty minutes post-gavage, mice were euthanized and mesenteries were dissected. For length measurement, the mesentery was gently stretched into a straight line, allowed to settle for 30 s, and measured under a tension-free condition. The total length of the small intestine was defined as L1, and the distance traveled by the charcoal powder was recorded as L2. The gastrointestinal transit ratio was calculated using the formula below: Gastrointestinal transit ratio (%) = Distance of charcoal powder migration (cm)/Total length of small intestine (cm)) × 100%.

### 2.5 Detection of plasma creatinine and blood urea nitrogen

Serum samples were collected from mice in each group following a 12-h fasting period. Blood was harvested via abdominal aorta puncture, and the upper serum fraction was separated by centrifugation. The concentrations of creatinine and blood urea nitrogen (BUN) were determined strictly in accordance with the manufacturer’s protocols of the corresponding assay kits.

### 2.6 ELISA analysis of serum

Serum samples were collected from mice and analyzed using an enzyme-linked immunosorbent assay (ELISA) kit. The concentration of PCS was determined with a commercial ELISA kit (Mouse PCS ELISA KIT, Cat. No. ELS-3558–1, 48 Test, Bomei Biotechnology Co., Ltd., Hefei, China) in accordance with the manufacturer’s protocol.

### 2.7 Immunofluorescence staining

To detect the expression of GSDMD and NLRP3 in mouse renal tissues, mouse kidney sections were fixed with 4% paraformaldehyde (PFA). Subsequently, the sections were subjected to permeabilization and blocking with phosphate-buffered saline (PBS) containing 0.3% Triton X-100% and 3% bovine serum albumin (BSA) for 1 h. The mouse kidney sections were then incubated overnight at 4 °C with primary antibodies against NLRP3 and GSDMD (1:100 dilution) in blocking buffer to avoid nonspecific binding. After washing with PBS, the sections were divided into two groups and incubated with CY3-conjugated secondary antibody and horseradish peroxidase (HRP)-conjugated secondary antibody, respectively. Additionally, staining for GSDMD and NLRP3 was performed with FITC-Tyramide (Servicebio, Cat. No. G1222) in accordance with the manufacturer’s instructions. Fluorescent images were captured using an inverted fluorescence microscope. Statistical analysis. Semi-quantitative analysis of immunofluorescence was performed using ImageJ software. For each group, representative fields were analyzed under identical acquisition settings, and the mean fluorescence intensity of GSDMD and NLRP3 was calculated.

### 2.8 Hematoxylin-eosin (H&E) and Masson’s trichrome staining

Mouse kidney tissues and distal colon tissue samples were fixed in 4% paraformaldehyde (PFA) for 24 h, followed by standardized dehydration. The processed tissues were then cut into 4-μm-thick sections. The sections were stained with hematoxylin and eosin (HE) for microscopic examination.

### 2.9 Western blotting analysis

Total proteins were extracted from distal colon and kidney tissues using RIPA lysis buffer supplemented with 1% protease inhibitor, respectively. The mixtures were centrifuged at 12,000 rpm for 15 min at 4 °C, and the supernatants were collected for subsequent analyses. The prepared protein samples were separated by Sodium Dodecyl Sulfate-Polyacrylamide Gel Electrophoresis (SDS-PAGE) and then transferred onto polyvinylidene fluoride (PVDF) membranes. After blocking with 5% bovine serum albumin (BSA) at room temperature for 2 h, the membranes were incubated overnight at 4 °C with specific primary antibodies as follows: anti-AhR (1:1000, ABclonal), anti-Occludin (1:1000, ABclonal), anti-TPH1 (1:6000, ABclonal), anti-Caspase-1 (1:1000, ABclonal), anti-IDO1 (1:1000, ABclonal), anti-KCNK3 (1:1000, ABclonal), anti-NLRP3 (1:1000, Bosterbio), anti-OAT3 (1:6000, ABclonal), anti-GSDMD (1:1000, Proteintech), anti-IL-1β (1:6000, ABclonal), and anti-β-actin (1:5000, ABclonal). Following three washes with TBST (10 min each), the membranes were incubated with horseradish peroxidase (HRP)-conjugated secondary antibody (1:5000, ABclonal) at room temperature for 2 h. Protein bands were detected using an enhanced chemiluminescence (ECL) substrate (Oriscience, China), and densitometric analysis was performed using ImageJ software for quantification.

### 2.10 16s rRNA sequencing of intestinal microbiota

For gut microbiota analysis, fecal samples were collected at the time of sacrifice after the 14-day treatment period and immediately stored at −80 °C until DNA extraction. Genomic DNA of intestinal flora was extracted using E. Z.N.A.® Soil DNA Kit (Omega Bio-Tek, Norcross, GA, United States of America), and then diluted to 1 ng/μL using sterile water. The targeted sequencing regions were amplified using specific primers. The PCR products, extracted from a 2% agarose gel, were detected and quantified using the QuantiFluor™-ST blue fluorescence quantification system (Promega Corporation, United States of America), and then mixed in corresponding proportions according to the sequencing requirements. The construction of libraries was executed using the TruSeq DNA Sample Prep Kit (Illumina, San Diego, CA, United States of America). Finally, purified amplicons were paired-end sequenced on an Illumina MiSeq platform. The paired-end reads obtained from Miseq sequencing were initially concatenated based on their overlap relationships. After sequence quality control and filtering, operational taxonomic unit clustering analysis and species classification analysis were performed to identify changes in gut bacteria between different populations.

### 2.11 PF molecular virtual docking analysis

Molecular virtual docking simulations of PF with target enzymes were conducted using Discovery Studio Client software (version 16.1.0.15350). The crystal structures of the target enzymes, namely, TyrB, PAH, TPL, HPDB, SULT1B1, and UGT2B28, were obtained from the Protein Data Bank (PDB). Docking tests involving PF and these enzymes were conducted using the CDOCKER algorithm as the primary docking approach. During the simulation procedure, the target proteins were kept in a fixed conformation, while the ligand molecules were allowed complete flexibility. A concluding energy minimization phase was executed to refine the docked conformations, with all other parameters set to their default configurations.

### 2.12 Quantification and statistical analysis

Statistical analysis was done by GraphPad Prism10.6. Unless otherwise indicated, data are represented as mean ± standard deviation (SD). Differences in the quantitative data were analyzed using 2-tailed unpaired t-test between two groups, and one-way analysis of variance (ANOVA) followed by Bonferroni *post hoc* test for more than two groups. N.S. represents no significance (ns), and ∗P < 0.05, ∗∗P < 0.01 and ∗∗∗P < 0.001 were considered significance of difference.

## 3 Results

### 3.1 PF dose-dependently improved constipation-related parameters and renal function in CKD mice

**FIGURE 2:**
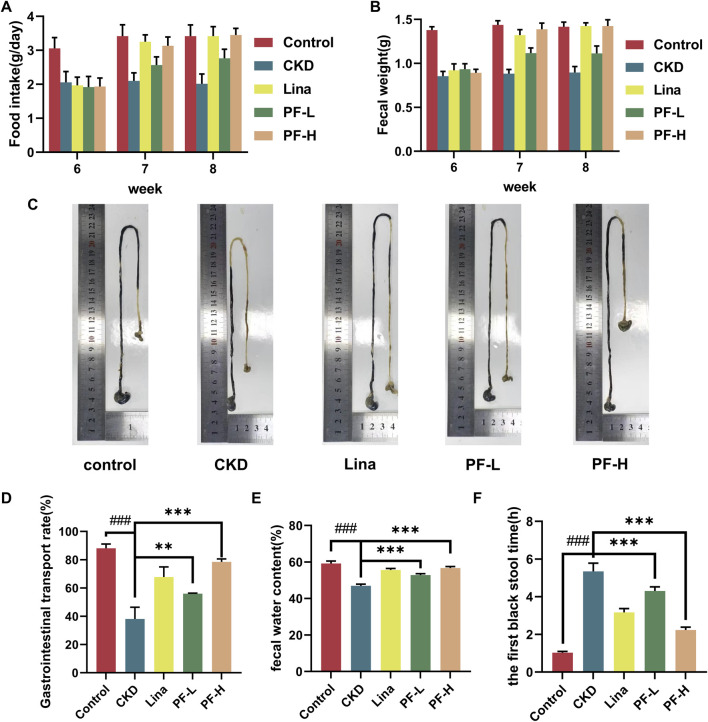
PF alleviated constipation-related symptoms in CKD mice. **(A)** Food intake during the experimental period. **(B)** Fecal weight. **(C)** Representative images of the first black stool. **(D)** Gastrointestinal transit ratio. **(E)** Fecal water content. **(F)** Time to the first black stool. Data are presented as mean ± SD (n = 6 per group). Statistical analysis was performed using one-way ANOVA followed by Bonferroni’s *post hoc* test. Compared with the control group, ##P < 0.01 and ###P < 0.001; compared with the CKD group, *P < 0.05, **P < 0.01, and ***P < 0.001.

To determine whether PF could ameliorate renal dysfunction in CKD mice, we first evaluated renal function and body weight in the adenine-induced CKD model. As shown in Figure 1B, mice fed a 0.2% adenine-containing diet developed typical features of CKD. Consistent with successful model establishment, serum creatinine and blood urea nitrogen (BUN) levels were markedly elevated in the CKD group compared with the control group (Figures 1C,D). PF treatment significantly reduced both serum creatinine and BUN levels in a dose-dependent manner. In addition, PF attenuated CKD-associated body weight loss during the experimental period. As shown in Figure 1E, no significant intergroup difference in body weight was observed at baseline before intervention. During the experimental period, body weight remained markedly lower in CKD mice than in control mice, whereas treatment with linaclotide and PF partially alleviated this reduction. We next assessed whether PF could improve constipation-related phenotypes in CKD mice after 14 consecutive days of treatment. As shown in Figure 2A, food intake was increased in the linaclotide (Lina), PF-L, and PF-H groups compared with the CKD group. In parallel, 24-h fecal weight was markedly reduced in the CKD group, confirming the successful establishment of constipation-like symptoms in CKD mice (Figure 2B). PF treatment dose-dependently increased fecal weight, and the effect of high-dose PF was comparable to that of linaclotide.

Consistent with these findings, fecal water content was reduced in CKD mice compared with control mice and was partially restored by linaclotide and PF treatment (Figure 2E). Intestinal transit was also impaired in CKD mice, as evidenced by representative images of the first black stool (Figure 2C), a reduced gastrointestinal transit ratio (Figure 2D), and prolonged time to the first black stool (Figure 2F). Treatment with linaclotide, PF-L, and PF-H improved these parameters to varying degrees, indicating enhanced intestinal motility. Collectively, these representative stool images and multiple functional indicators demonstrate that PF markedly alleviated constipation-related symptoms in CKD mice, as reflected by improvements in fecal weight, fecal water content, gastrointestinal transit ratio, and time to the first black stool. Taken together, PF exerted dose-dependent protective effects on both constipation-related phenotypes and renal dysfunction in CKD mice. Among the tested doses, the high dose of PF showed the most robust overall efficacy. Therefore, the high dose was selected for subsequent mechanistic analyses to further investigate the reno-intestinal protective actions of PF.

### 3.2 PF attenuated renal histopathological injury and fibrosis in CKD mice

**FIGURE 3:**
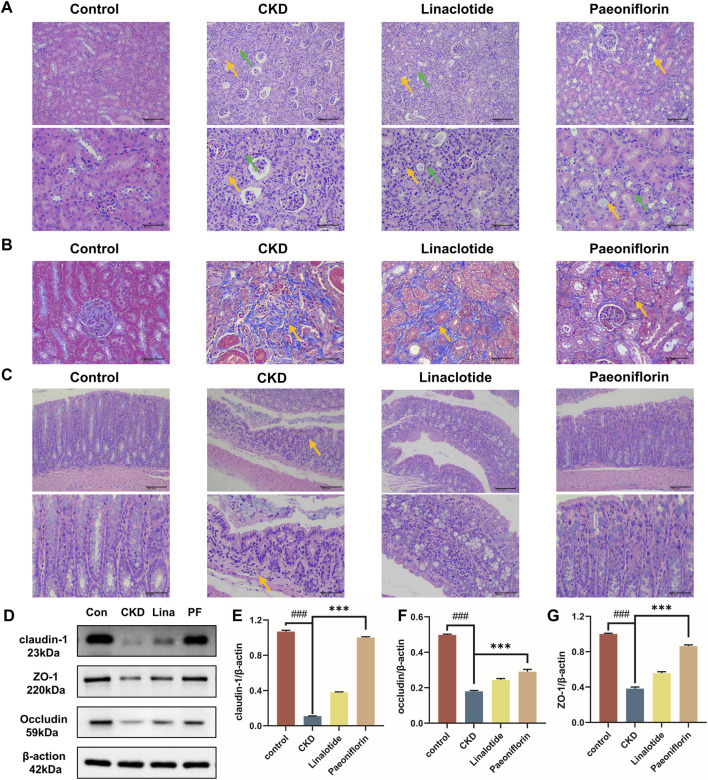
PF improved renal histopathological injury, fibrosis, and colonic barrier integrity in CKD mice. **(A)** Representative hematoxylin and eosin (H&E) staining images of renal tissues from the control, CKD, linaclotide, and paeoniflorin groups. Green arrows indicate dilated renal tubules, and yellow arrows indicate fibrotic lesions. Scale bars = 100 μm. **(B)** Representative Masson’s trichrome staining images of renal tissues. Yellow arrows indicate collagen fiber deposition. Scale bars = 100 μm. **(C)** Representative H&E staining images of distal colonic tissues. Yellow arrows indicate epithelial degeneration/necrosis and inflammatory cell infiltration. Scale bars = 100 μm. **(D)** Representative Western blot bands of claudin-1, occludin, ZO-1, and β-actin in distal colonic tissues. **(E–G)** Quantitative analysis of claudin-1 **(E)**, occludin **(F)**, and ZO-1 **(G)** protein expression levels. Data are presented as mean ± SD (n = 6 per group). Statistical analysis was performed using one-way ANOVA followed by Bonferroni’s *post hoc* test. Compared with the control group, ##P < 0.01 and ###P < 0.001; compared with the CKD group, *P < 0.05, **P < 0.01, and ***P < 0.001.

To further investigate the renoprotective effects of PF, renal histopathological changes and fibrosis were evaluated in subsequent experiments using high-dose PF. As shown in Figure 3A, hematoxylin and eosin (H&E) staining revealed marked renal histopathological injury in the CKD group, characterized by blurred tubular architecture, extensive fibrotic changes, and glomerular atrophy, compared with the control group. Linaclotide treatment did not substantially improve these renal pathological alterations. In contrast, high-dose PF markedly alleviated renal injury, as reflected by reduced tubular damage and less severe fibrotic lesions. Consistent with the H&E findings, Masson’s trichrome staining showed a significant increase in collagen fiber deposition in the kidneys of CKD mice compared with control mice (Figure 3B). This was accompanied by glomerular hypertrophy, basement membrane expansion, and increased extracellular matrix accumulation. Although the linaclotide group showed a decreasing trend in collagen deposition, the difference was not statistically significant. By contrast, high-dose PF significantly reduced the collagen fiber-positive area compared with the CKD group (P < 0.01), indicating that PF effectively attenuated renal fibrosis in CKD mice.

### 3.3 PF improved colonic mucosal injury and intestinal barrier integrity in CKD mice

We next assessed the effects of PF on colonic histopathology. As shown in Figure 3C, the distal colonic mucosa of CKD mice exhibited marked degeneration and necrosis, disorganized epithelial cell arrangement, and inflammatory cell infiltration compared with the control group. Linaclotide partially alleviated mucosal necrosis; however, substantial inflammatory cell infiltration remained evident in the mucosal and lamina propria layers. In contrast, high-dose PF markedly restored colonic mucosal architecture, with clearer tissue organization, near-normal epithelial morphology, and minimal inflammatory cell infiltration. These findings indicate that PF effectively ameliorated colonic tissue injury and inflammatory changes in CKD mice.

To further determine whether PF improved intestinal barrier integrity at the molecular level, we examined the expression of tight junction proteins in distal colonic tissues by Western blotting. As shown in Figures 3D–G, the expression levels of claudin, occludin, and ZO-1 were significantly downregulated in the CKD group compared with the control group. Linaclotide treatment induced only a modest increase in these proteins, without statistical significance. In contrast, high-dose PF significantly upregulated the expression of claudin, occludin, and ZO-1 (P < 0.05). Taken together, these results suggest that high-dose PF alleviated colonic barrier injury by restoring the expression of key tight junction proteins and improving intestinal epithelial barrier integrity.

### 3.4 PF-H increased colonic 5-HT content and was associated with modulation of TPH1/AHR-related signaling

3.4.1

**FIGURE 4:**
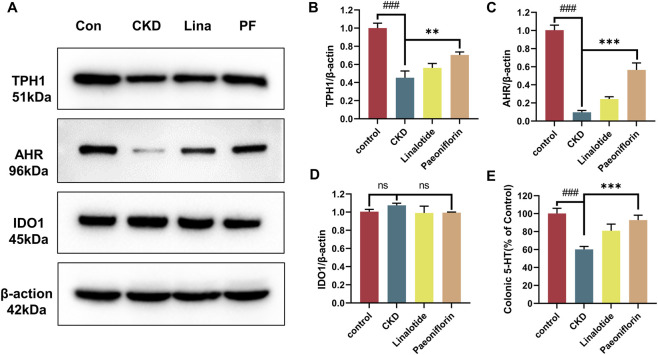
PF-H increased colonic 5-HT content and was associated with modulation of TPH1/AHR-related signaling. **(A)** Representative Western blot bands of TPH1, AHR, IDO1, and β-actin in colonic tissues from the control, CKD, linaclotide, and PF-H groups. **(B–D)** Quantitative analysis of TPH1 **(B)**, AHR **(C)**, and IDO1 **(D)** protein expression levels. **(E)** Colonic 5-HT content. Data are presented as mean ± SD (n = 6 per group). Statistical analysis was performed using one-way ANOVA followed by Bonferroni’s post hoc test. Compared with the control group, ##P < 0.01 and ###P < 0.001; compared with the CKD group, *P < 0.05, **P < 0.01, and ***P < 0.001.

To further investigate whether PF-H affected TPH1/AHR-related signaling in the colon, we examined the protein expression of TPH1, AHR, and IDO1, together with colonic 5-HT content. As shown in Figures 4A–D, the protein expression levels of TPH1 and AHR were markedly reduced in the CKD group compared with the control group, whereas PF-H treatment significantly increased the expression of both proteins. Linaclotide also showed a partial restorative effect. In contrast, no marked difference in IDO1 expression was observed among groups.

Consistent with these findings, colonic 5-HT content was significantly decreased in the CKD group compared with the control group and was restored after PF-H treatment (Figure 4E). Linaclotide also increased colonic 5-HT to some extent, although the effect was less evident than that observed in the PF-H group. Taken together, these results further support that PF-H was associated with modulation of TPH1/AHR-related signaling and increased colonic 5-HT availability, which may be relevant to the improvement of intestinal motility in CKD-associated constipation.

### 3.5 PF was associated with PCS-related metabolic alterations and molecular interactions with key enzymes

**FIGURE 5:**
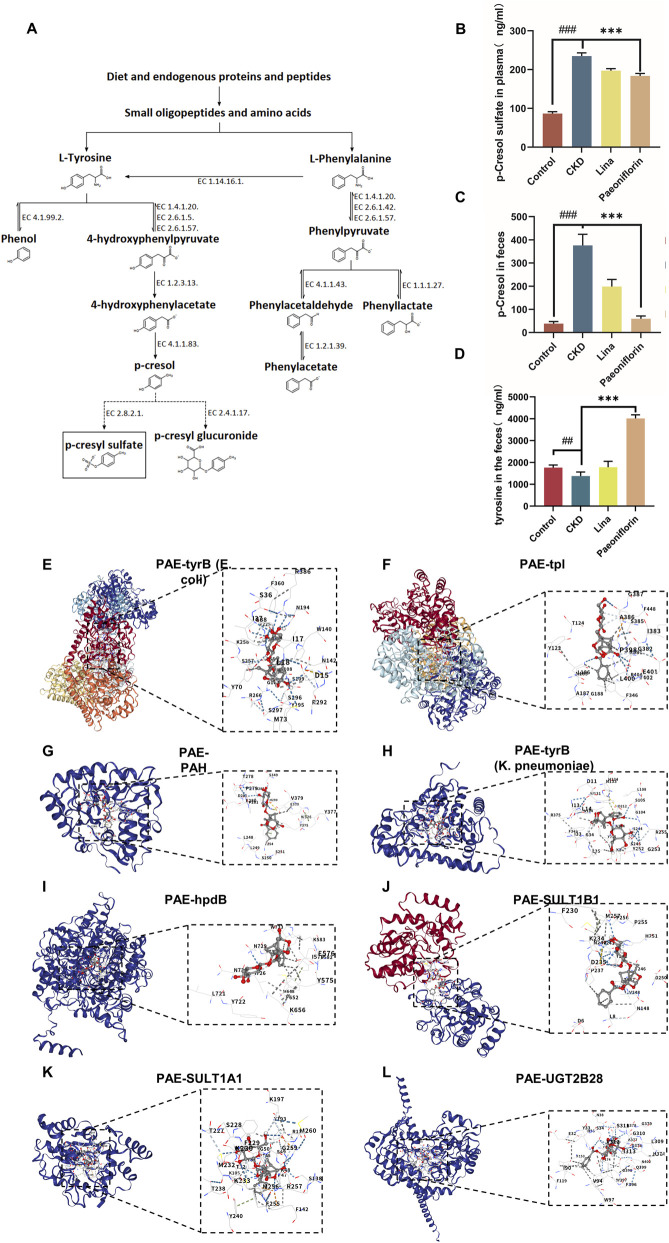
PF was associated with PCS-related metabolic alterations and molecular interactions with key enzymes. **(A)** Schematic overview of the tyrosine-p-cresol-PCS metabolic pathway, adapted from Gryp et al. (2017) under the Creative Commons Attribution License (CC BY 4.0). **(B)** Plasma p-cresol sulfate (PCS) levels. **(C)** Fecal p-cresol levels. **(D)** Fecal tyrosine levels. **(E)** Representative molecular docking model of PF with TyrB from Escherichia coli. **(F)** Representative molecular docking model of PF with TPL. **(G)** Representative molecular docking model of PF with PAH. **(H)** Representative molecular docking model of PF with TyrB from Klebsiella pneumoniae. **(I)** Representative molecular docking model of PF with HPDB. **(J)** Representative molecular docking model of PF with SULT1B1. **(K)** Representative molecular docking model of PF with SULT1A1. **(L)** Representative molecular docking model of PF with UGT2B28. Statistical analysis was performed using one-way ANOVA followed by Bonferroni’s post hoc test. Compared with the control group, ##P < 0.01 and ###P < 0.001; compared with the CKD group, *P < 0.05, **P < 0.01, and ***P < 0.001.

Because gut-derived uremic toxins are closely involved in gut-kidney axis dysfunction, we next examined whether PF affected PCS-related metabolic alterations in CKD mice with constipation. To this end, enzyme-linked immunosorbent assay (ELISA) was used to quantify metabolites associated with the tyrosine-p-cresol-PCS pathway in fecal and serum samples. As shown in Figure 5B, plasma PCS levels were markedly elevated in CKD mice compared with control mice. Consistently, fecal p-cresol levels were also increased in the CKD group (Figure 5C). In contrast, fecal tyrosine levels did not show the same pattern and appeared to be higher in PF-treated groups, particularly in the PF-L and PF-H groups (Figure 5D). These findings suggest that CKD was associated with enhanced PCS-related metabolic burden, whereas PF treatment may have altered metabolite profiles along the tyrosine-p-cresol-PCS pathway. After 2 weeks of treatment, linaclotide and PF reduced plasma PCS and fecal p-cresol levels to varying degrees (Figures 5B,C). Among the PF-treated groups, high-dose PF showed a greater downward trend in these metabolites than the CKD group, although the extent and statistical significance of these changes should be interpreted according to the quantitative analysis. In parallel, fecal tyrosine levels were increased in PF-treated mice (Figure 5D), suggesting that PF may have influenced metabolic flux along the tyrosine-p-cresol-PCS pathway.

To further explore the potential interaction between PF and key enzymes involved in PCS-related metabolism, molecular docking analysis was performed. A schematic overview of the tyrosine-p-cresol-PCS pathway is shown in Figure 5A. Phenylalanine hydroxylase (PAH), tyrosine aminotransferase B (TyrB), beta-tyrosine lyase (TPL), p-hydroxyphenylacetate decarboxylase (HPDB), sulfotransferase 1B1 (SULT1B1), sulfotransferase 1A1 (SULT1A1), and UDP-glucuronosyltransferase 2B28 (UGT2B28) were selected as candidate targets based on their reported roles in this pathway. The crystal structures of these enzymes were retrieved from the Protein Data Bank and subjected to molecular docking analysis. As shown in Figures 5E–L, PF exhibited favorable binding patterns with several key enzymes involved in PCS-related metabolism, including TyrB from *Escherichia coli* (Figure 5E), TPL (Figure 5F), PAH (Figure 5G), TyrB from *Klebsiella pneumoniae* (Figure 5H), HPDB (Figure 5I), SULT1B1 (Figure 5J), SULT1A1 (Figure 5K), and UGT2B28 (Figure 5L). Two-dimensional interaction analysis suggested that PF formed multiple hydrophilic interactions within the active pockets of these enzymes, supporting the possibility of stable intermolecular binding.

Overall, these findings suggest that PF was associated with PCS-related metabolic alterations and may interact with key enzymes involved in the tyrosine-p-cresol-PCS pathway. However, the mechanistic relevance of these interactions requires further experimental validation.

### 3.6 PF selectively remodeled gut microbial composition in CKD-associated constipation

**FIGURE 6:**
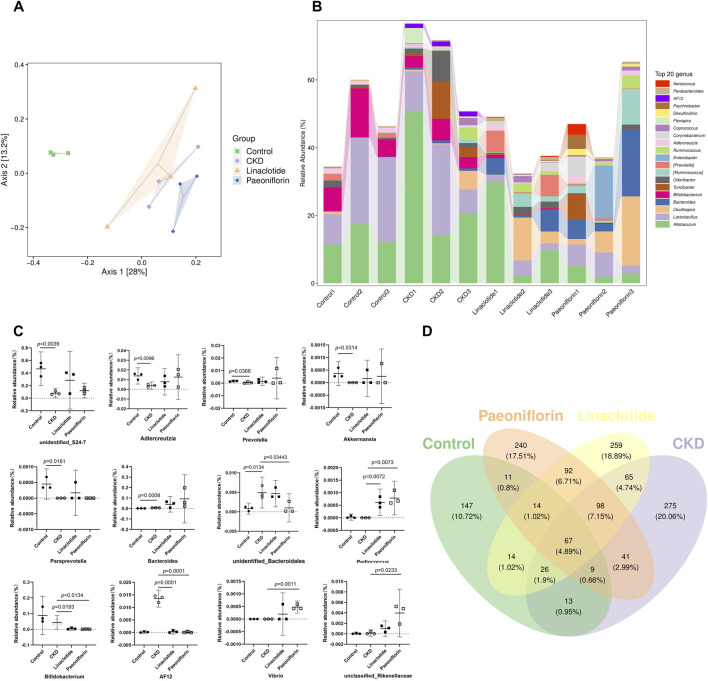
PF selectively remodeled gut microbial composition in CKD-associated constipation. **(A)** Alpha diversity analysis of gut microbiota among the Control, CKD, Linaclotide, and Paeoniflorin groups using Chao1, Simpson, and Shannon indices. **(B)** Relative abundance of the top 20 genera in each sample at the genus level. **(C)** Differential abundance analysis of selected bacterial genera among groups. **(D)** Venn diagram showing shared and unique OTUs/ASVs among the Control, CKD, Linaclotide, and Paeoniflorin groups. Data are presented as mean ± SD. Statistical analysis was performed using one-way ANOVA followed by Bonferroni’s post hoc test. Compared with the control group, ^##^P < 0.01 and ^###^P < 0.001; compared with the CKD group, *P < 0.05, **P < 0.01, and ***P < 0.001.

To determine whether the protective effects of PF were associated with alterations in the gut microbiota, fecal samples collected at sacrifice after the 14-day treatment period were analyzed by 16S rRNA sequencing. Alpha diversity was first assessed using the Chao1, Simpson, and Shannon indices. No significant differences in these indices were observed among the Control, CKD, Linaclotide, and Paeoniflorin groups, suggesting that PF did not markedly alter the overall richness or diversity of the gut microbial community (Figure 6A). However, genus-level compositional analysis demonstrated marked microbial dysbiosis in CKD mice, whereas PF treatment partially reversed these alterations (Figure 6B). In particular, PF modulated the relative abundance of several representative genera that were disrupted under CKD conditions, indicating a selective corrective effect on microbial composition (Figure 6C). Moreover, the microbial profile of the PF-treated group appeared closer to that of the control group than to that of the CKD group. A Venn diagram of shared and unique OTUs/ASVs among groups is shown in Figure 6D. Together, these results indicate that PF selectively remodeled gut microbial composition without significantly changing overall alpha diversity, which may contribute to the improvement of intestinal dysfunction and renal injury.

### 3.7 PF-associated microbial functional changes were linked to amino acid metabolism and inflammatory signaling

**FIGURE 7:**
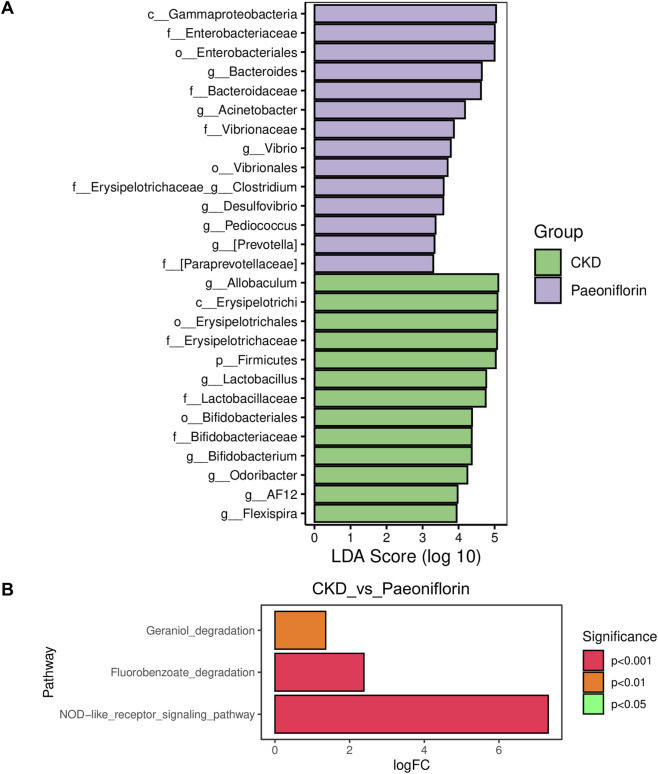
PF-associated microbial functional changes were linked to amino acid metabolism and inflammatory signaling. **(A)** LEfSe analysis showing discriminative microbial taxa in the CKD versus PF treatment group comparison. **(B)** KEGG pathway prediction analysis showing significantly altered pathways in the CKD versus PF treatment group comparison. Statistical analysis was performed using one-way ANOVA followed by Bonferroni’s *post hoc* test. Compared with the control group, ##P < 0.01 and ###P < 0.001; compared with the CKD group, *P < 0.05, **P < 0.01, and ***P < 0.001.

To further explore the potential functional implications of PF-mediated microbial remodeling, LEfSe and KEGG pathway prediction analyses were performed. To improve focus and clarity, we concentrated on the most relevant comparison, namely, the CKD versus PF treatment group. LEfSe analysis identified distinct discriminatory microbial taxa between these two groups (Figure 7A), further supporting the observation that PF selectively regulated gut microbial composition in CKD-associated constipation. KEGG pathway prediction analysis further suggested that PF-associated microbial changes were linked to pathways related to metabolism and inflammatory signaling (Figure 7B).

### 3.8 PF suppressed NLRP3/GSDMD-mediated pyroptosis and improved renal transporter expression in CKD mice

**FIGURE 8:**
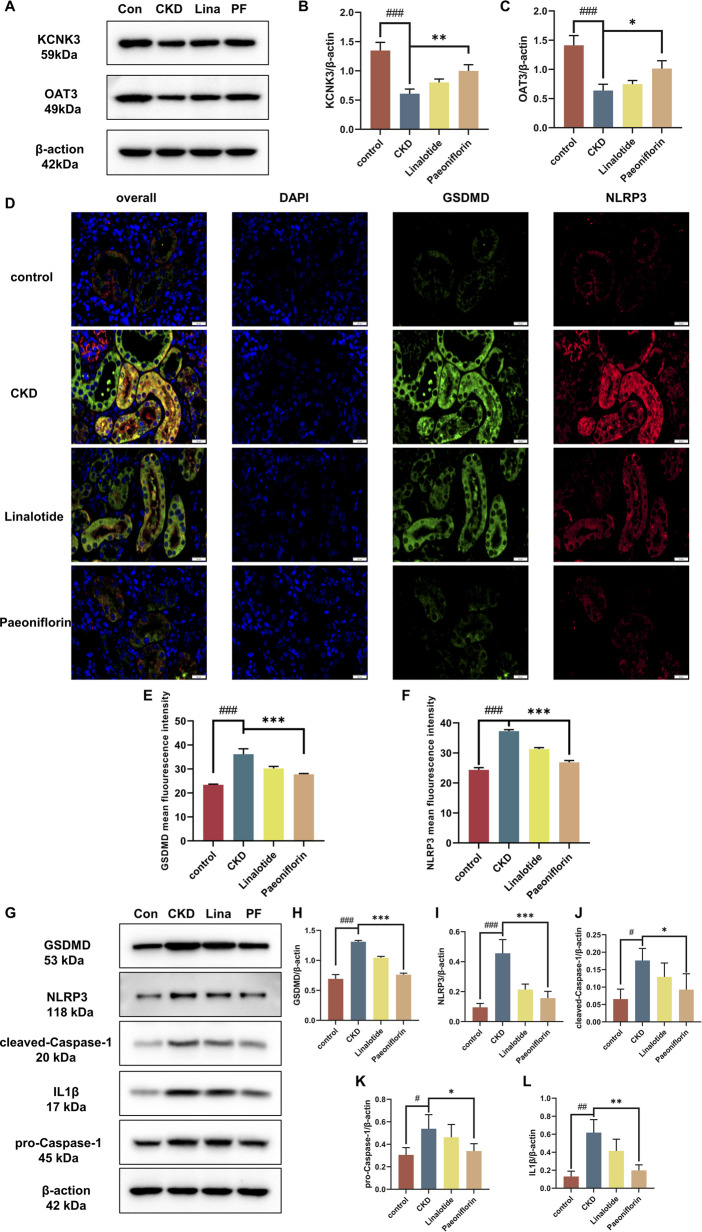
PF suppressed NLRP3/GSDMD-mediated pyroptosis and improved renal transporter expression in CKD mice. **(A)** Representative Western blot bands of KCNK3, OAT3, and β-actin in renal tissues from the control, CKD, linaclotide, and paeoniflorin groups. **(B,C)** Quantitative analysis of KCNK3 **(B)** and OAT3 **(C)** protein expression levels. **(D)** Representative immunofluorescence staining of renal tissues showing merged images, DAPI-stained nuclei (blue), GSDMD (green), and NLRP3 (red) in the control, CKD, linaclotide, and paeoniflorin groups. Scale bars = 50 μm. **(E,F)** Semi-quantitative analysis of GSDMD **(E)** and NLRP3 **(F)** fluorescence intensity. **(G)** Representative Western blot bands of GSDMD, NLRP3, cleaved caspase-1, IL-1β, pro-caspase-1, and β-actin in renal tissues **(H–L)** Quantitative analysis of GSDMD **(H)**, NLRP3 **(I)**, cleaved caspase-1 **(J)**, pro-caspase-1 **(K)**, and IL-1β **(L)** protein expression levels. Data are presented as mean ± SD (n = 6 per group). Statistical analysis was performed using one-way ANOVA followed by Bonferroni’s *post hoc* test. Compared with the control group, ##P < 0.01 and ###P < 0.001; compared with the CKD group, *P < 0.05, **P < 0.01, and ***P < 0.001.

Based on the above findings, we next investigated whether PF affected renal transporter expression and pyroptosis-related signaling in CKD mice. Because renal handling of gut-derived metabolites is closely associated with kidney injury, and their excretion involves transporters such as KCNK3 and OAT3, we first examined the expression of these two proteins. As shown in Figures 8A–C, compared with the control group, the protein expression levels of KCNK3 and OAT3 were significantly decreased in the CKD group. Relative to the CKD group, KCNK3 expression was increased in the linaclotide group, whereas OAT3 expression did not show a statistically significant change. In contrast, high-dose PF significantly upregulated the expression of both KCNK3 and OAT3, suggesting that PF may alleviate renal transporter dysfunction in CKD mice.

We next performed immunofluorescence staining to examine the localization and expression of the core pyroptosis-related proteins GSDMD and NLRP3 in renal tissues. As shown in Figure 8D, CKD markedly increased the fluorescence signals of GSDMD and NLRP3 in renal tubular epithelial cells compared with the control group, indicating activation of the NLRP3/GSDMD pyroptosis pathway. In contrast, these fluorescence signals were attenuated after linaclotide and PF treatment, with a more evident reduction observed in the PF-treated group. Semi-quantitative analysis further confirmed that the mean fluorescence intensities of GSDMD and NLRP3 were significantly increased in the CKD group and reduced after treatment (Figures 8E,F).

To further validate these findings, we examined the expression of pyroptosis-related proteins by Western blotting. As shown in Figures 8G–L, compared with the control group, the protein expression levels of GSDMD, NLRP3, cleaved caspase-1, and IL-1β were increased in the CKD group, whereas pro-caspase-1 expression showed no marked change. Relative to the CKD group, both linaclotide and high-dose PF reduced the expression of NLRP3, GSDMD, and IL-1β, while cleaved caspase-1 was more evidently decreased in the PF-treated group. No statistically significant differences in pro-caspase-1 expression were observed among groups, suggesting that the intervention primarily affected caspase-1 activation rather than total pro-caspase-1 expression. These findings were consistent with the immunofluorescence results and indicate that high-dose PF attenuated renal pyroptosis-related signaling in CKD mice.

## 4 Discussion

Paeoniflorin (PF) demonstrated clear therapeutic effects in adenine-induced CKD mice with constipation. In the present study, PF improved constipation-related phenotypes, including food intake, fecal weight, fecal water content, intestinal transit, and gastrointestinal motility, while simultaneously ameliorating renal dysfunction, as reflected by reduced serum creatinine and BUN levels. Histopathological analyses further showed that PF attenuated renal tubular injury and collagen deposition, restored colonic mucosal architecture, and upregulated the expression of tight junction proteins. These data indicate that PF exerts coordinated protective effects on both intestinal and renal injury in CKD-associated constipation, rather than acting solely as a prokinetic agent.

This overall pattern is consistent with the current understanding of the gut-kidney axis in CKD. CKD is increasingly recognized as a systemic disorder in which impaired renal function is accompanied by gastrointestinal dysmotility, barrier impairment, gut microbial dysbiosis, and metabolite-related disturbances that reinforce one another (Hayeeawaema et al., 2023). Experimental studies using adenine-induced CKD models have likewise shown that renal injury can coexist with intestinal dysfunction and microbial imbalance, supporting the biological plausibility of assessing reno-intestinal protection as an integrated outcome rather than as isolated organ effects. One of the most robust findings of the present study is the protective effect of PF on intestinal barrier integrity. CKD-associated constipation in our model was accompanied by colonic mucosal damage and reduced expression of claudin, occludin, and ZO-1, whereas high-dose PF substantially restored mucosal architecture and increased the expression of these tight junction proteins. This finding is mechanistically important because epithelial barrier disruption can enhance the translocation of luminal inflammatory stimuli and microbial products, thereby aggravating both intestinal dysfunction and extraintestinal organ injury. Previous work has shown that PF protects epithelial barrier function and suppresses inflammatory injury in intestinal epithelial models (Wu et al., 2019). Taken together, these observations suggest that preservation of intestinal barrier integrity may represent a central component of the reno-intestinal protective effects of PF.

Our microbiota data further support this interpretation. PF did not simply normalize all microbial features indiscriminately; rather, it selectively remodeled gut microbial composition. Although alpha diversity indices did not differ significantly among groups, genus-level compositional analysis indicated that PF partially reversed CKD-associated dysbiosis. This selective remodeling pattern is noteworthy because recent studies have increasingly emphasized that the gut-kidney axis should be understood as an integrated network involving intestinal barrier injury, microbial community restructuring, microbe-derived metabolite imbalance, and host inflammatory responses, all of which may jointly influence renal injury progression (Ou et al., 2026a; Liu et al., 2025; Ou et al., 2026b). In this context, our results are best understood as evidence that PF may improve CKD-associated constipation partly through targeted remodeling of gut microbial composition within a broader reno-intestinal regulatory framework. However, the KEGG pathway changes in the present study were inferred from 16 S rRNA-based functional prediction, which is inherently indirect and cannot accurately represent the actual metabolic activity of the microbial community or the dynamic variation of host-microbe co-metabolites. Recent studies have likewise emphasized that predicted microbial functional shifts should be interpreted with caution and require further validation by higher-resolution approaches, such as shotgun metagenomic sequencing and targeted metabolomics (Wang et al., 2026; Chang et al., 2025). In particular, future studies should directly assess relevant metabolites, including short-chain fatty acids (SCFAs), tryptophan-related metabolites, and PCS-related precursor metabolites, to better define the biological significance of the predicted functional changes. The TPH1/AHR-related findings in this study provide a plausible mechanistic bridge between microbial remodeling and host intestinal physiology, but they should be interpreted with appropriate caution. We found that PF increased colonic TPH1 and AHR expression, suggesting modulation of signaling pathways relevant to serotonin production, intestinal motility, and mucosal homeostasis. The biological plausibility of this observation is supported by evidence that serotonin is a key regulator of intestinal homeostasis and motility (Koopman et al., 2021). In parallel, AHR is now recognized as an important mediator of intestinal barrier function and mucosal immune balance, particularly in response to diet- and microbiota-derived ligands (Stockinger et al., 2021). However, because downstream metabolites of the relevant tryptophan pathways were not directly quantified in our study, these data are better interpreted as evidence of TPH1/AHR-related signaling modulation rather than definitive restoration of the full tryptophan metabolic network.

Compared with the TPH1/AHR axis, the evidence for anti-inflammatory effects in the kidney is relatively stronger. In our study, CKD markedly increased the renal expression of NLRP3, GSDMD, cleaved caspase-1, and IL-1β, whereas PF significantly reduced these changes and attenuated both the renal fluorescence signals and the semi-quantitative fluorescence intensities of GSDMD and NLRP3. These findings indicate that PF suppresses renal pyroptosis-related signaling in CKD mice. This result is relevant because the uremic milieu and gut-derived toxins have been linked to dysregulated inflammasome-related inflammatory responses in CKD (Ho et al., 2021). In addition, PF improved the renal expression of KCNK3 and OAT3, suggesting partial recovery of renal transporter dysfunction. Taken together, these results support a model in which PF attenuates renal injury through combined suppression of pyroptosis-related signaling and improvement of renal metabolite-handling capacity.

The PCS-related findings should be interpreted more cautiously than the histological, barrier, or pyroptosis data. In classical CKD literature, p-cresyl sulfate is commonly discussed as a gut-derived uremic toxin that accumulates as renal excretory function declines. However, our current data do not support a strong conclusion that PF definitively reduced PCS burden. Instead, PF was associated with alterations in metabolites linked to the tyrosine-p-cresol-PCS pathway, and molecular docking suggested potential interactions with several key enzymes involved in this route. Thus, the present results are more appropriately framed as PCS-related metabolic alterations rather than direct proof that PF suppresses PCS production or accumulation. In the same way, the docking analysis should be viewed as supportive evidence for possible molecular interactions, not as definitive proof of direct enzymatic inhibition. Further targeted metabolomics and enzyme activity assays will be required to determine whether PF directly modulates flux through the tyrosine-p-cresol-PCS pathway and whether such modulation is biologically relevant to the renoprotective effect observed here. Taken together, the present findings support a layered mechanistic interpretation. At the intestinal level, PF improved bowel function, restored mucosal architecture, and increased tight junction protein expression. At the microbial level, PF selectively reshaped community composition and was associated with predicted functional changes related to amino acid metabolism and inflammatory signaling. At the host signaling level, PF modulated TPH1/AHR-related signaling and suppressed renal NLRP3/GSDMD-mediated pyroptosis. At the renal level, PF improved renal function, attenuated fibrosis, and restored transporter expression. Therefore, rather than acting through a single dominant target, PF appears to exert integrated protective effects across multiple levels of the gut-kidney axis. Several limitations should also be acknowledged. First, the microbial functional changes were inferred from 16 S rRNA sequencing rather than directly measured by shotgun metagenomics or targeted metabolomics. Second, although PF was associated with TPH1/AHR-related signaling changes, downstream metabolites of tryptophan metabolism were not directly quantified, limiting mechanistic interpretation at the metabolic level. Third, although PF was associated with PCS-related metabolic alterations and potential interactions with key enzymes, the biochemical significance of these findings remains uncertain. Fourth, the contribution of specific microbial taxa to intestinal barrier repair, TPH1/AHR-related signaling, PCS-related changes, and renal pyroptosis was not directly tested. Future studies should therefore adopt more specific mechanistic strategies. In particular, fecal microbiota transplantation experiments will be necessary to determine whether the protective effects of PF are dependent on gut microbial remodeling. In addition, rescue or blocking experiments using TPH1 inhibitors or AHR antagonists will help clarify whether TPH1/AHR-related signaling is required for the intestinal effects of PF. Furthermore, cell-based studies using renal tubular epithelial cells and intestinal epithelial cells will be valuable to determine whether PF directly suppresses NLRP3 inflammasome activation. Together with targeted metabolomics and enzyme activity assays, these approaches will help define the microbiota-dependent and metabolite-dependent basis of PF action more precisely.

Overall, our data suggest that PF alleviates CKD-associated constipation and ameliorates renal injury through integrated modulation of intestinal barrier integrity, gut microbial composition, TPH1/AHR-related signaling, renal transporter expression, and pyroptosis-related inflammatory signaling. These findings strengthen the rationale for further investigation of PF as a potential therapeutic candidate for CKD-associated constipation and related gut-kidney axis disorders. The future validation strategies proposed above may also help improve the translational relevance of PF by clarifying whether its effects depend on microbiota remodeling, TPH1/AHR-related signaling, and direct suppression of inflammasome activation.

## 5 Conclusion

Collectively, this study demonstrates that PF alleviated CKD-associated constipation and ameliorated renal injury in mice. These protective effects may be attributed, at least in part, to integrated modulation of intestinal barrier integrity, gut microbial composition, TPH1/AHR-related signaling, and renal NLRP3/GSDMD-mediated pyroptosis along the gut-kidney axis. These findings suggest that PF may represent a promising pharmacological candidate for the treatment of CKD-associated constipation.

## Data Availability

The data presented in the study are deposited in the NCBI repository, accession number PRJNA1482327.
