# Supplementary figures and images for: Paeoniflorin alleviates CKD-associated constipation by modulating TPH1/AHR-related signaling and suppressing NLRP3/GSDMD-mediated pyroptosis (part 1 of 2)
Source: Front Pharmacol. 2026 Jul 10;17:1844043. doi: 10.3389/fphar.2026.1844043 (PMC13396008; doi:10.3389/fphar.2026.1844043)

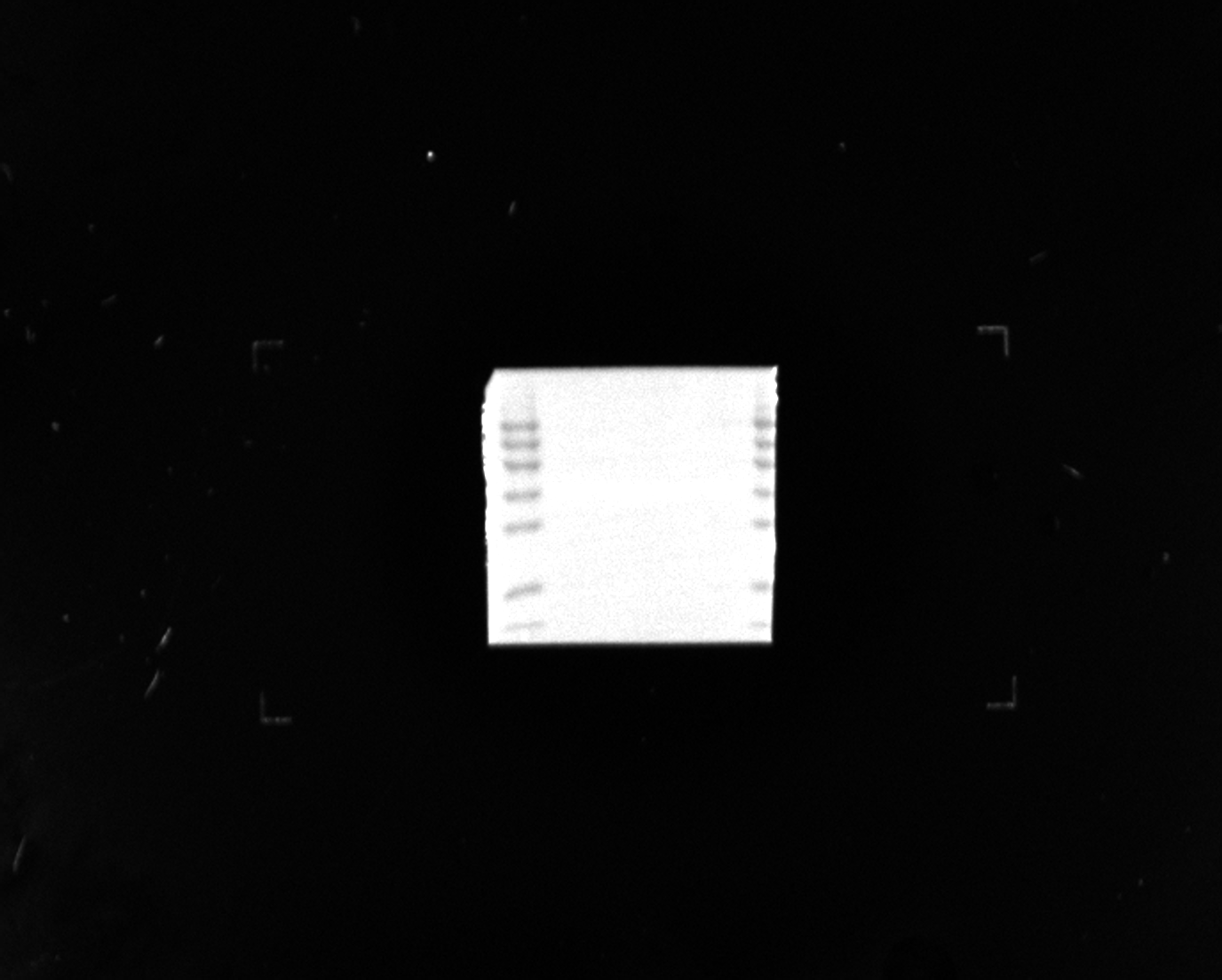

Supplement: Supplementary file 1 [file DataSheet3.zip › 原图1/Caspase1/1-t.Tif]

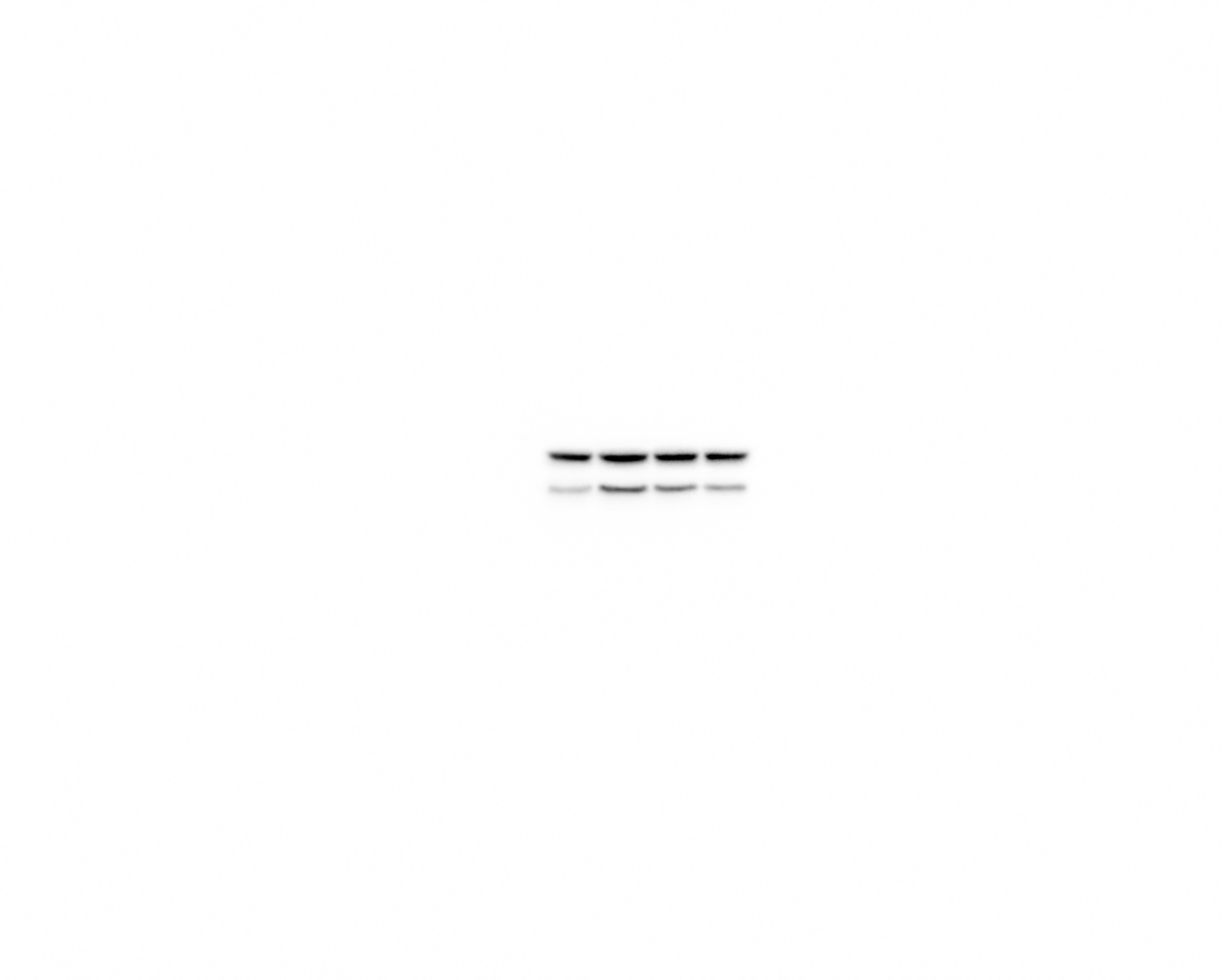

Supplement: Supplementary file 1 [file DataSheet3.zip › 原图1/Caspase1/1.Tif]

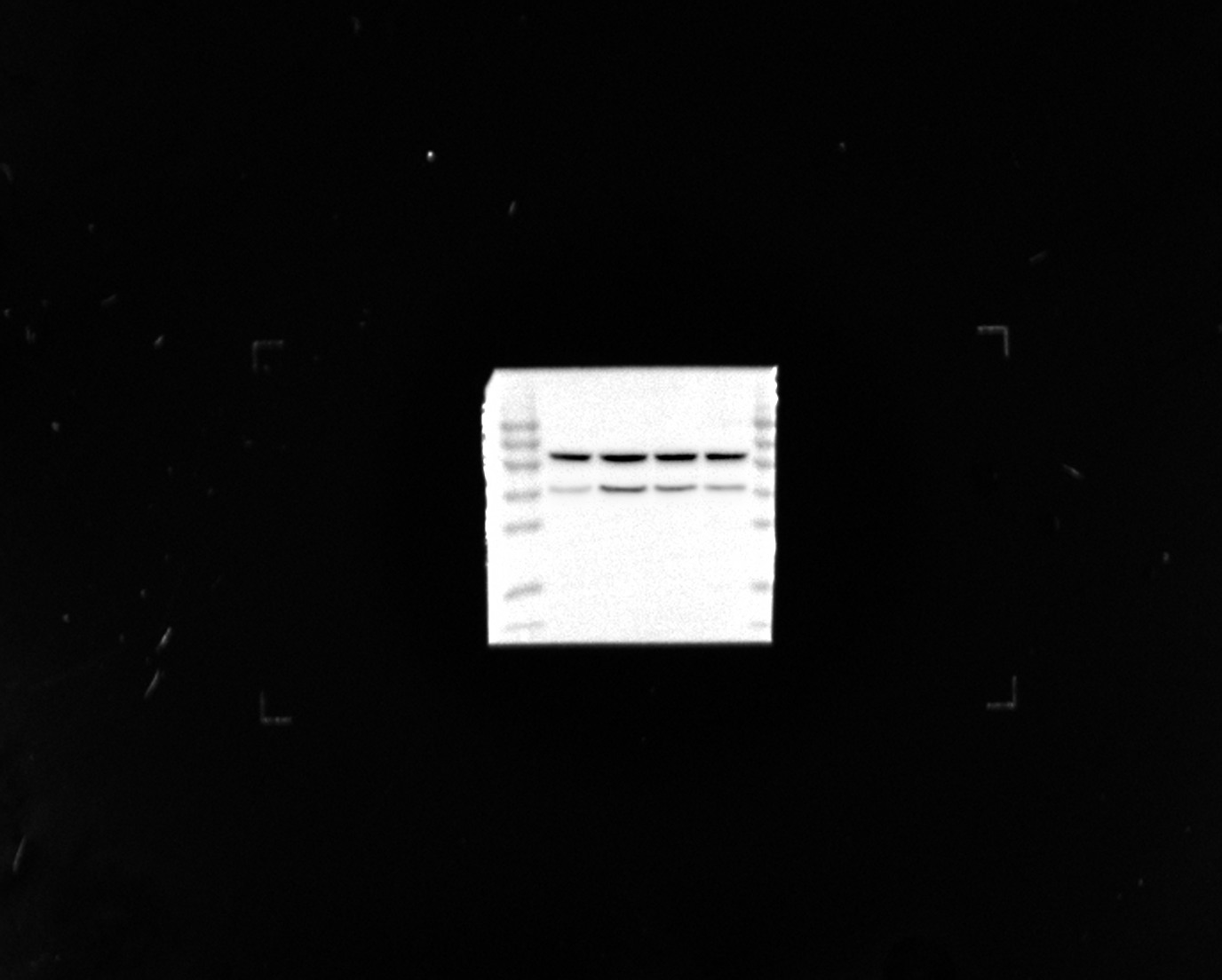

Supplement: Supplementary file 1 [file DataSheet3.zip › 原图1/Caspase1/1副本.tif]

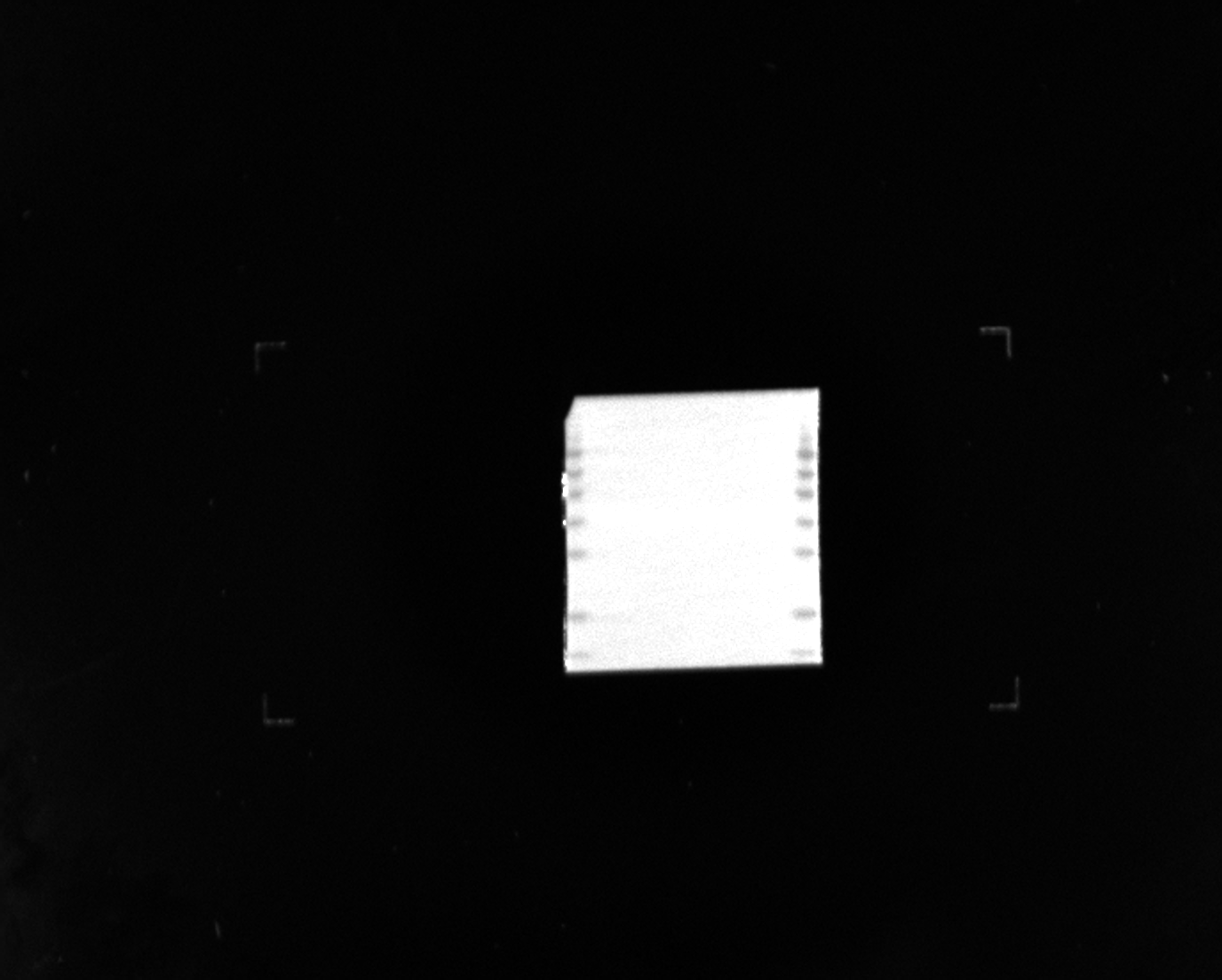

Supplement: Supplementary file 1 [file DataSheet3.zip › 原图1/Caspase1/2-t.Tif]

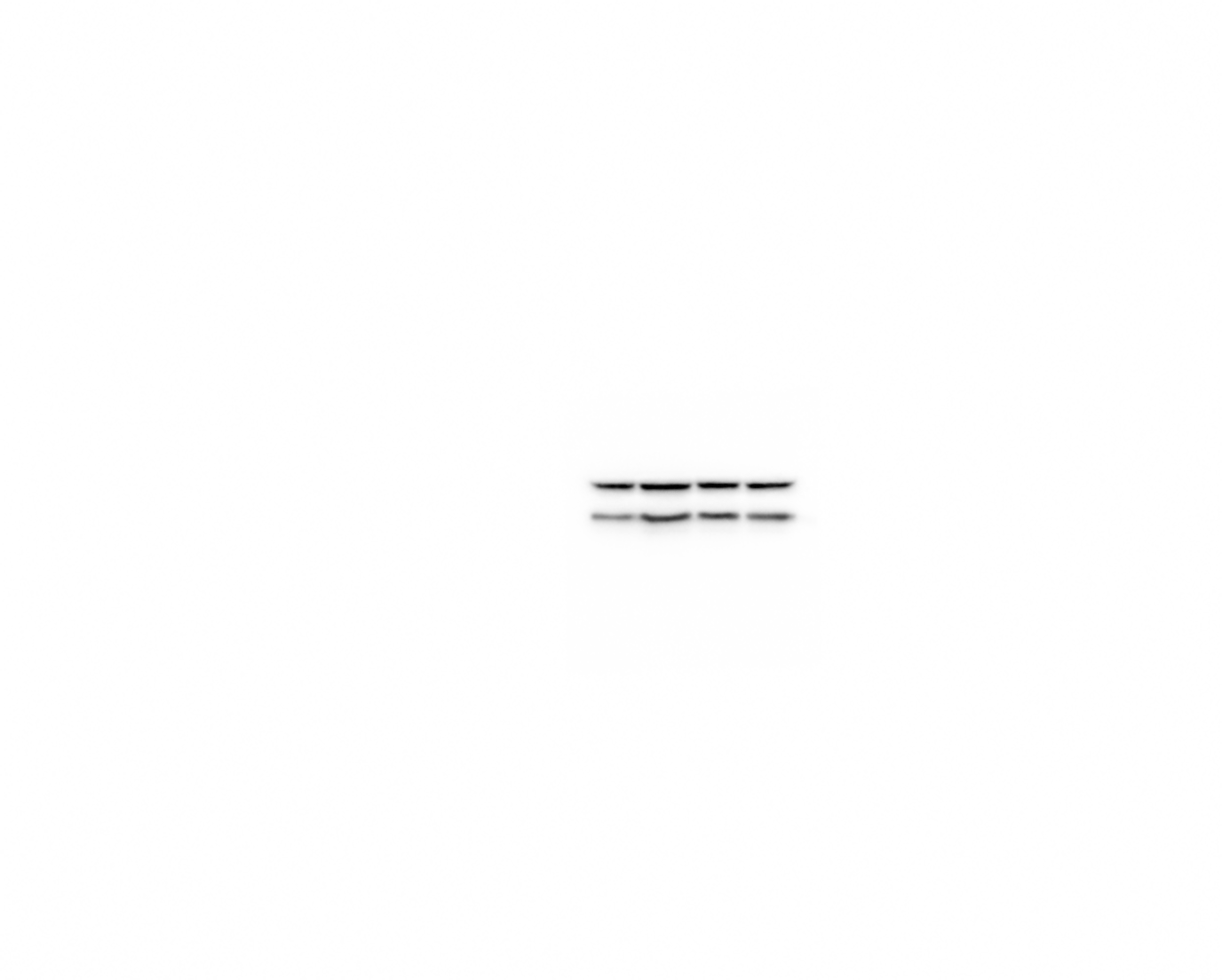

Supplement: Supplementary file 1 [file DataSheet3.zip › 原图1/Caspase1/2.Tif]

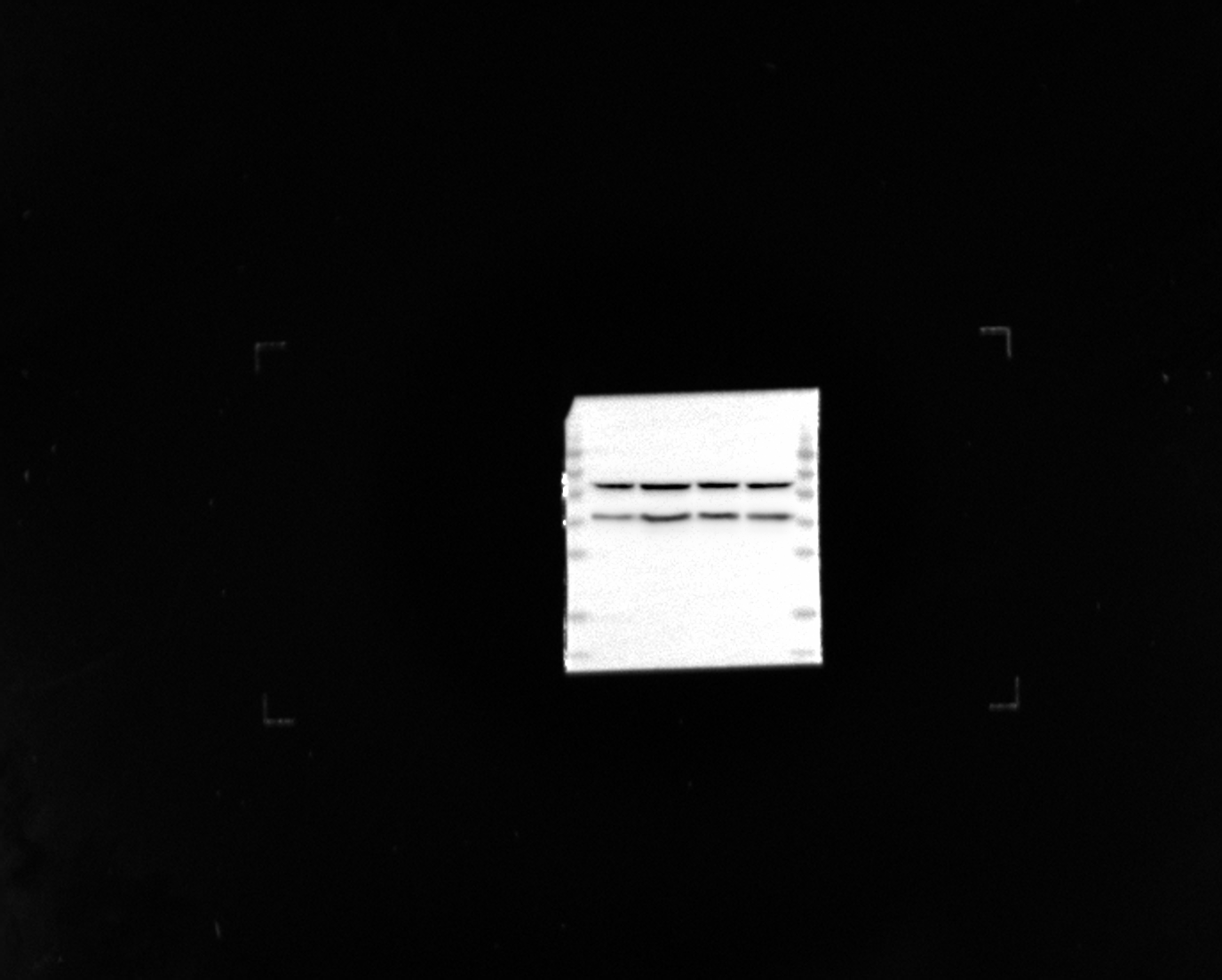

Supplement: Supplementary file 1 [file DataSheet3.zip › 原图1/Caspase1/2副本.tif]

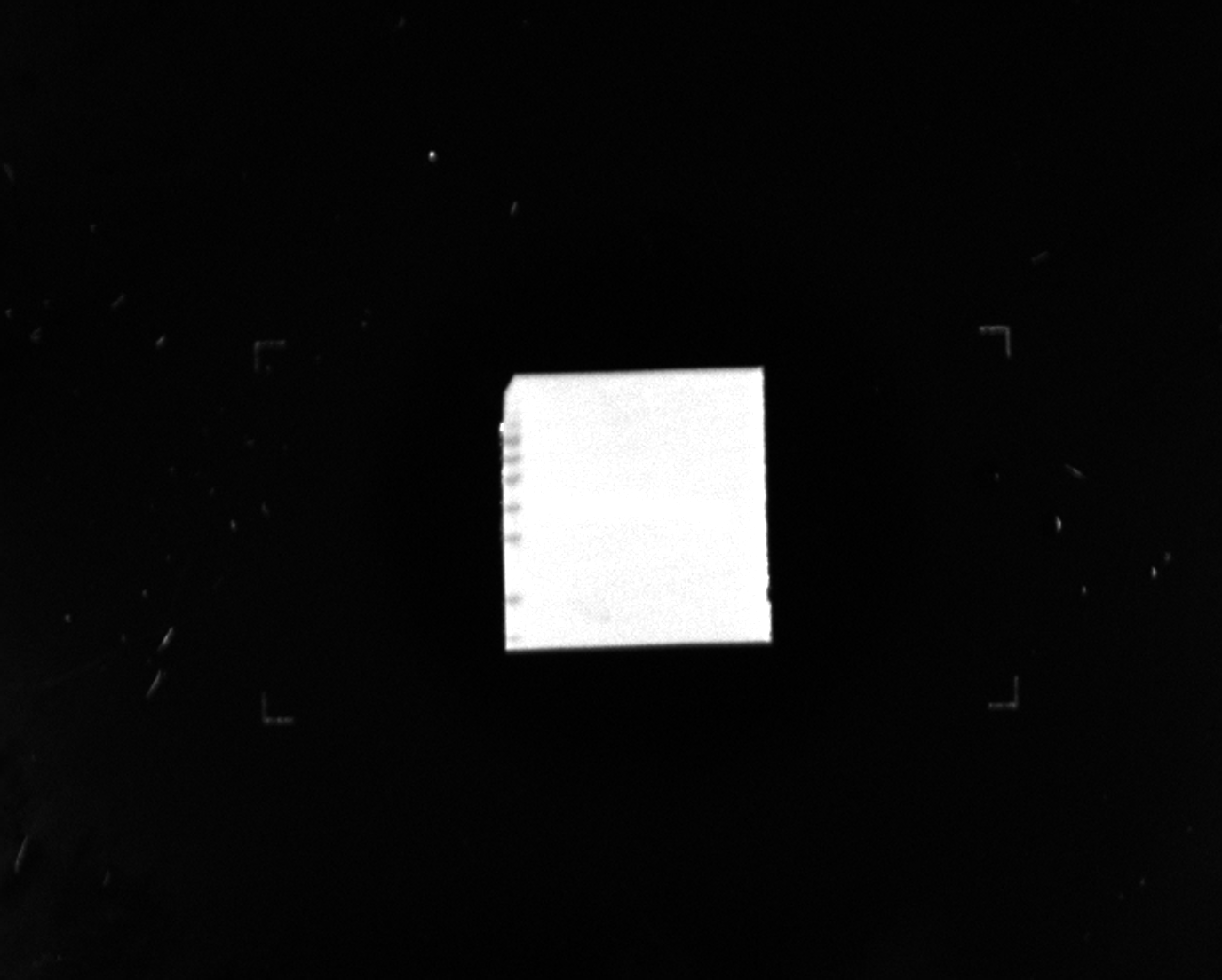

Supplement: Supplementary file 1 [file DataSheet3.zip › 原图1/Caspase1/3-t.Tif]

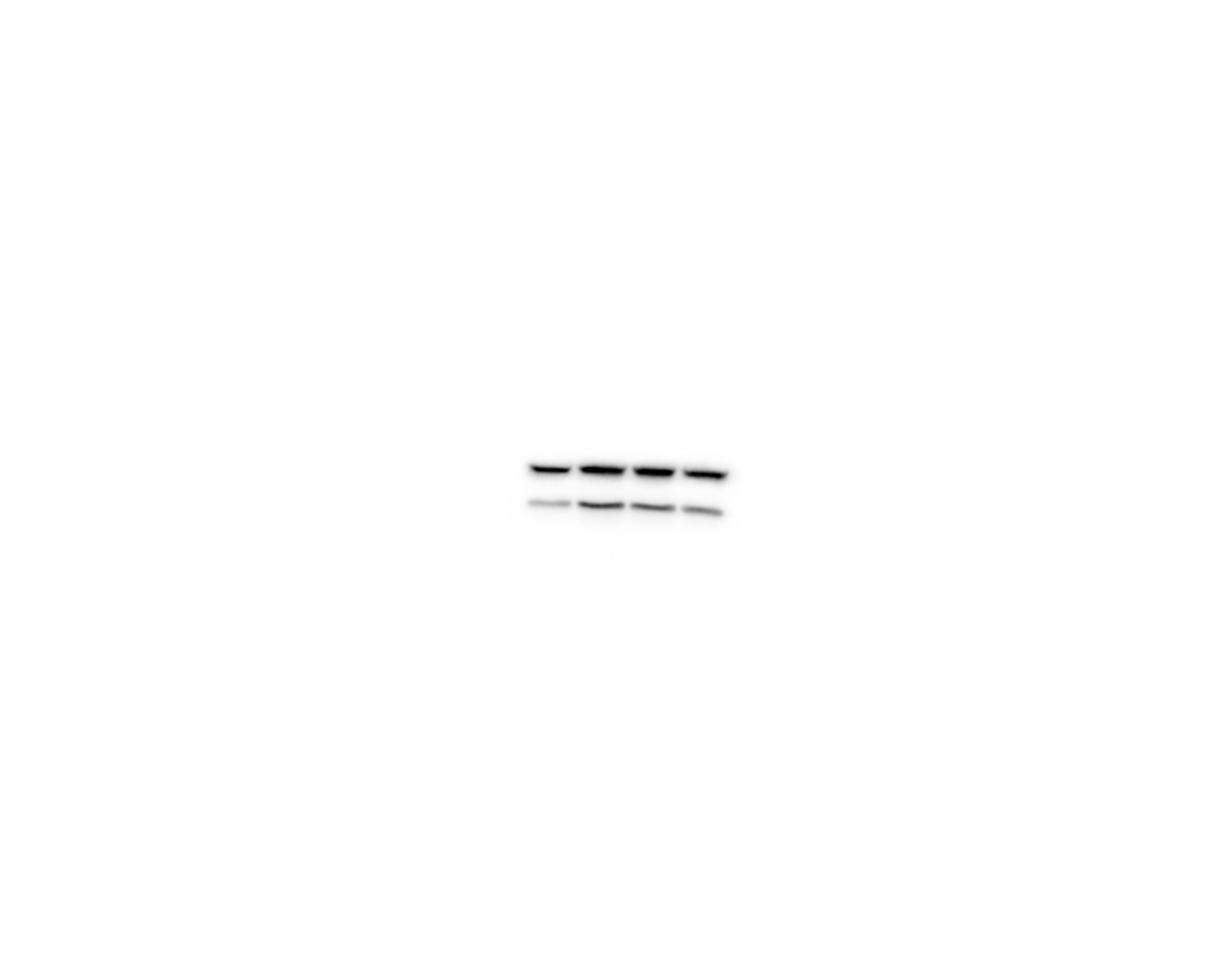

Supplement: Supplementary file 1 [file DataSheet3.zip › 原图1/Caspase1/3.Tif]

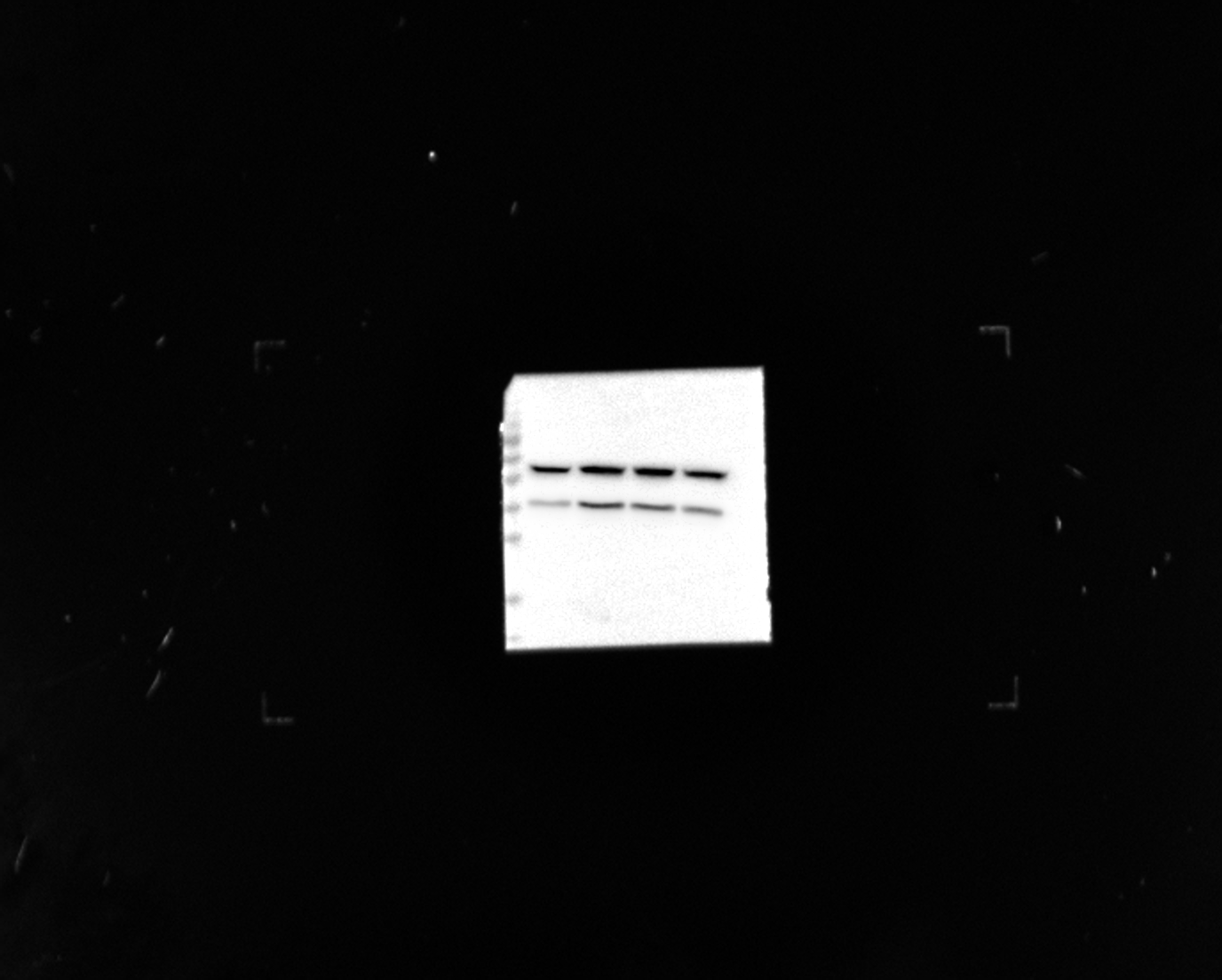

Supplement: Supplementary file 1 [file DataSheet3.zip › 原图1/Caspase1/3副本.tif]

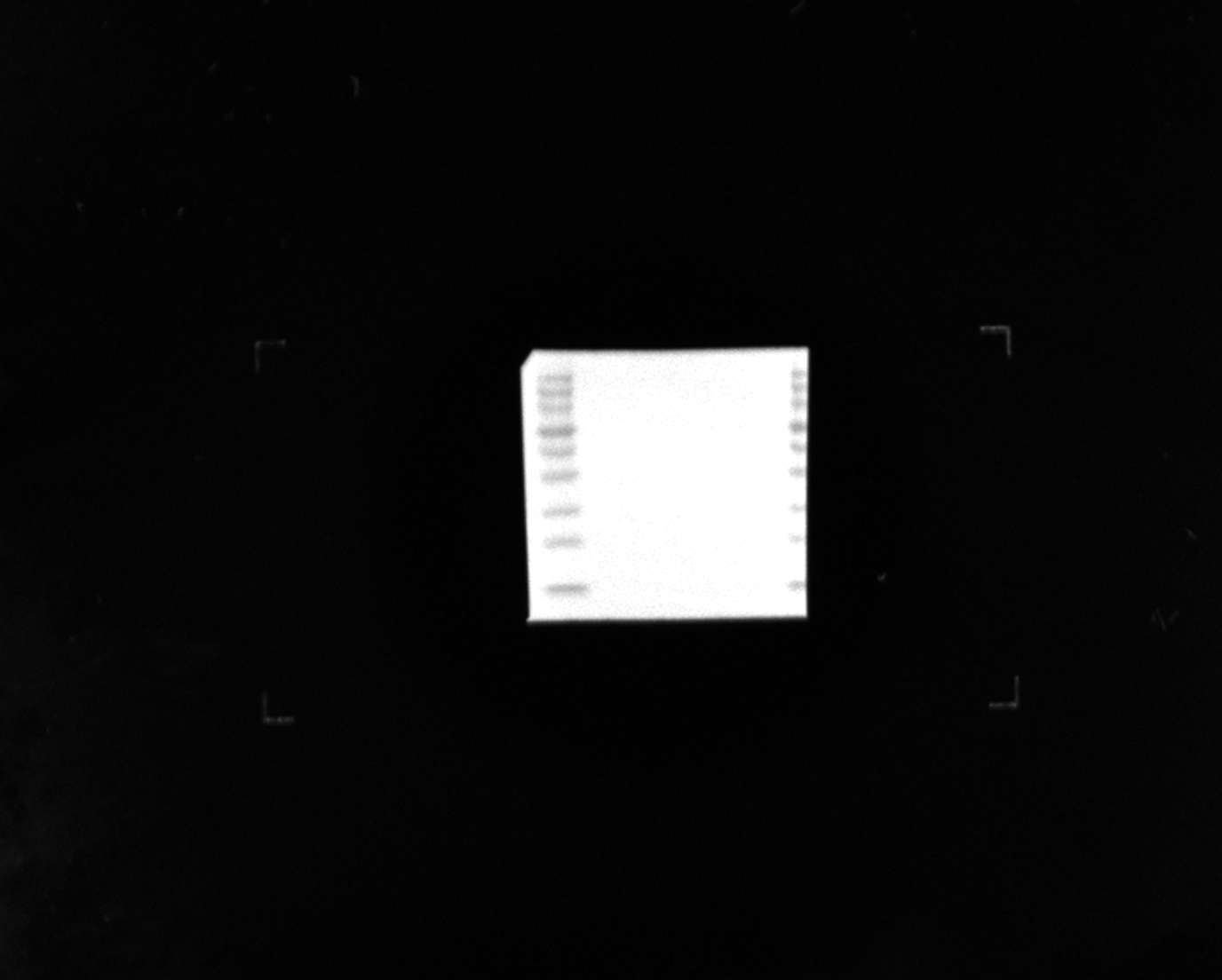

Supplement: Supplementary file 1 [file DataSheet3.zip › 原图1/GSDMD/1-t.Tif]

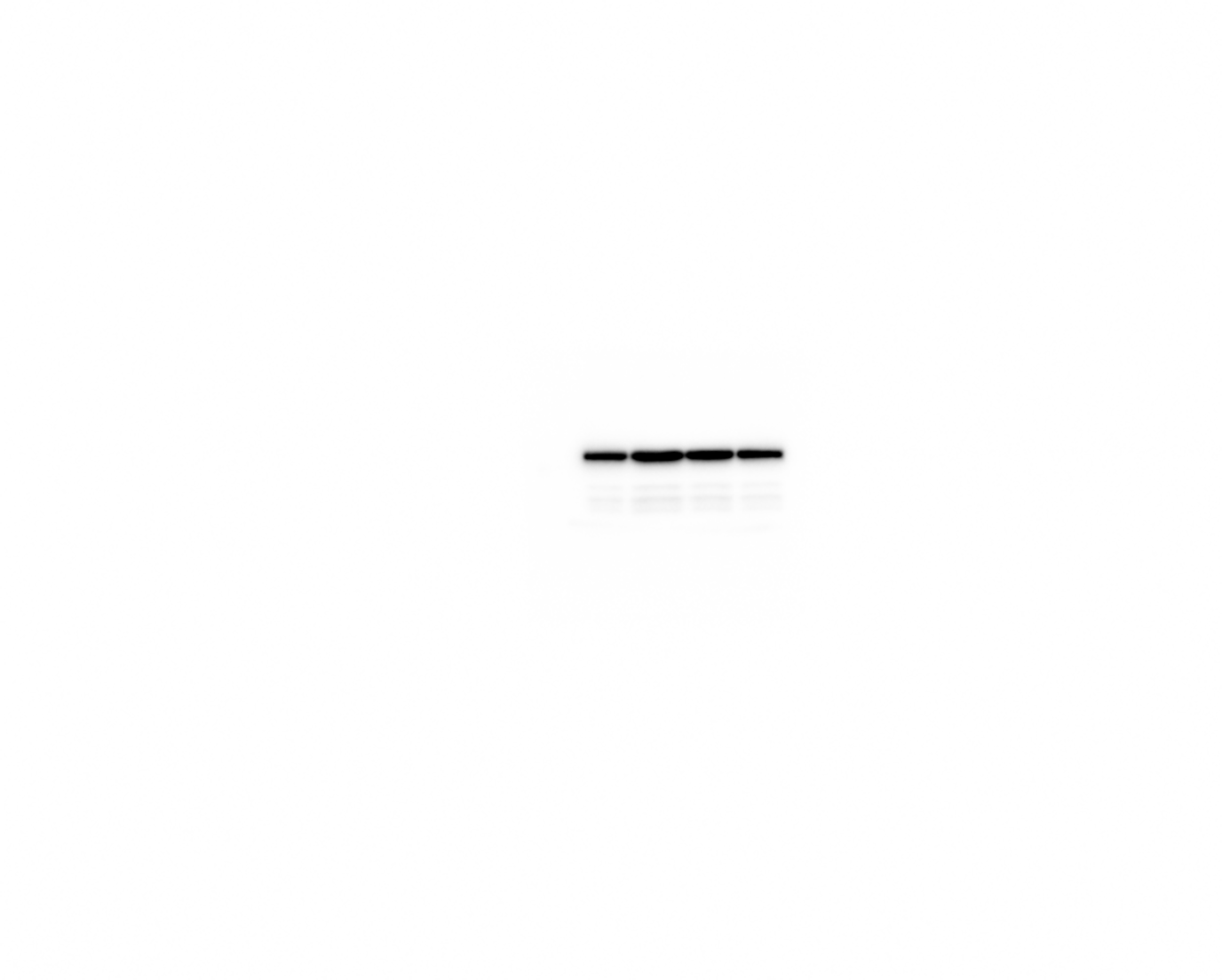

Supplement: Supplementary file 1 [file DataSheet3.zip › 原图1/GSDMD/1.Tif]

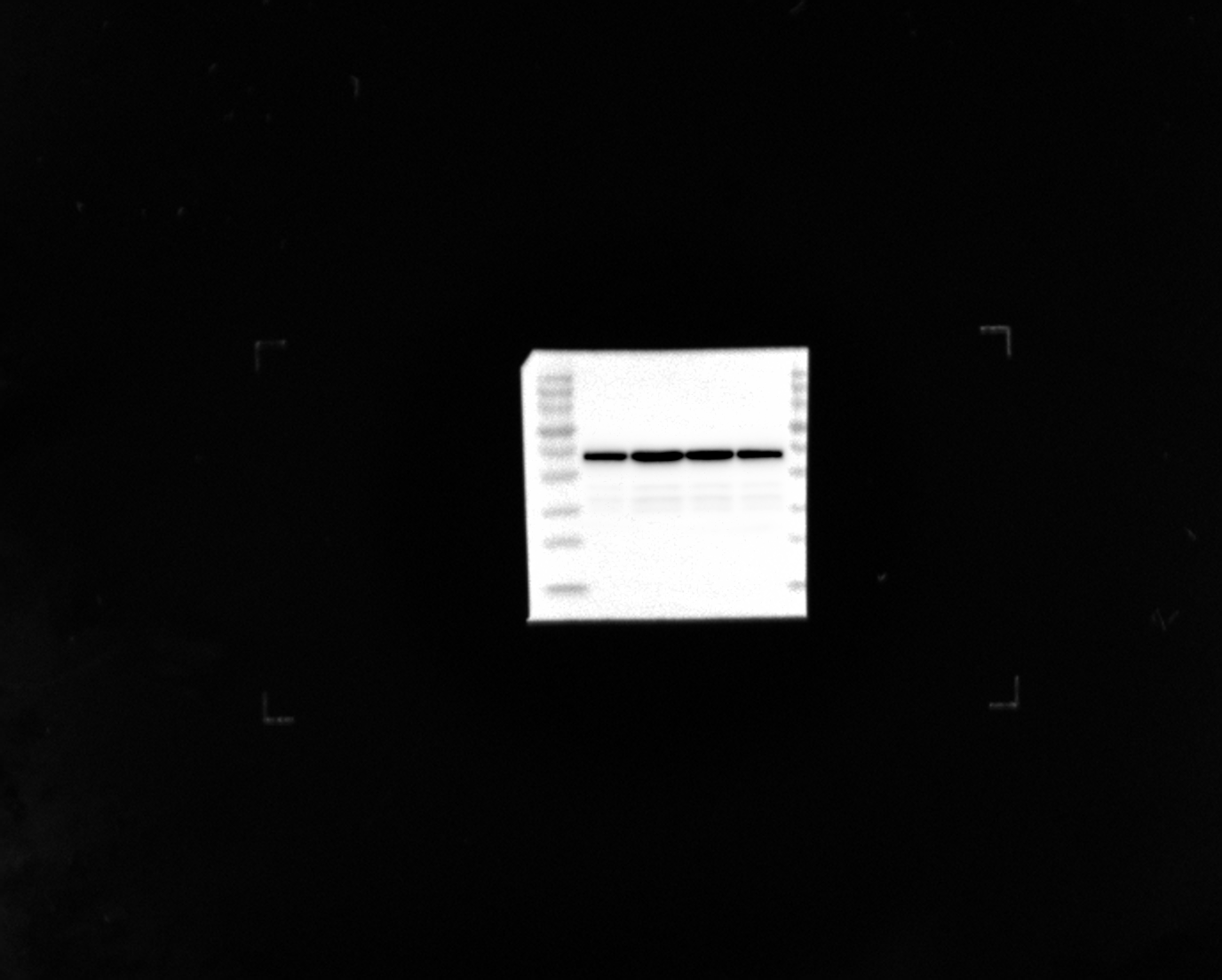

Supplement: Supplementary file 1 [file DataSheet3.zip › 原图1/GSDMD/1副本.tif]

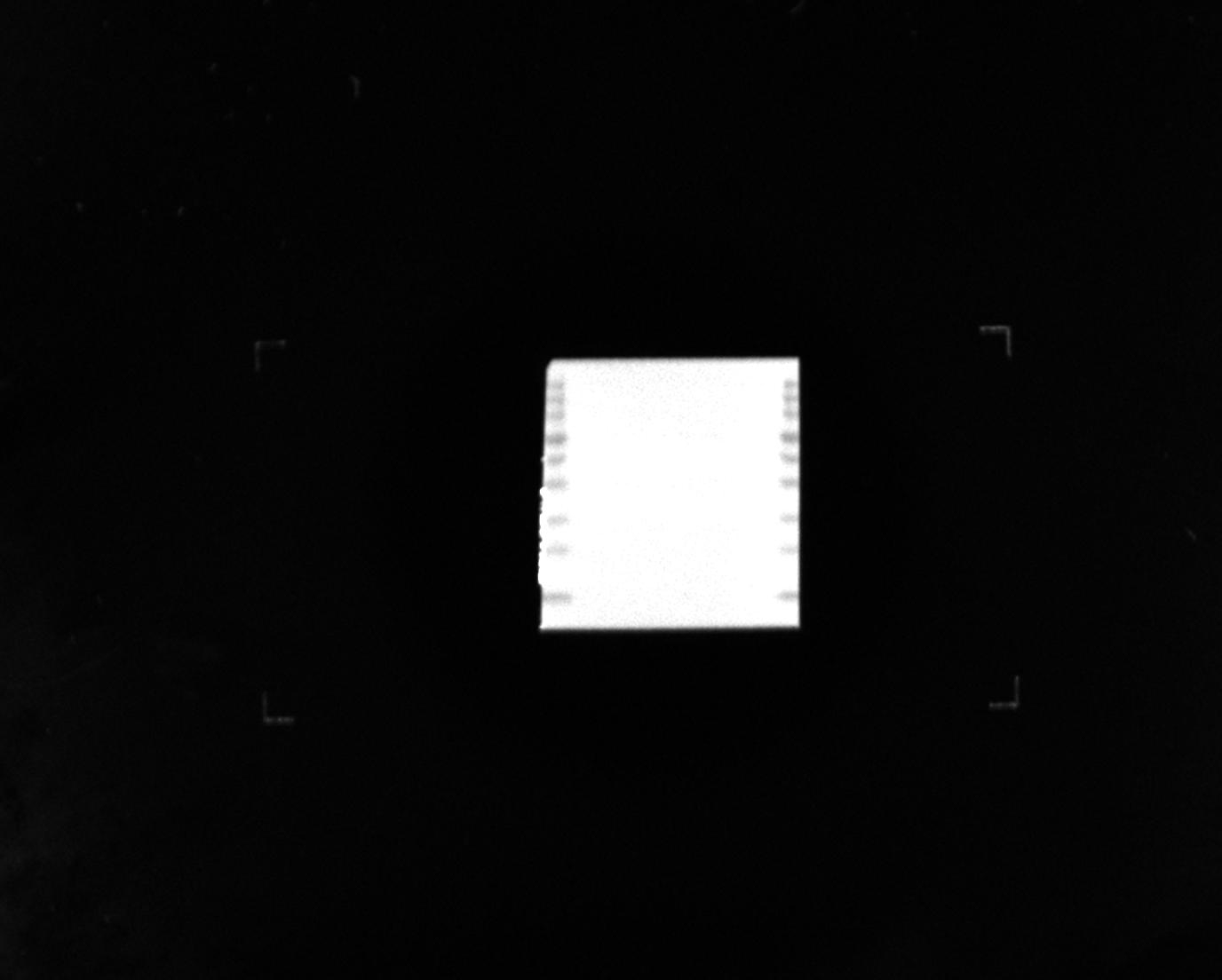

Supplement: Supplementary file 1 [file DataSheet3.zip › 原图1/GSDMD/2-t.Tif]

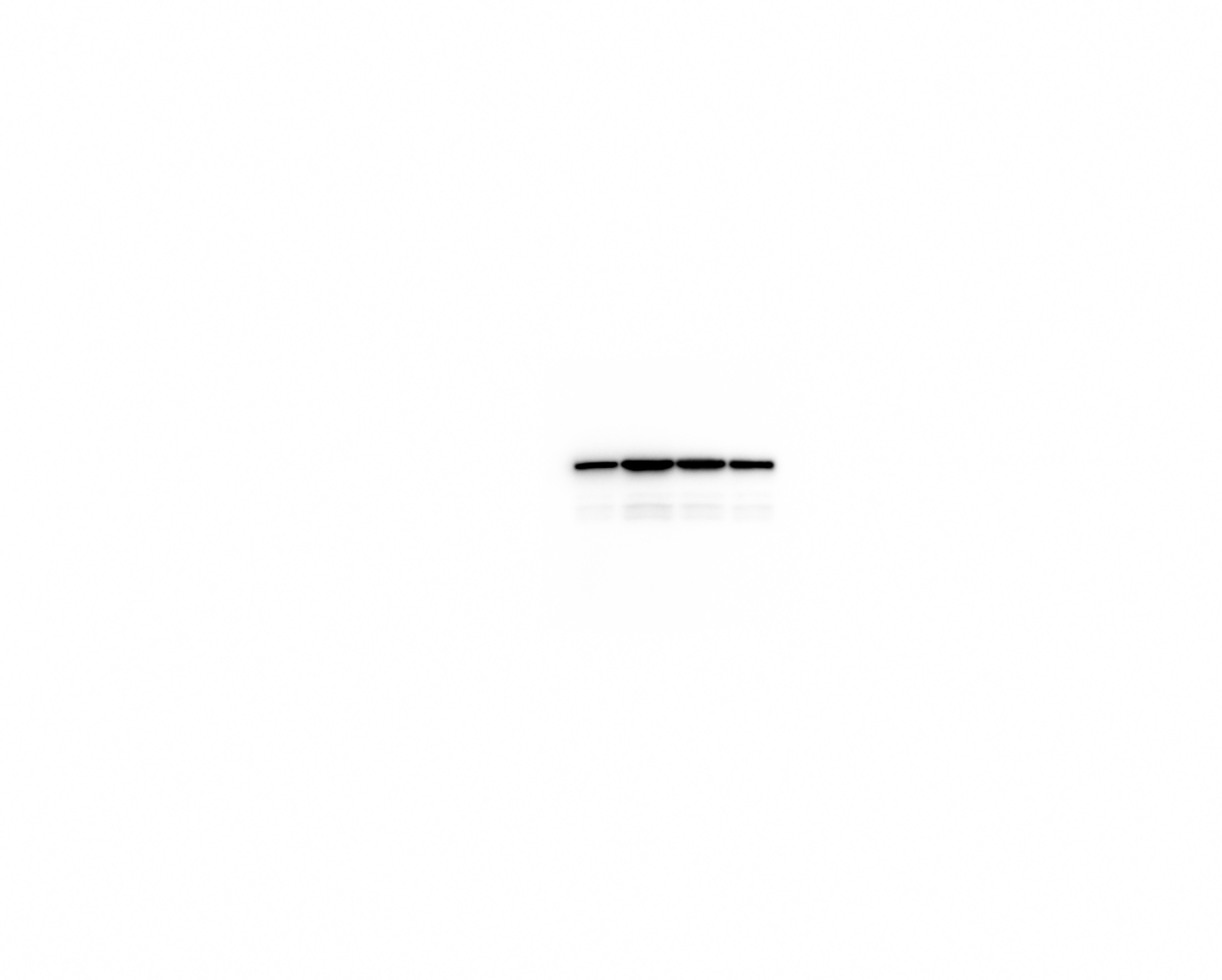

Supplement: Supplementary file 1 [file DataSheet3.zip › 原图1/GSDMD/2.Tif]

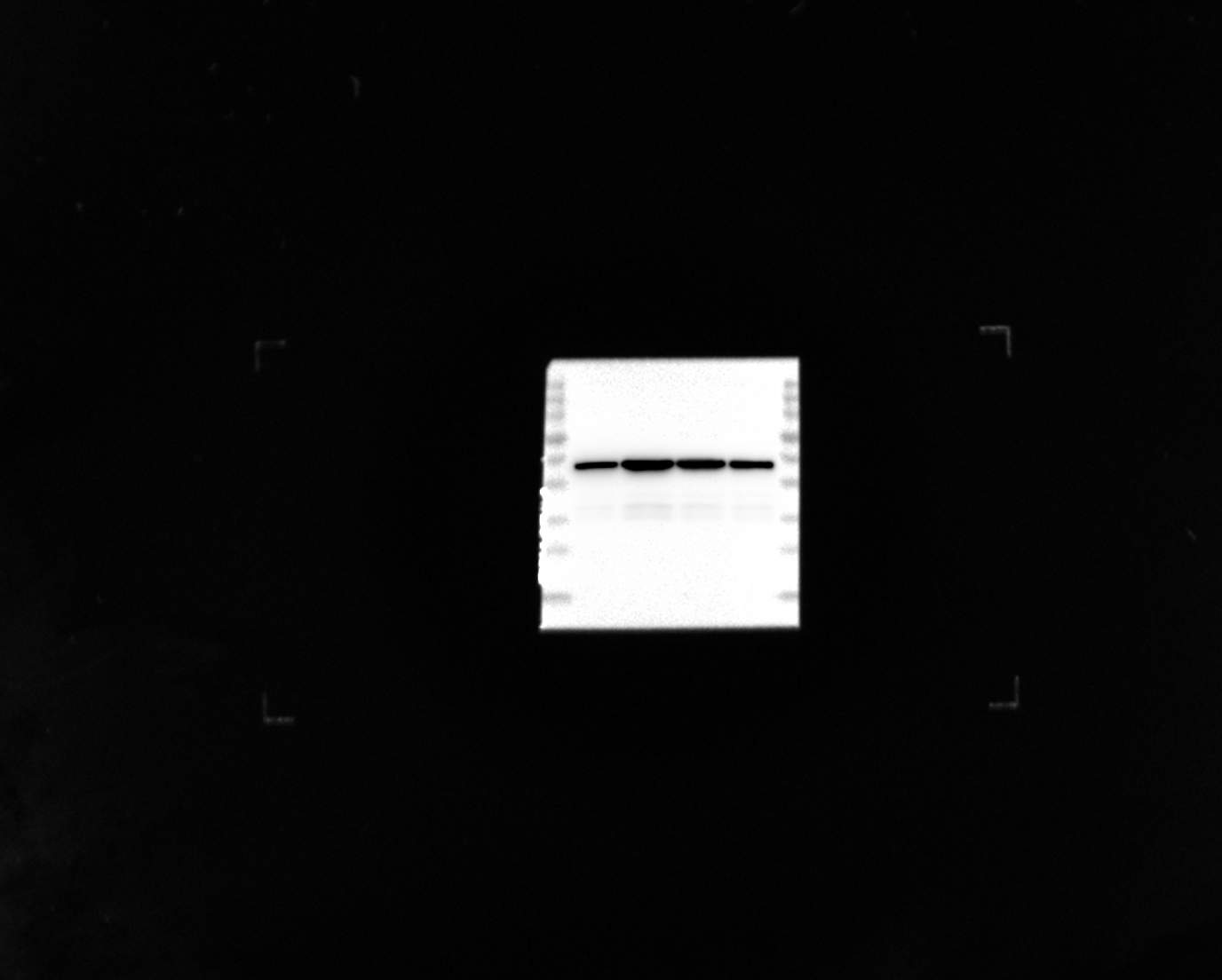

Supplement: Supplementary file 1 [file DataSheet3.zip › 原图1/GSDMD/2副本.tif]

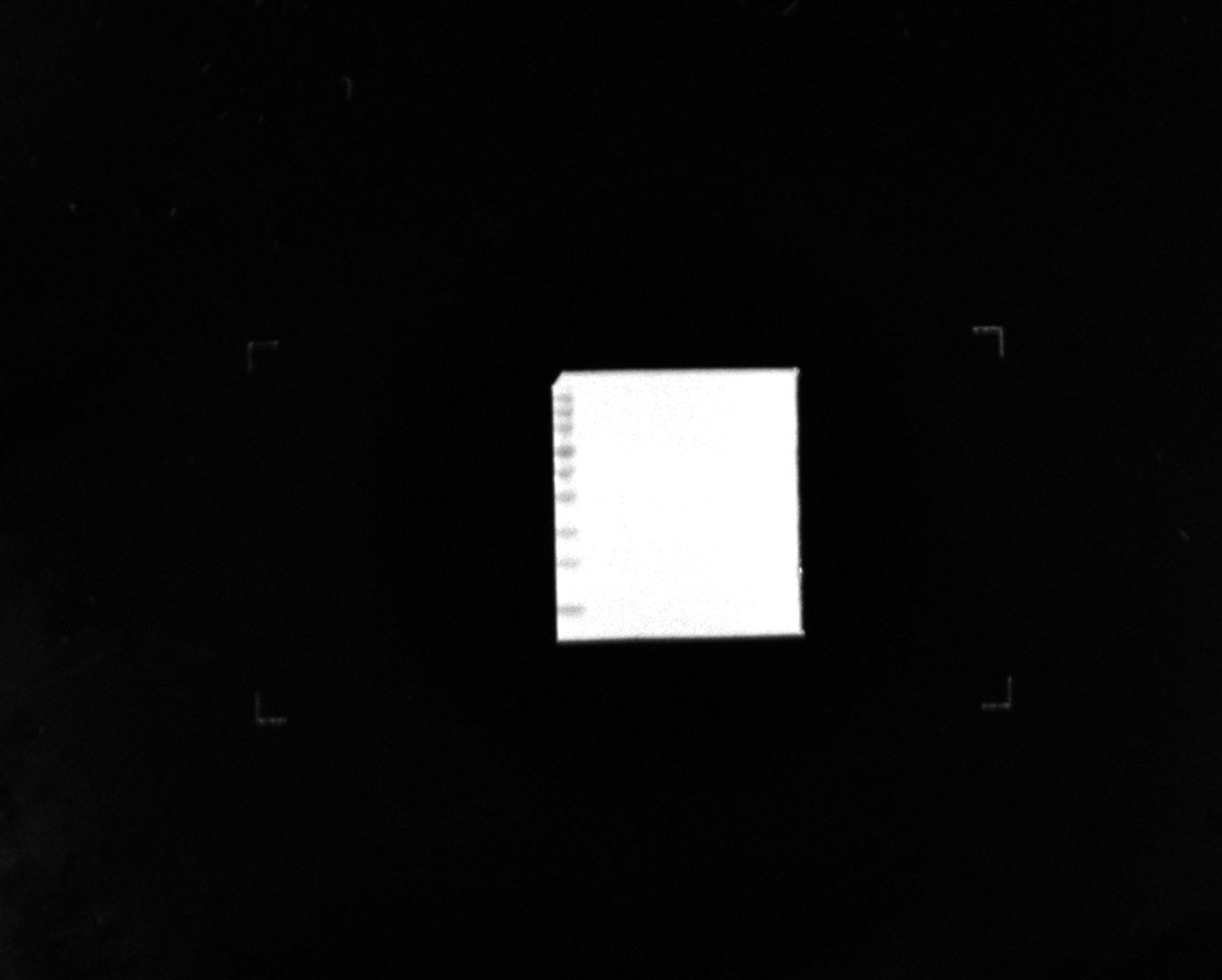

Supplement: Supplementary file 1 [file DataSheet3.zip › 原图1/GSDMD/3-t.Tif]

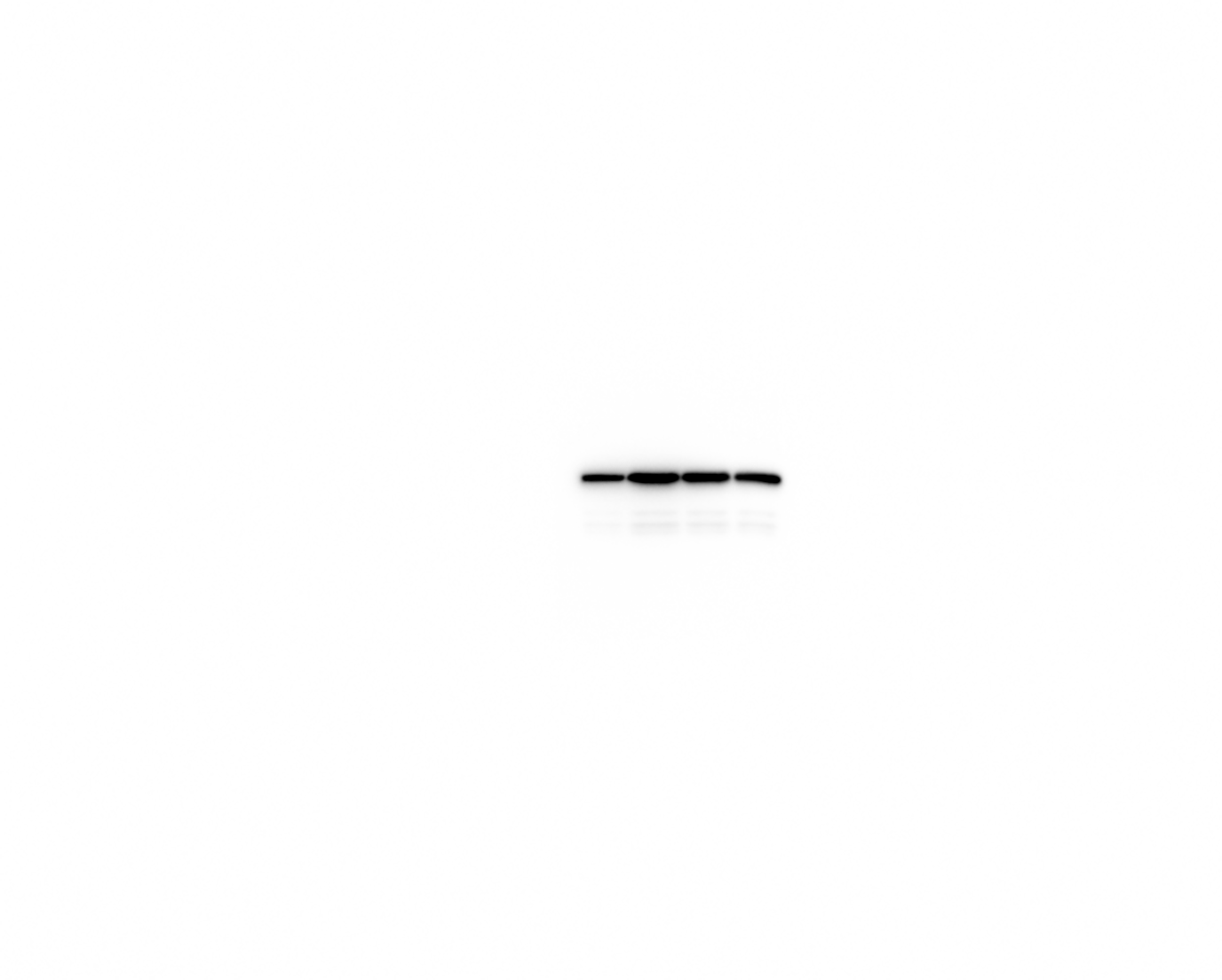

Supplement: Supplementary file 1 [file DataSheet3.zip › 原图1/GSDMD/3.Tif]

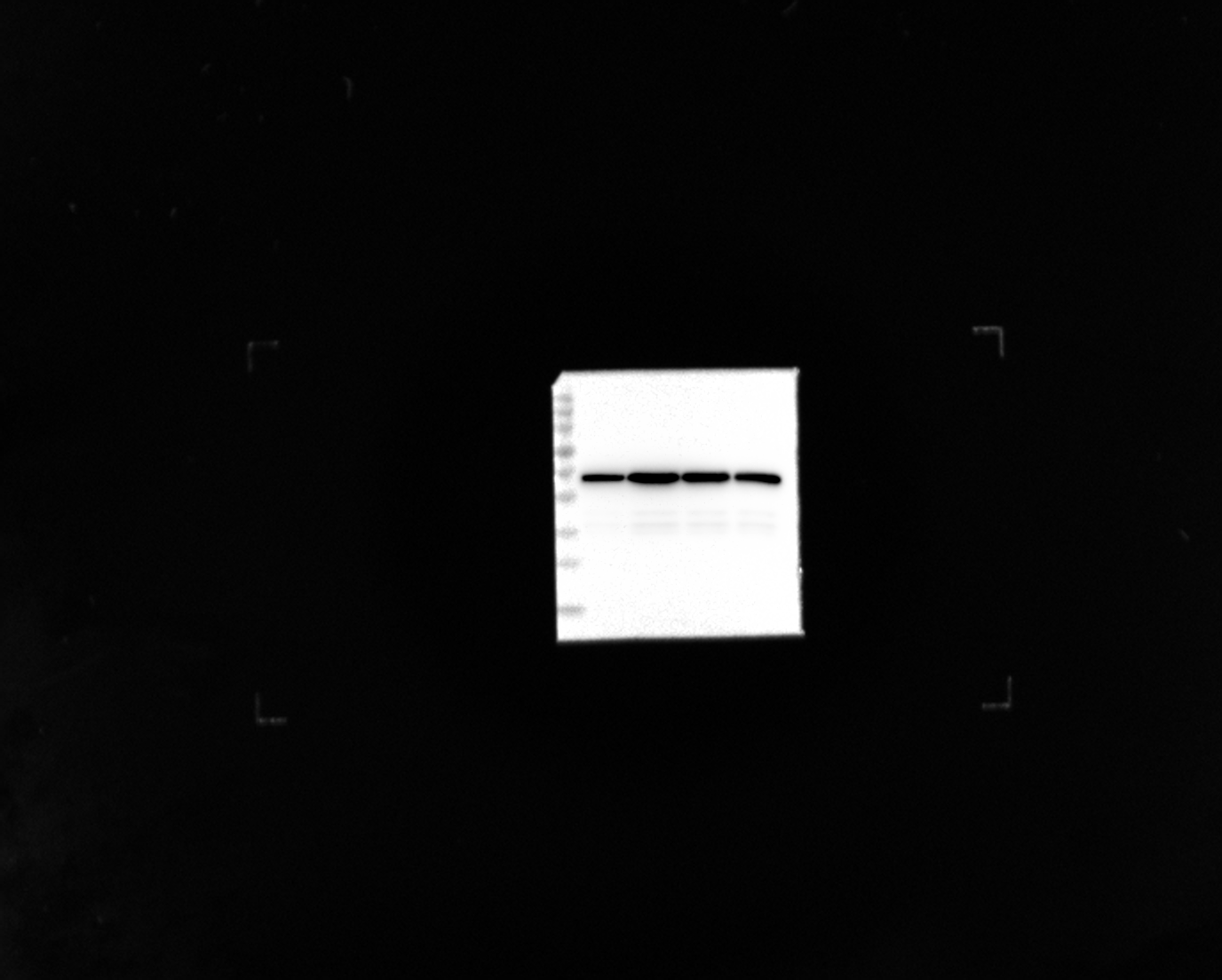

Supplement: Supplementary file 1 [file DataSheet3.zip › 原图1/GSDMD/3副本.tif]

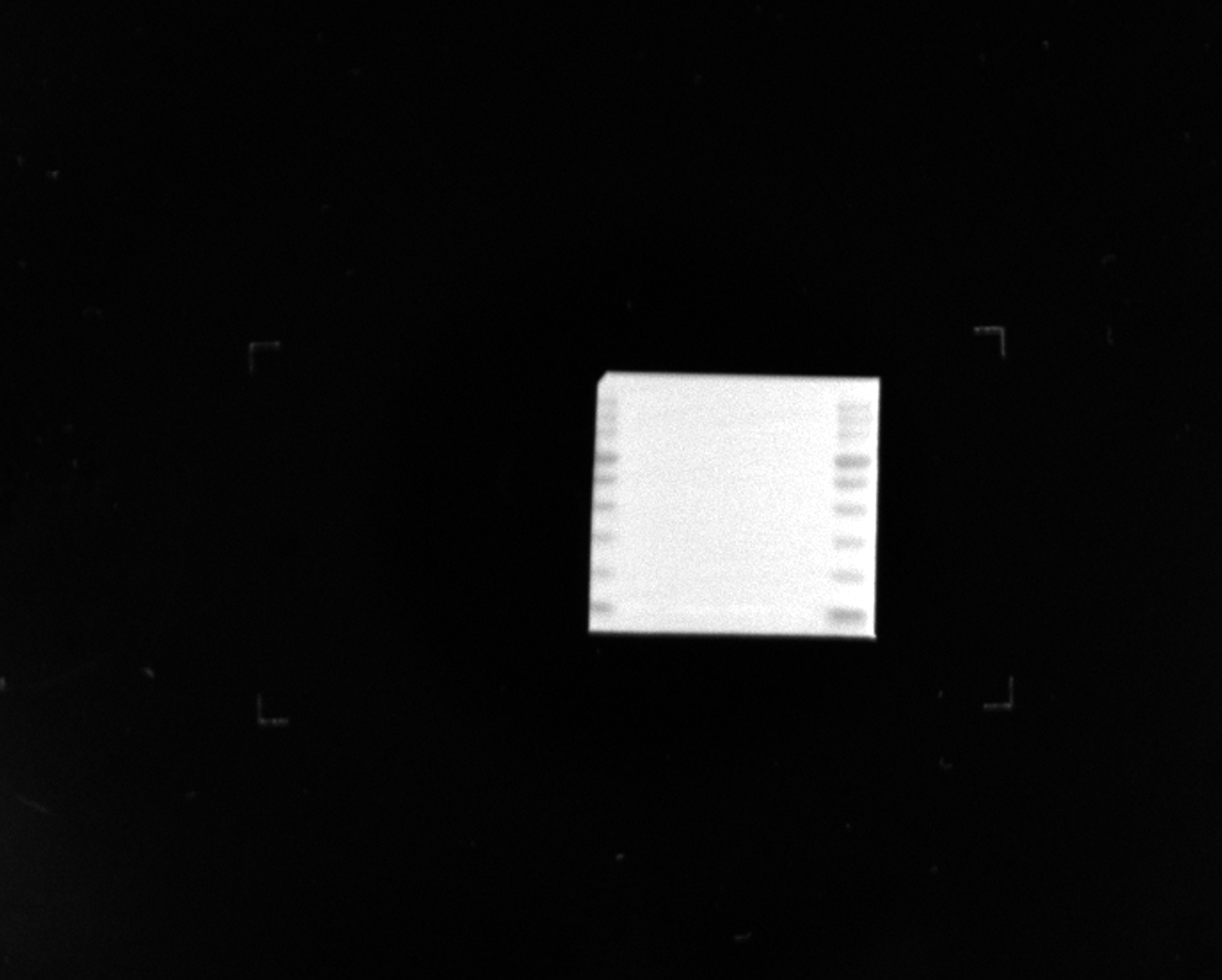

Supplement: Supplementary file 1 [file DataSheet3.zip › 原图1/IL1β/1-t.Tif]

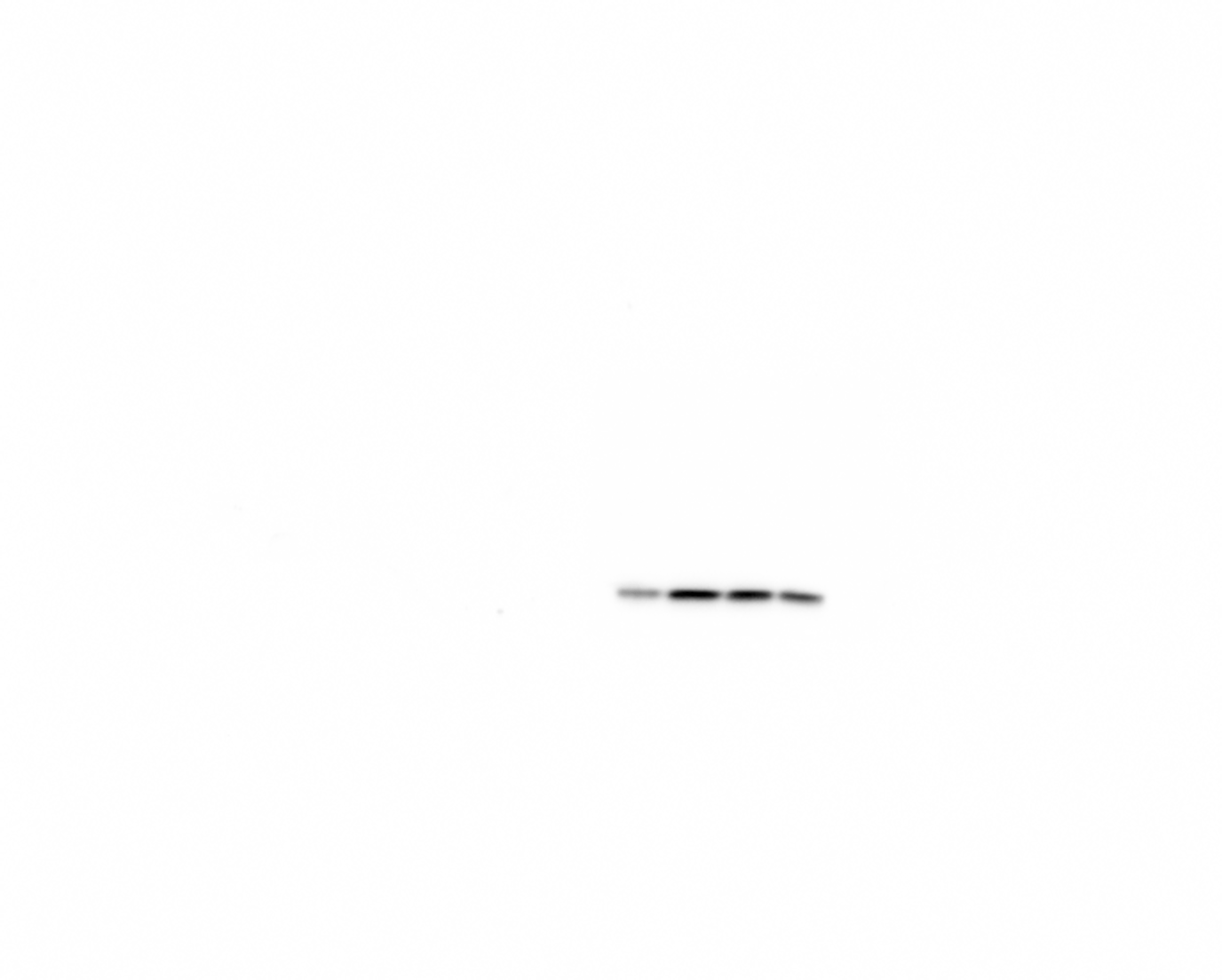

Supplement: Supplementary file 1 [file DataSheet3.zip › 原图1/IL1β/1.Tif]

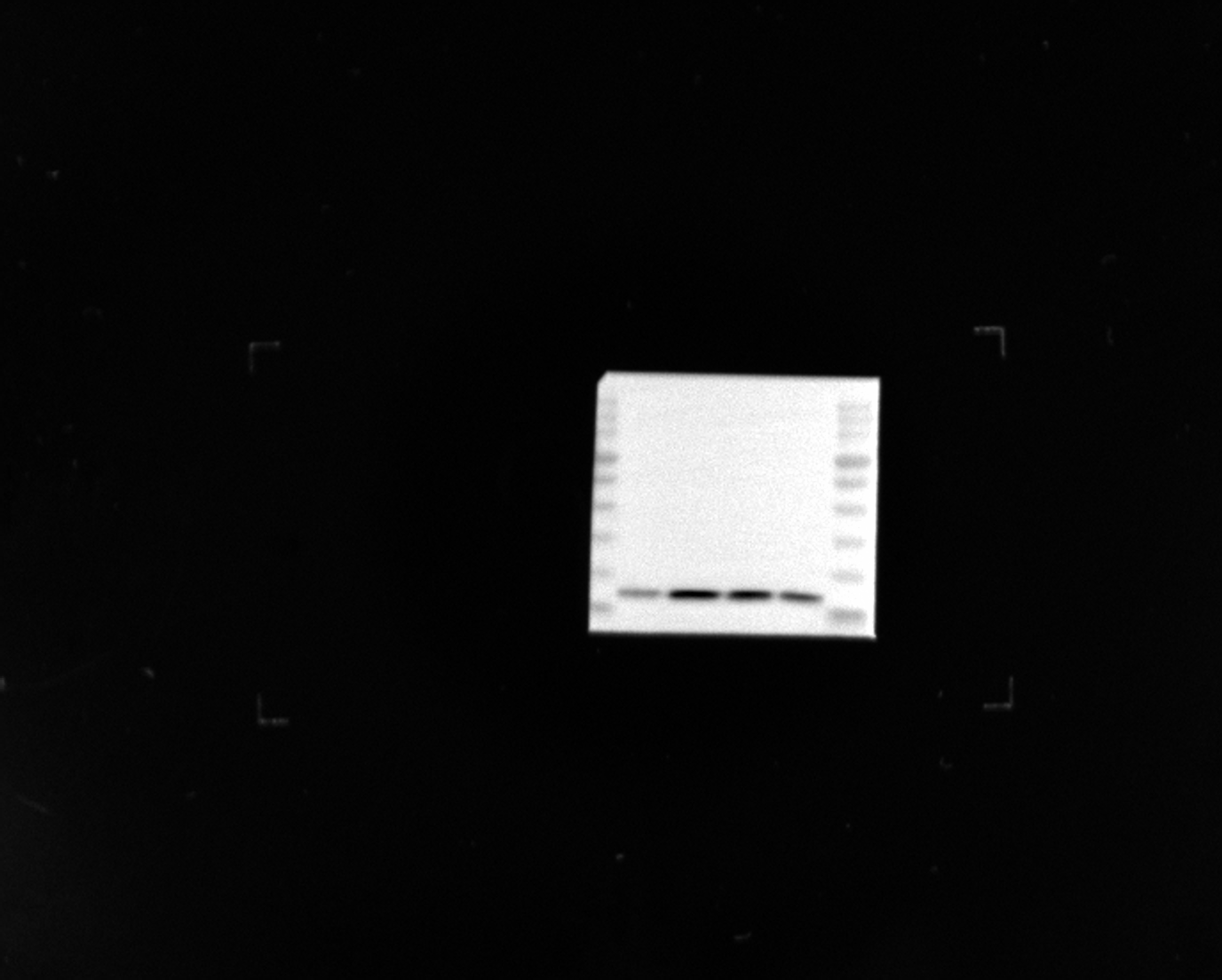

Supplement: Supplementary file 1 [file DataSheet3.zip › 原图1/IL1β/1副本.tif]

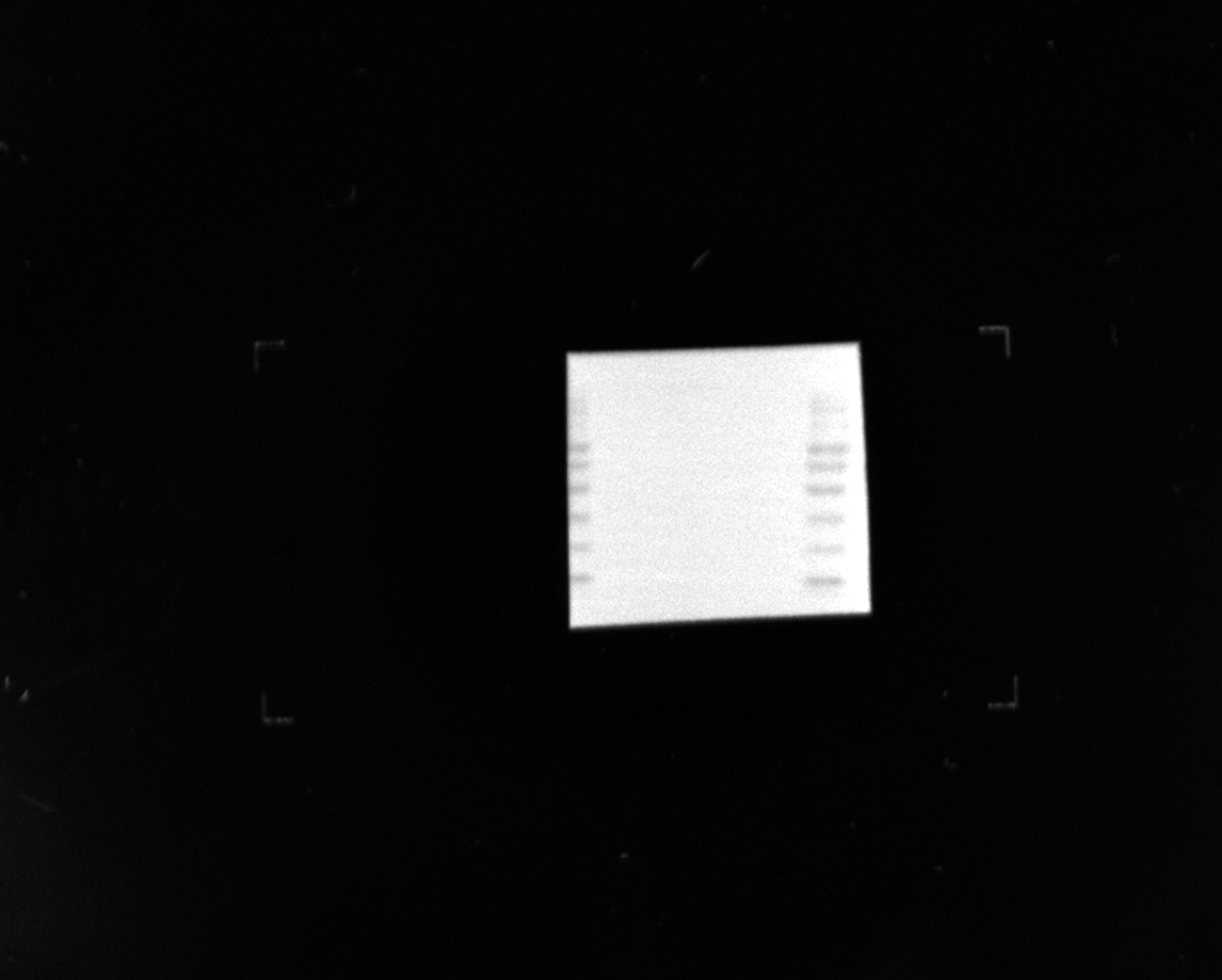

Supplement: Supplementary file 1 [file DataSheet3.zip › 原图1/IL1β/2-t.Tif]

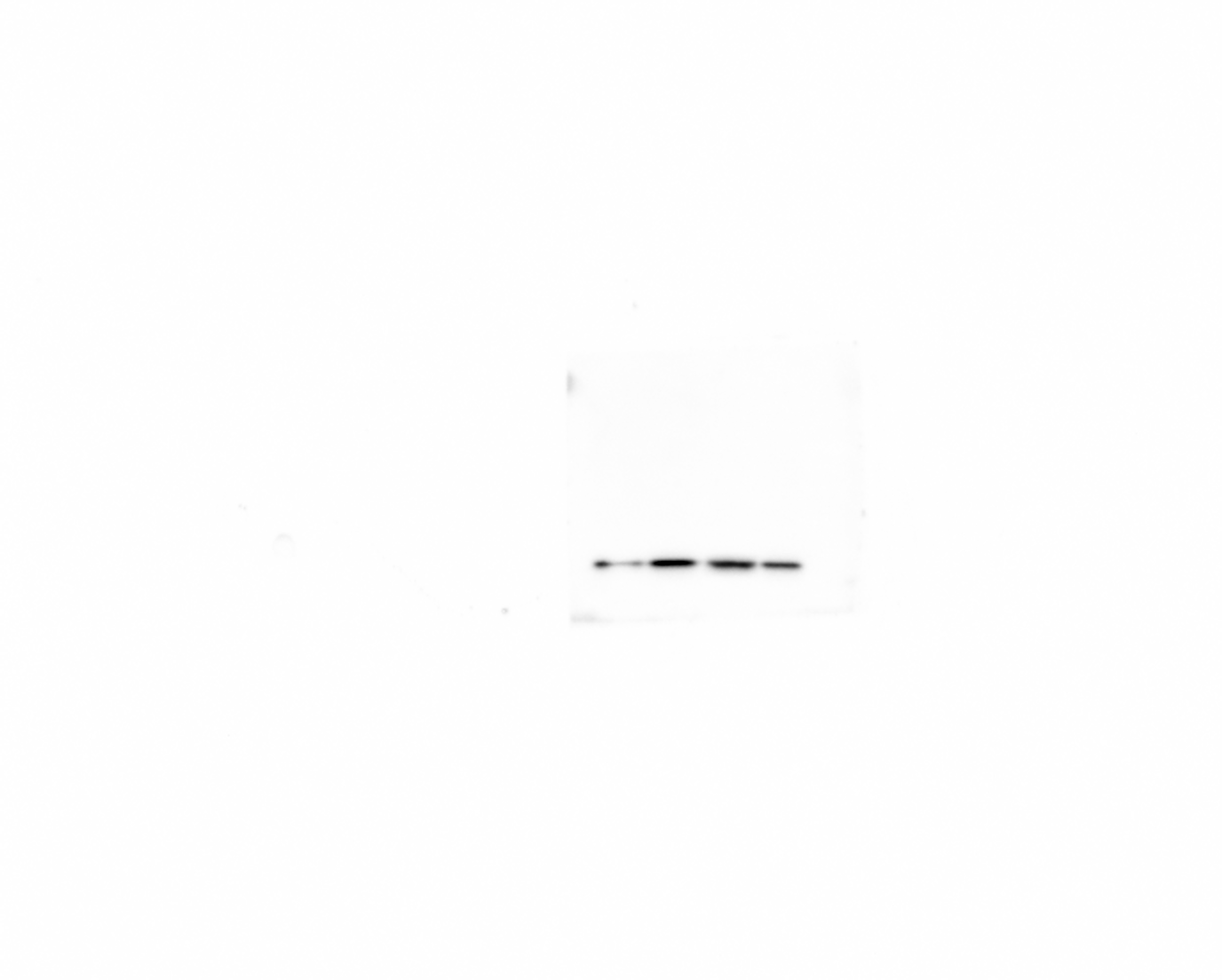

Supplement: Supplementary file 1 [file DataSheet3.zip › 原图1/IL1β/2.Tif]

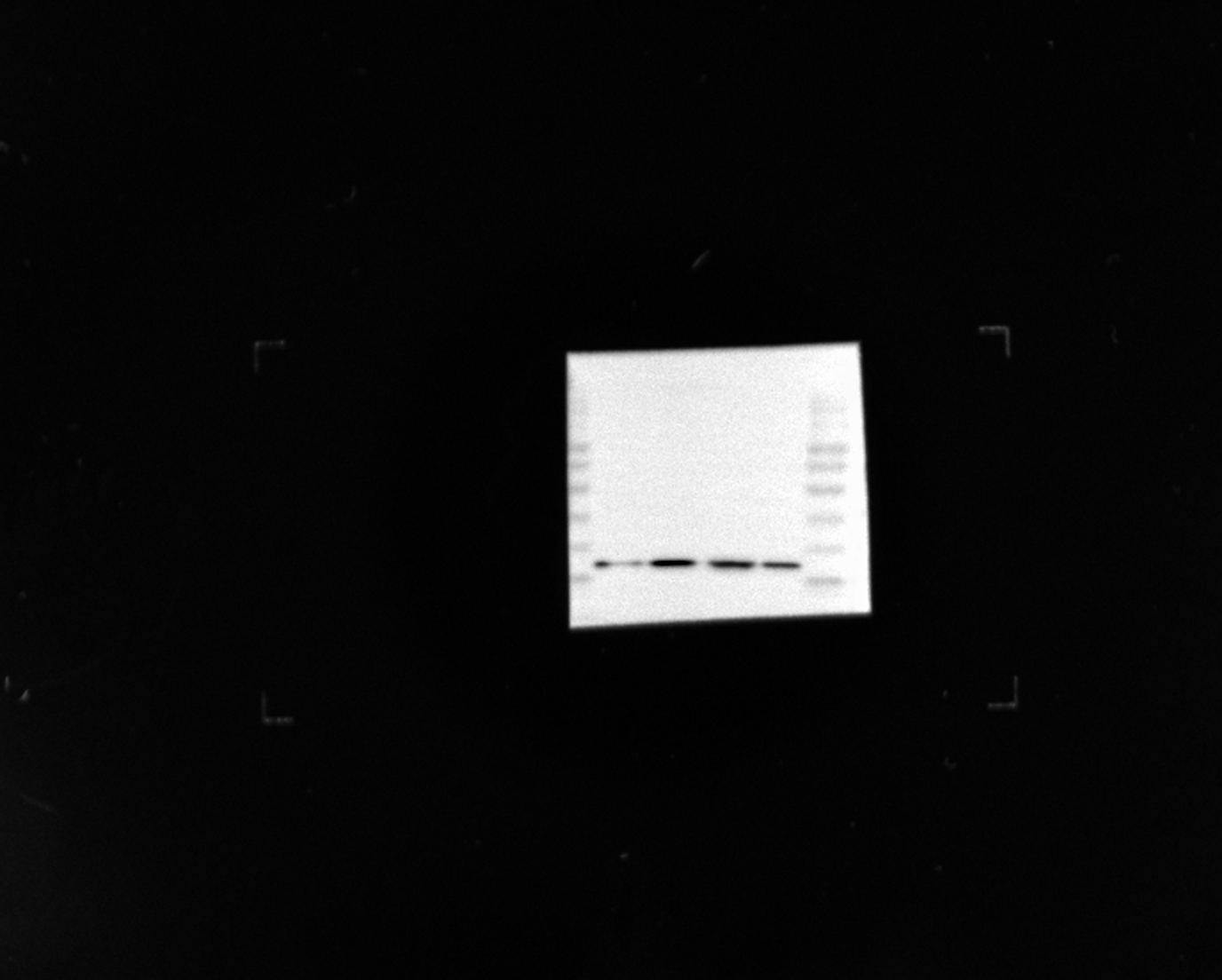

Supplement: Supplementary file 1 [file DataSheet3.zip › 原图1/IL1β/2副本.tif]

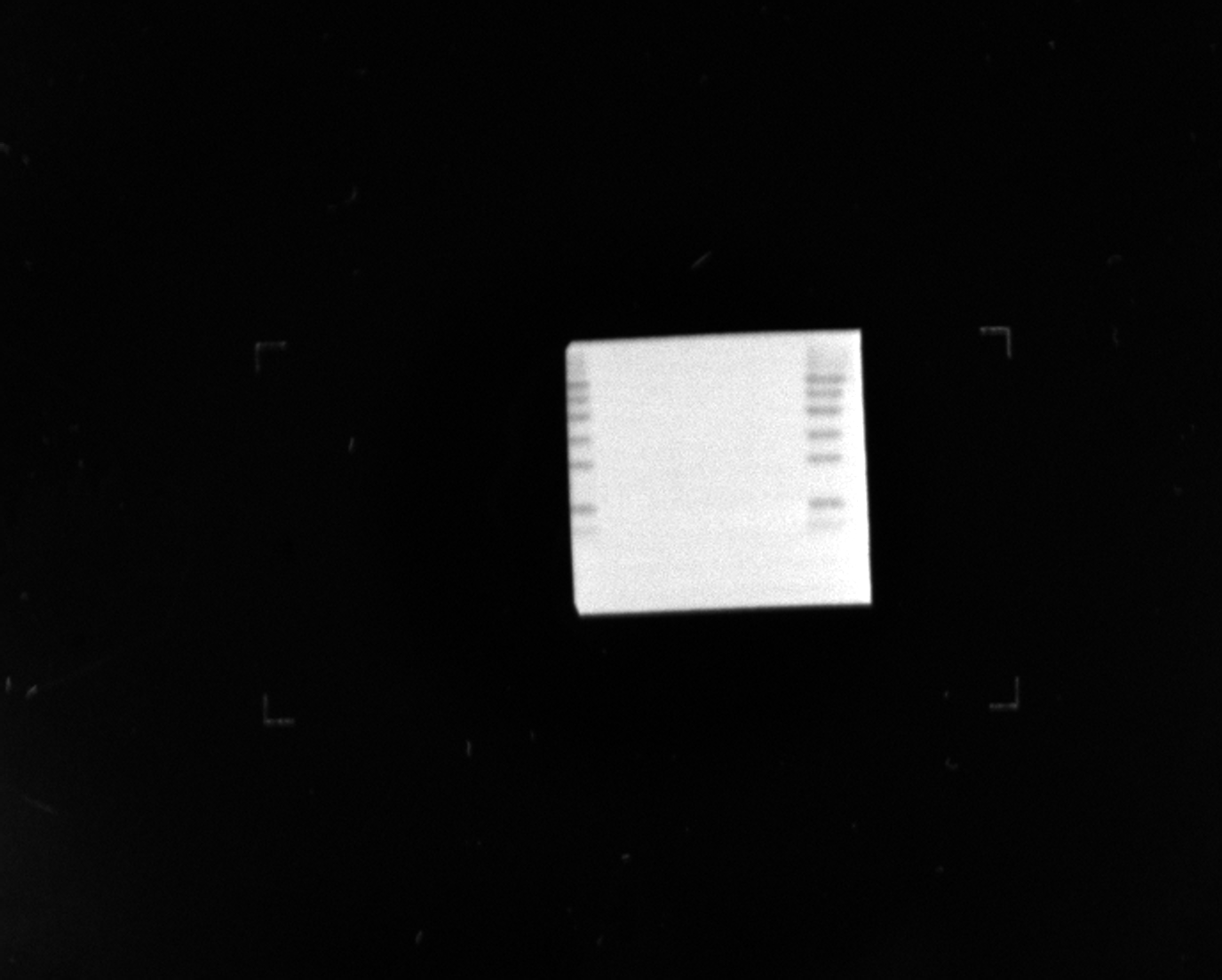

Supplement: Supplementary file 1 [file DataSheet3.zip › 原图1/IL1β/3-t.Tif]

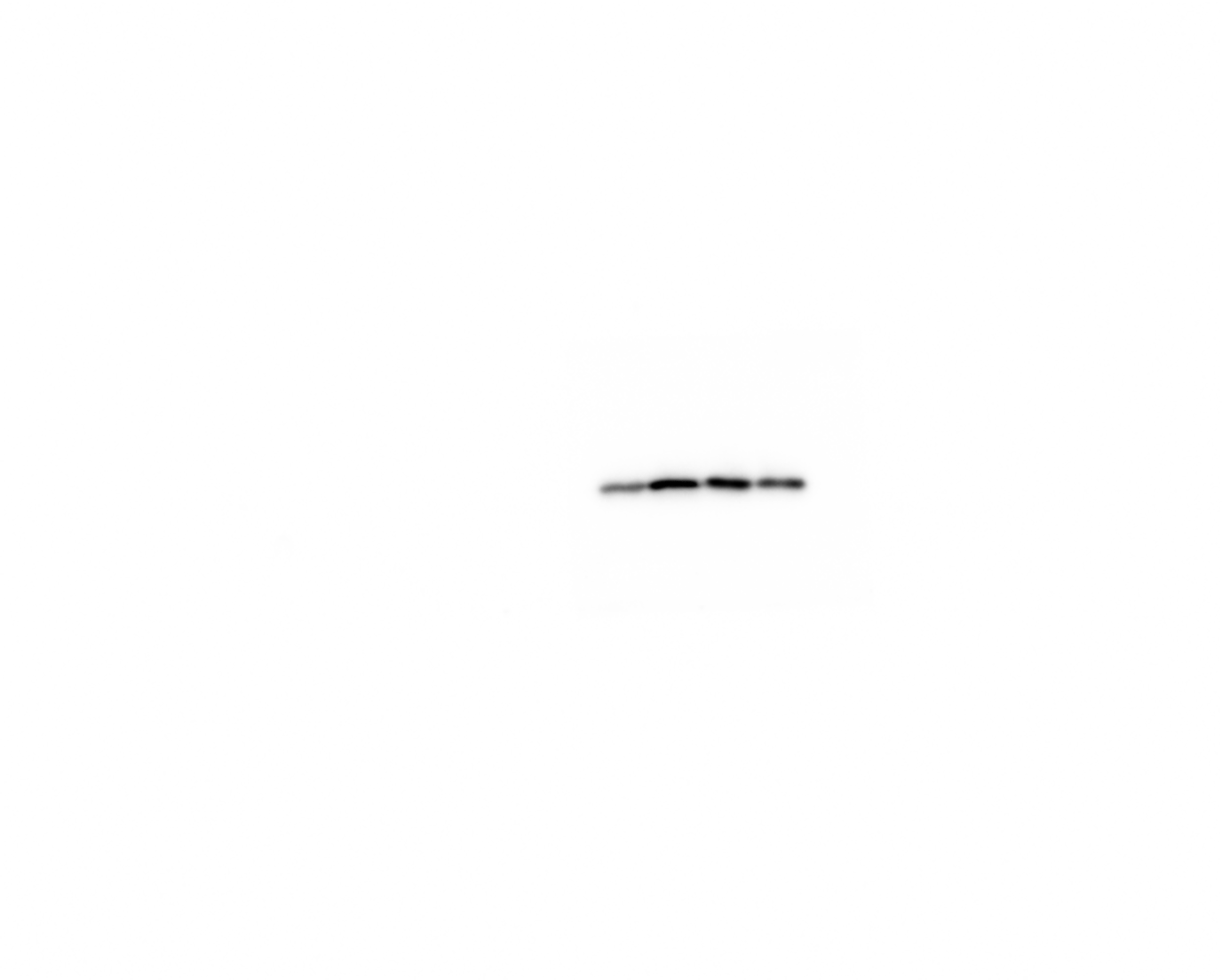

Supplement: Supplementary file 1 [file DataSheet3.zip › 原图1/IL1β/3.Tif]

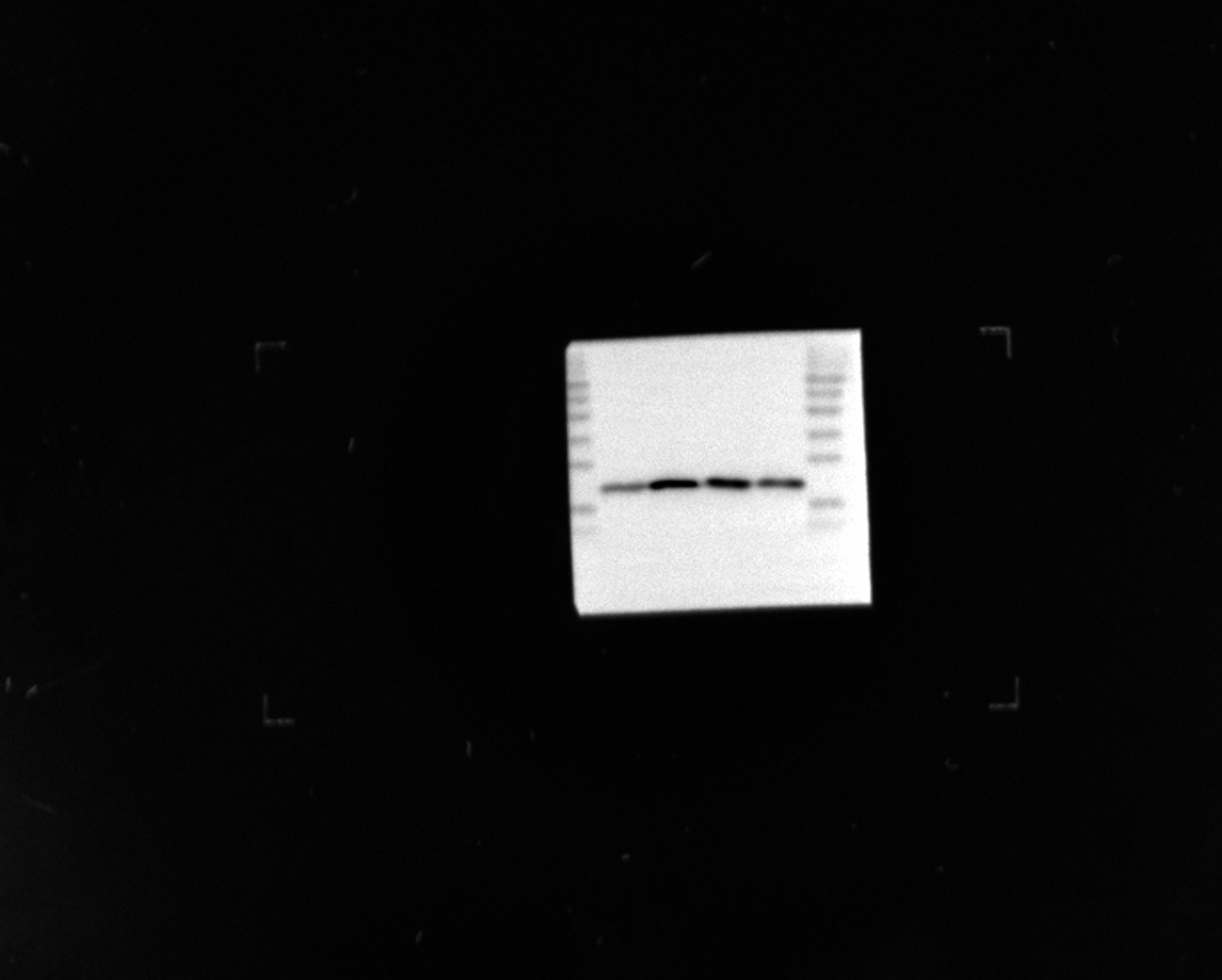

Supplement: Supplementary file 1 [file DataSheet3.zip › 原图1/IL1β/3副本.tif]

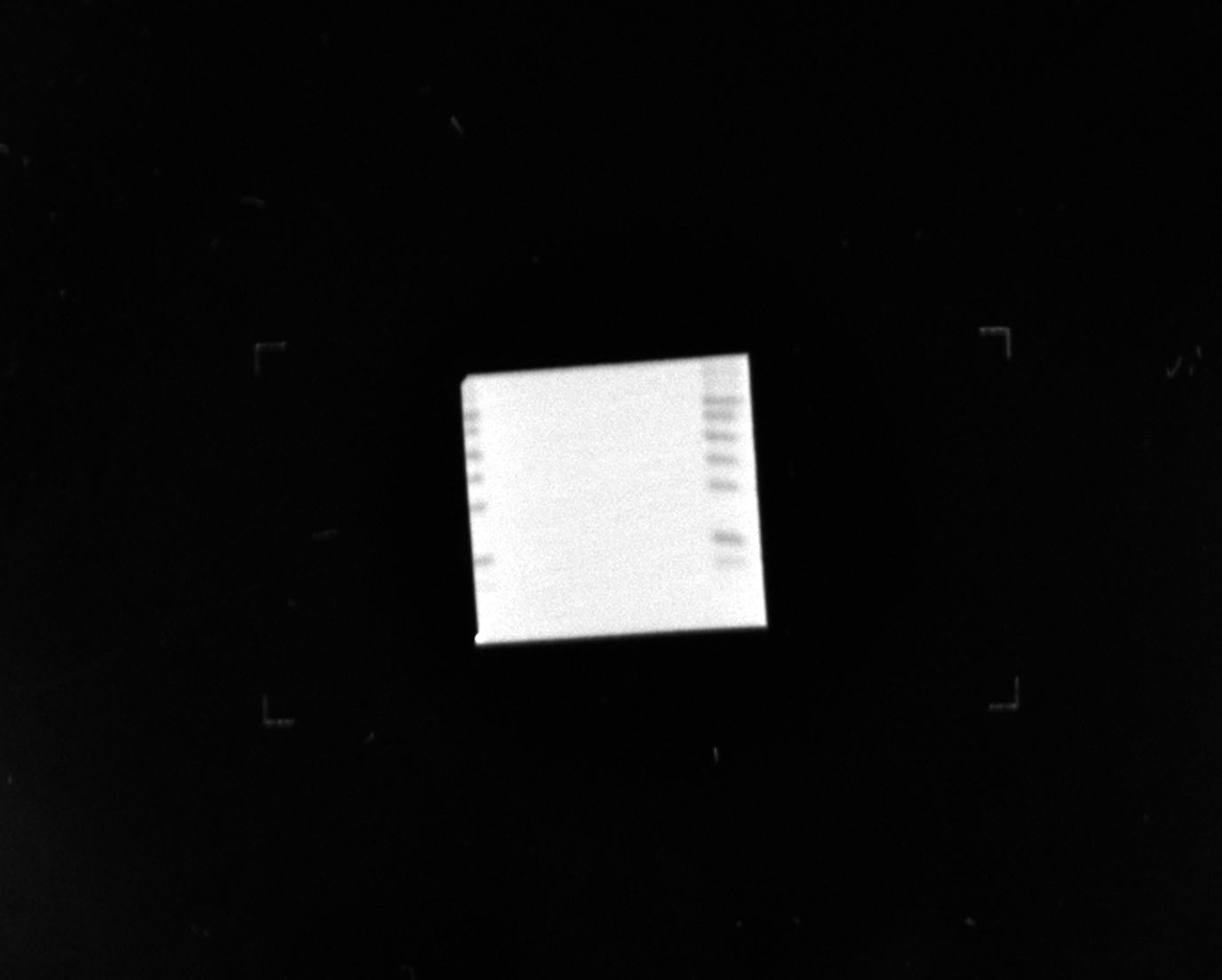

Supplement: Supplementary file 1 [file DataSheet3.zip › 原图1/KCNK3/1-t.Tif]

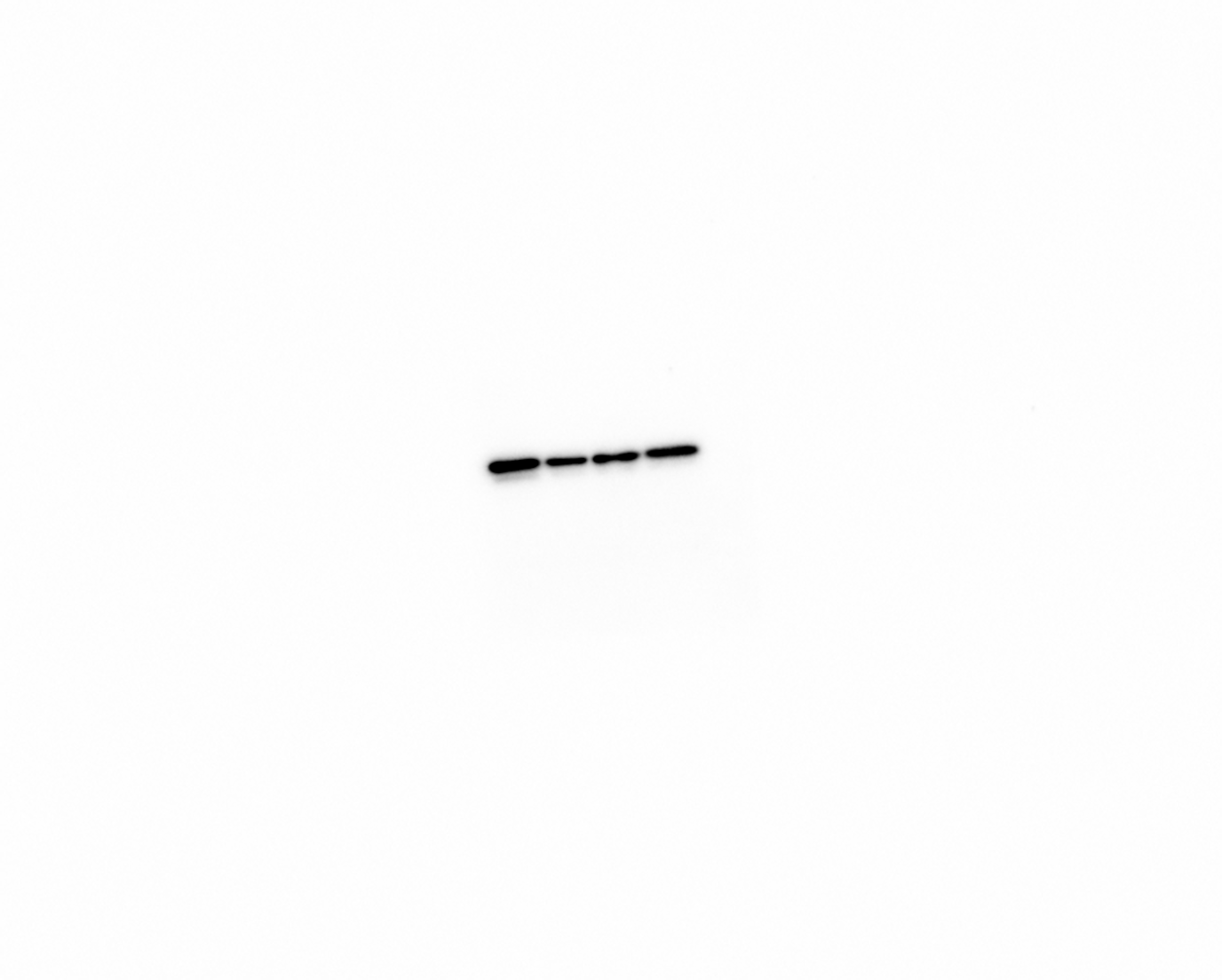

Supplement: Supplementary file 1 [file DataSheet3.zip › 原图1/KCNK3/1.Tif]

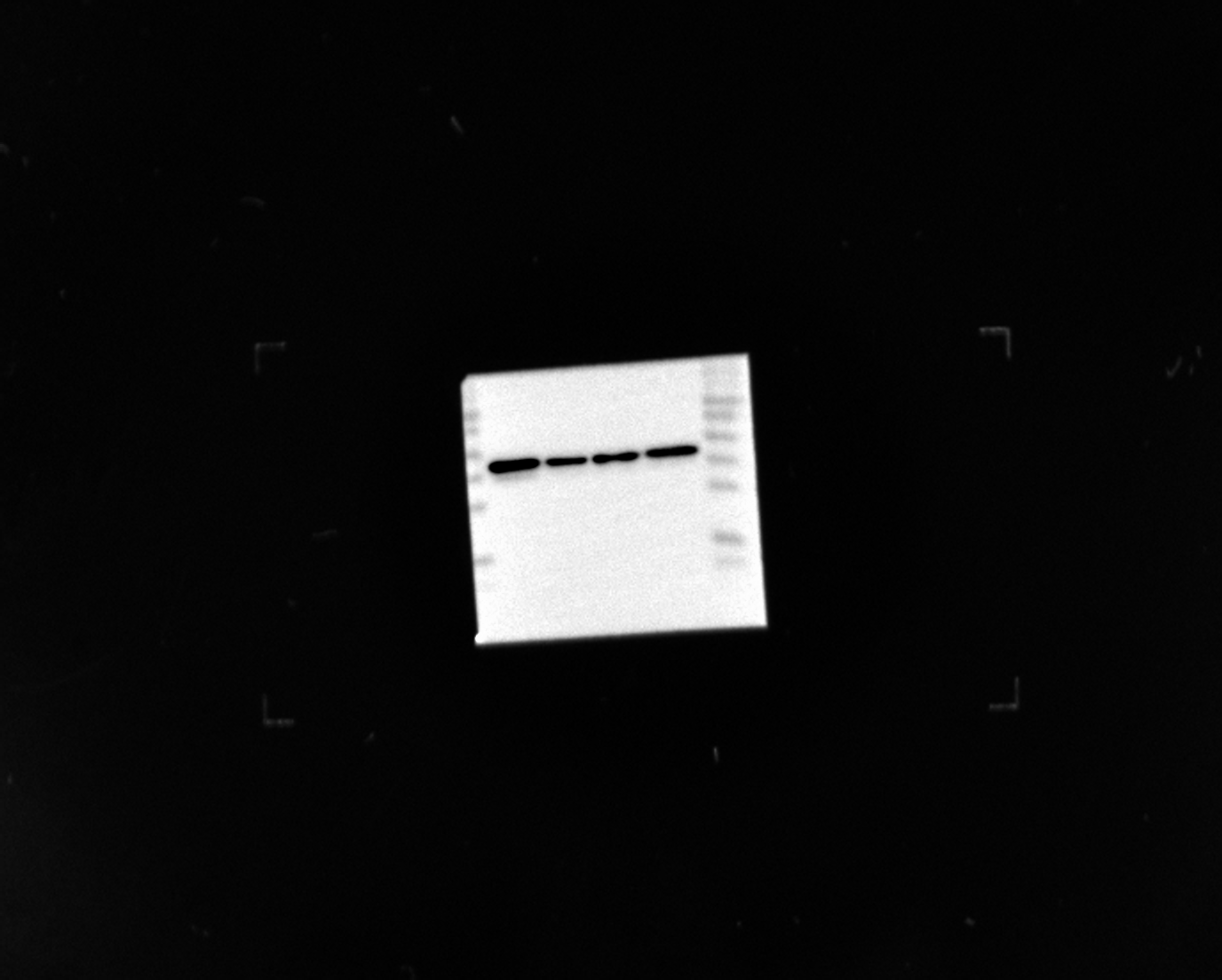

Supplement: Supplementary file 1 [file DataSheet3.zip › 原图1/KCNK3/1副本.tif]

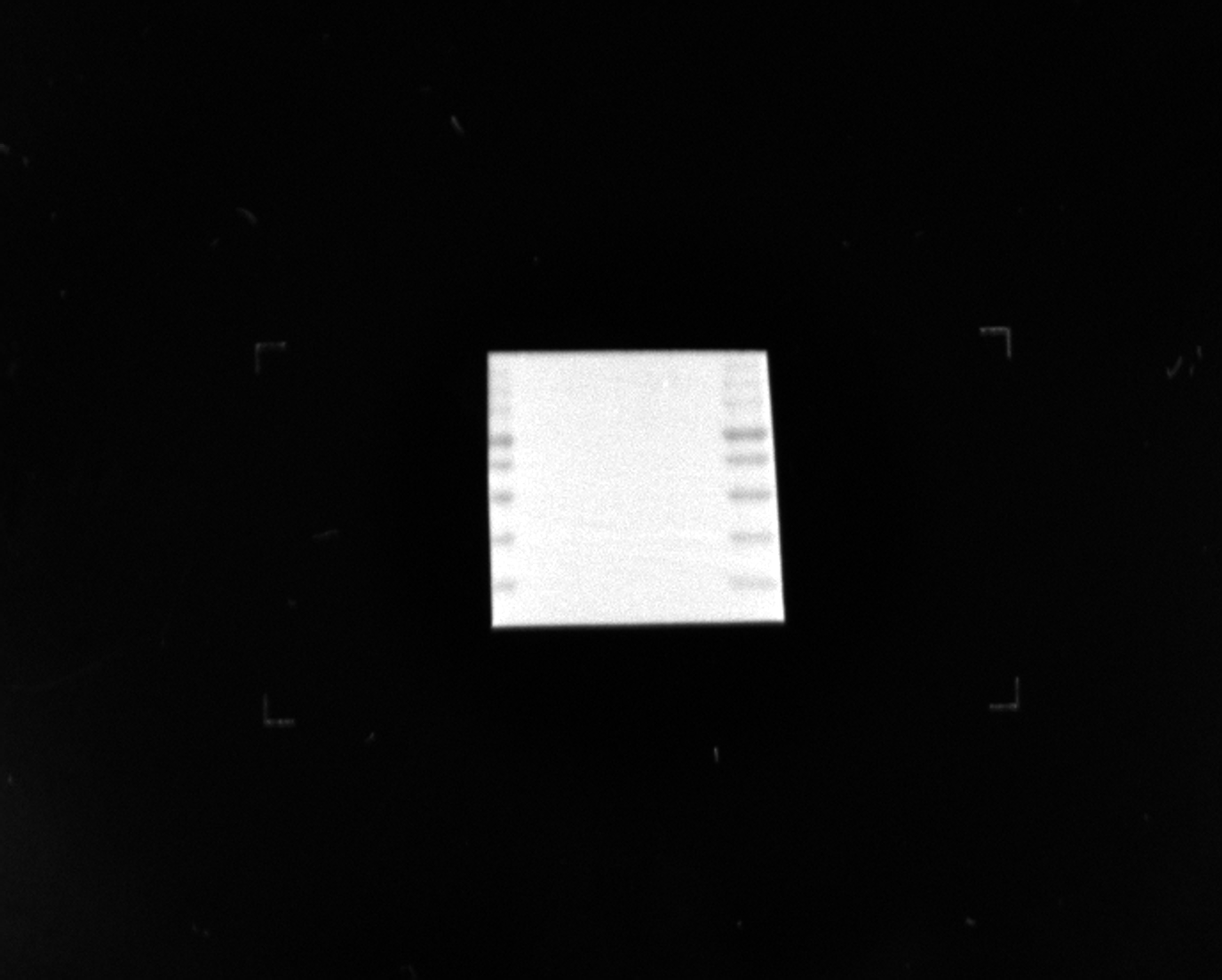

Supplement: Supplementary file 1 [file DataSheet3.zip › 原图1/KCNK3/2-t.Tif]

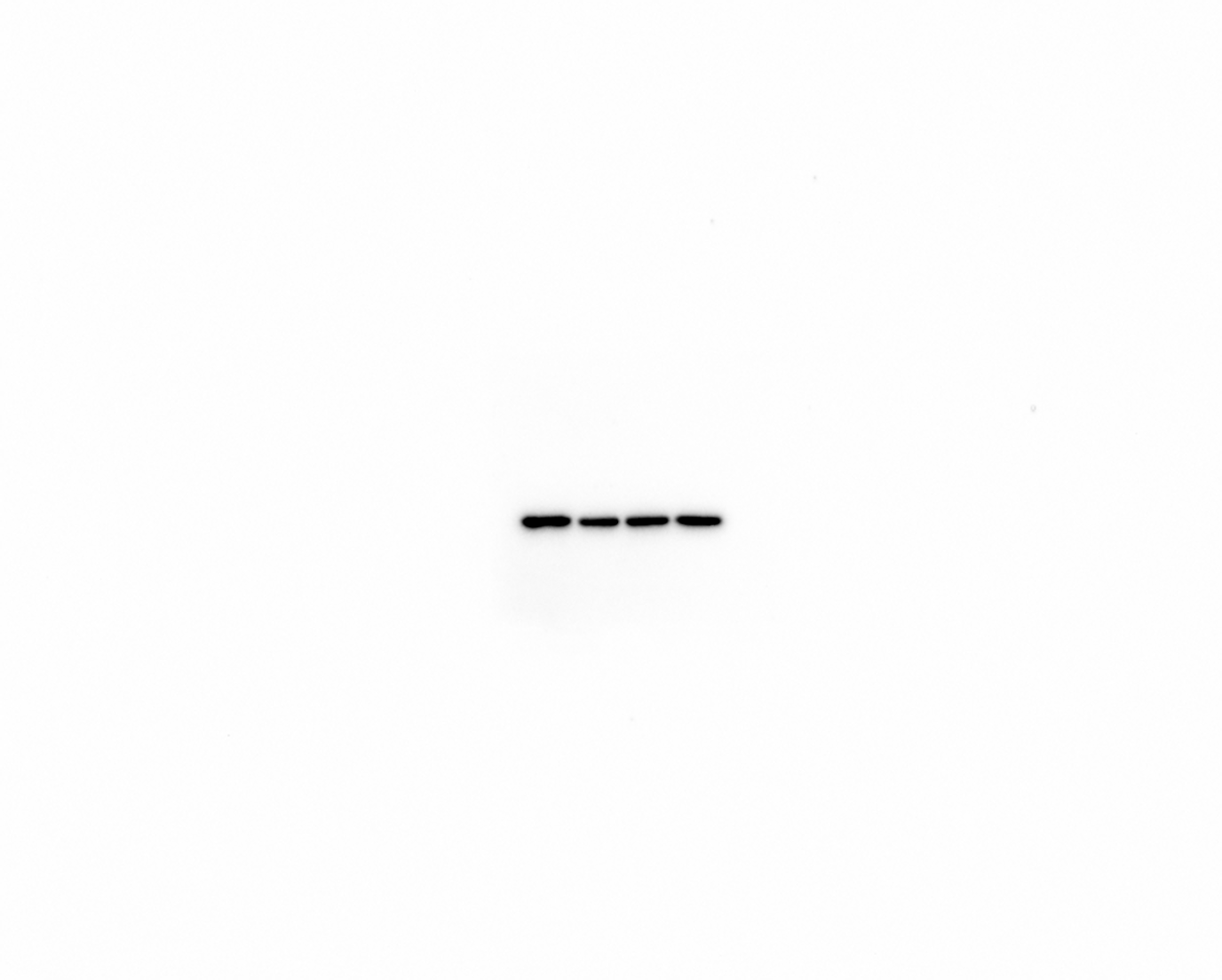

Supplement: Supplementary file 1 [file DataSheet3.zip › 原图1/KCNK3/2.Tif]

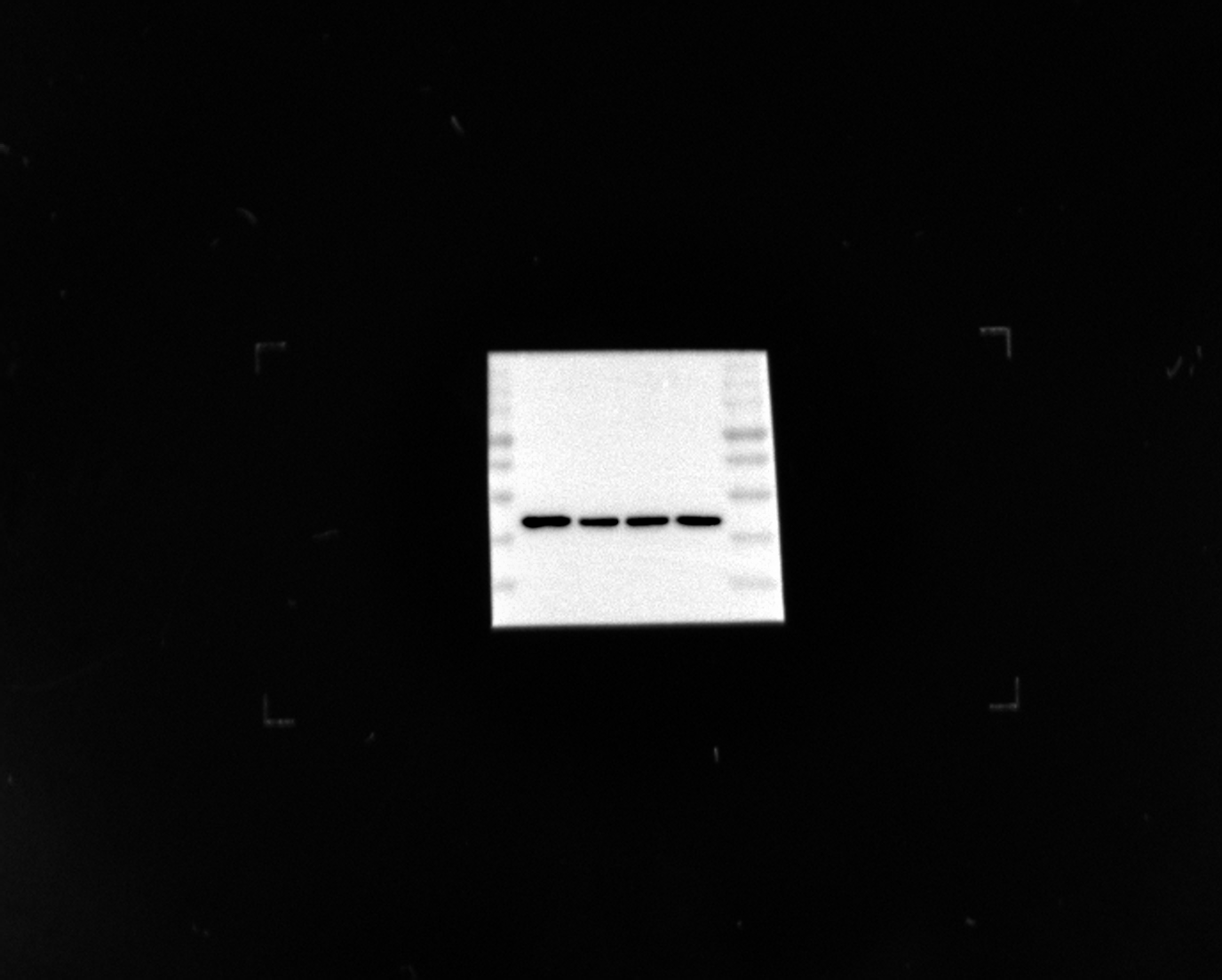

Supplement: Supplementary file 1 [file DataSheet3.zip › 原图1/KCNK3/2副本.tif]

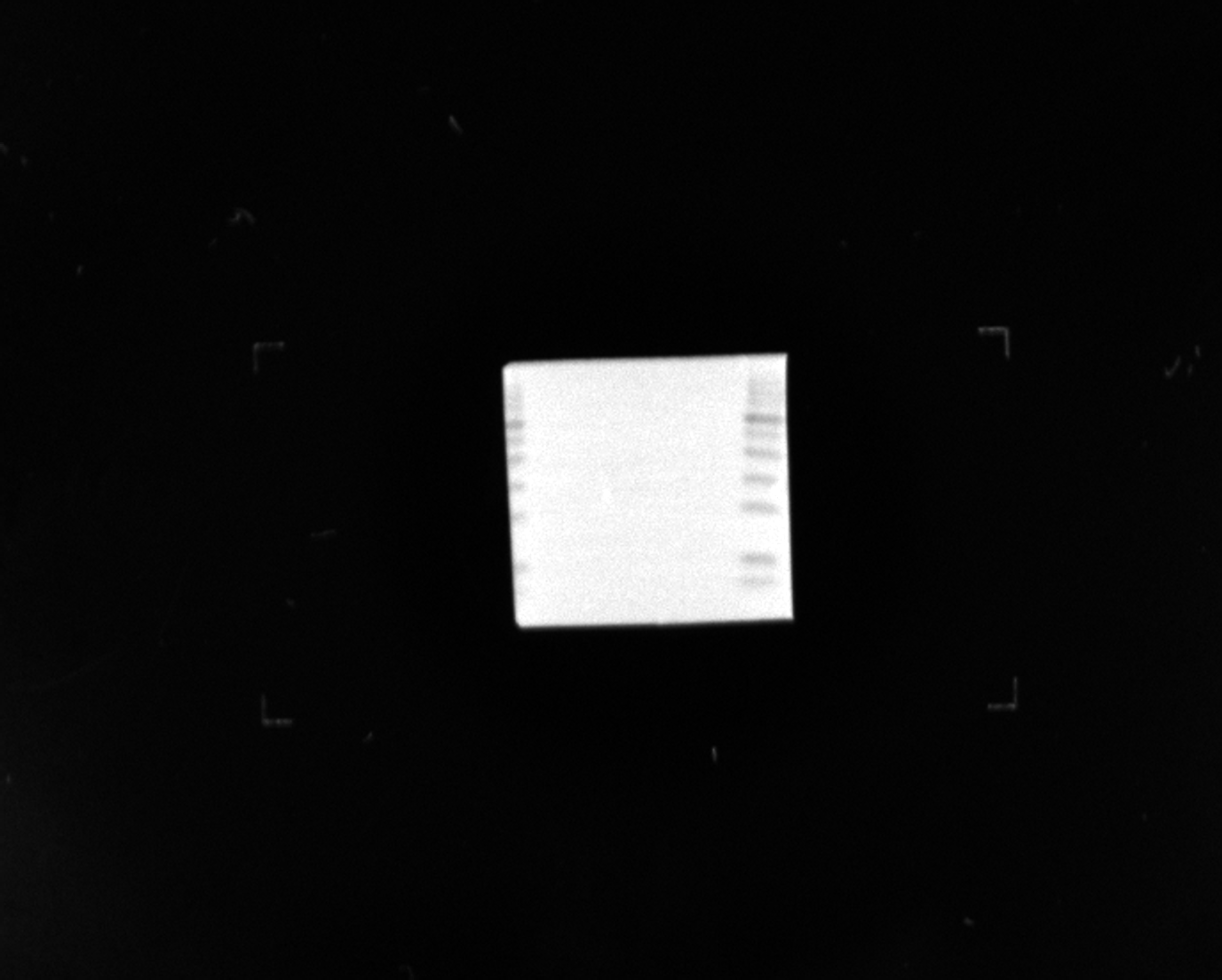

Supplement: Supplementary file 1 [file DataSheet3.zip › 原图1/KCNK3/3-t.Tif]

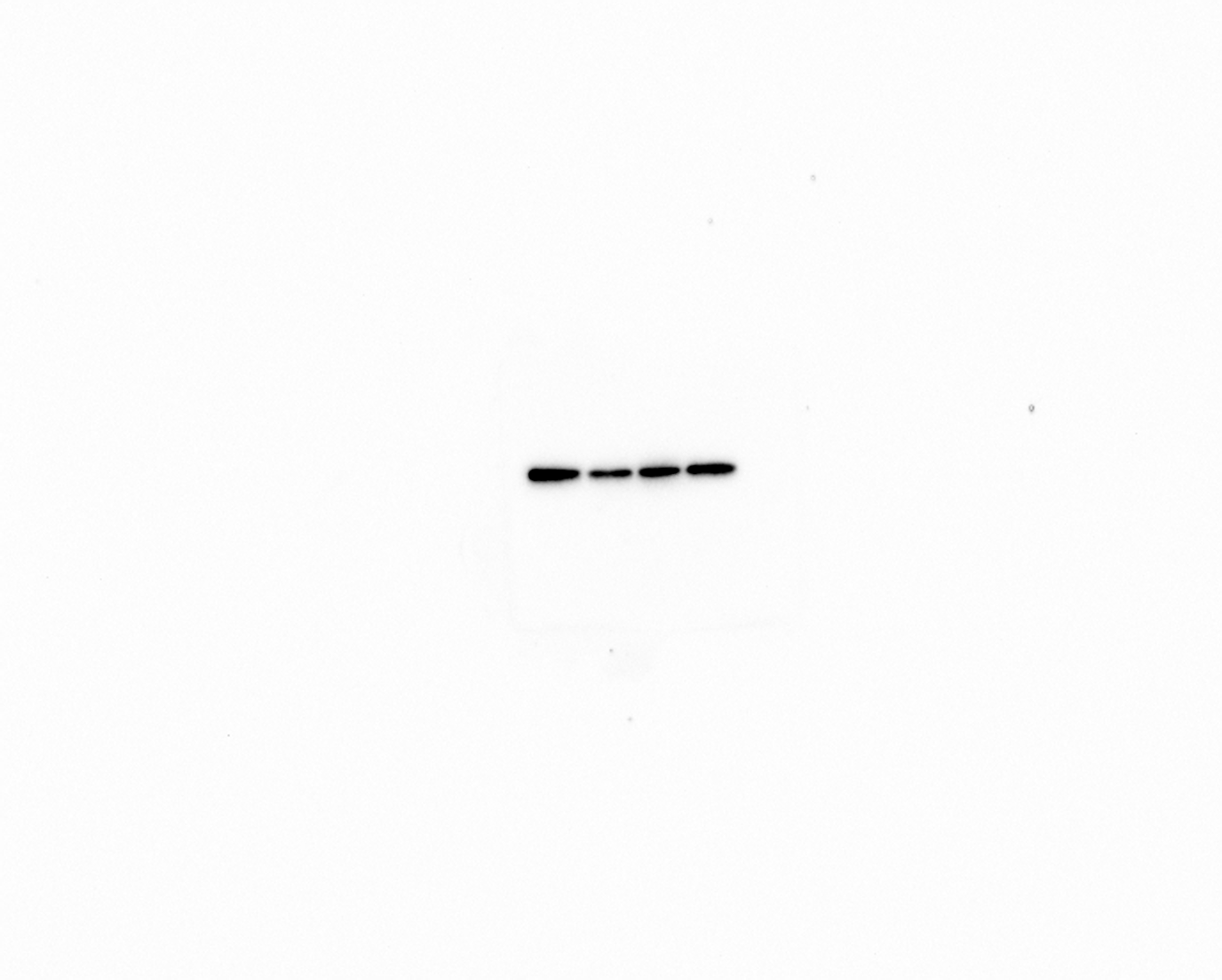

Supplement: Supplementary file 1 [file DataSheet3.zip › 原图1/KCNK3/3.Tif]

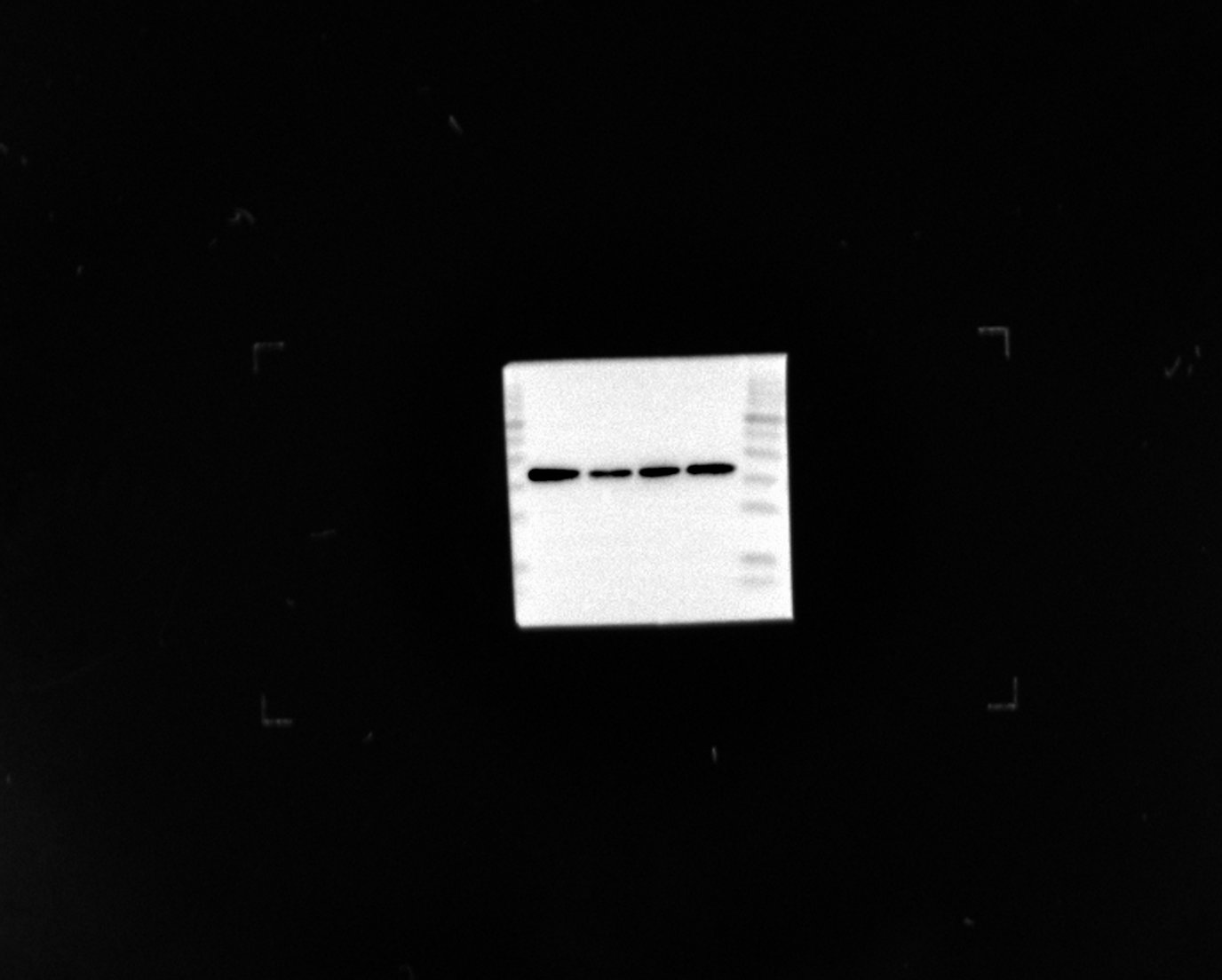

Supplement: Supplementary file 1 [file DataSheet3.zip › 原图1/KCNK3/3副本.tif]

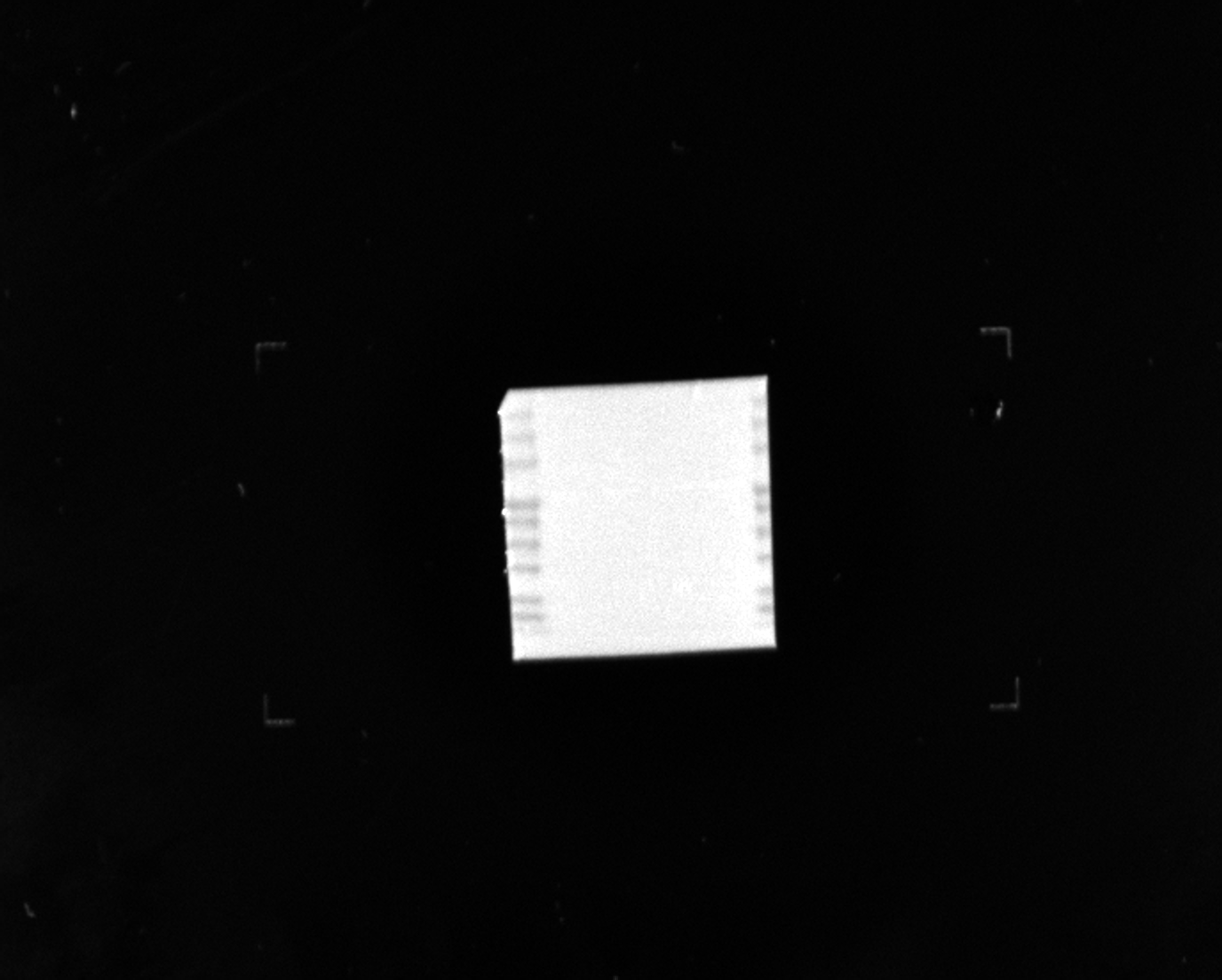

Supplement: Supplementary file 1 [file DataSheet3.zip › 原图1/NLRP3/1-t.Tif]

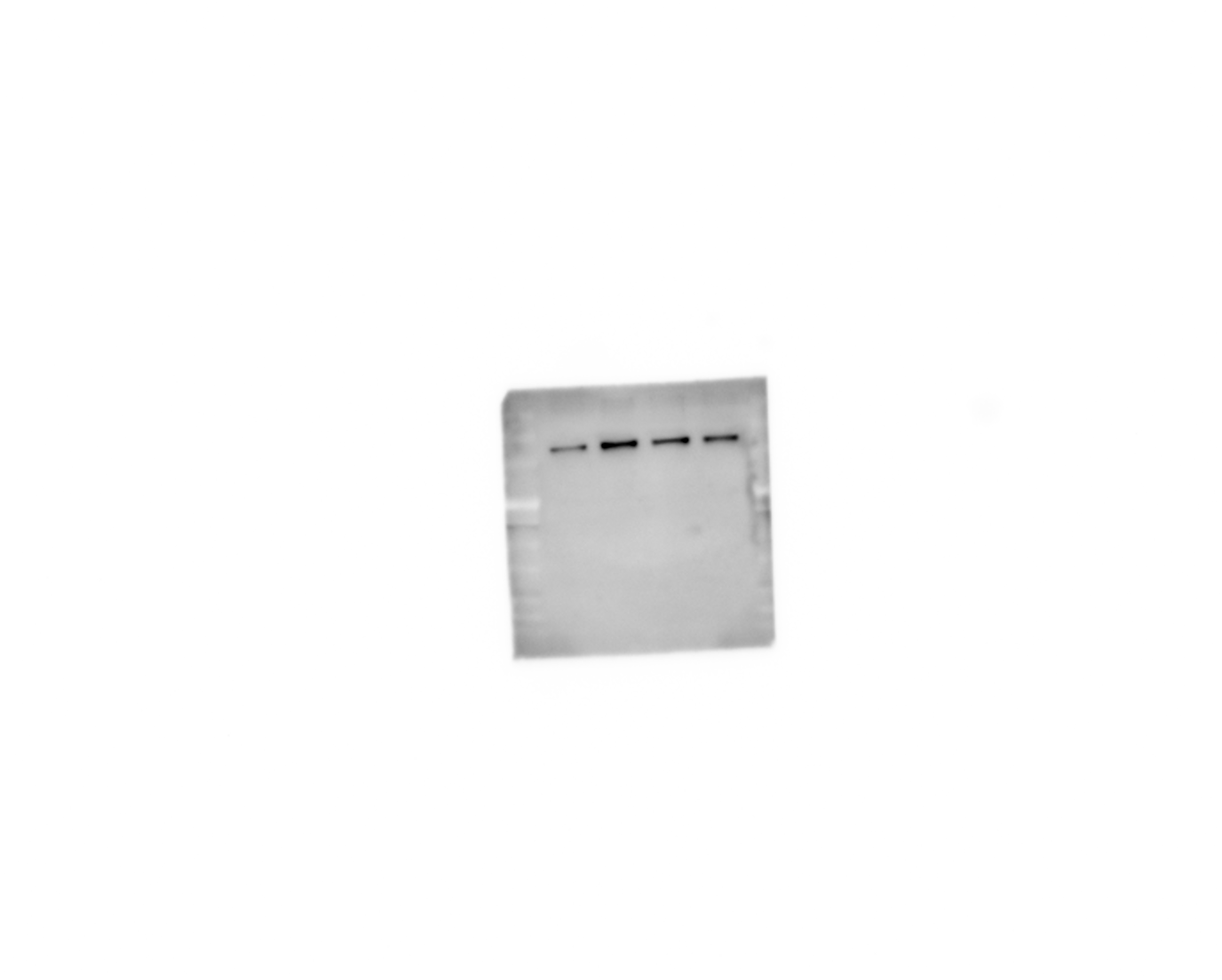

Supplement: Supplementary file 1 [file DataSheet3.zip › 原图1/NLRP3/1.Tif]

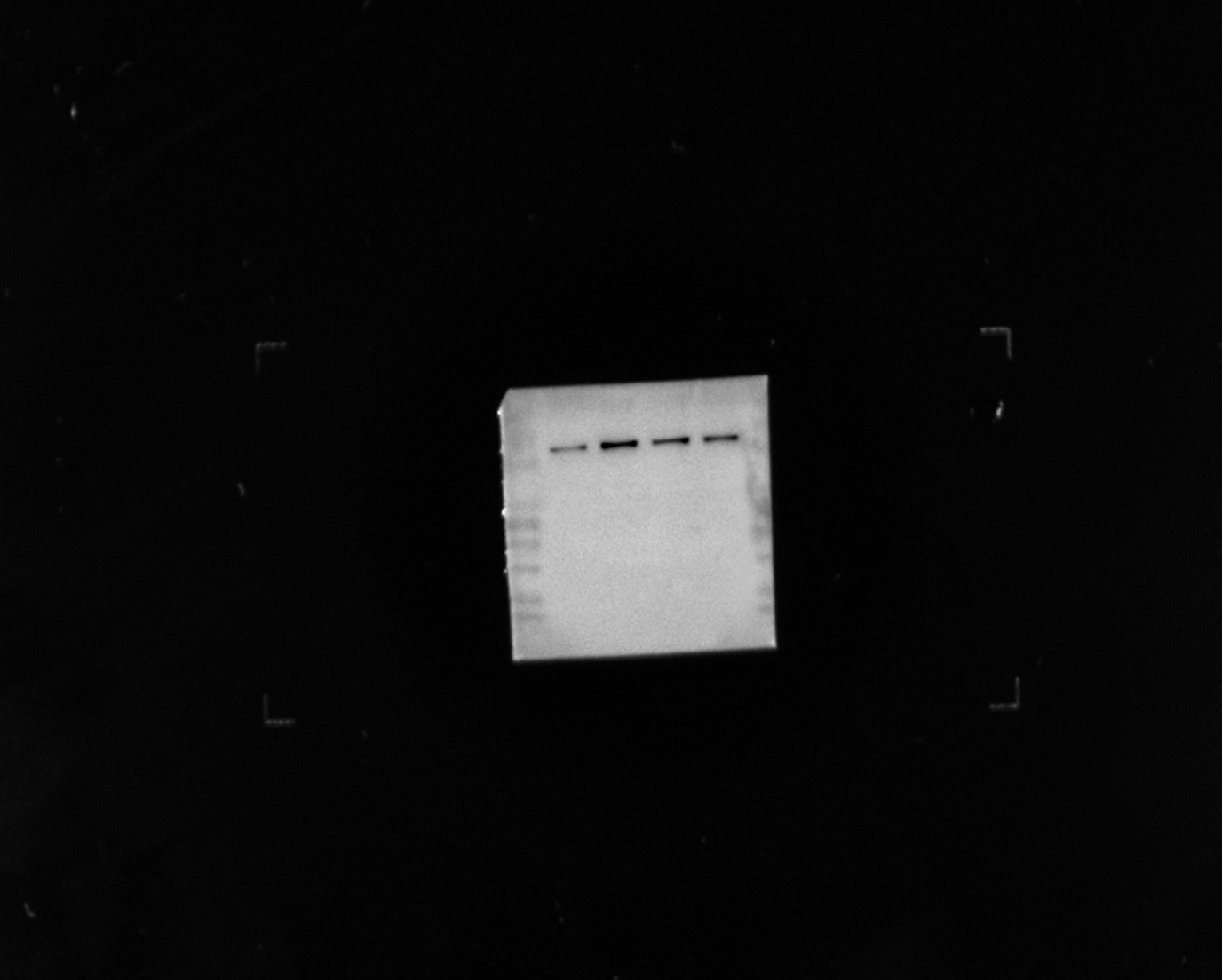

Supplement: Supplementary file 1 [file DataSheet3.zip › 原图1/NLRP3/1副本.tif]

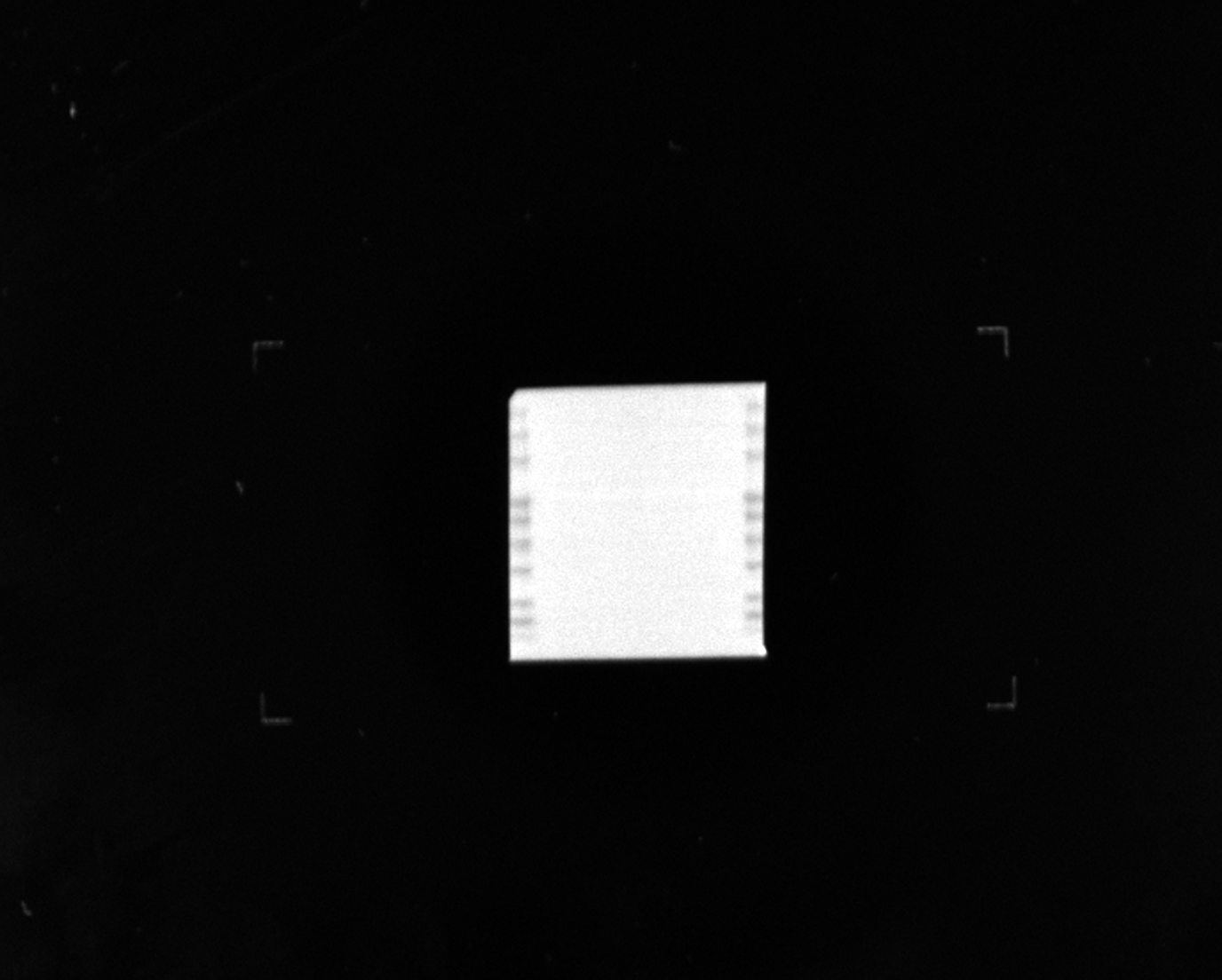

Supplement: Supplementary file 1 [file DataSheet3.zip › 原图1/NLRP3/2-t.Tif]

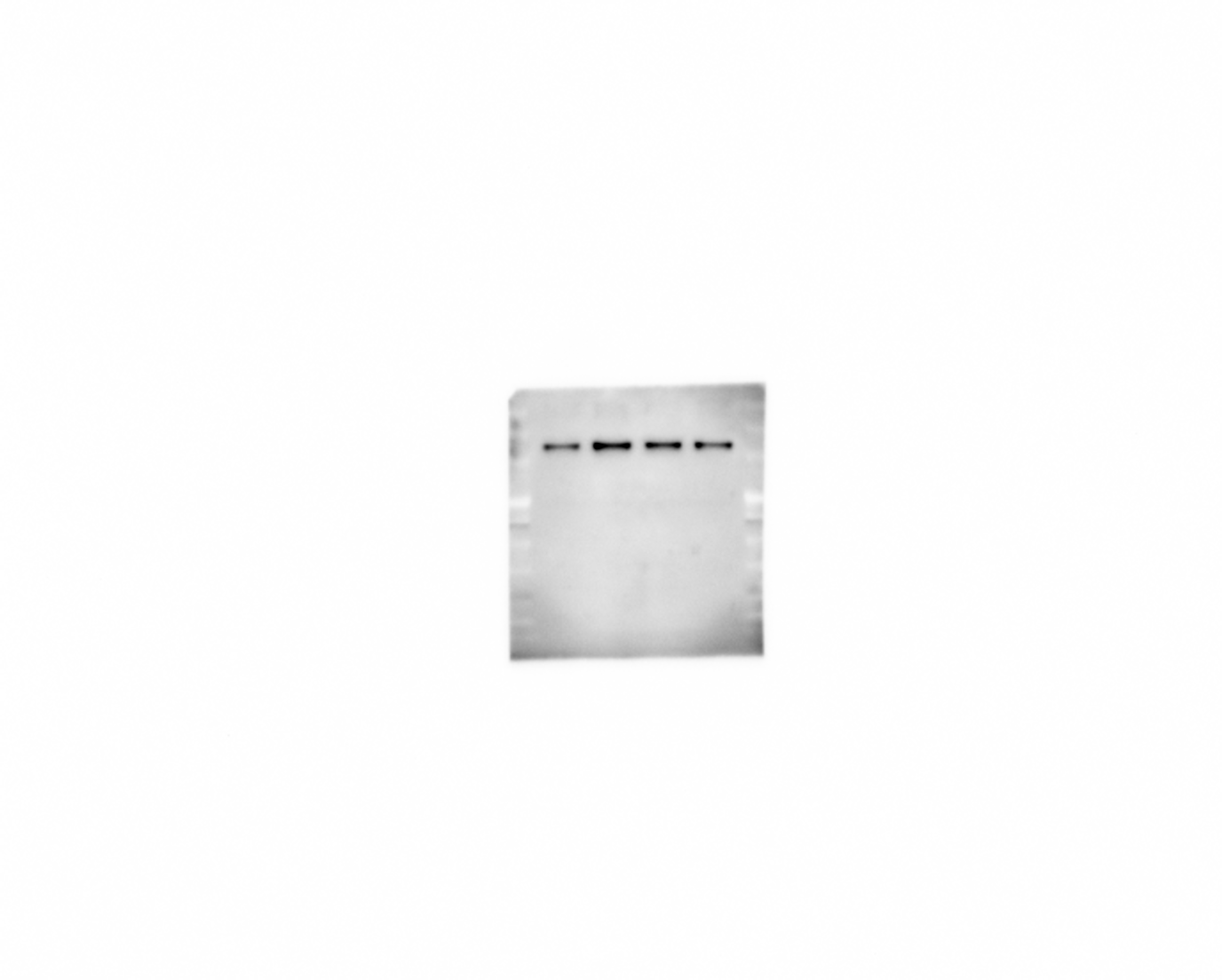

Supplement: Supplementary file 1 [file DataSheet3.zip › 原图1/NLRP3/2.Tif]

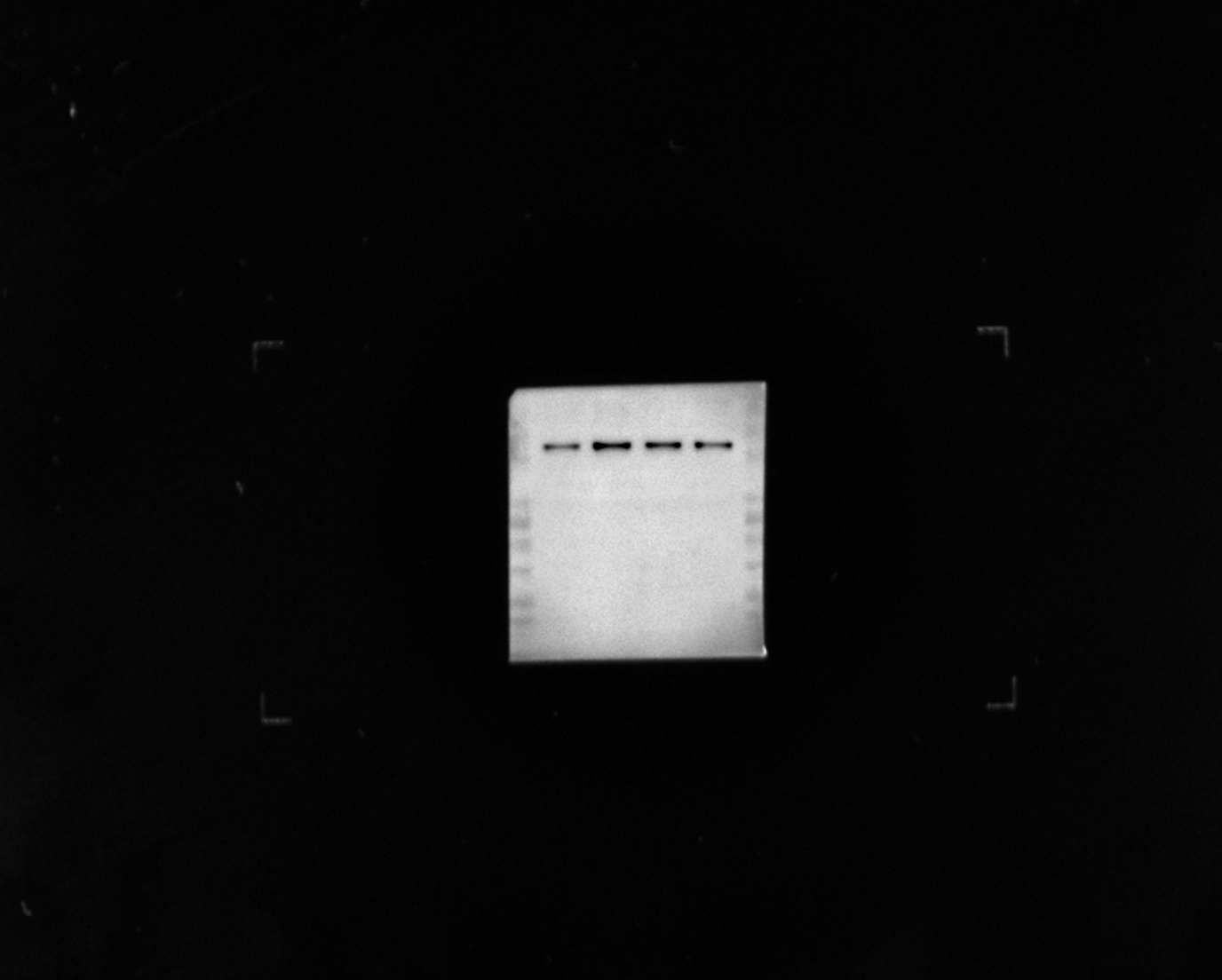

Supplement: Supplementary file 1 [file DataSheet3.zip › 原图1/NLRP3/2副本.tif]

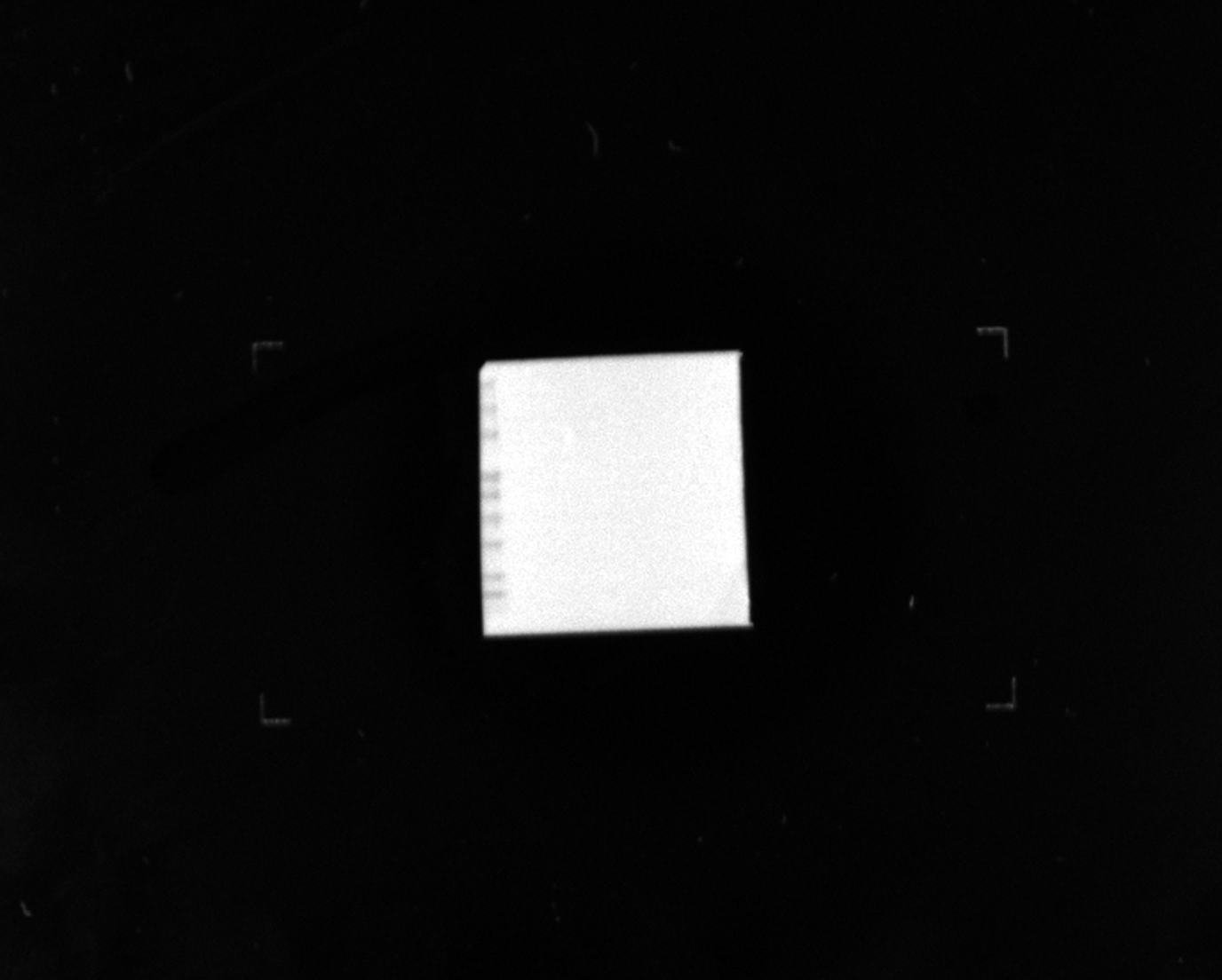

Supplement: Supplementary file 1 [file DataSheet3.zip › 原图1/NLRP3/3-t.Tif]

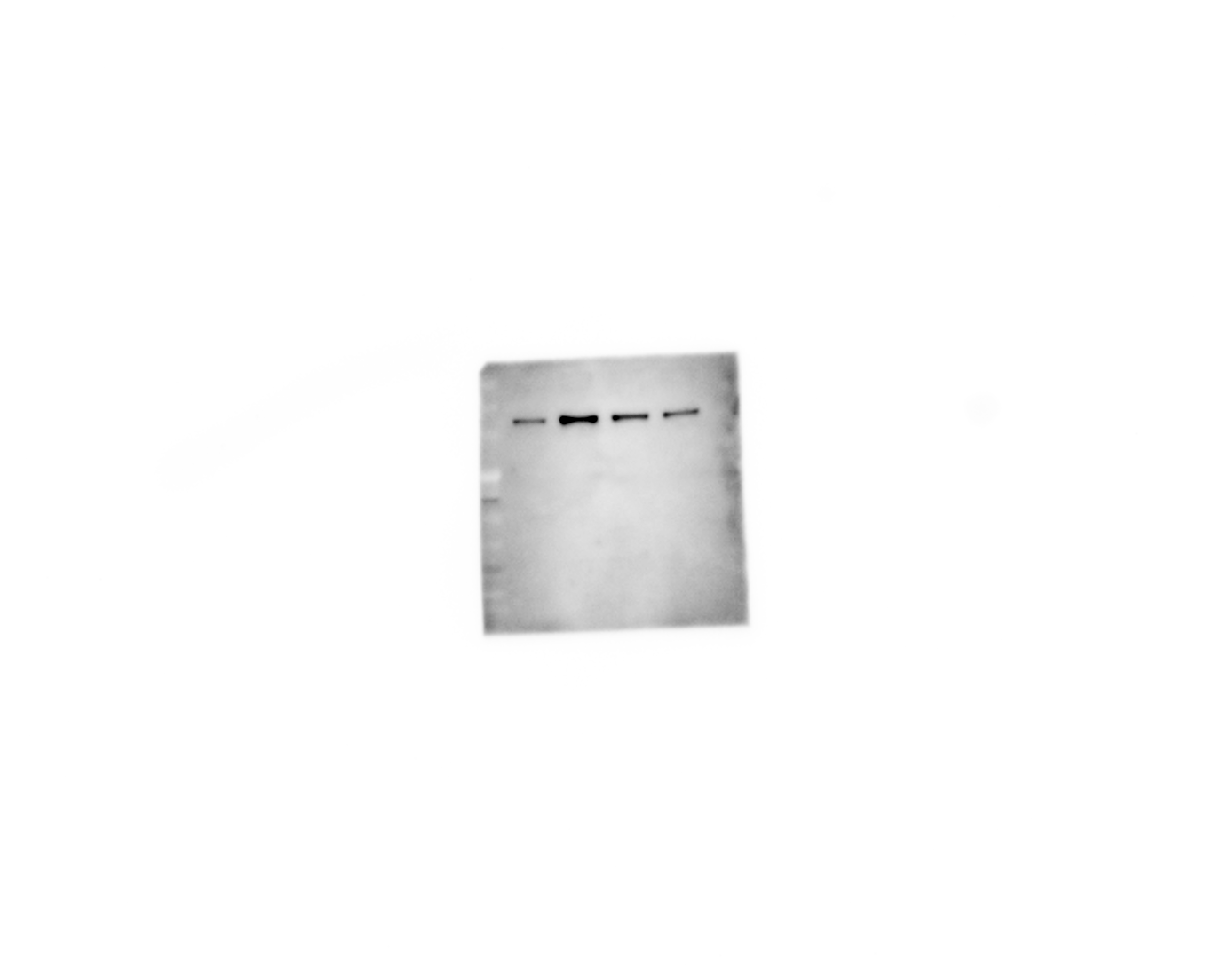

Supplement: Supplementary file 1 [file DataSheet3.zip › 原图1/NLRP3/3.Tif]

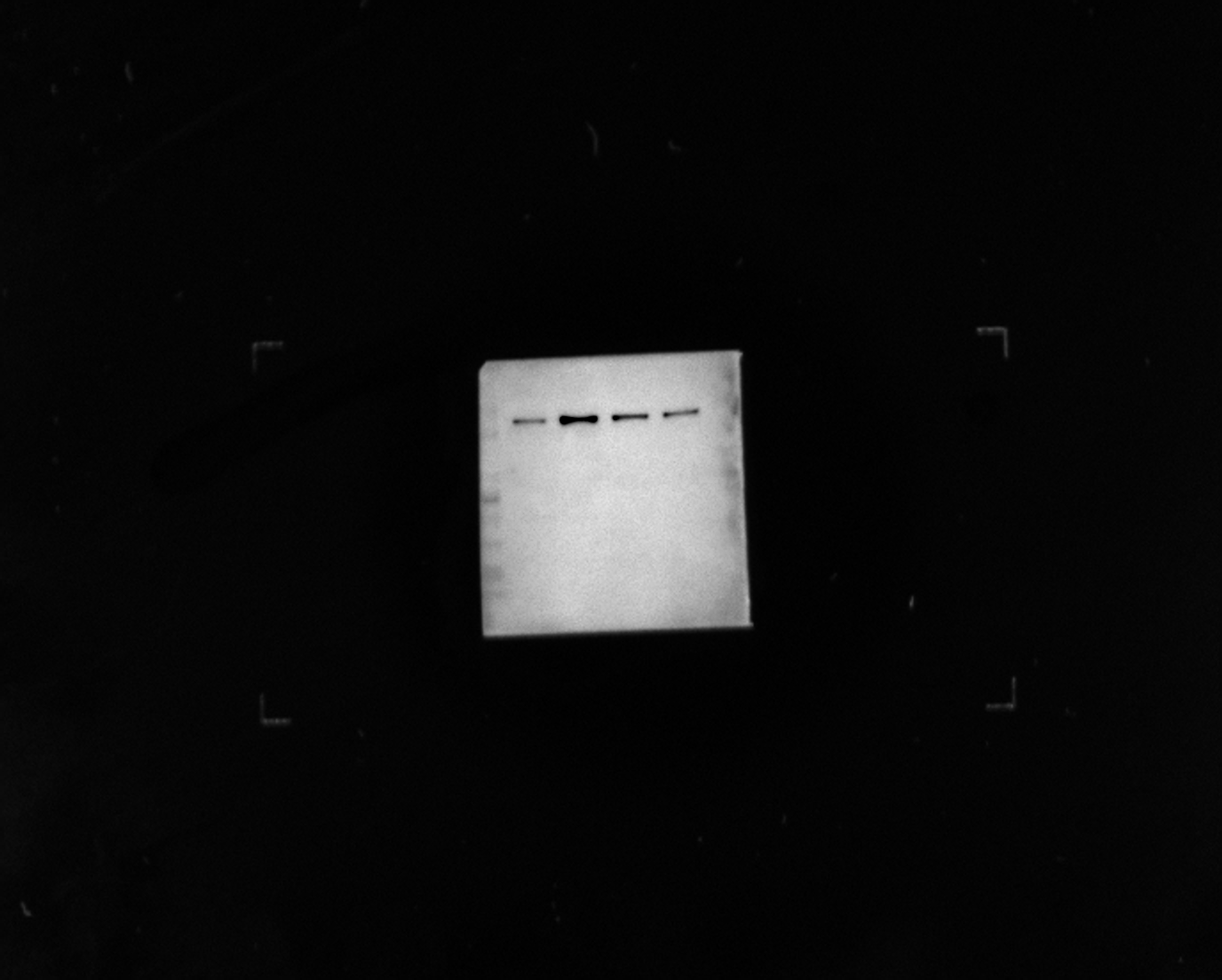

Supplement: Supplementary file 1 [file DataSheet3.zip › 原图1/NLRP3/3副本.tif]

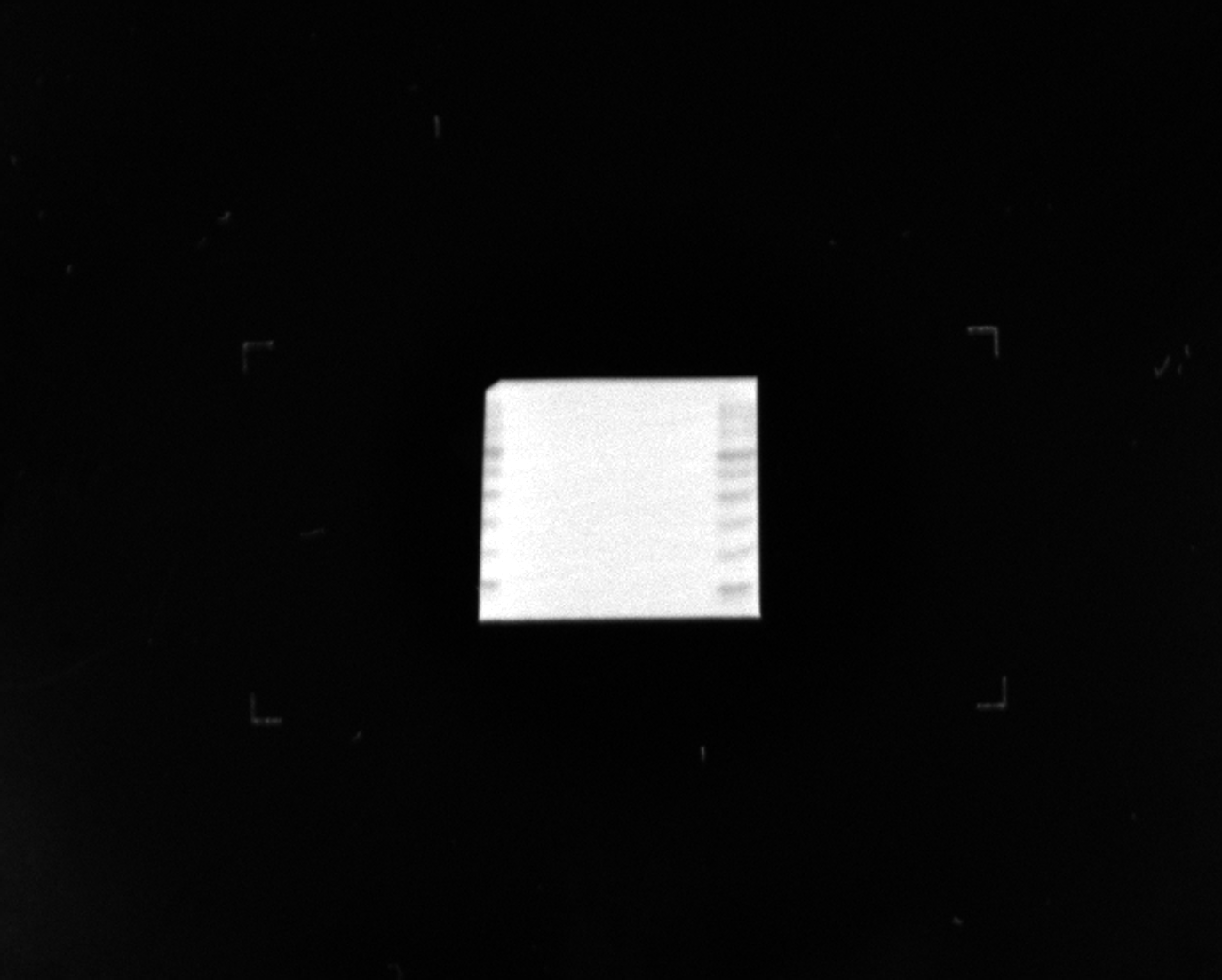

Supplement: Supplementary file 1 [file DataSheet3.zip › 原图1/OAT3/1-t.Tif]

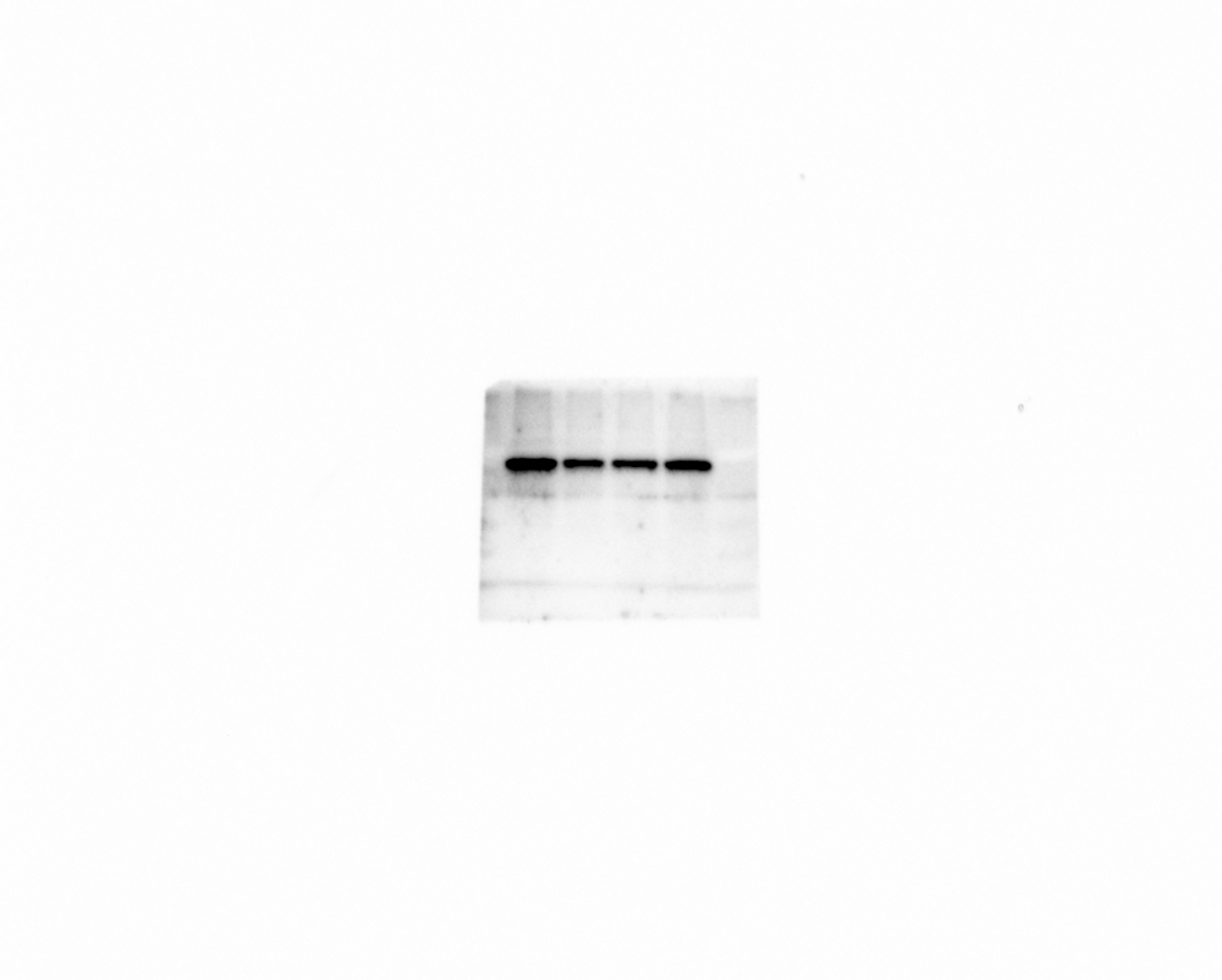

Supplement: Supplementary file 1 [file DataSheet3.zip › 原图1/OAT3/1.Tif]

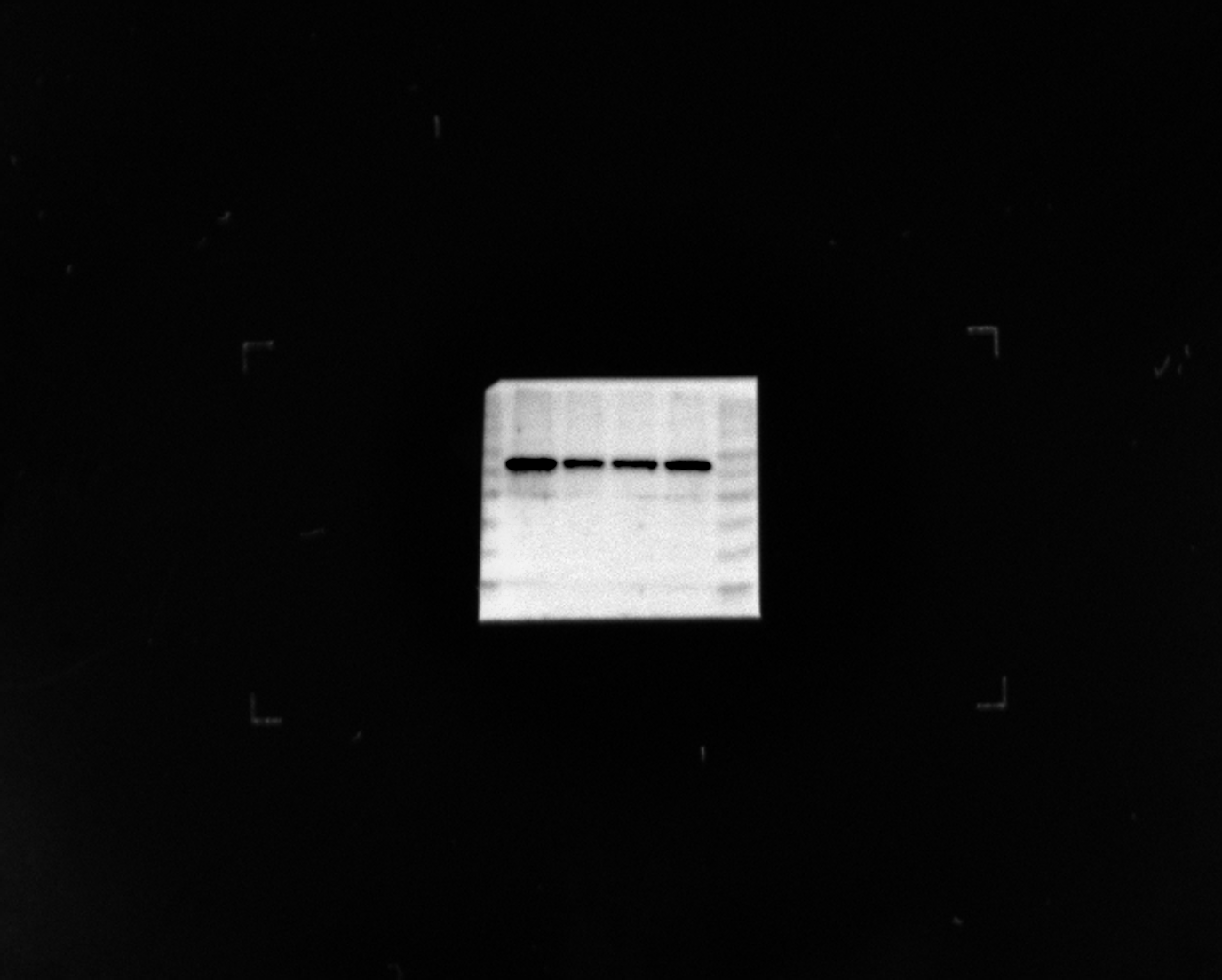

Supplement: Supplementary file 1 [file DataSheet3.zip › 原图1/OAT3/1副本.tif]

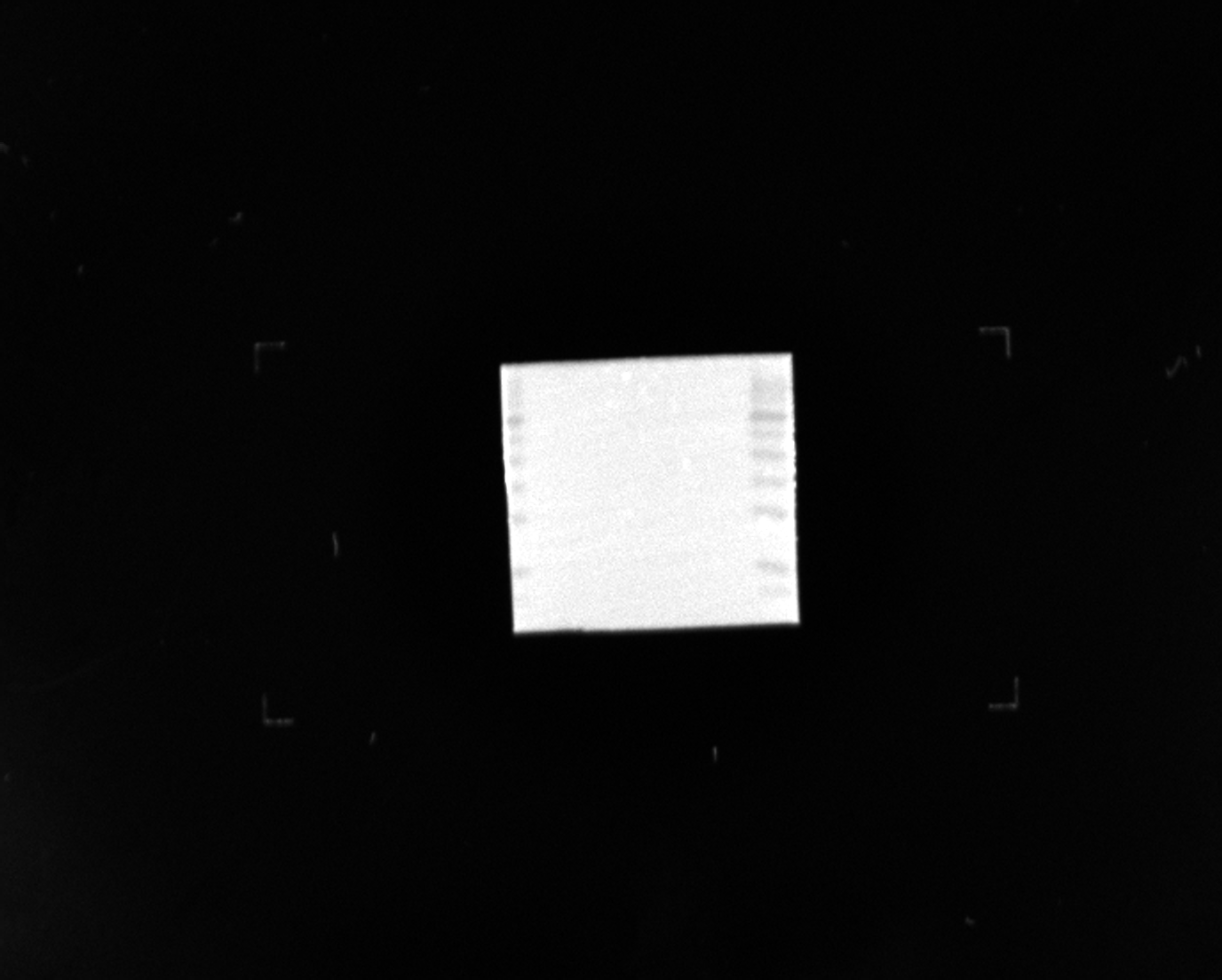

Supplement: Supplementary file 1 [file DataSheet3.zip › 原图1/OAT3/2-t.Tif]

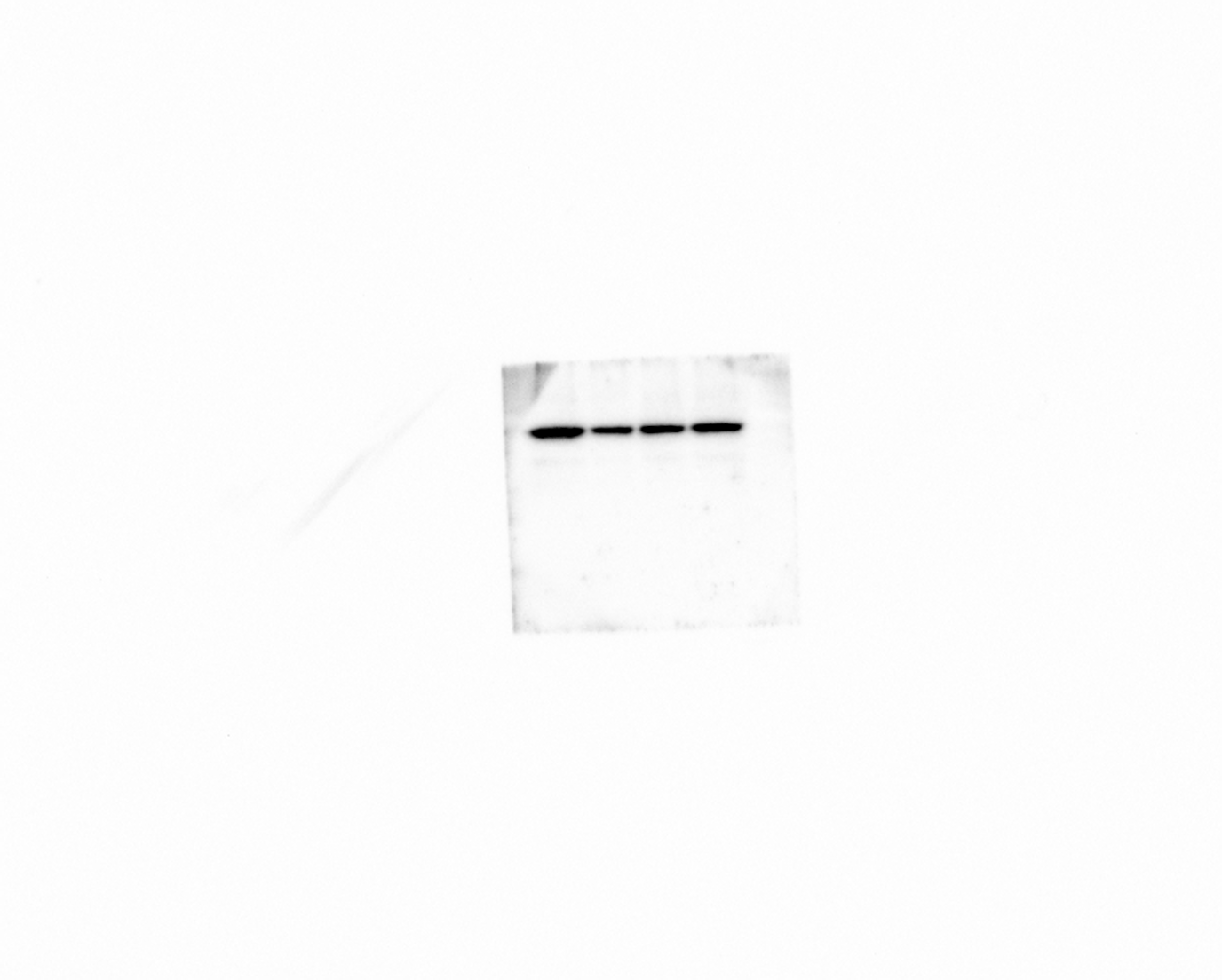

Supplement: Supplementary file 1 [file DataSheet3.zip › 原图1/OAT3/2.Tif]

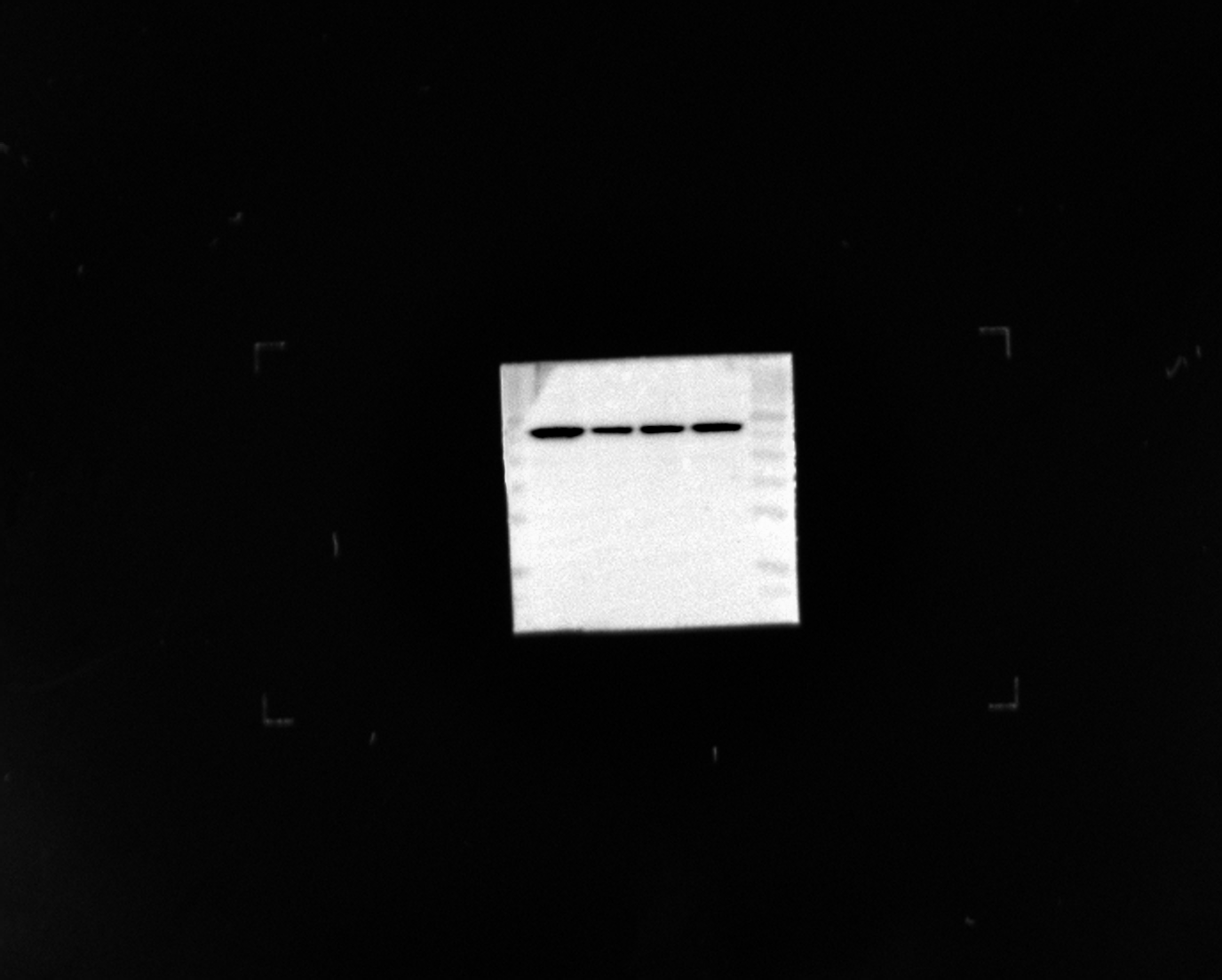

Supplement: Supplementary file 1 [file DataSheet3.zip › 原图1/OAT3/2副本.tif]

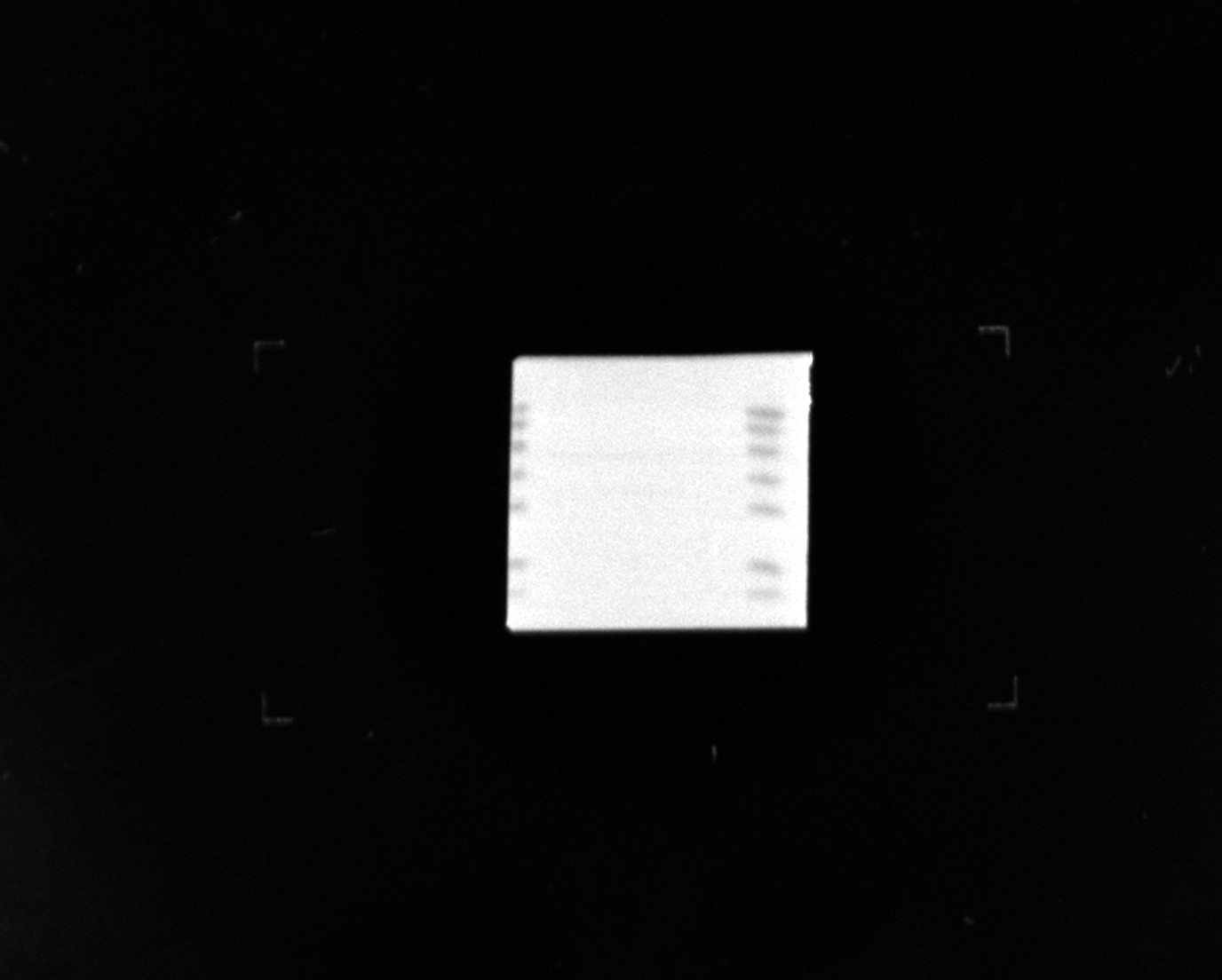

Supplement: Supplementary file 1 [file DataSheet3.zip › 原图1/OAT3/3-t.Tif]

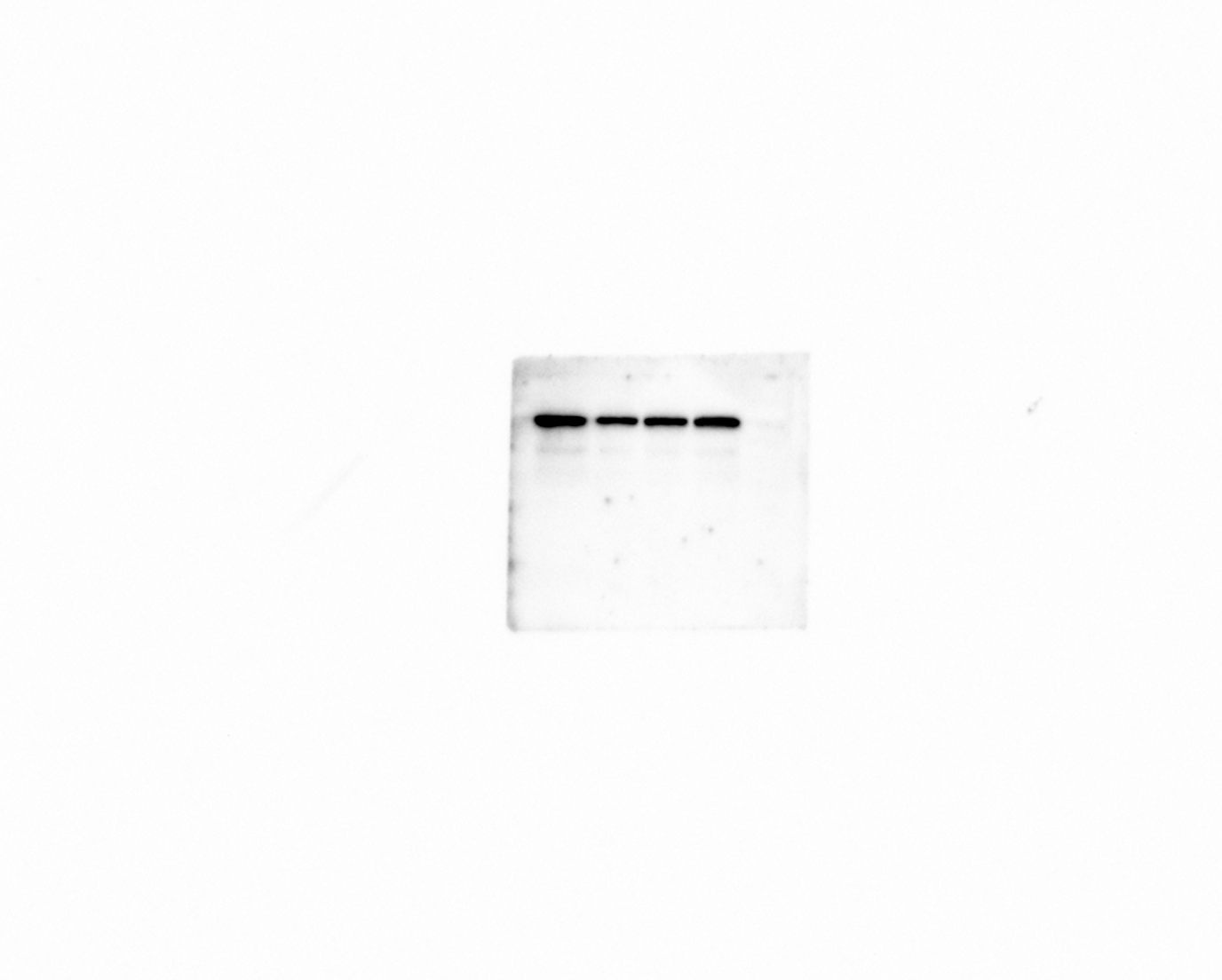

Supplement: Supplementary file 1 [file DataSheet3.zip › 原图1/OAT3/3.Tif]

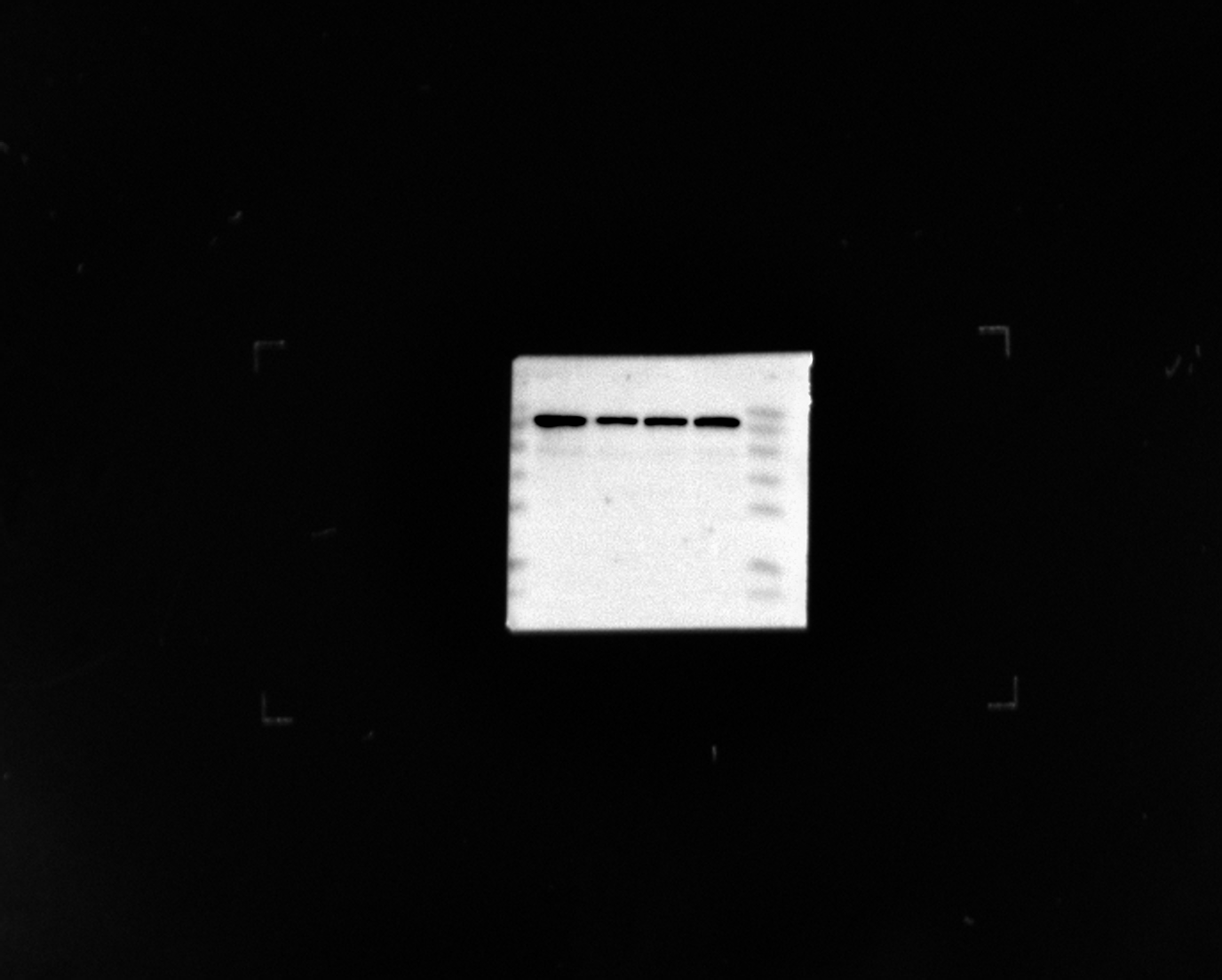

Supplement: Supplementary file 1 [file DataSheet3.zip › 原图1/OAT3/3副本.tif]

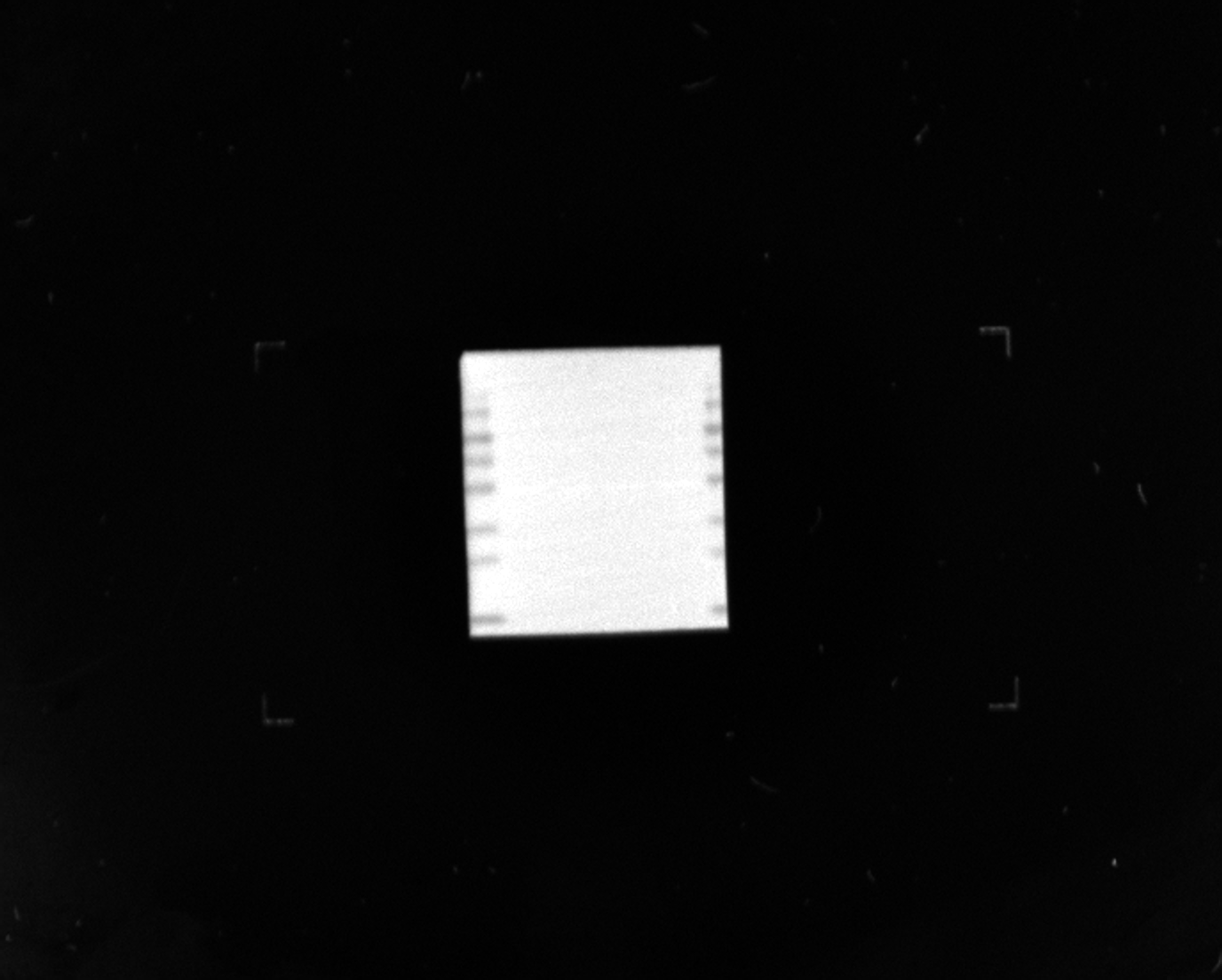

Supplement: Supplementary file 1 [file DataSheet3.zip › 原图1/β-actin/1-t.Tif]

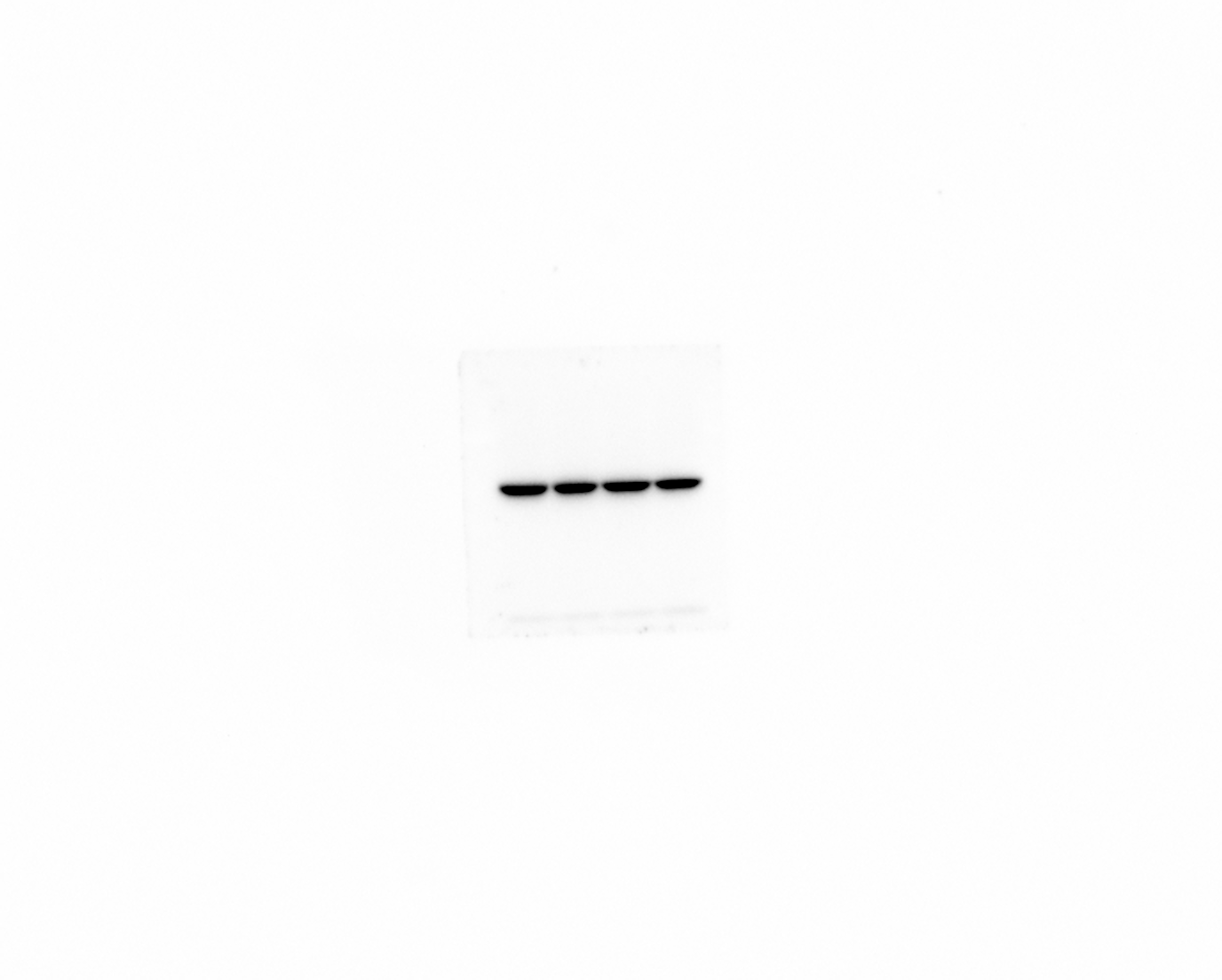

Supplement: Supplementary file 1 [file DataSheet3.zip › 原图1/β-actin/1.Tif]

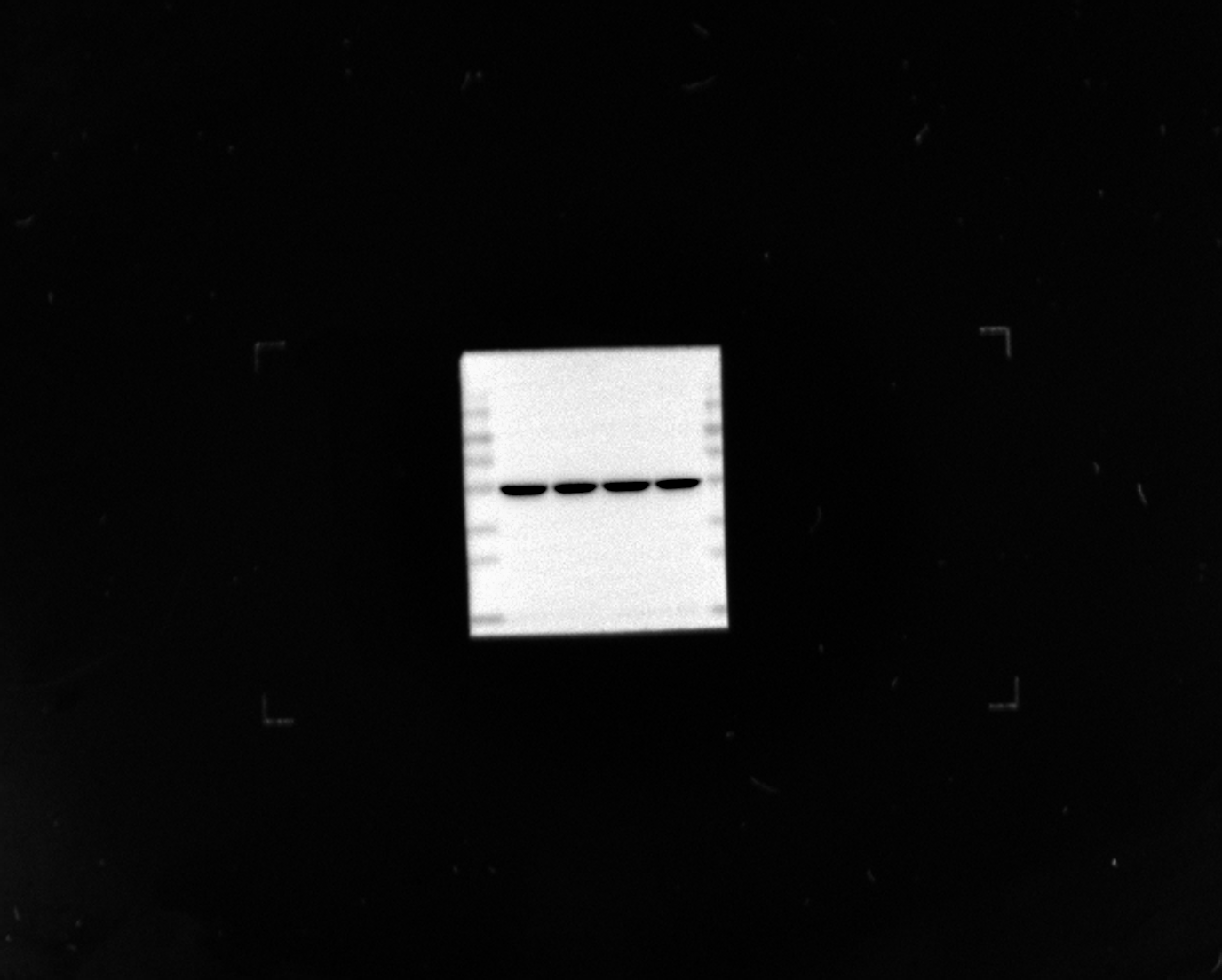

Supplement: Supplementary file 1 [file DataSheet3.zip › 原图1/β-actin/1副本.tif]

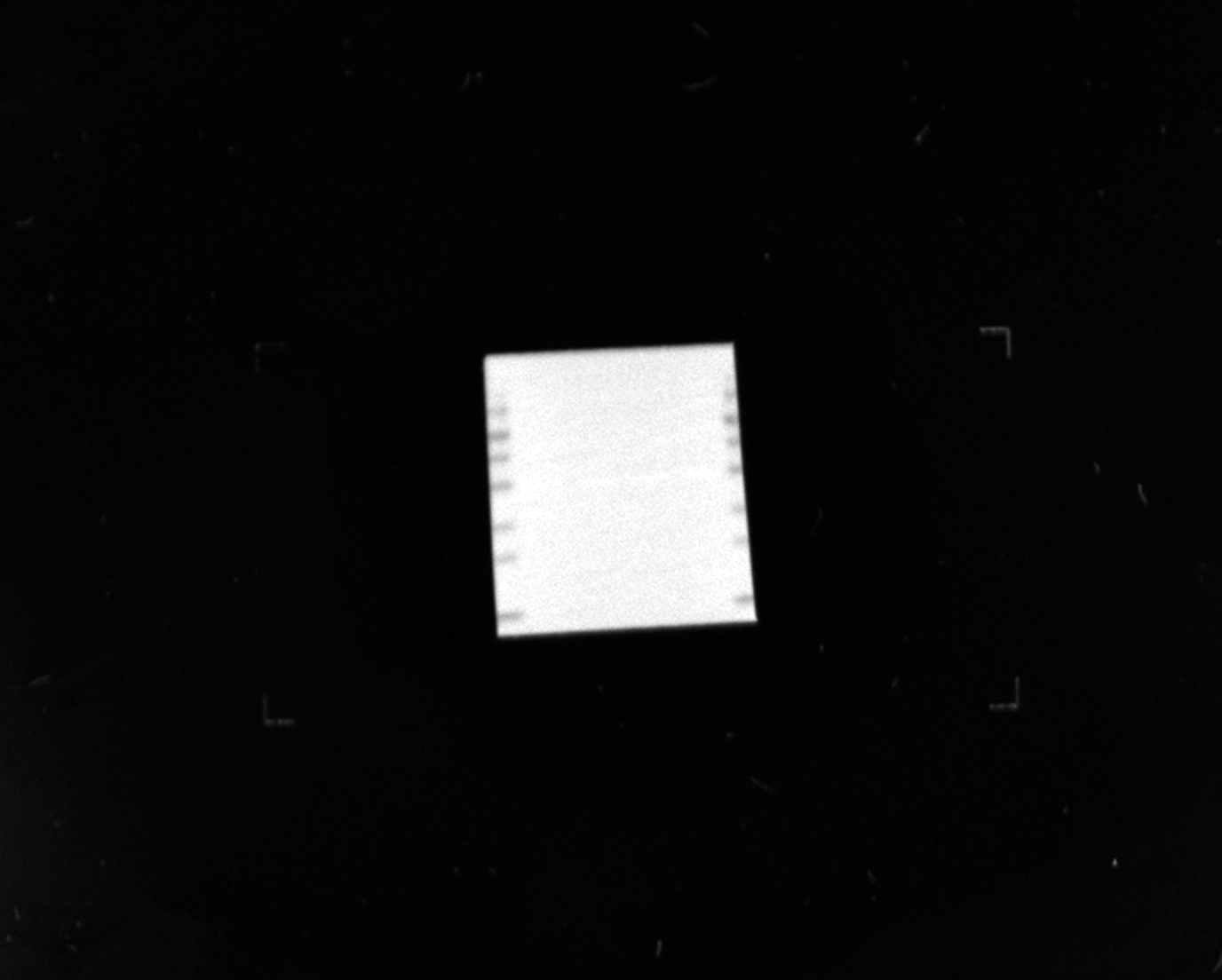

Supplement: Supplementary file 1 [file DataSheet3.zip › 原图1/β-actin/2-t.Tif]

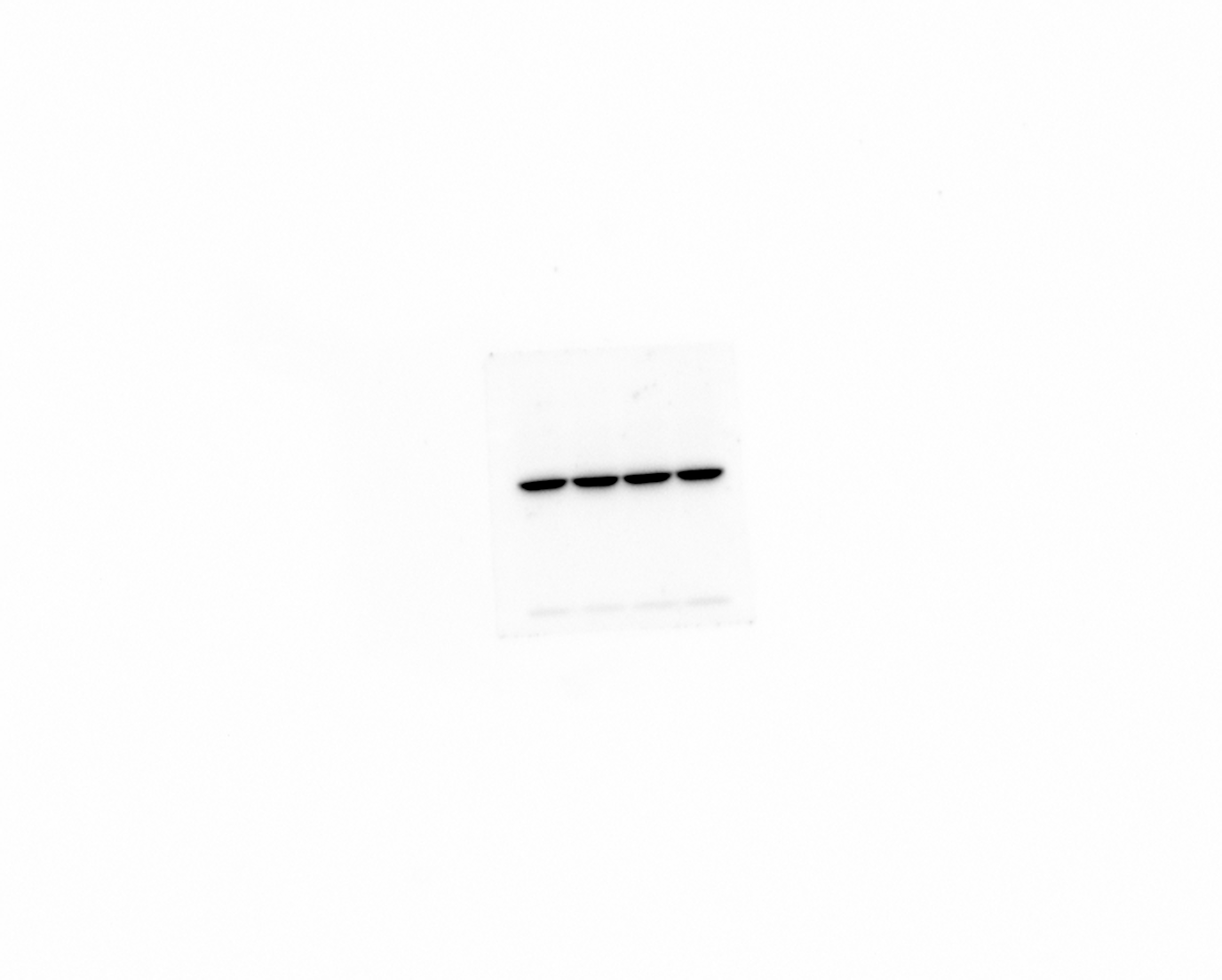

Supplement: Supplementary file 1 [file DataSheet3.zip › 原图1/β-actin/2.Tif]

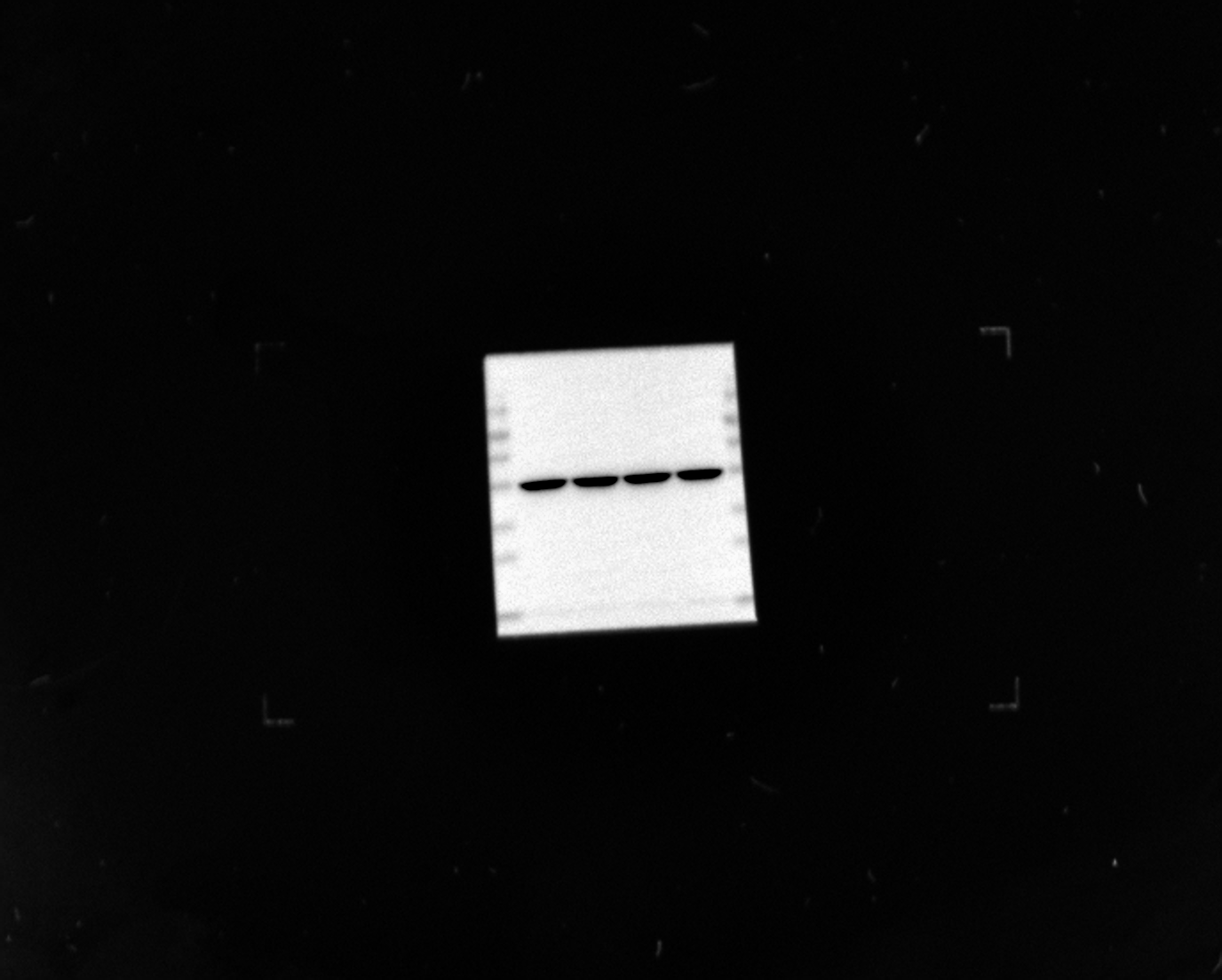

Supplement: Supplementary file 1 [file DataSheet3.zip › 原图1/β-actin/2副本.tif]

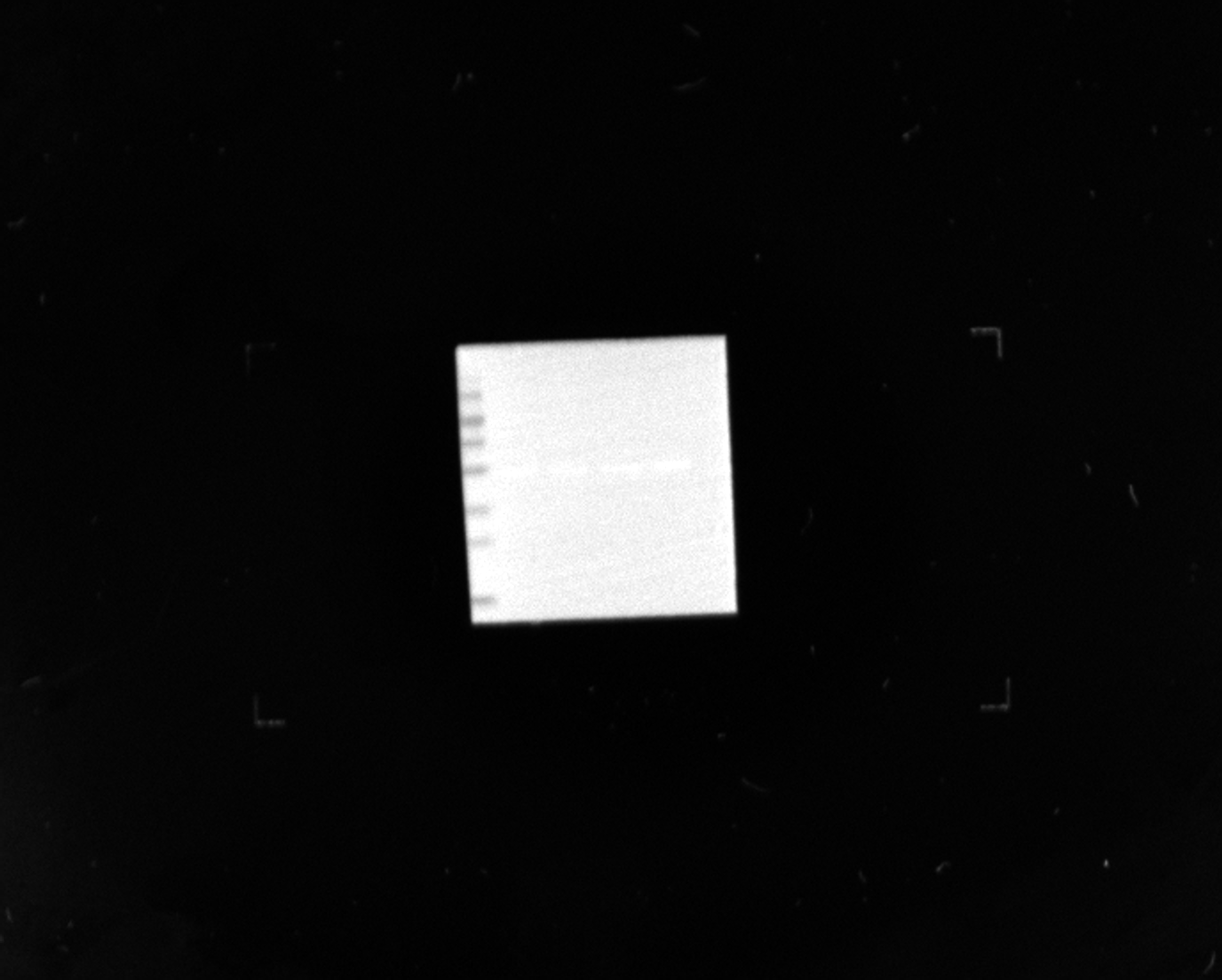

Supplement: Supplementary file 1 [file DataSheet3.zip › 原图1/β-actin/3-t.Tif]

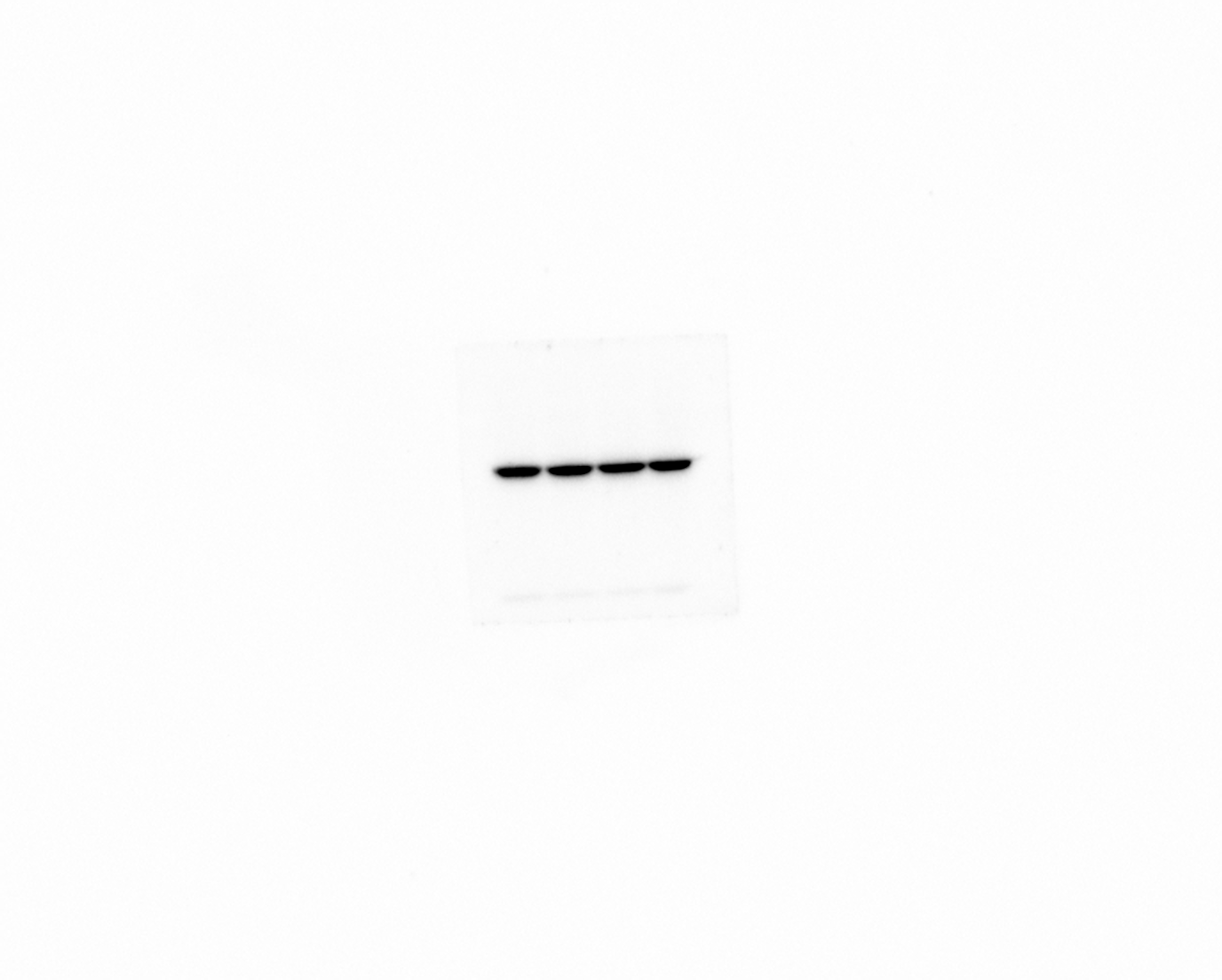

Supplement: Supplementary file 1 [file DataSheet3.zip › 原图1/β-actin/3.Tif]

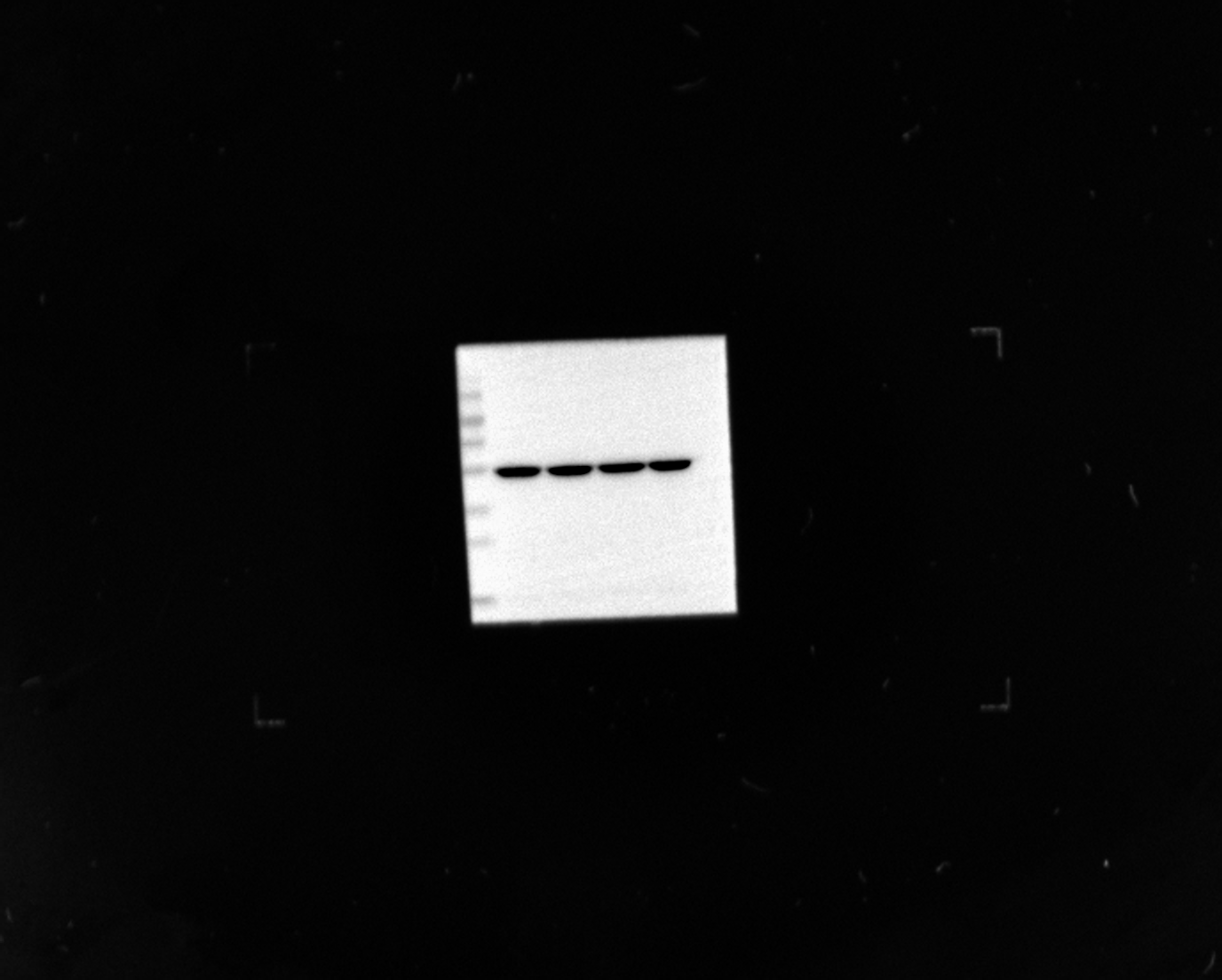

Supplement: Supplementary file 1 [file DataSheet3.zip › 原图1/β-actin/3副本.tif]

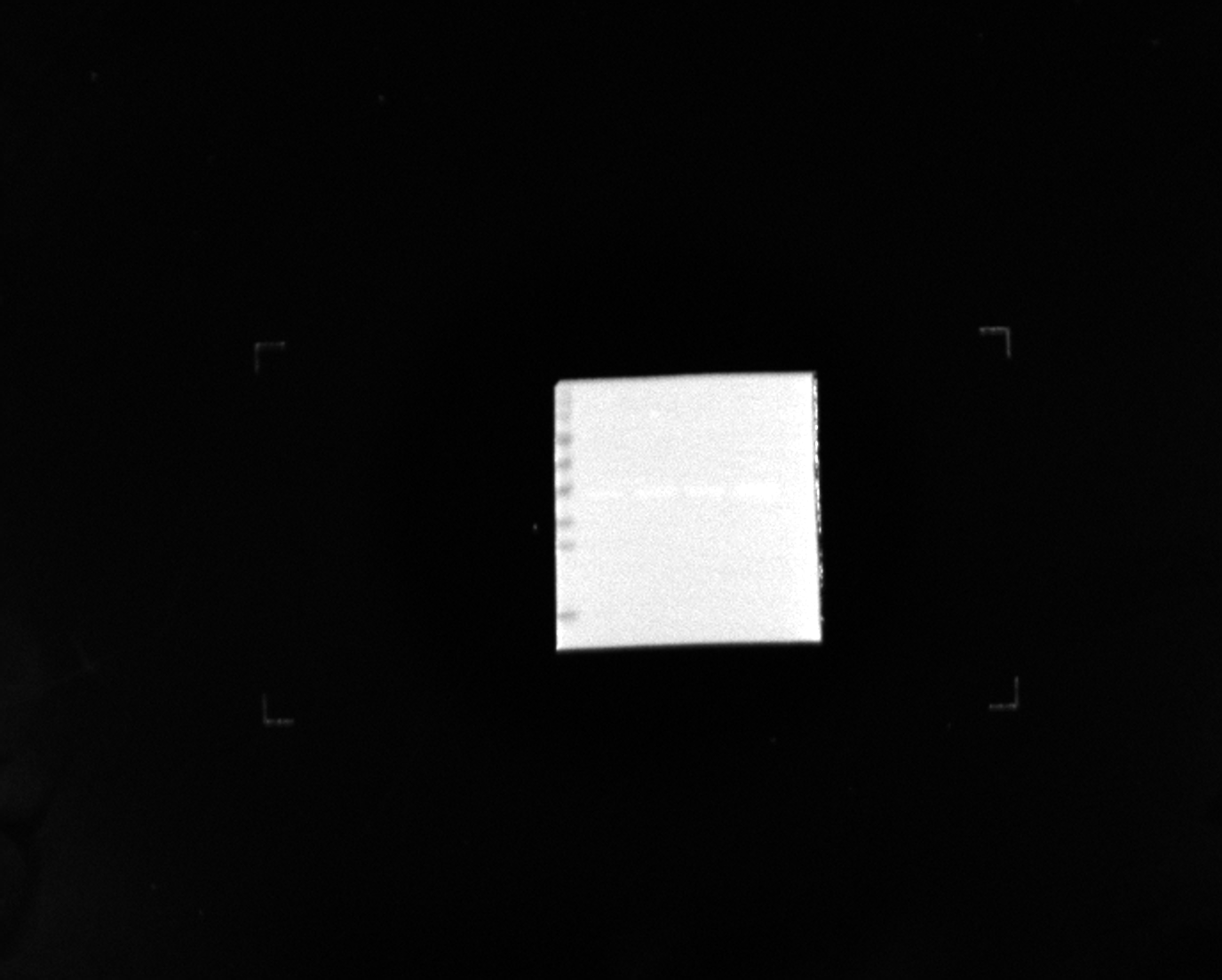

Supplement: Supplementary file 2 [file DataSheet1.zip › IDO/IDO1/1-t.Tif]

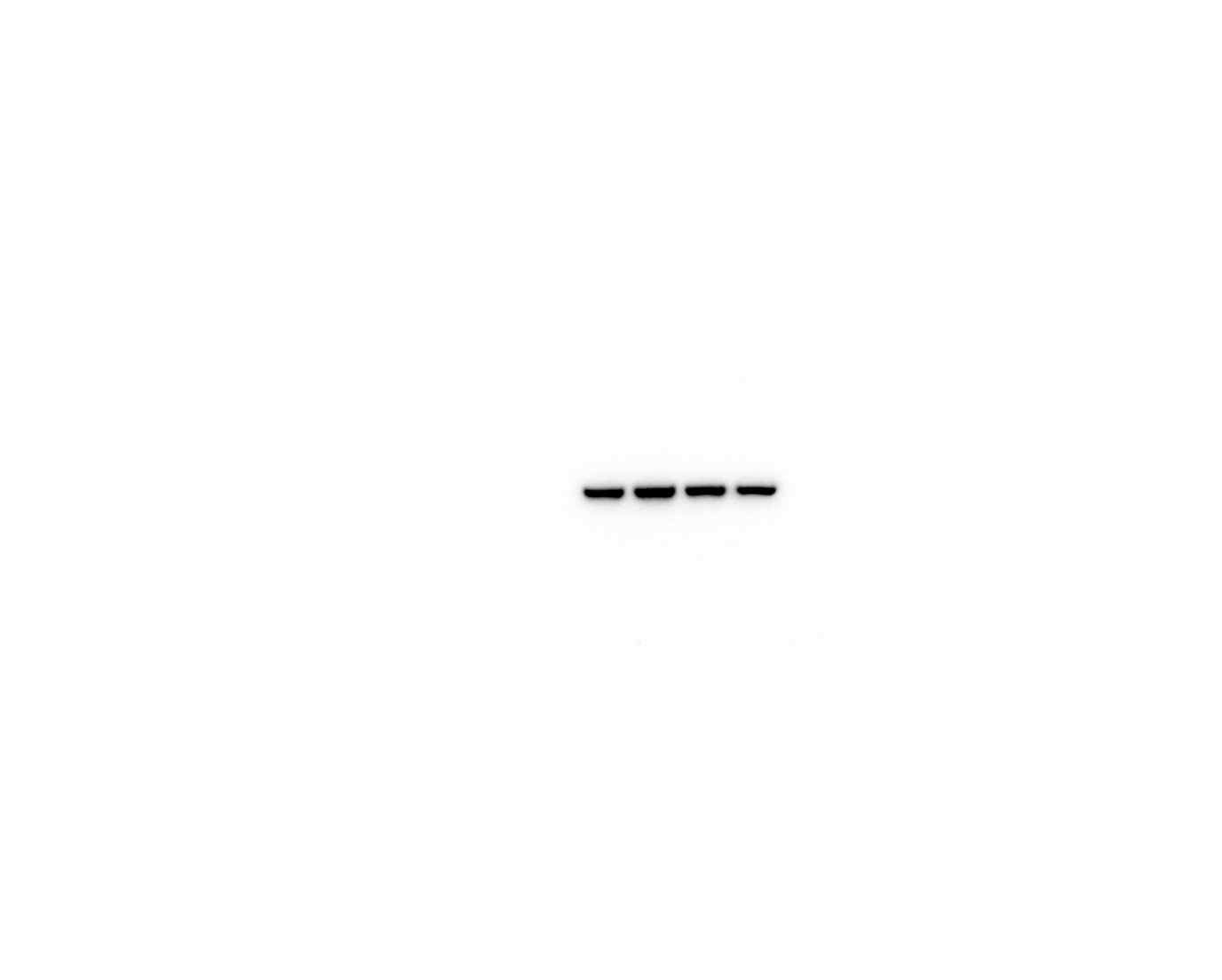

Supplement: Supplementary file 2 [file DataSheet1.zip › IDO/IDO1/1.Tif]

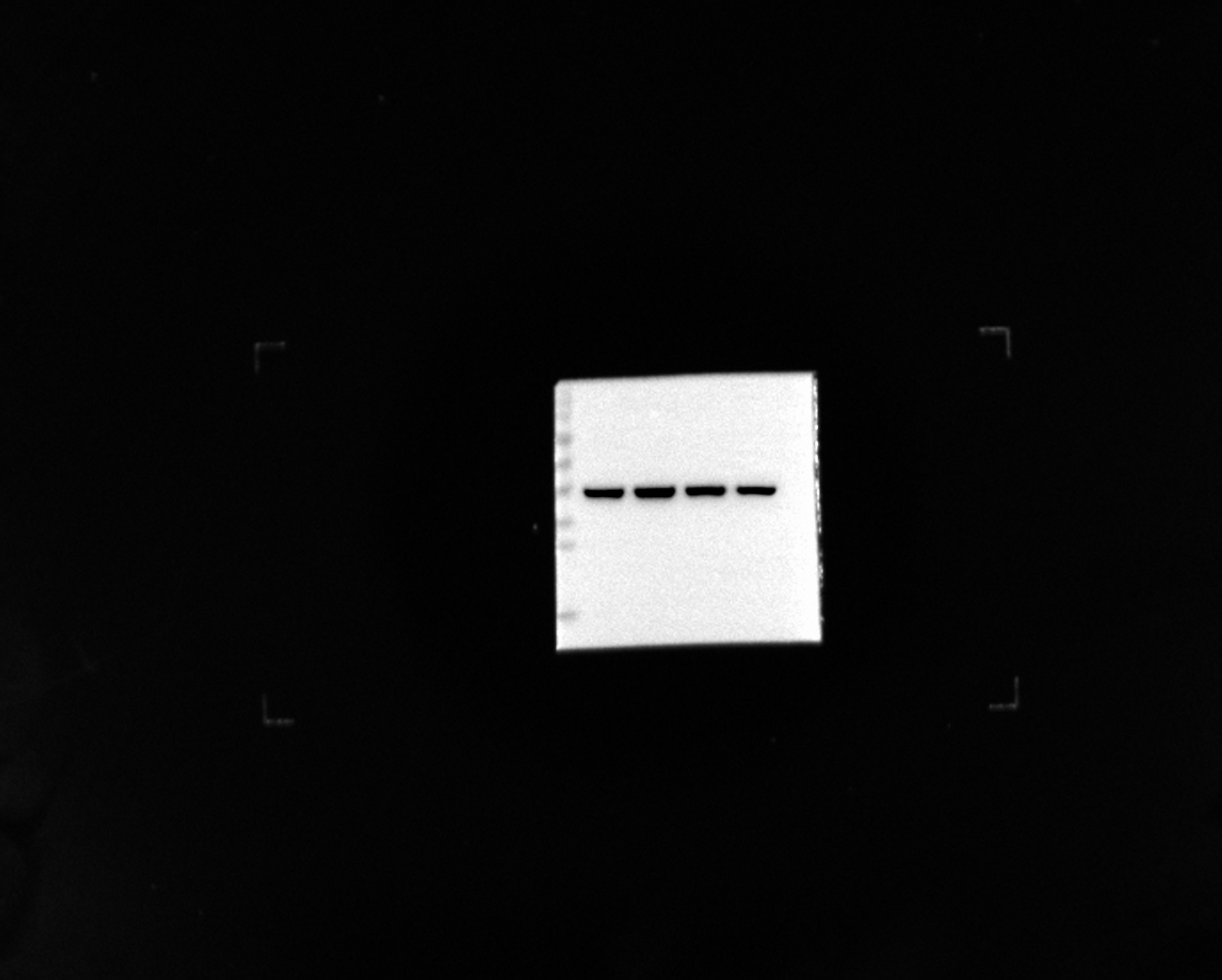

Supplement: Supplementary file 2 [file DataSheet1.zip › IDO/IDO1/1副本.tif]

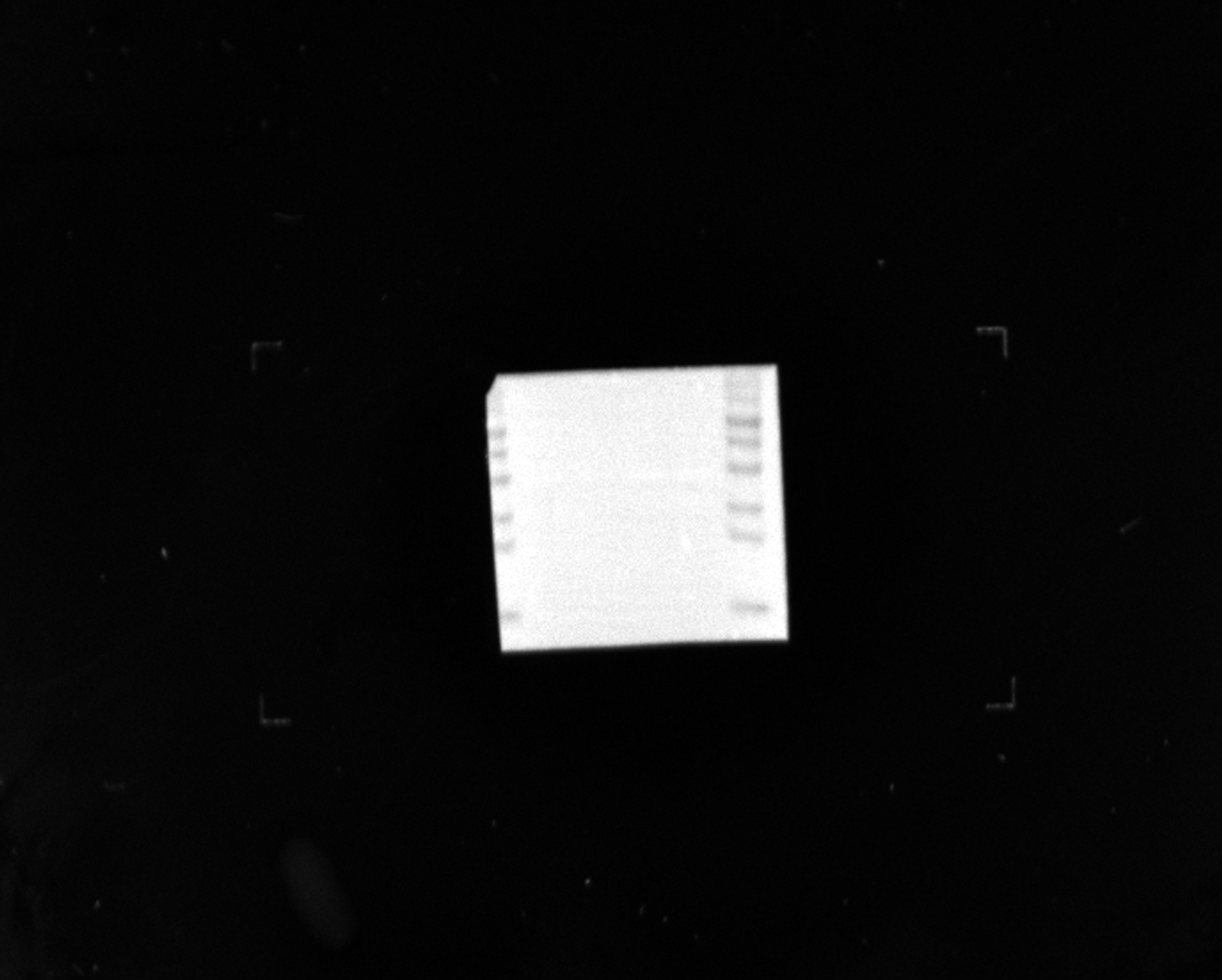

Supplement: Supplementary file 2 [file DataSheet1.zip › IDO/IDO1/2-t.Tif]

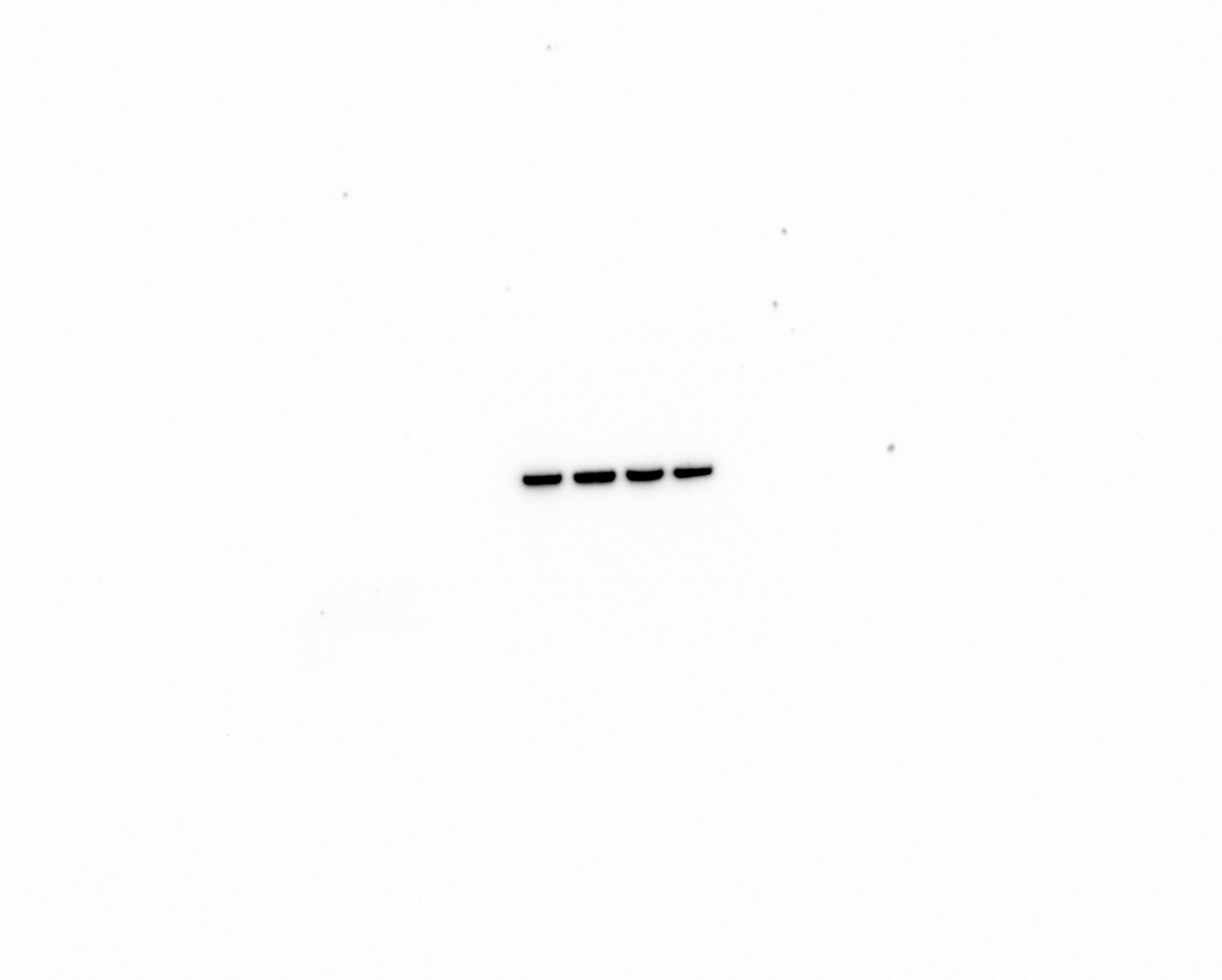

Supplement: Supplementary file 2 [file DataSheet1.zip › IDO/IDO1/2.Tif]

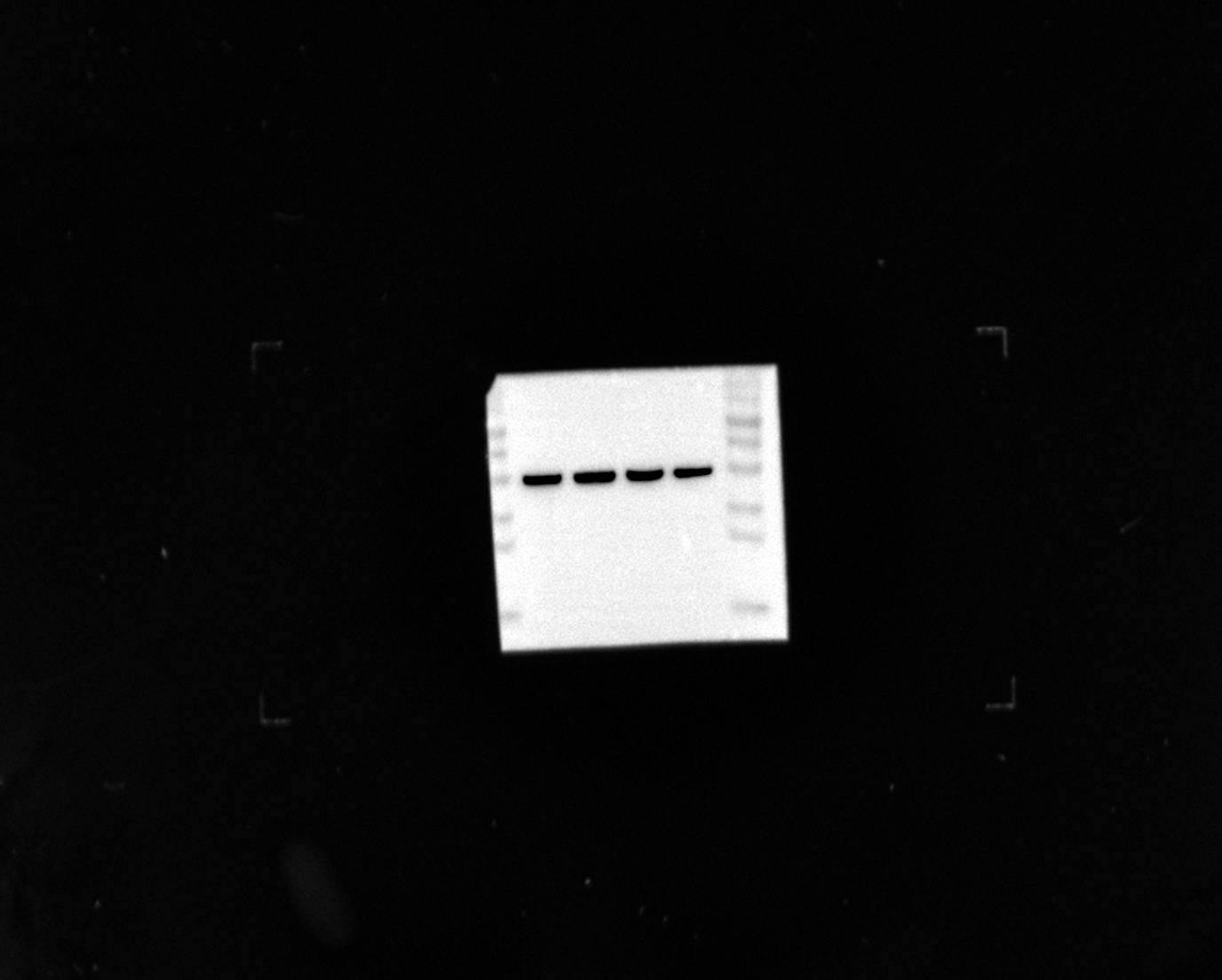

Supplement: Supplementary file 2 [file DataSheet1.zip › IDO/IDO1/2副本.tif]

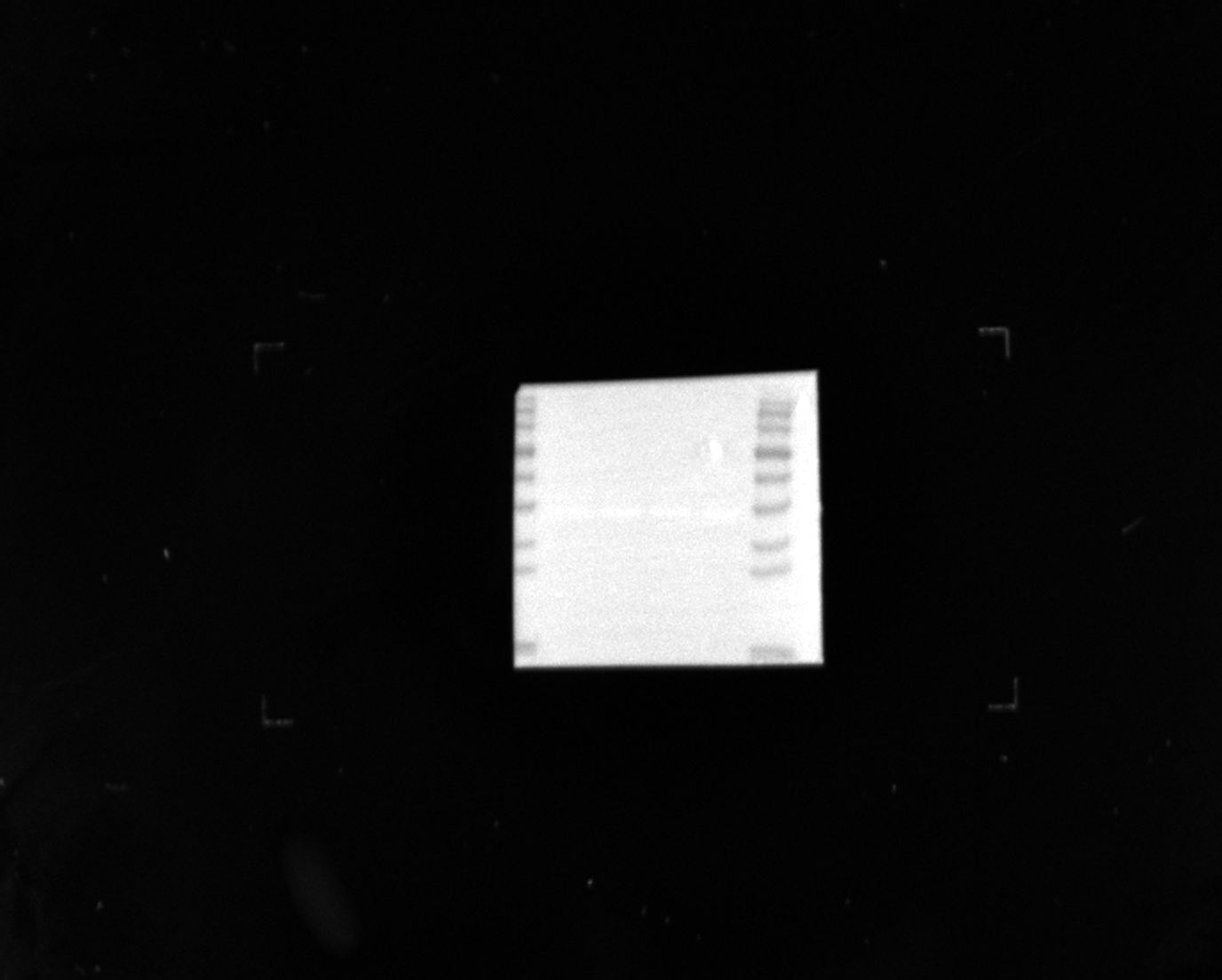

Supplement: Supplementary file 2 [file DataSheet1.zip › IDO/IDO1/3-t.Tif]

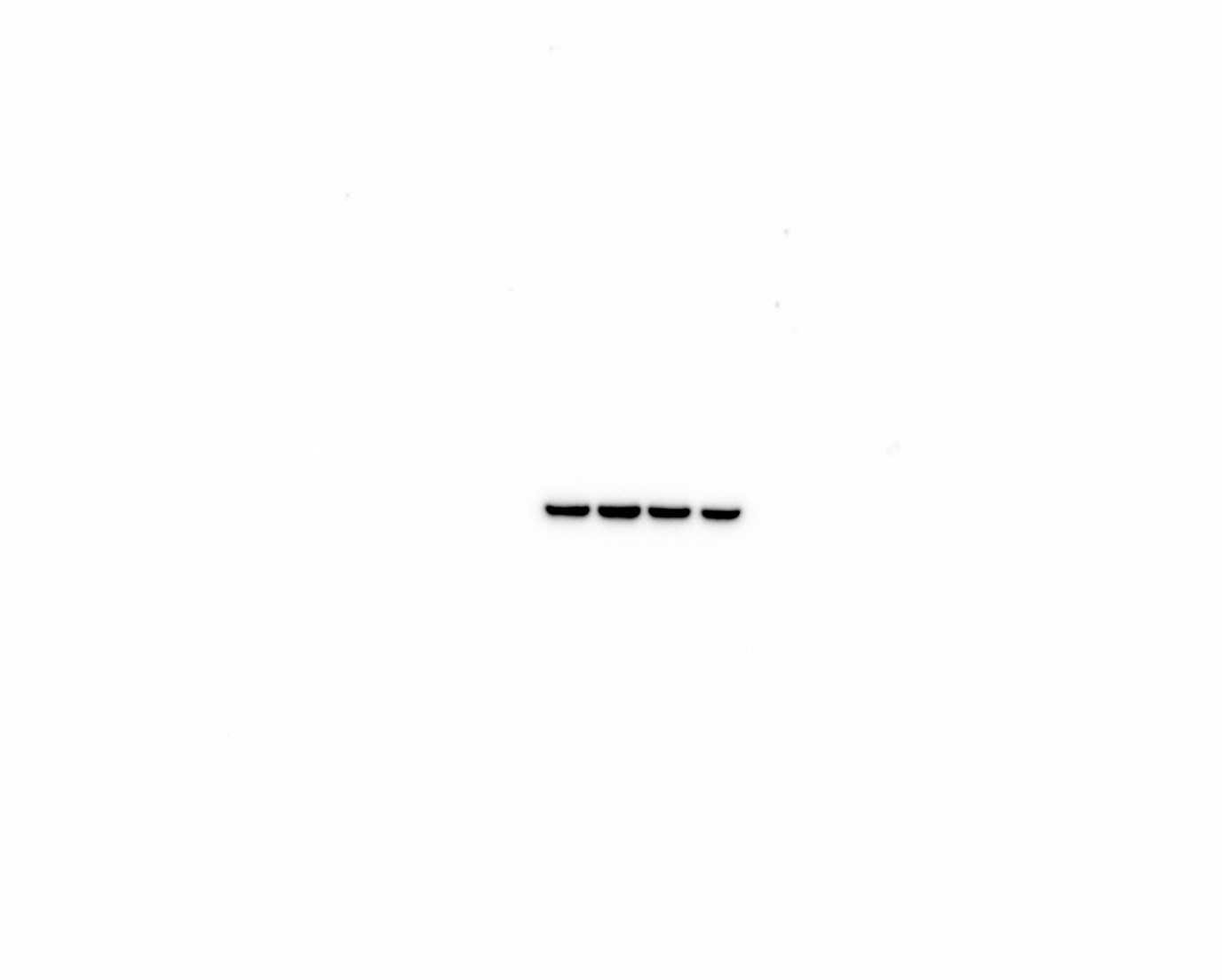

Supplement: Supplementary file 2 [file DataSheet1.zip › IDO/IDO1/3.Tif]

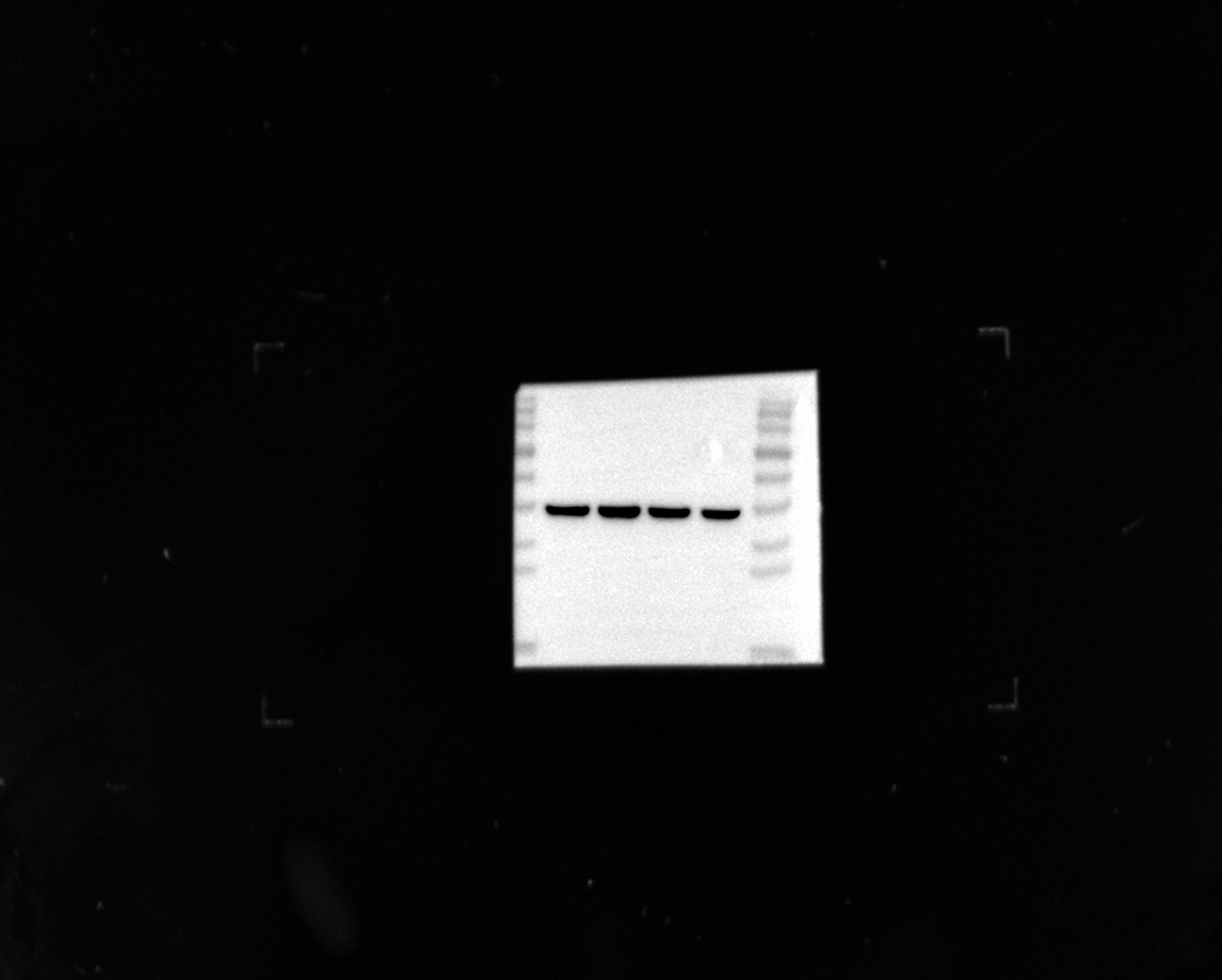

Supplement: Supplementary file 2 [file DataSheet1.zip › IDO/IDO1/3副本.tif]

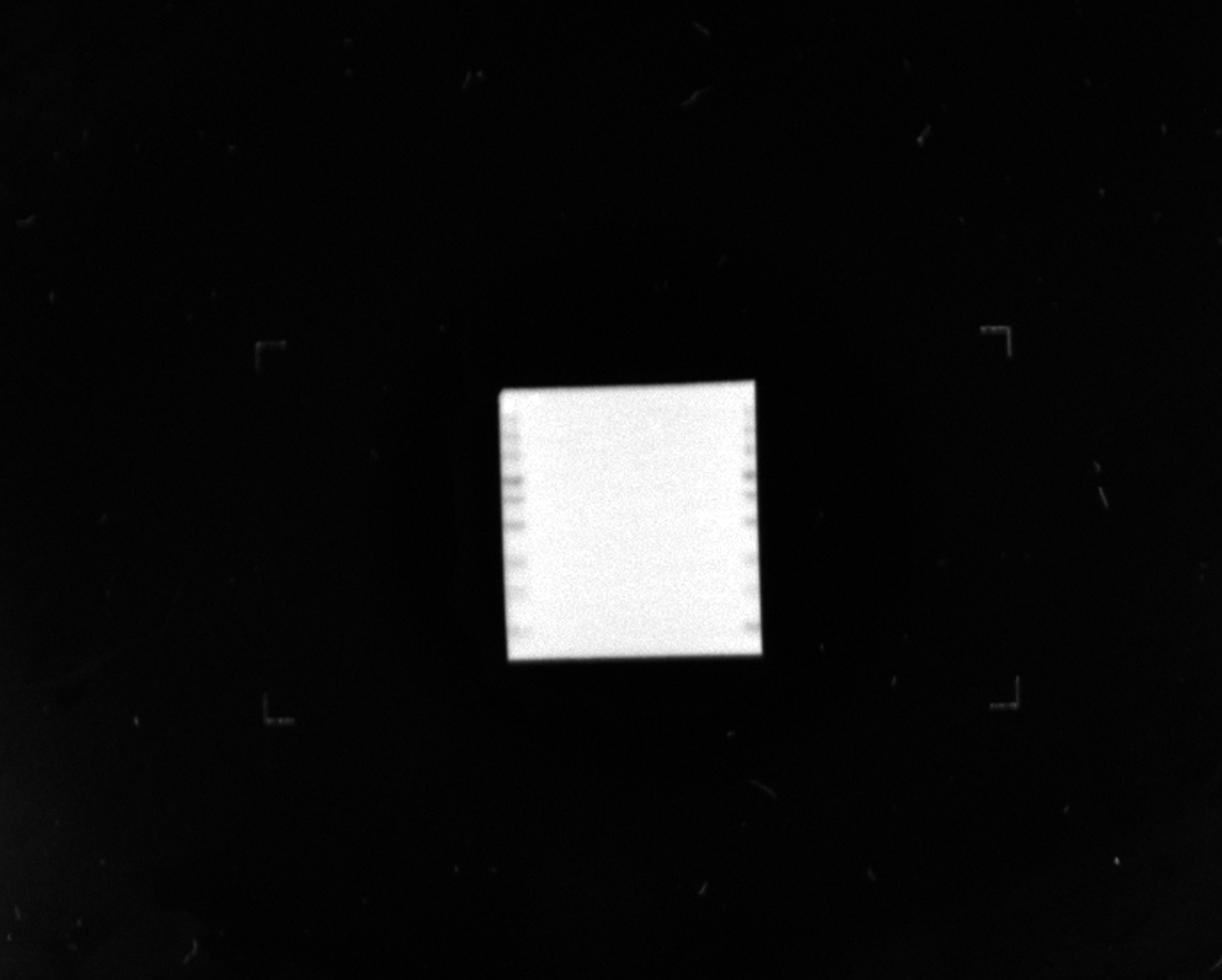

Supplement: Supplementary file 2 [file DataSheet1.zip › IDO/β-actin/1-t.Tif]

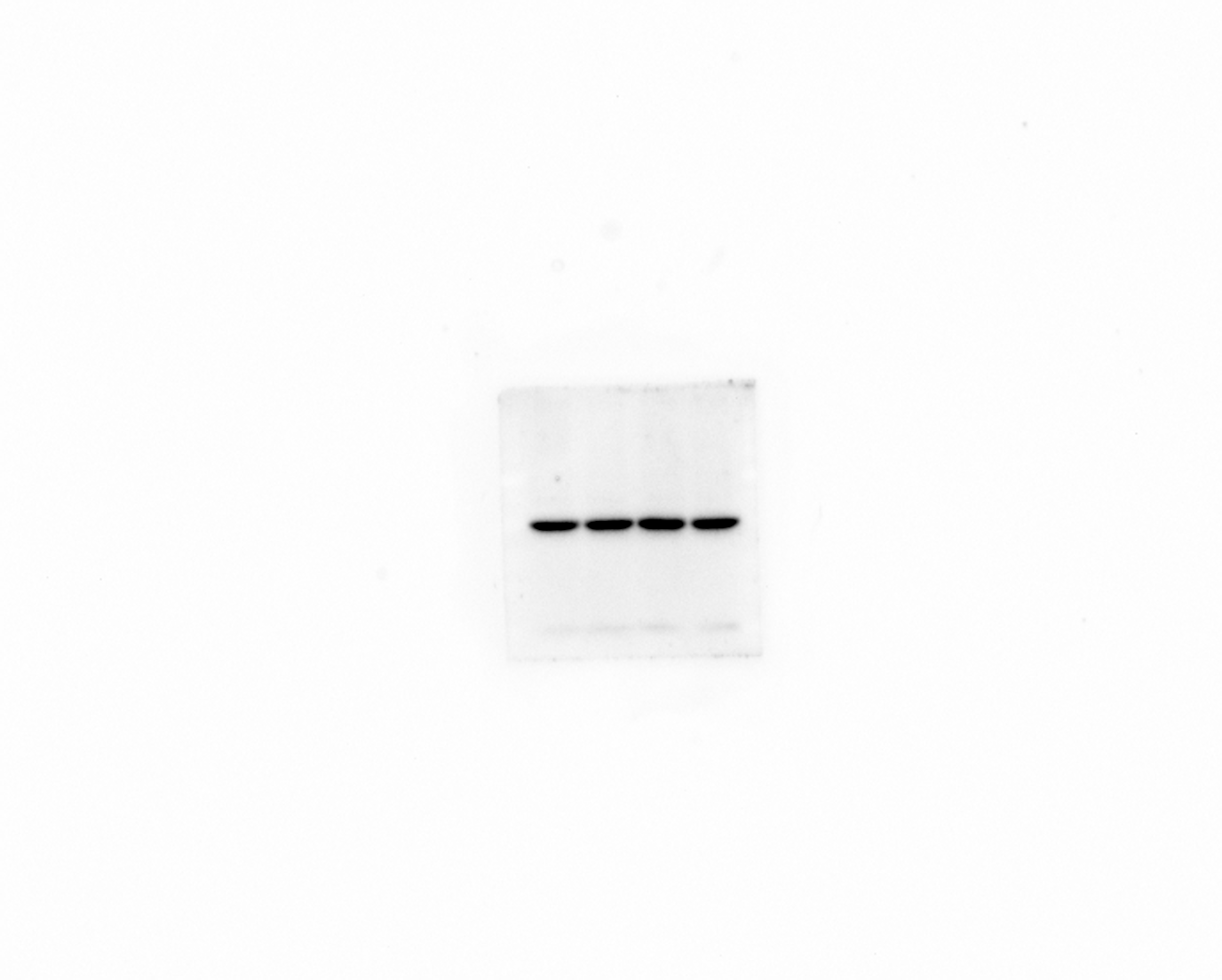

Supplement: Supplementary file 2 [file DataSheet1.zip › IDO/β-actin/1.Tif]

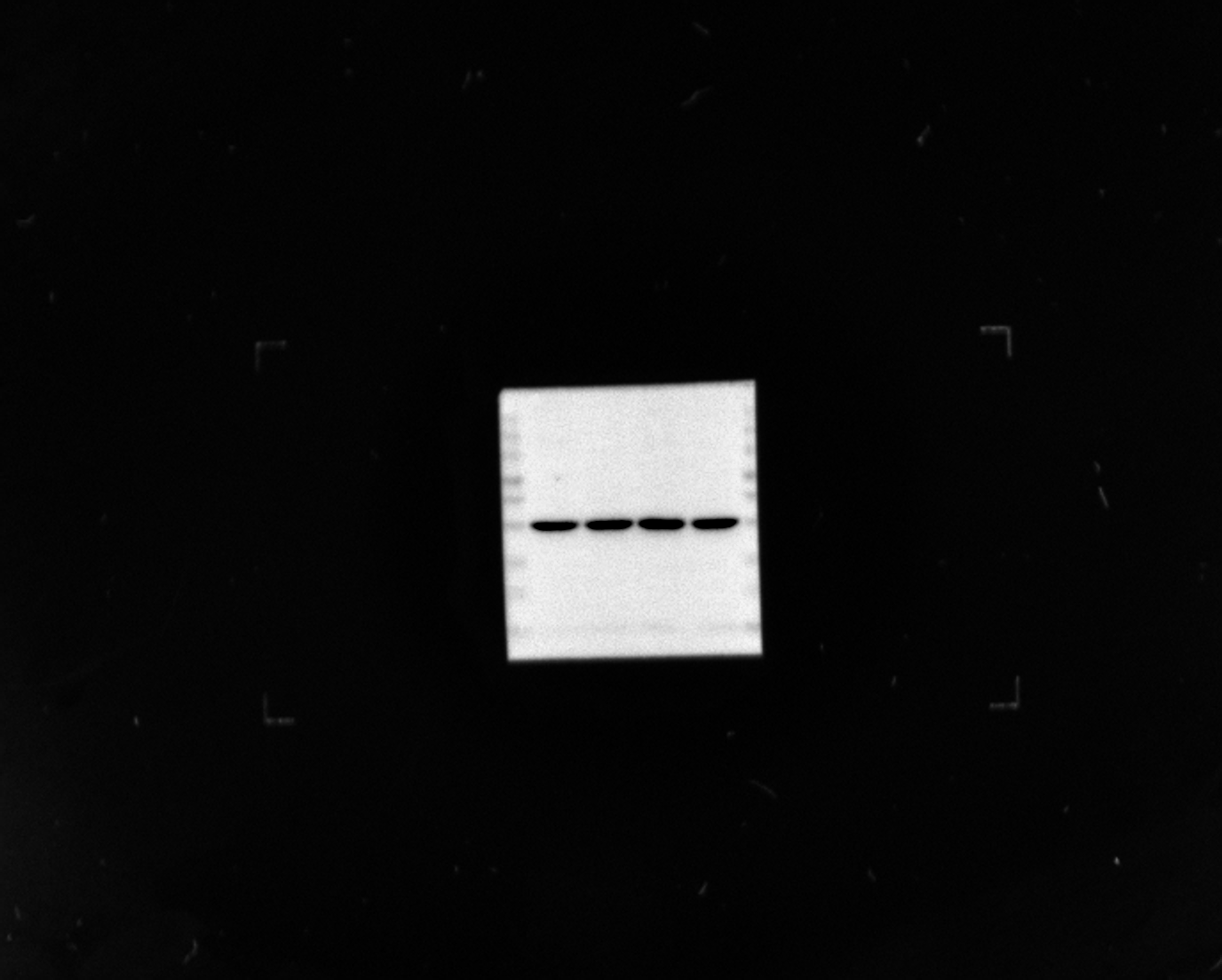

Supplement: Supplementary file 2 [file DataSheet1.zip › IDO/β-actin/1副本.tif]

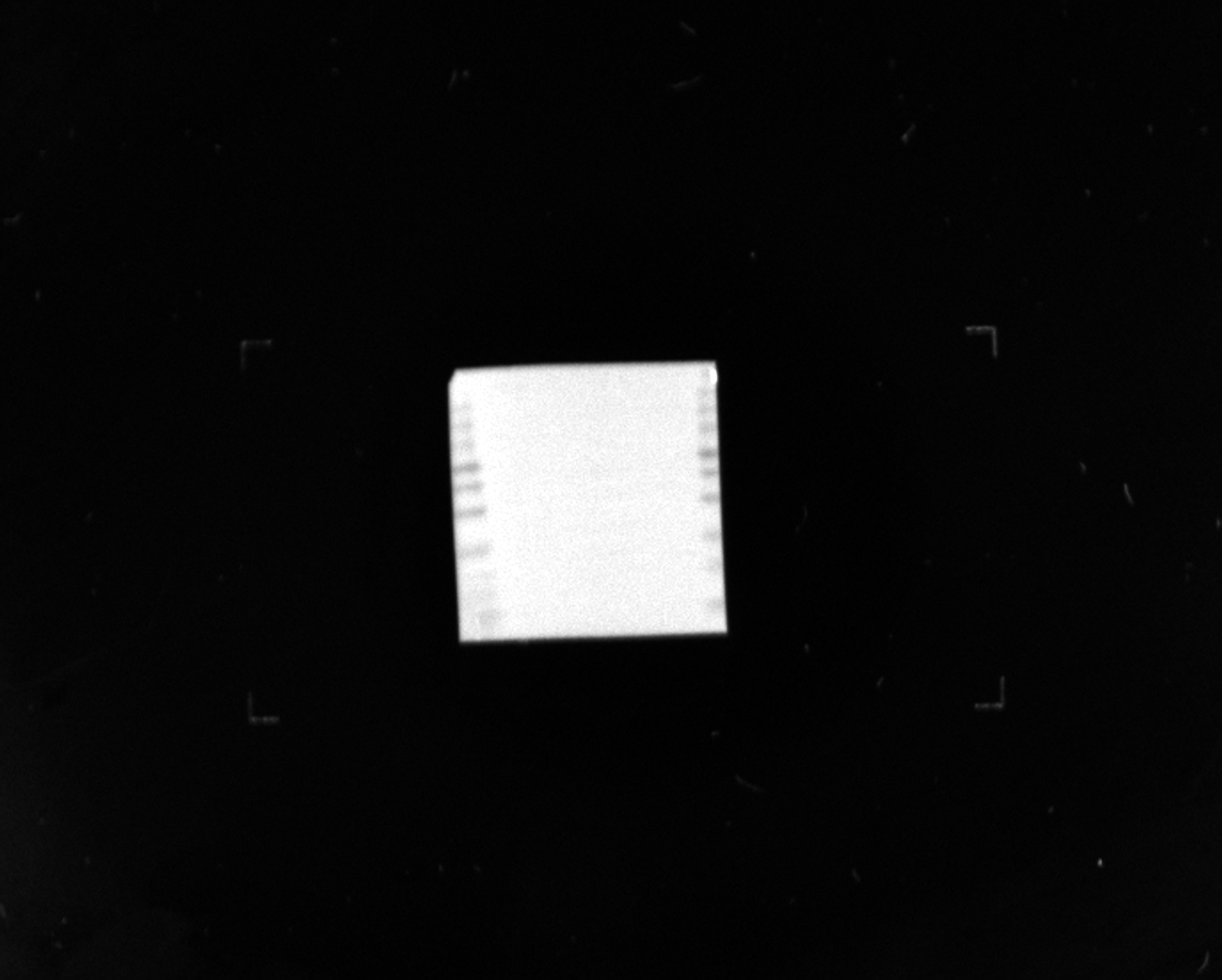

Supplement: Supplementary file 2 [file DataSheet1.zip › IDO/β-actin/2-t.Tif]

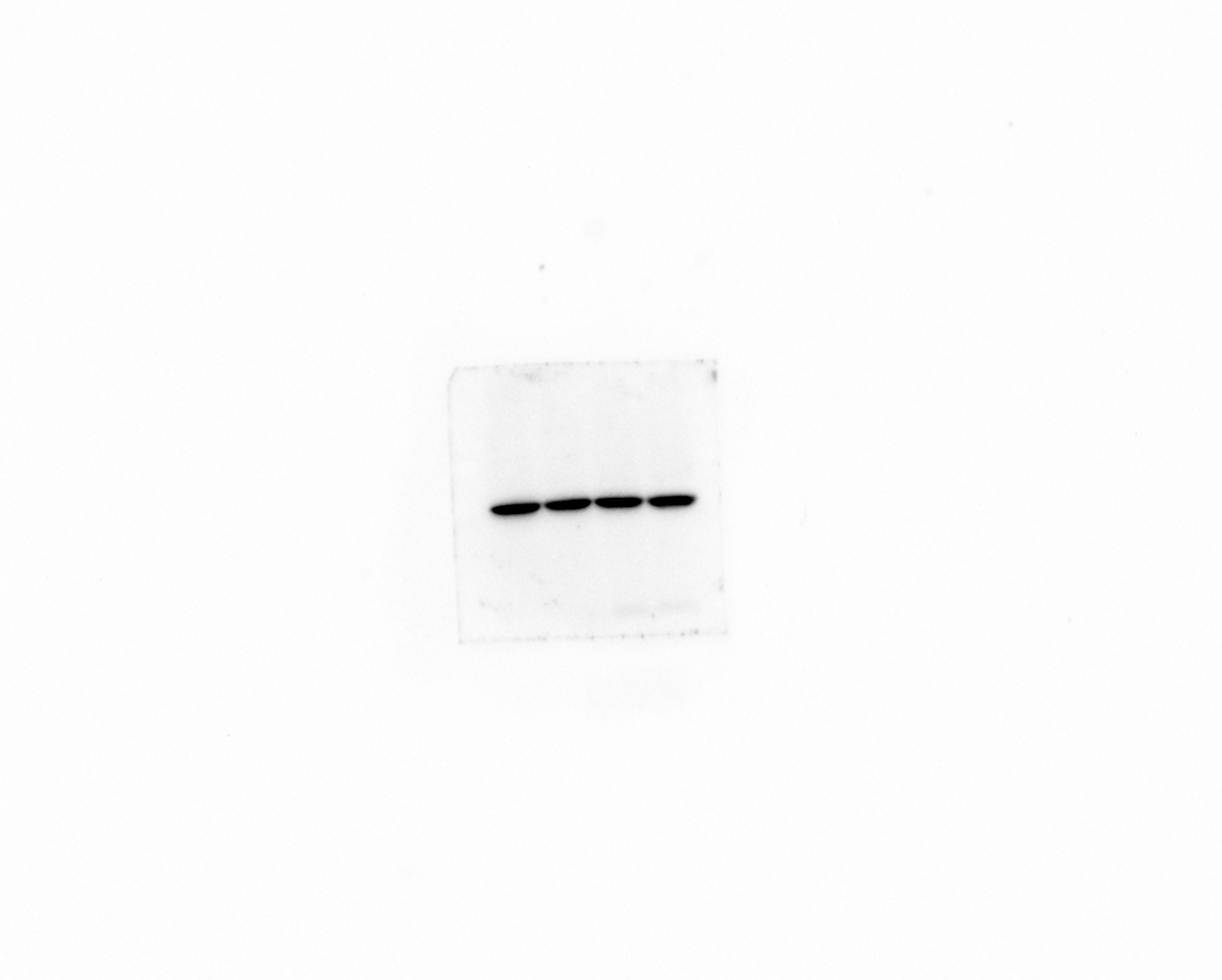

Supplement: Supplementary file 2 [file DataSheet1.zip › IDO/β-actin/2.Tif]

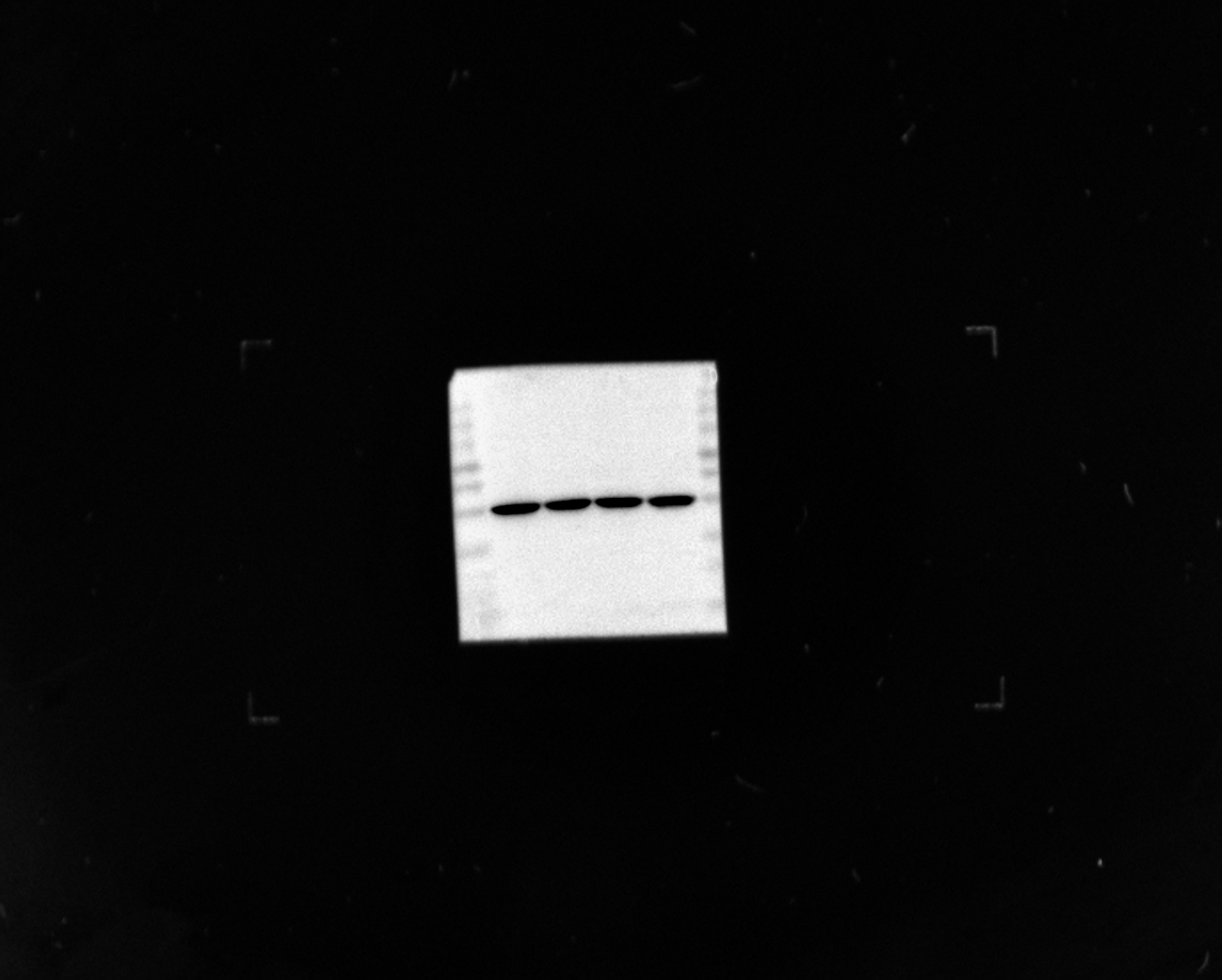

Supplement: Supplementary file 2 [file DataSheet1.zip › IDO/β-actin/2副本.tif]

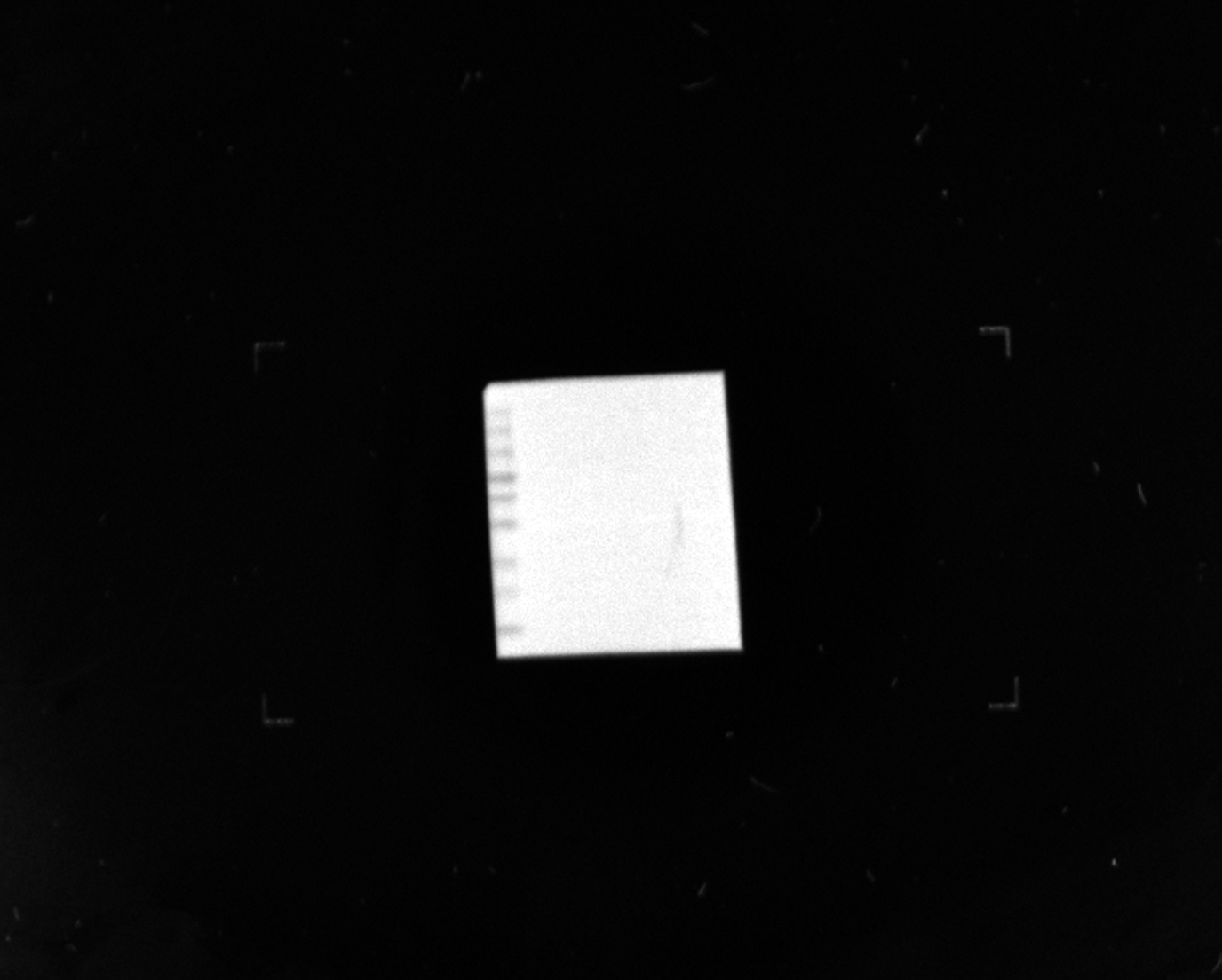

Supplement: Supplementary file 2 [file DataSheet1.zip › IDO/β-actin/3-t.Tif]

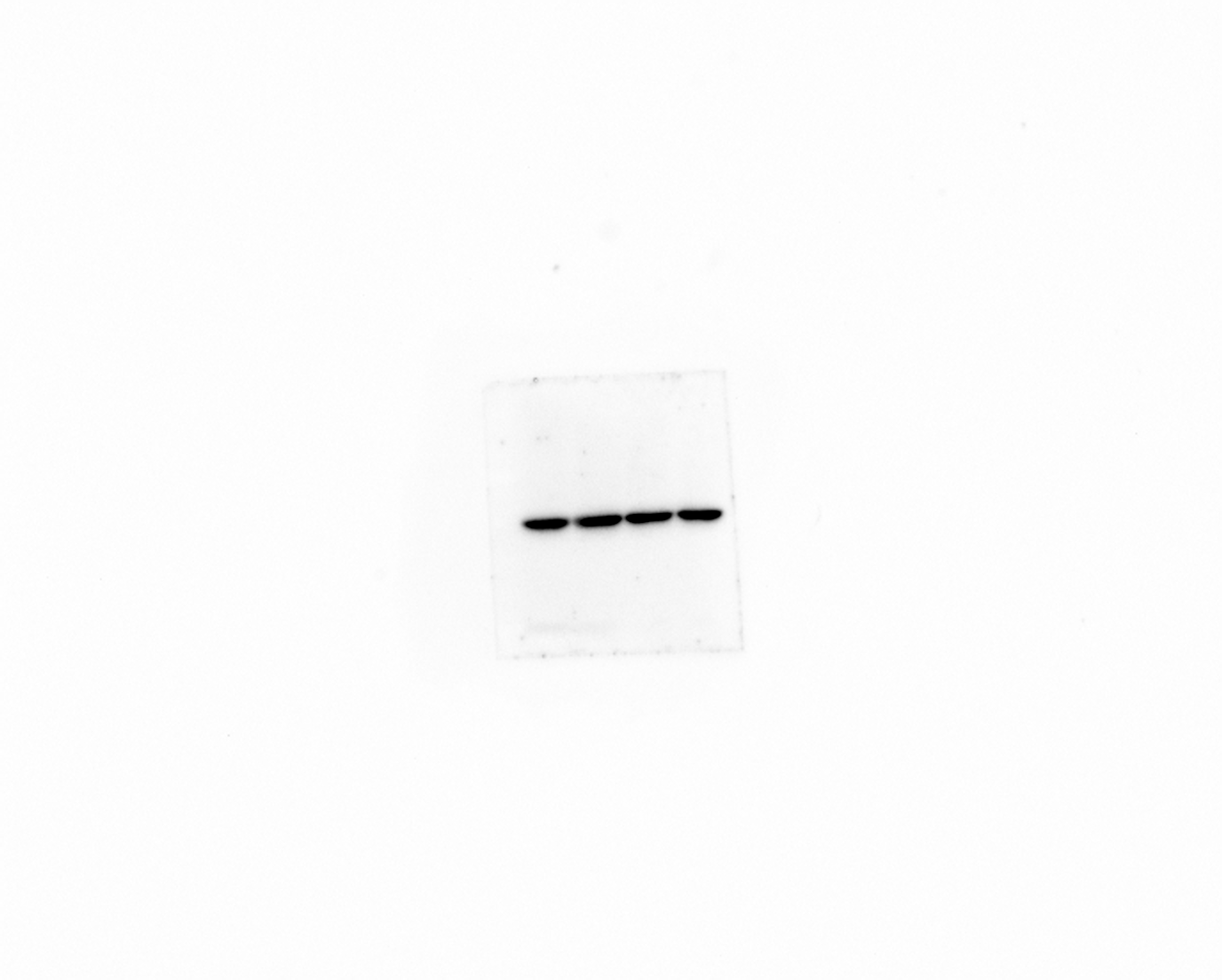

Supplement: Supplementary file 2 [file DataSheet1.zip › IDO/β-actin/3.Tif]

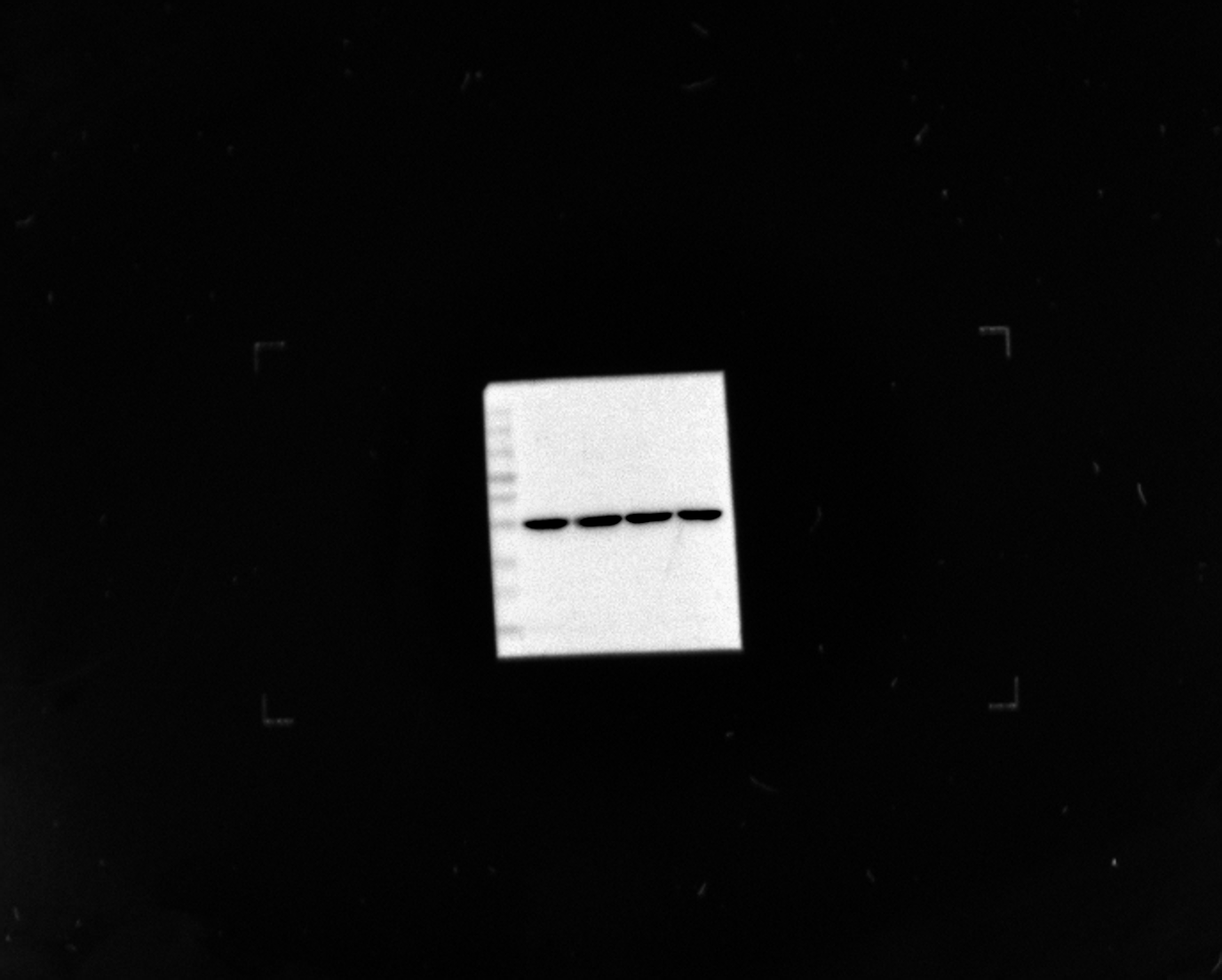

Supplement: Supplementary file 2 [file DataSheet1.zip › IDO/β-actin/3副本.tif]

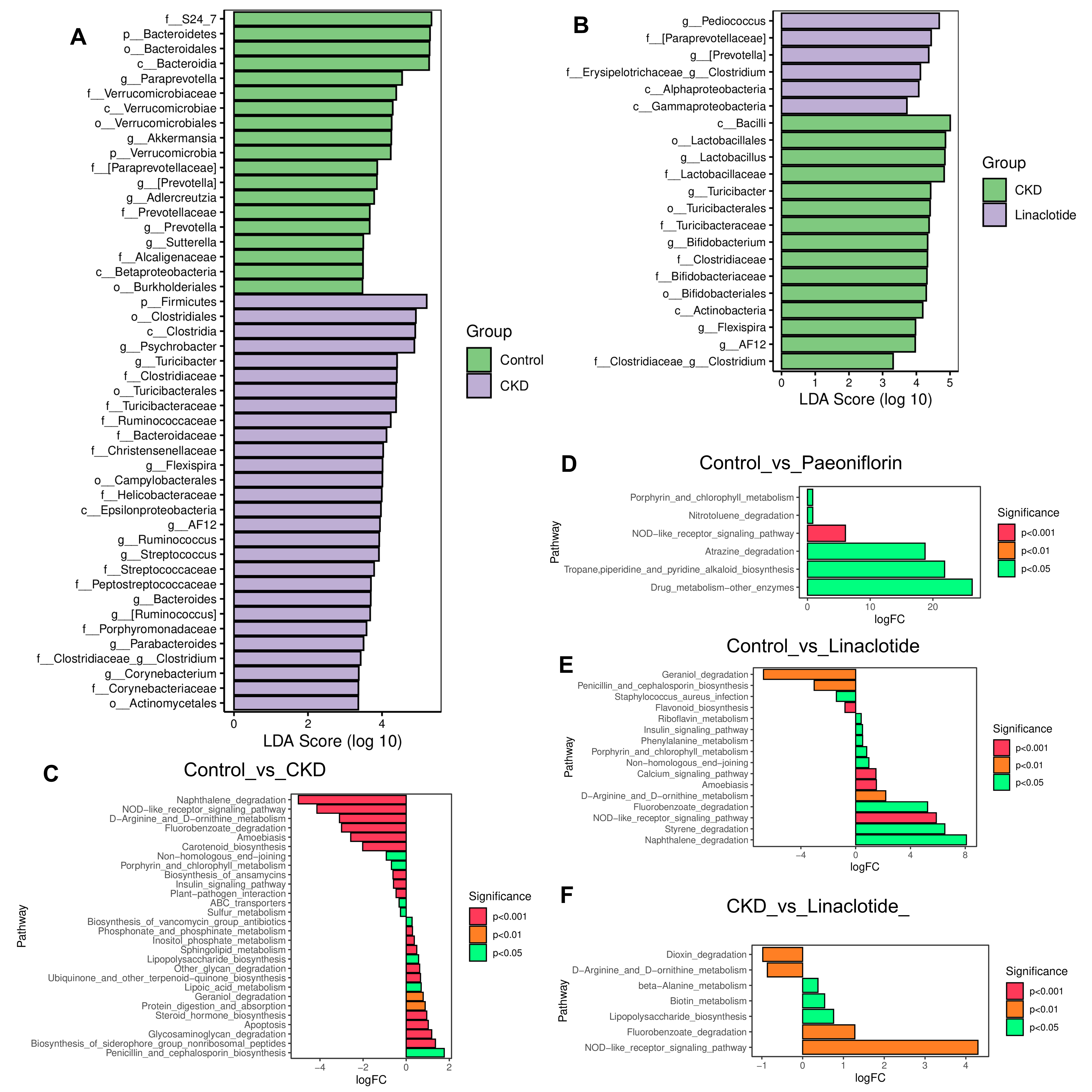

Supplement: Supplementary file 3 [file Image1.tif]

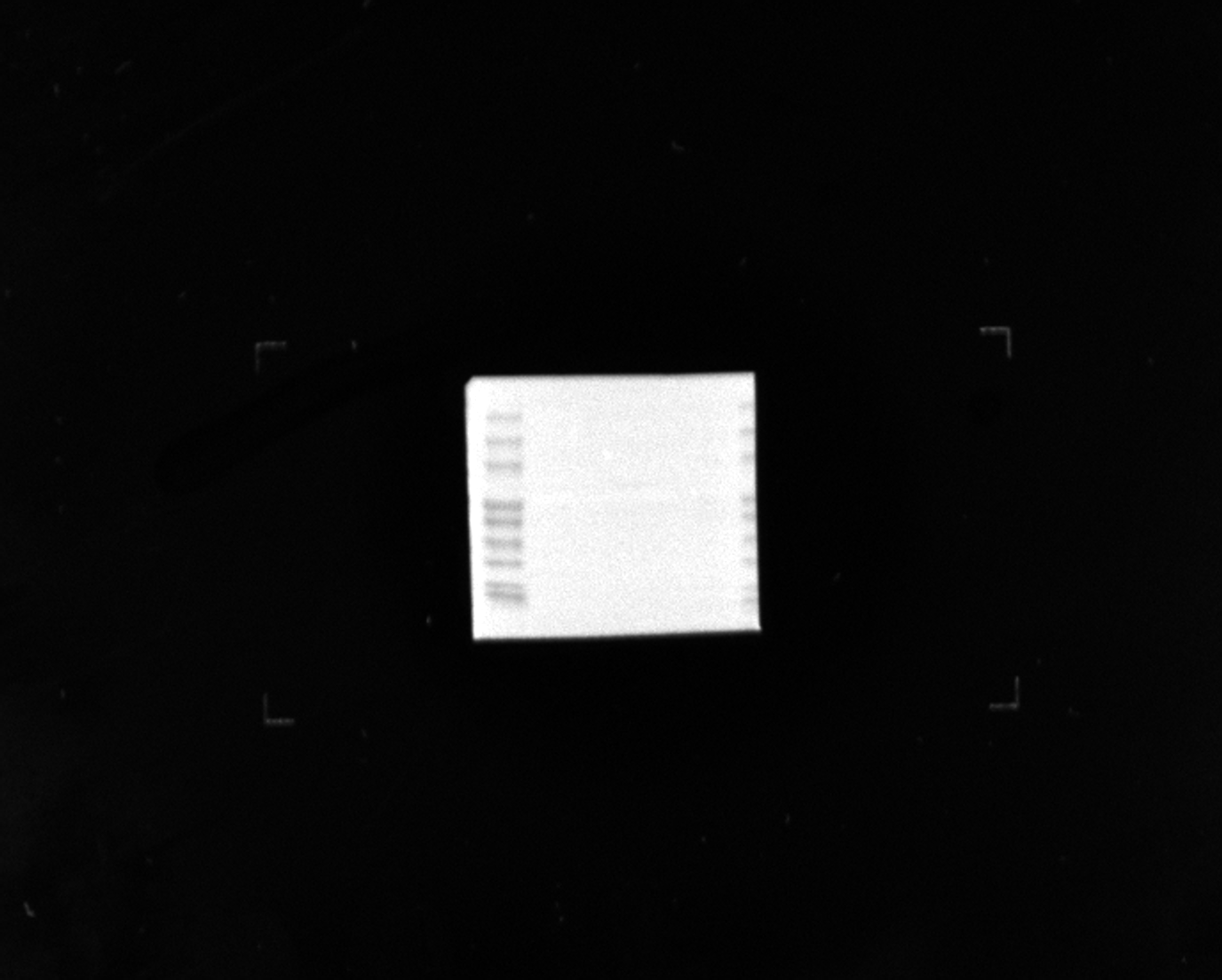

Supplement: Supplementary file 4 [file DataSheet2.zip › 原图2/AHR/1-t.Tif]

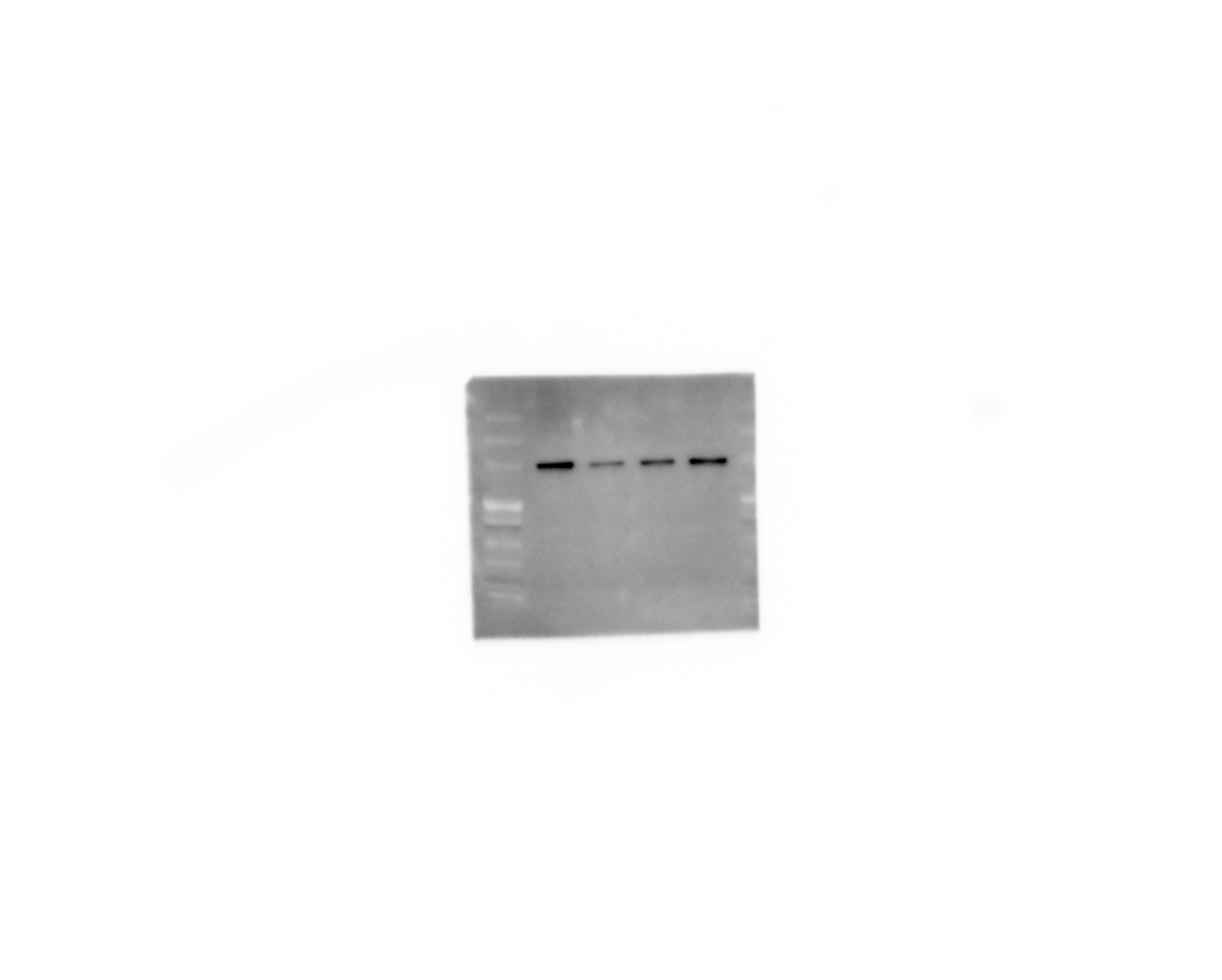

Supplement: Supplementary file 4 [file DataSheet2.zip › 原图2/AHR/1.Tif]

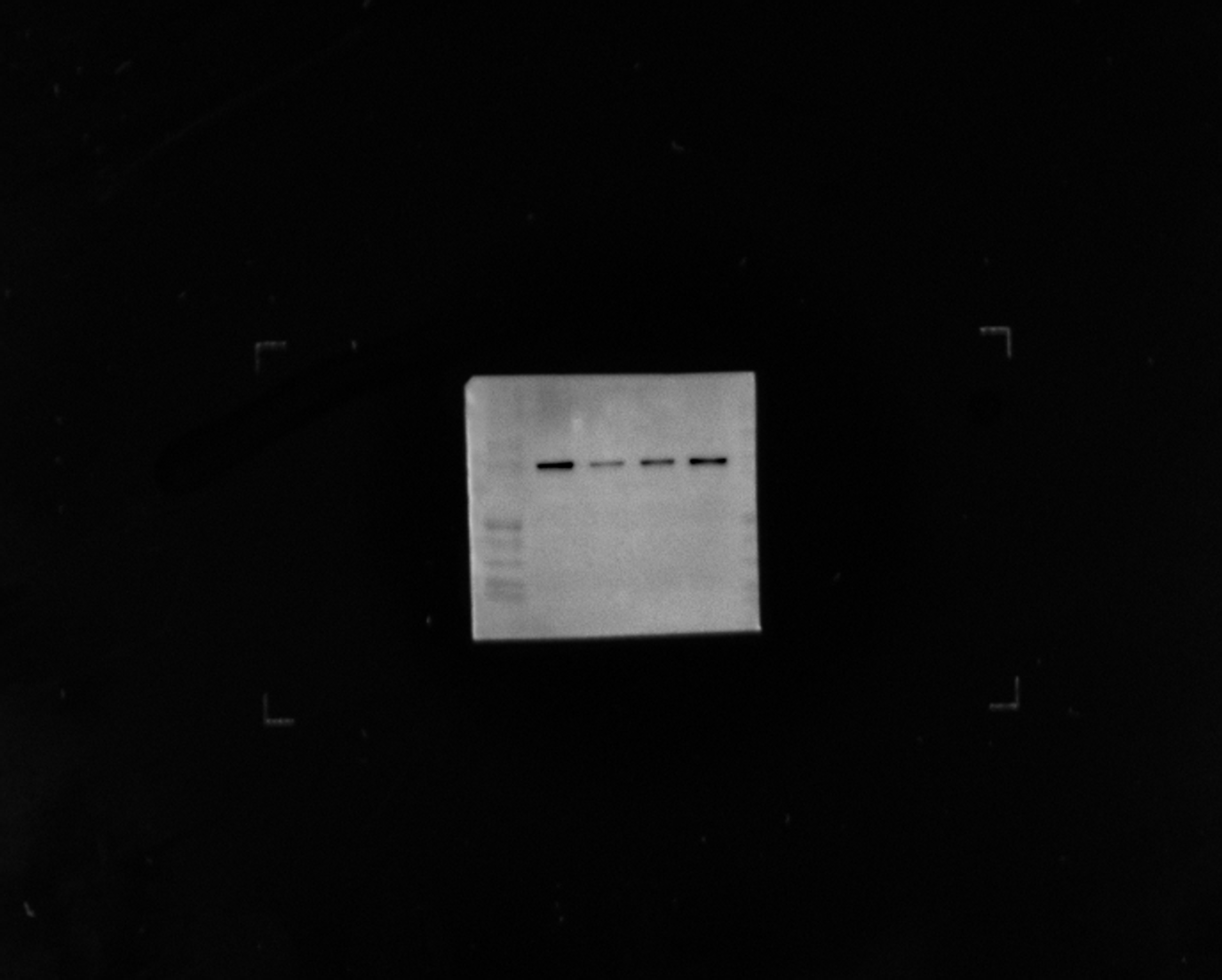

Supplement: Supplementary file 4 [file DataSheet2.zip › 原图2/AHR/1副本.tif]

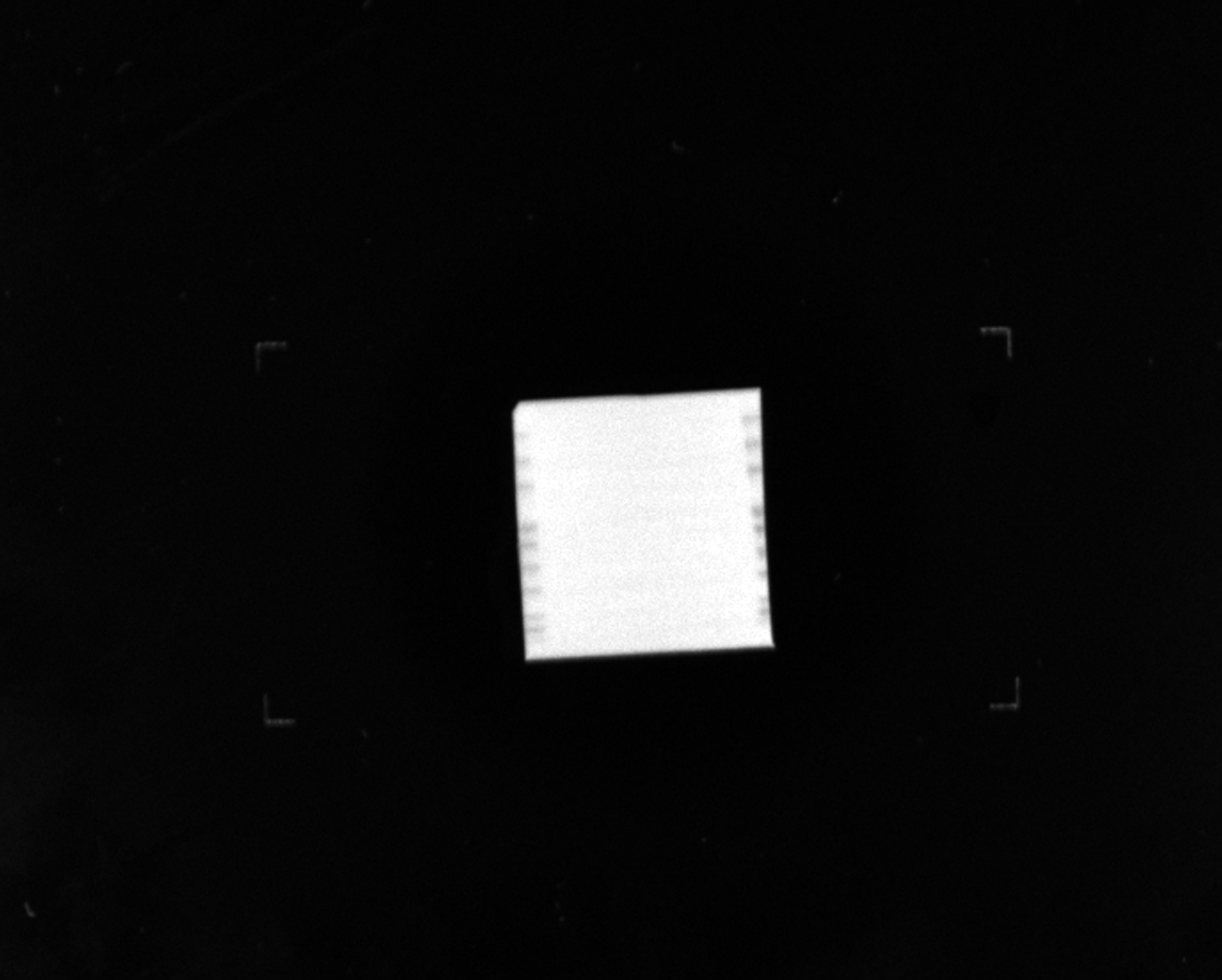

Supplement: Supplementary file 4 [file DataSheet2.zip › 原图2/AHR/2-t.Tif]

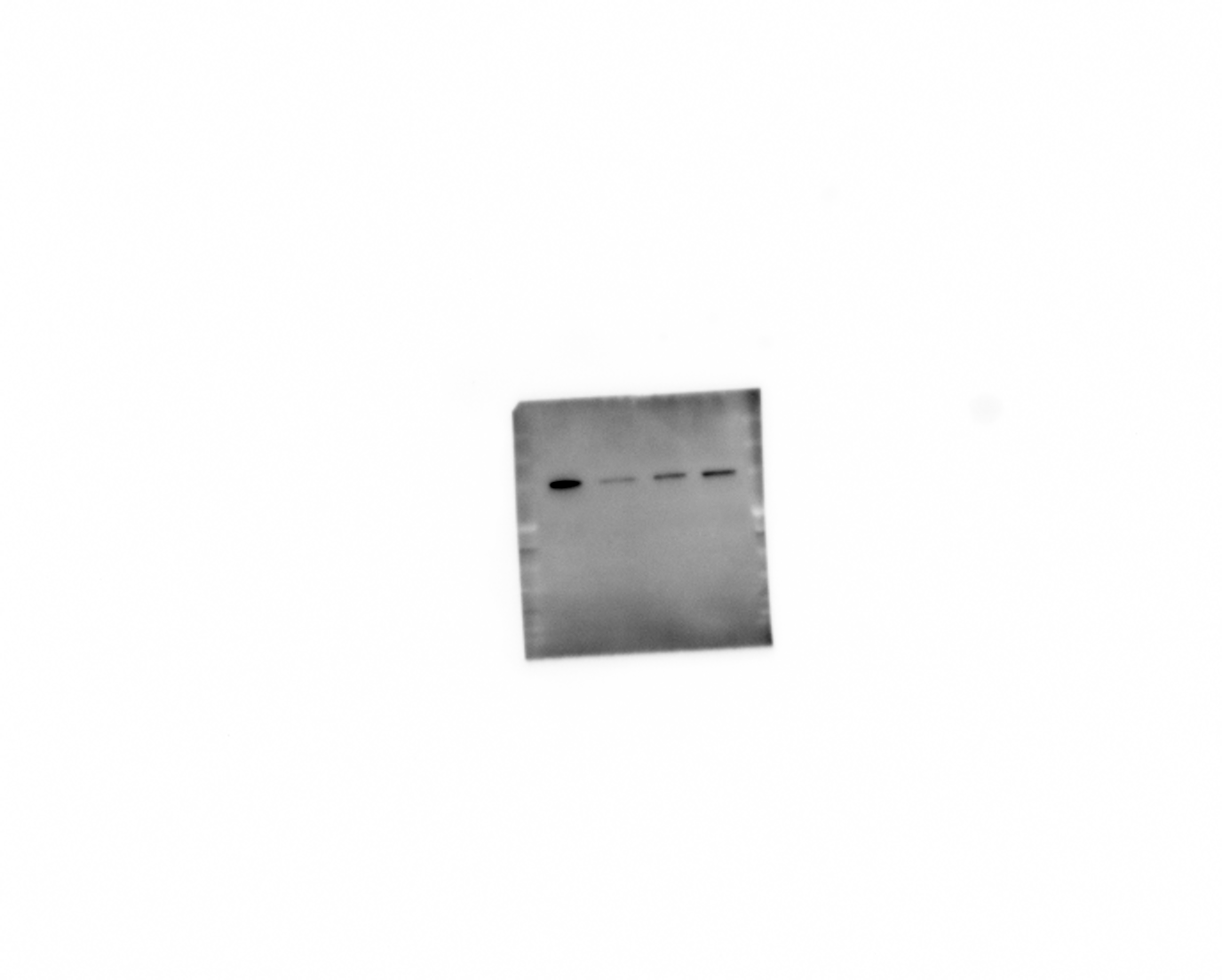

Supplement: Supplementary file 4 [file DataSheet2.zip › 原图2/AHR/2.Tif]

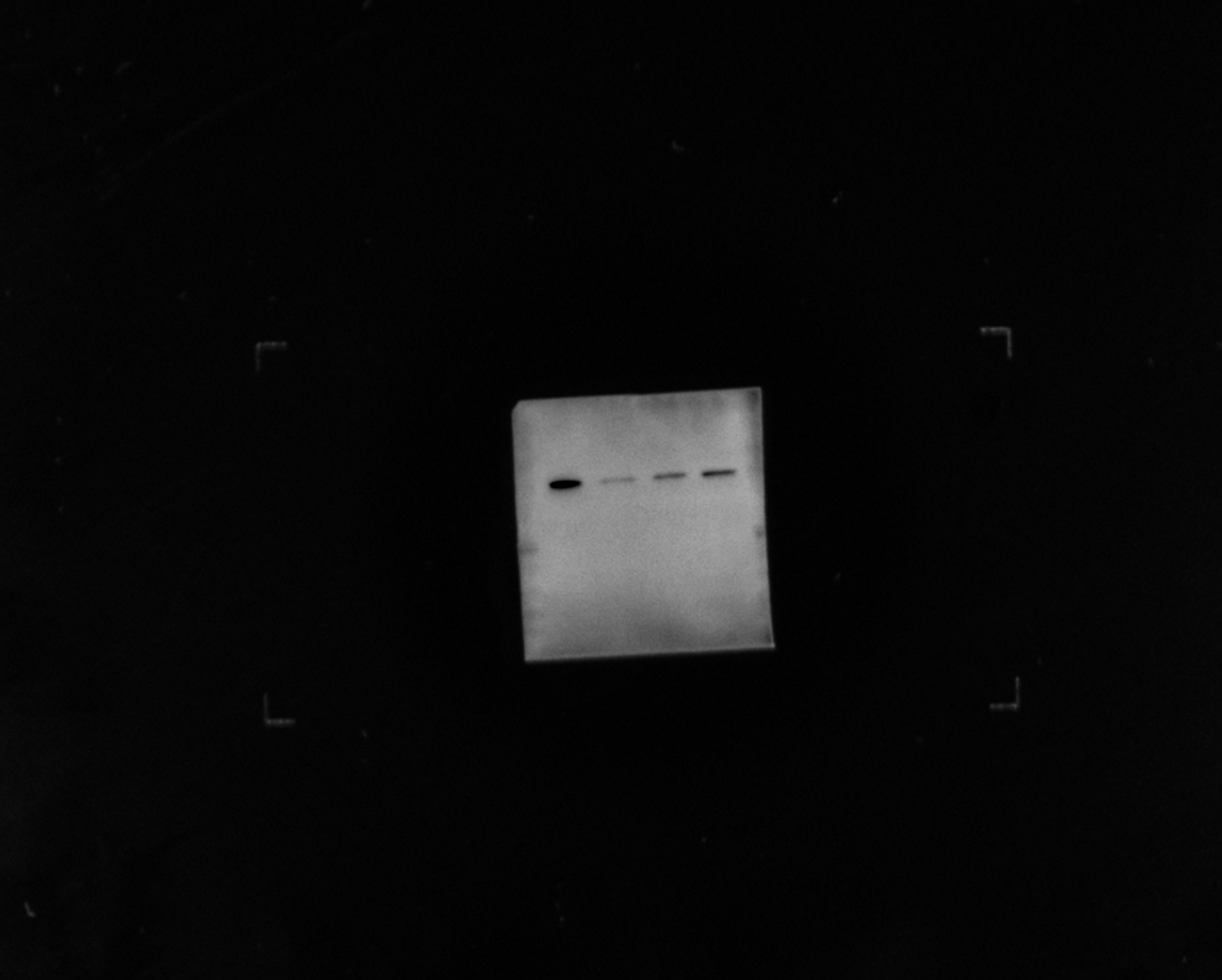

Supplement: Supplementary file 4 [file DataSheet2.zip › 原图2/AHR/2副本.tif]

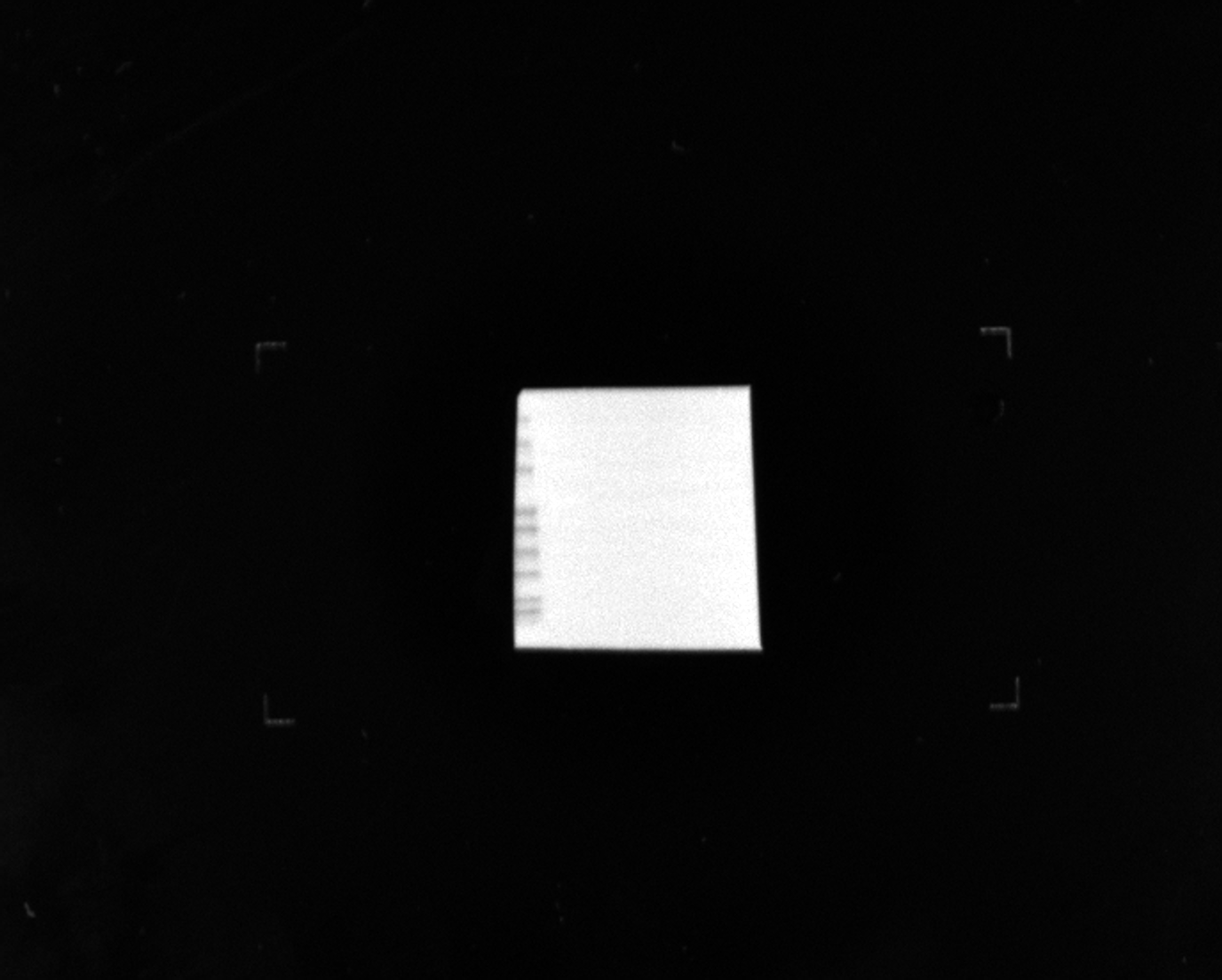

Supplement: Supplementary file 4 [file DataSheet2.zip › 原图2/AHR/3-t.Tif]

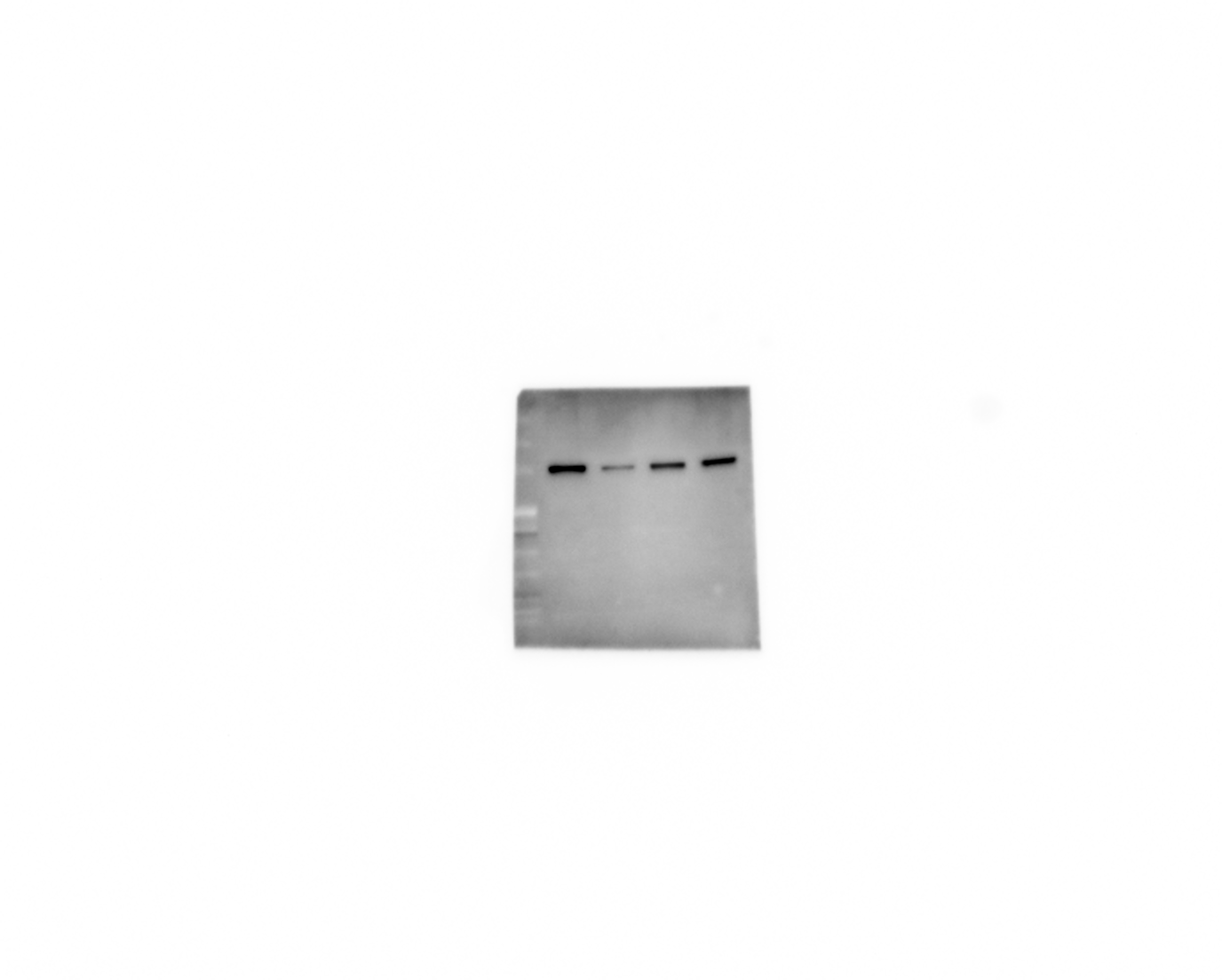

Supplement: Supplementary file 4 [file DataSheet2.zip › 原图2/AHR/3.Tif]

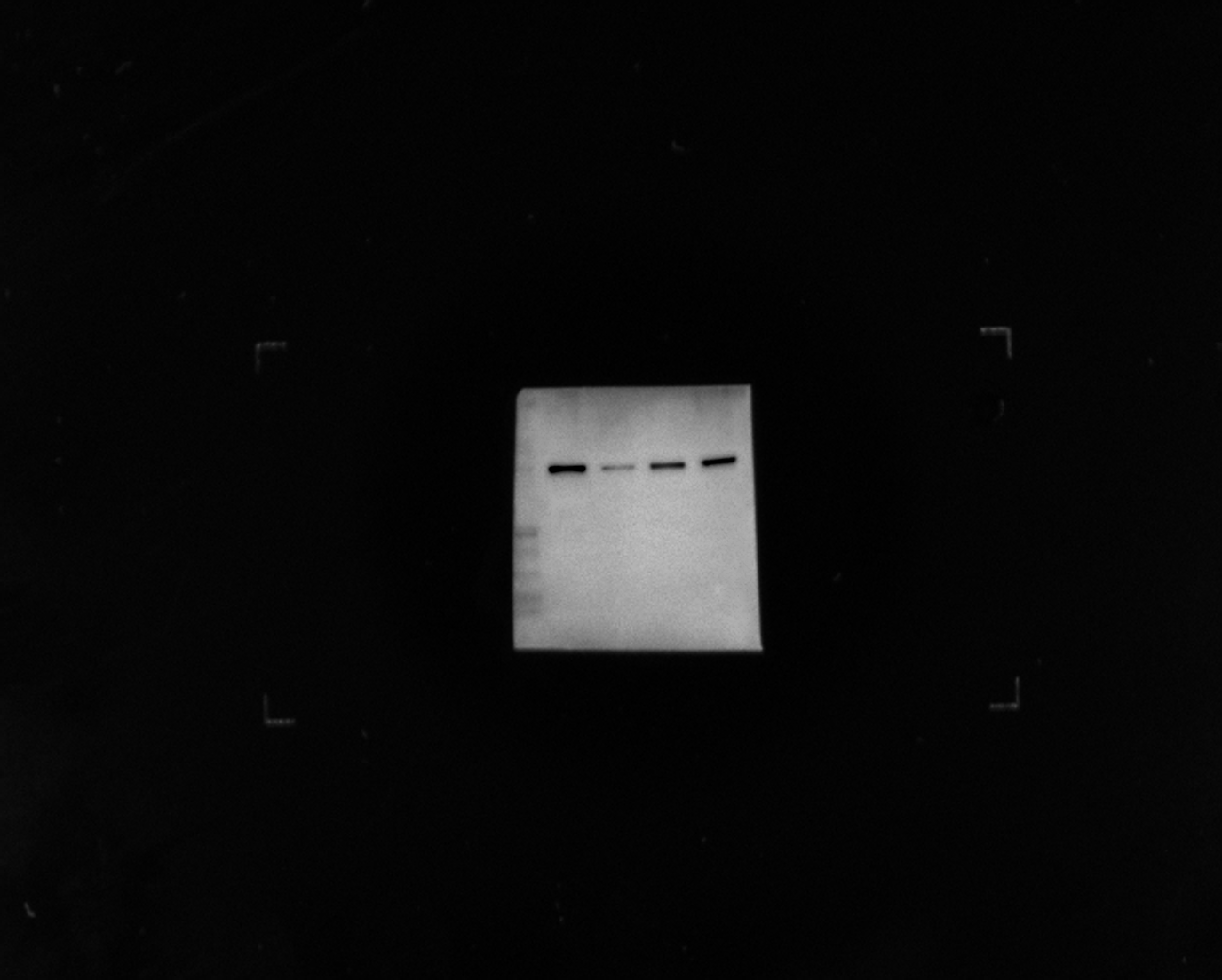

Supplement: Supplementary file 4 [file DataSheet2.zip › 原图2/AHR/3副本.tif]

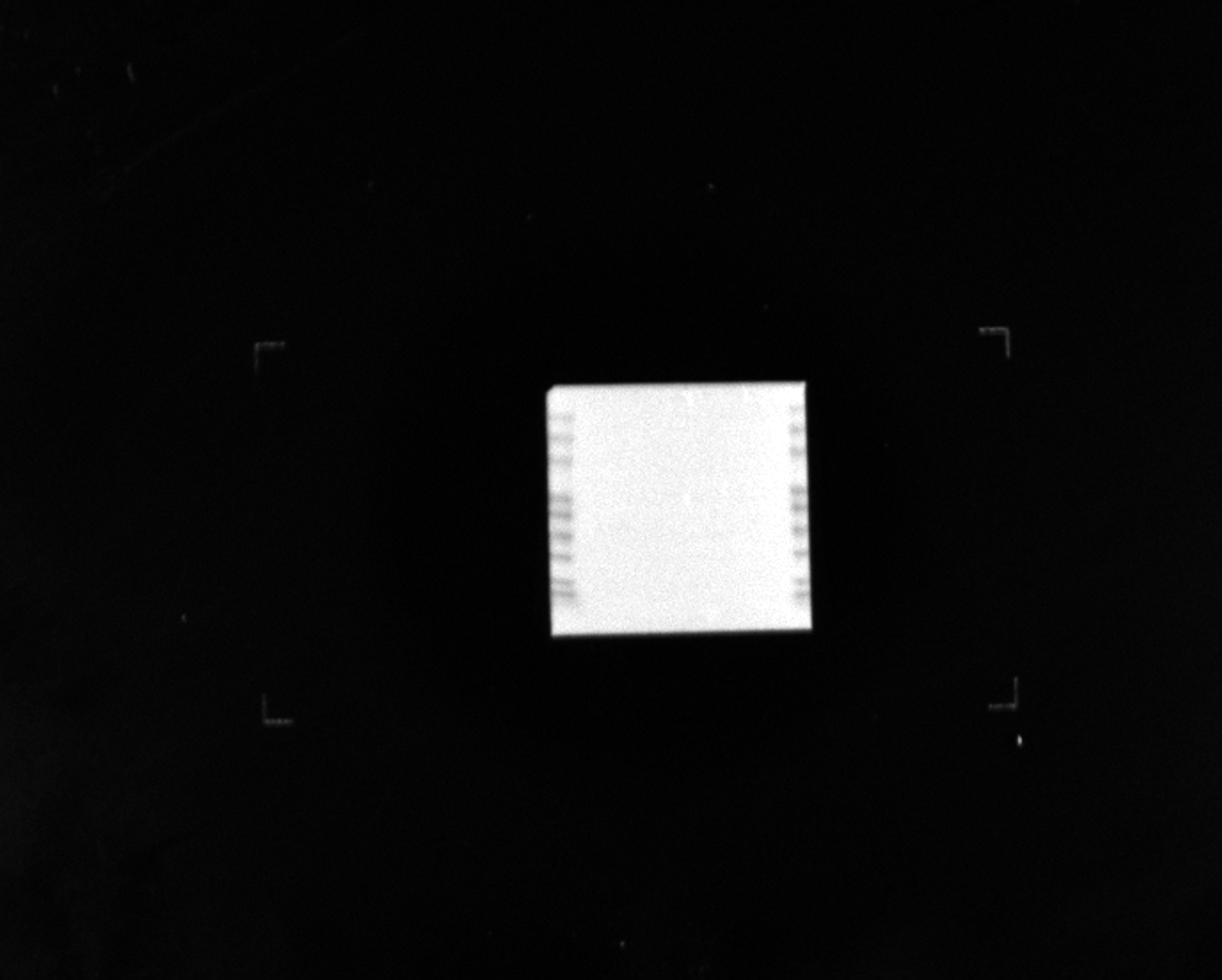

Supplement: Supplementary file 4 [file DataSheet2.zip › 原图2/Occludin/1-t.Tif]

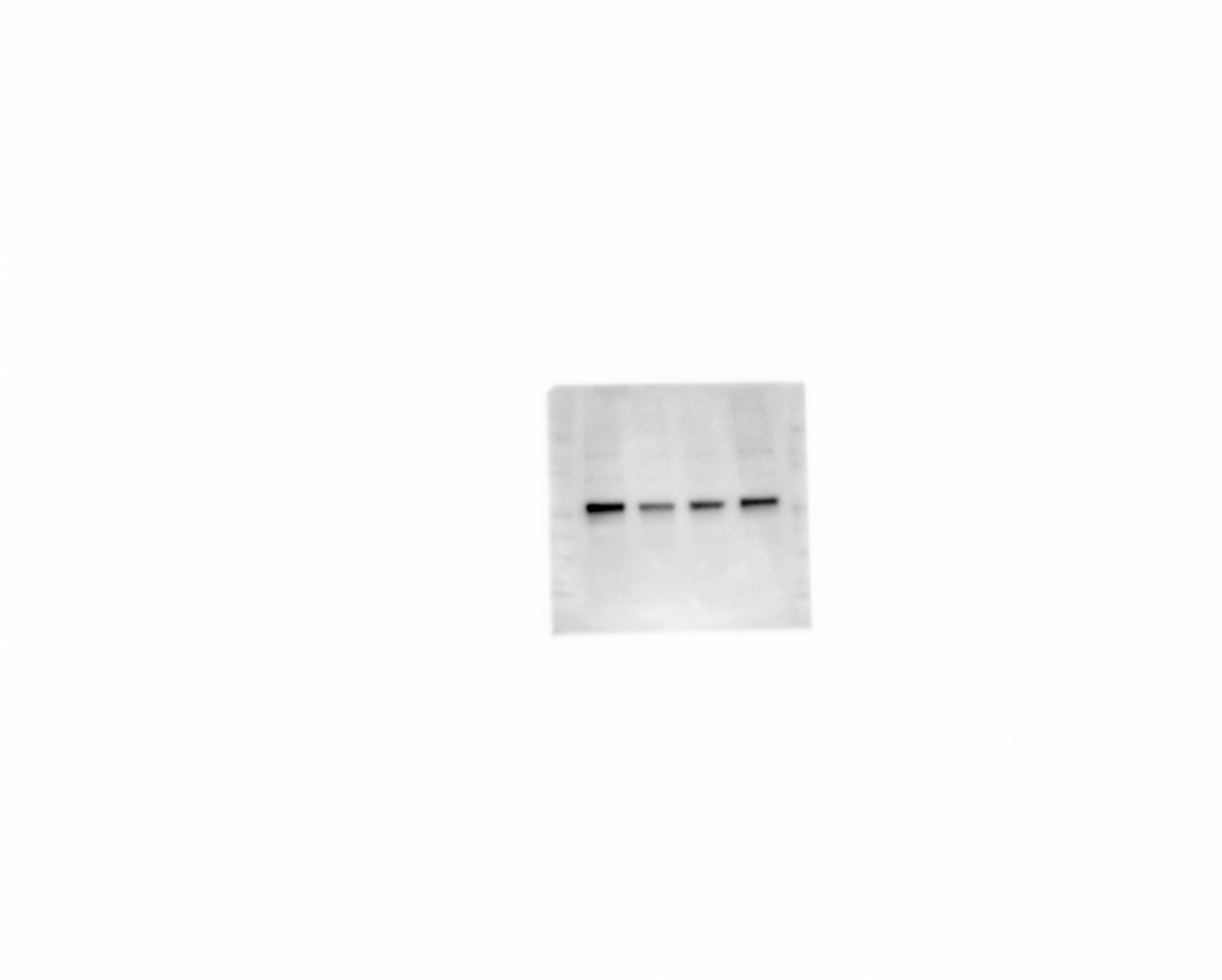

Supplement: Supplementary file 4 [file DataSheet2.zip › 原图2/Occludin/1.Tif]

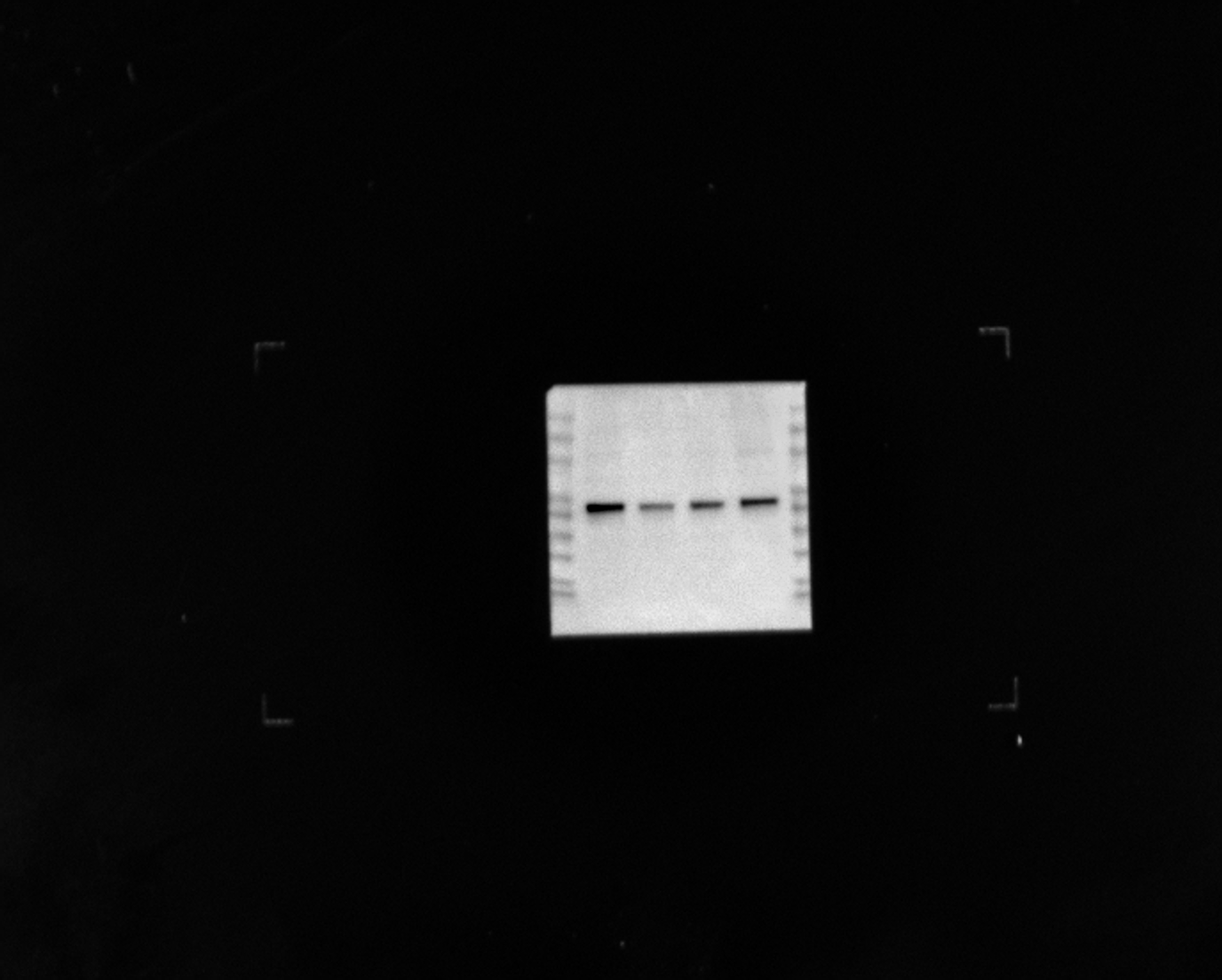

Supplement: Supplementary file 4 [file DataSheet2.zip › 原图2/Occludin/1副本.tif]

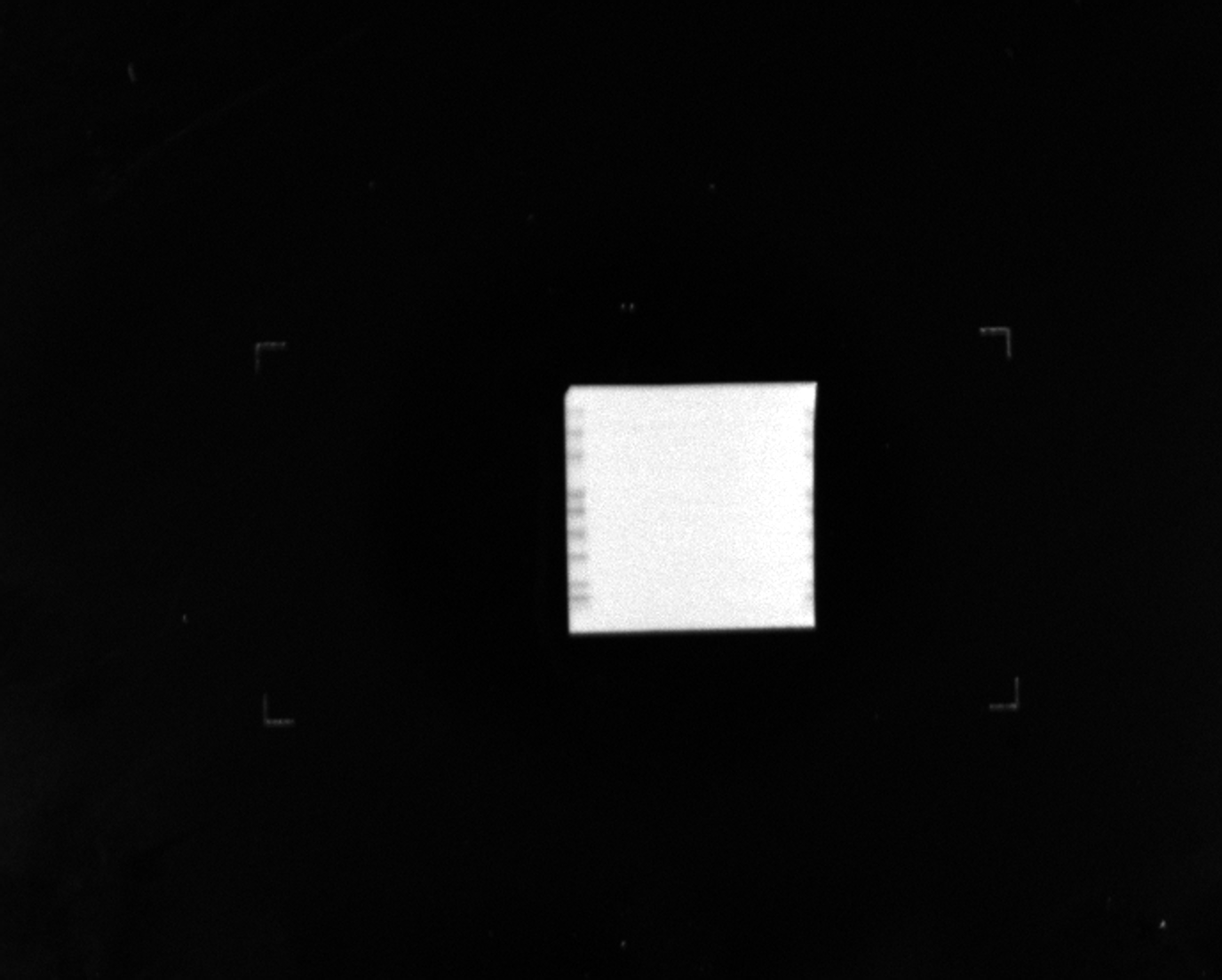

Supplement: Supplementary file 4 [file DataSheet2.zip › 原图2/Occludin/2-t.Tif]

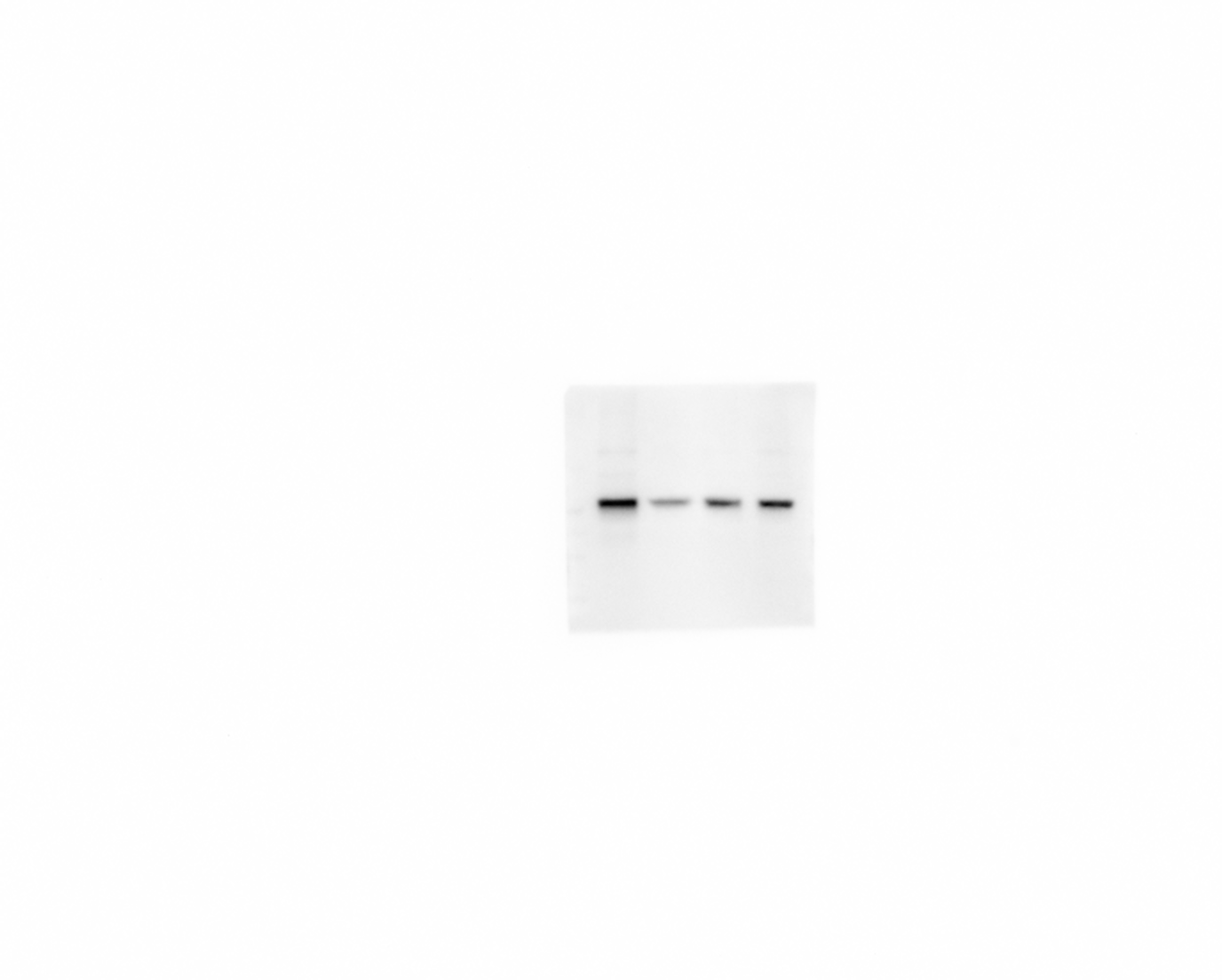

Supplement: Supplementary file 4 [file DataSheet2.zip › 原图2/Occludin/2.Tif]

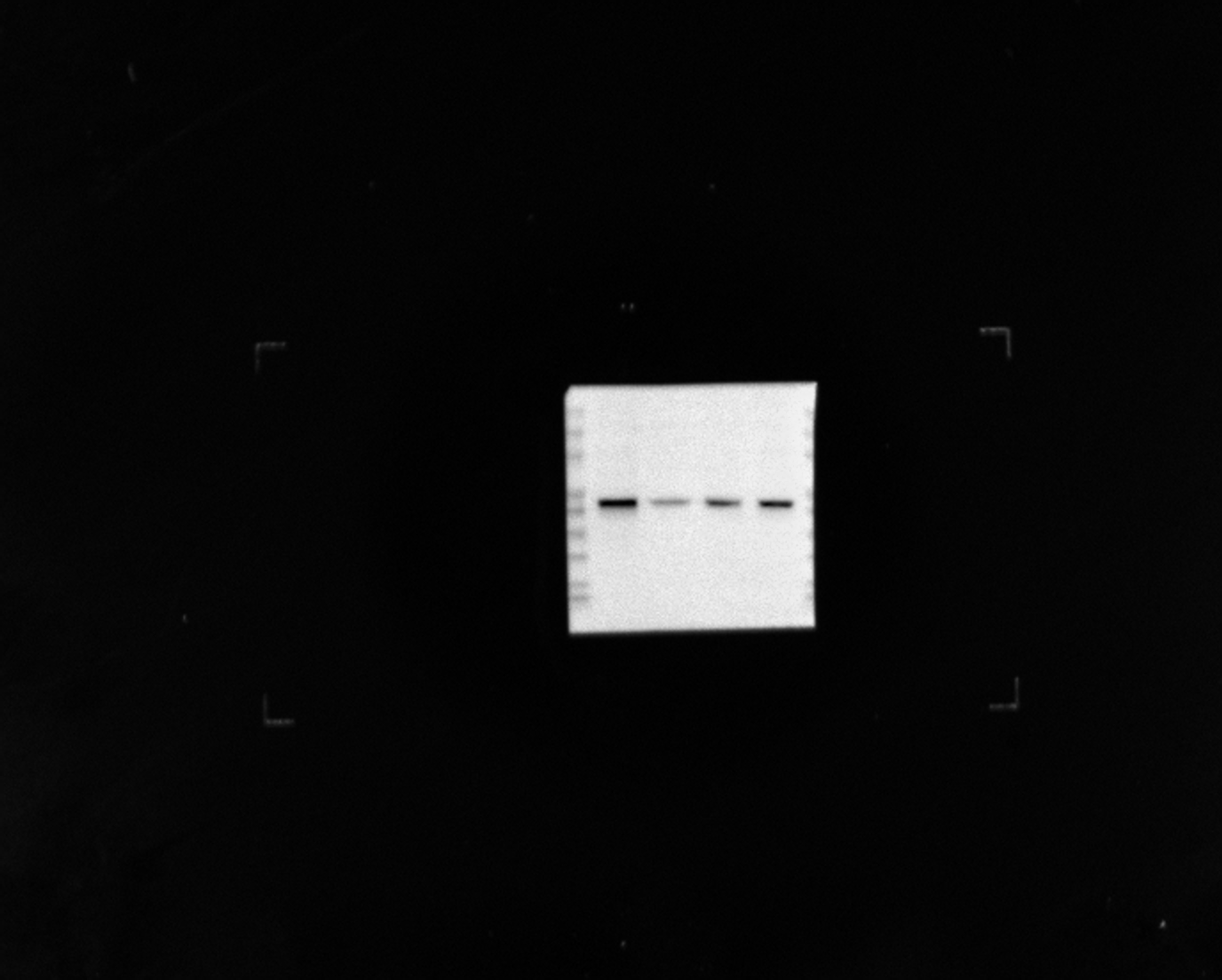

Supplement: Supplementary file 4 [file DataSheet2.zip › 原图2/Occludin/2副本.tif]

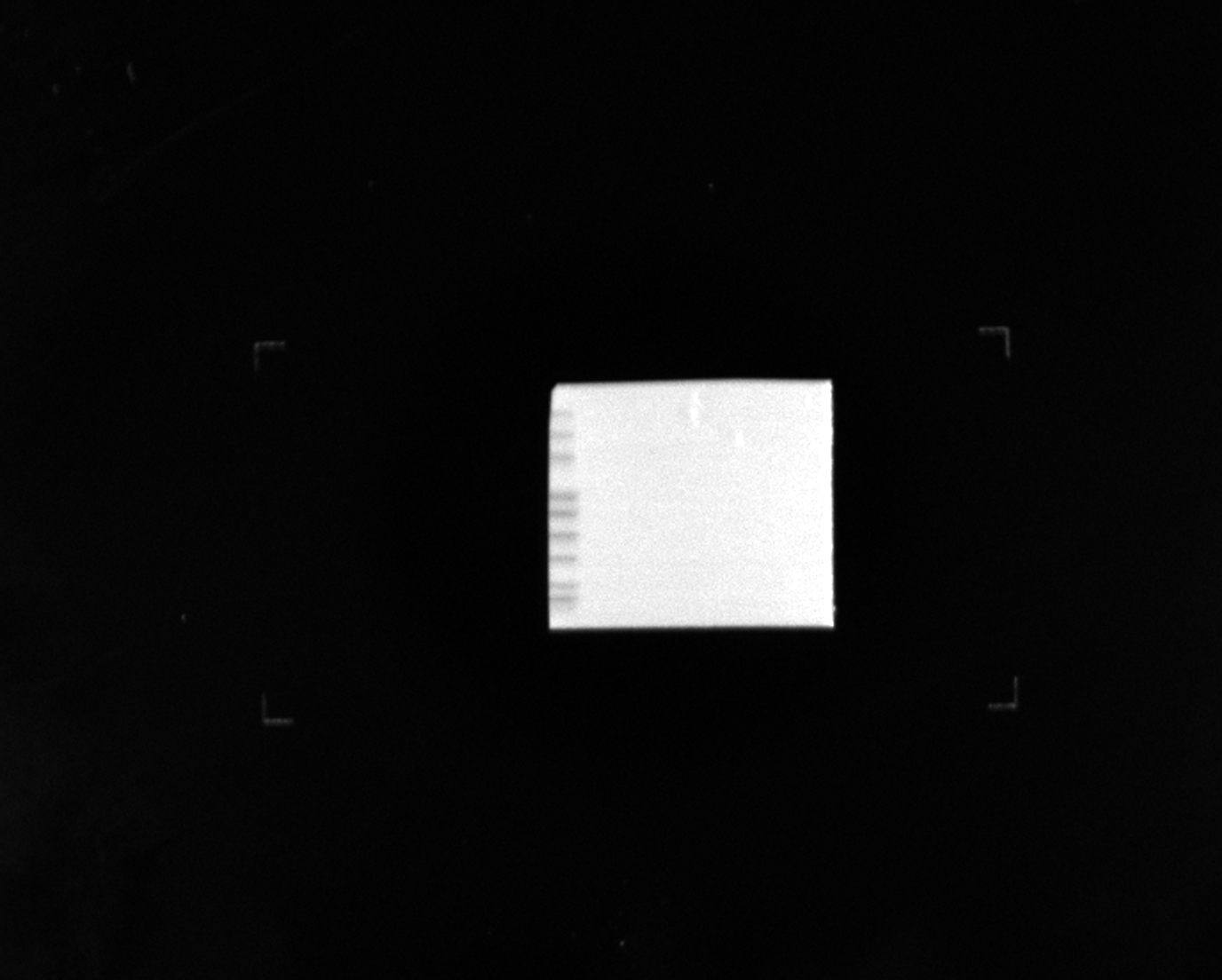

Supplement: Supplementary file 4 [file DataSheet2.zip › 原图2/Occludin/3-t.Tif]

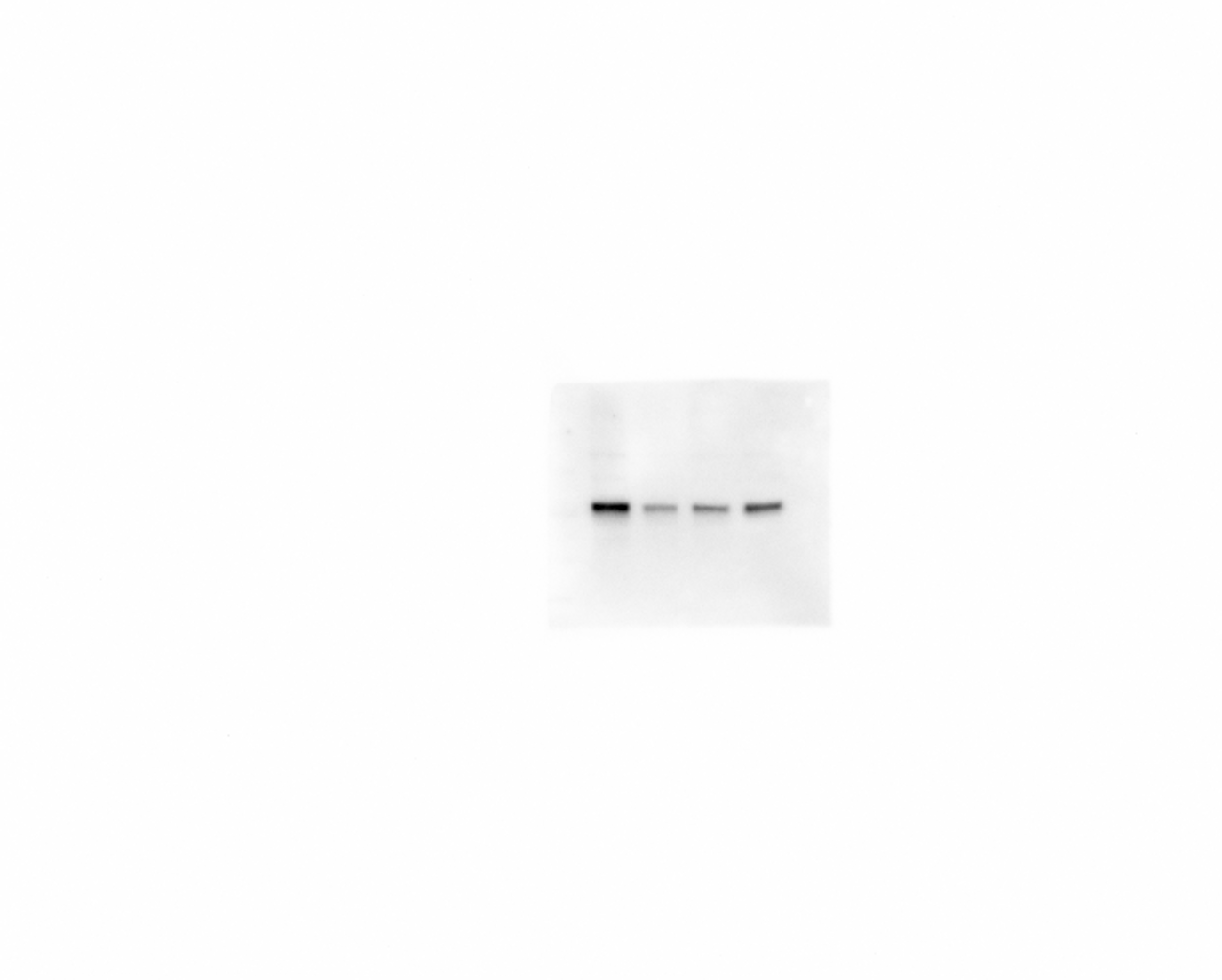

Supplement: Supplementary file 4 [file DataSheet2.zip › 原图2/Occludin/3.Tif]

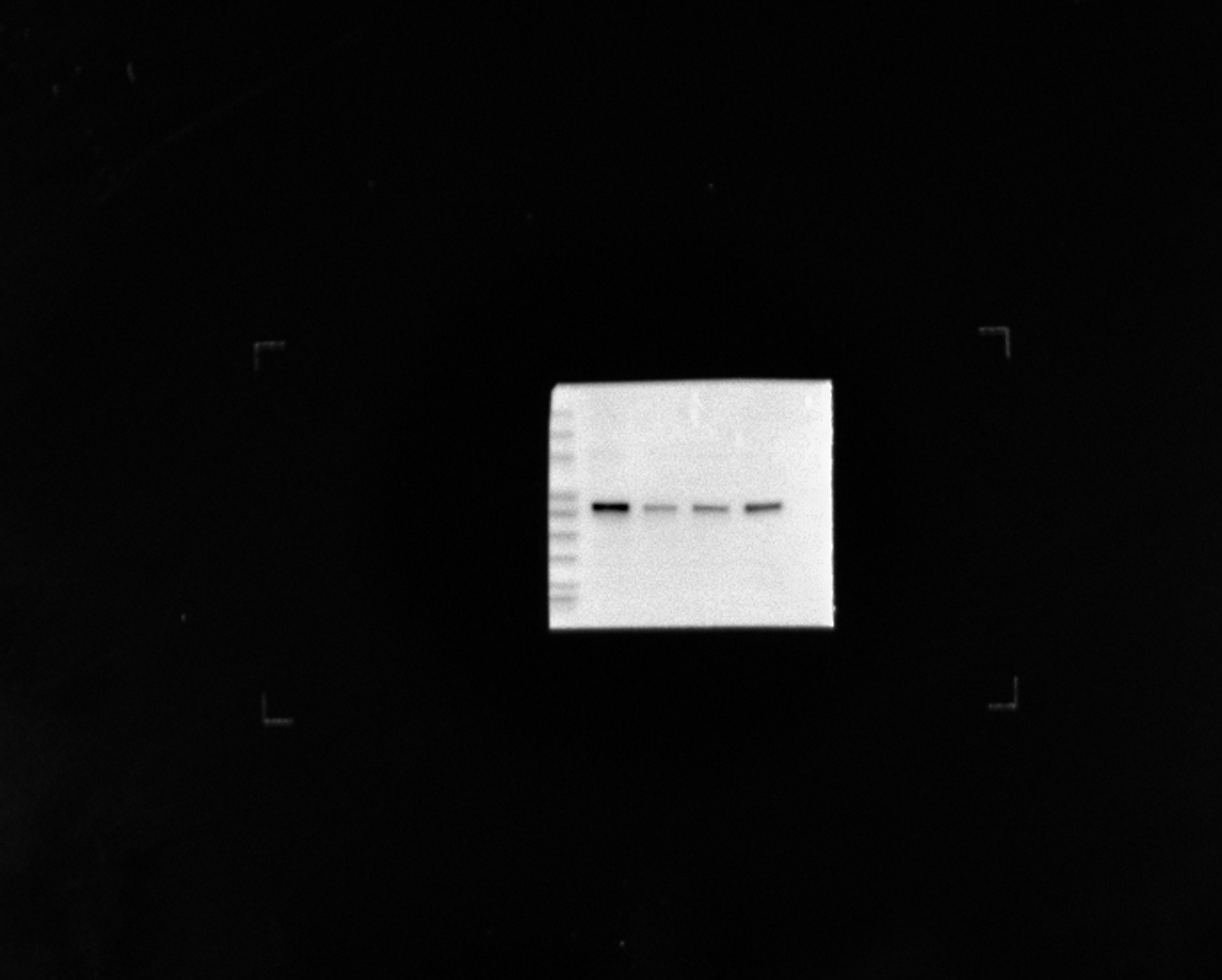

Supplement: Supplementary file 4 [file DataSheet2.zip › 原图2/Occludin/3副本.tif]
