# Supplementary figures and images for: Paeoniflorin alleviates CKD-associated constipation by modulating TPH1/AHR-related signaling and suppressing NLRP3/GSDMD-mediated pyroptosis (part 2 of 2)
Source: Front Pharmacol. 2026 Jul 10;17:1844043. doi: 10.3389/fphar.2026.1844043 (PMC13396008; doi:10.3389/fphar.2026.1844043)

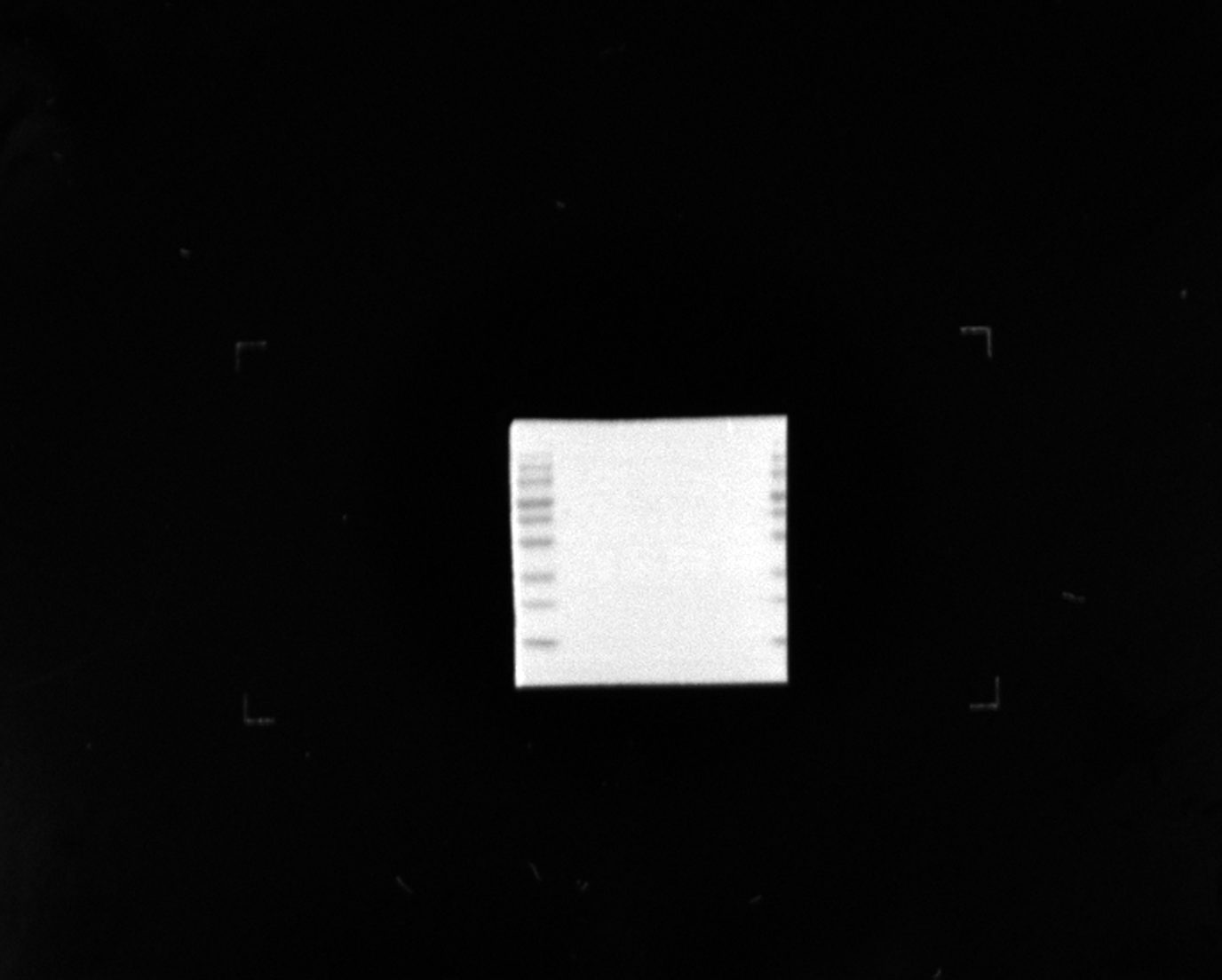

Supplement: Supplementary file 4 [file DataSheet2.zip › 原图2/TPH1/1-t.Tif]

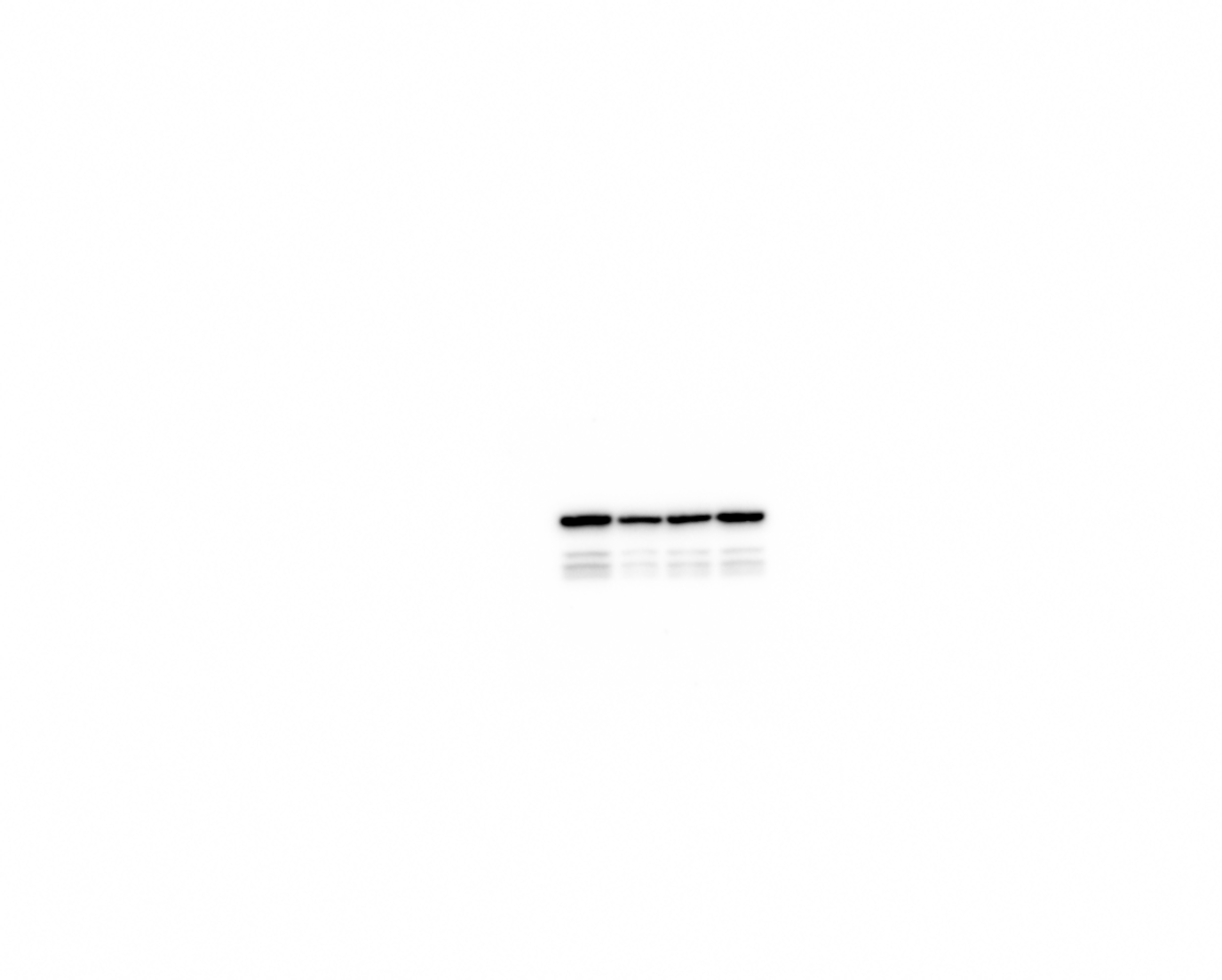

Supplement: Supplementary file 4 [file DataSheet2.zip › 原图2/TPH1/1.Tif]

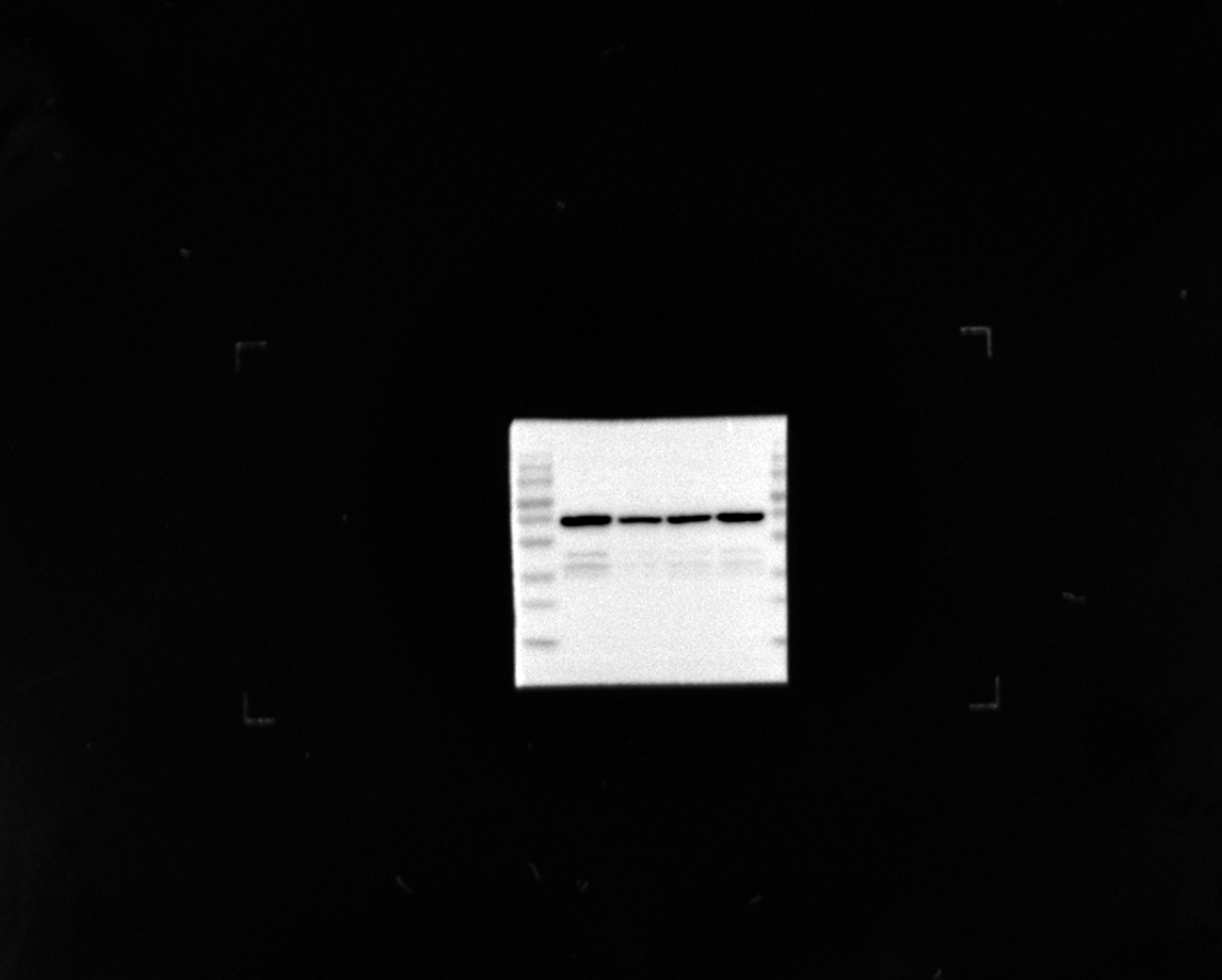

Supplement: Supplementary file 4 [file DataSheet2.zip › 原图2/TPH1/1副本.tif]

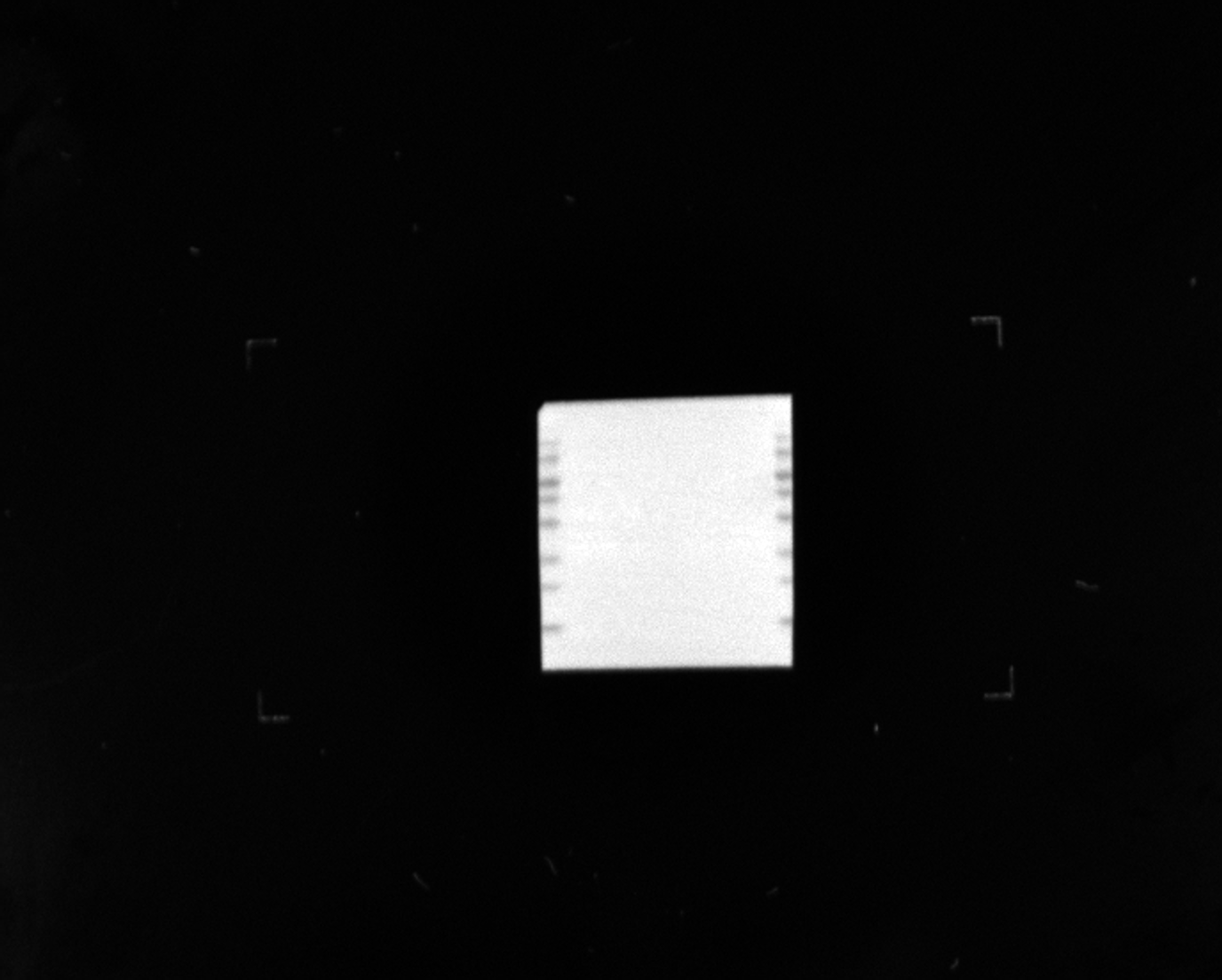

Supplement: Supplementary file 4 [file DataSheet2.zip › 原图2/TPH1/2-t.Tif]

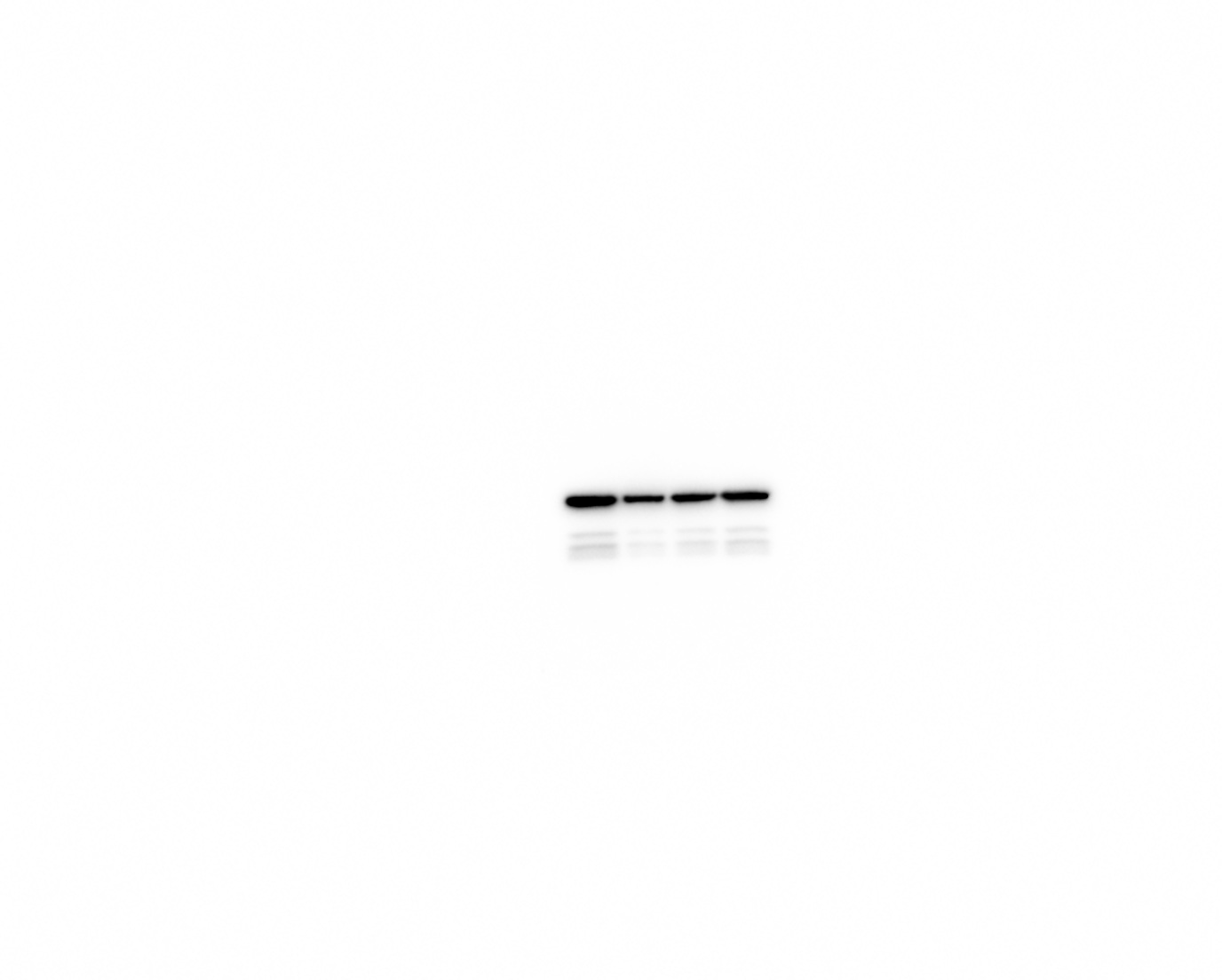

Supplement: Supplementary file 4 [file DataSheet2.zip › 原图2/TPH1/2.Tif]

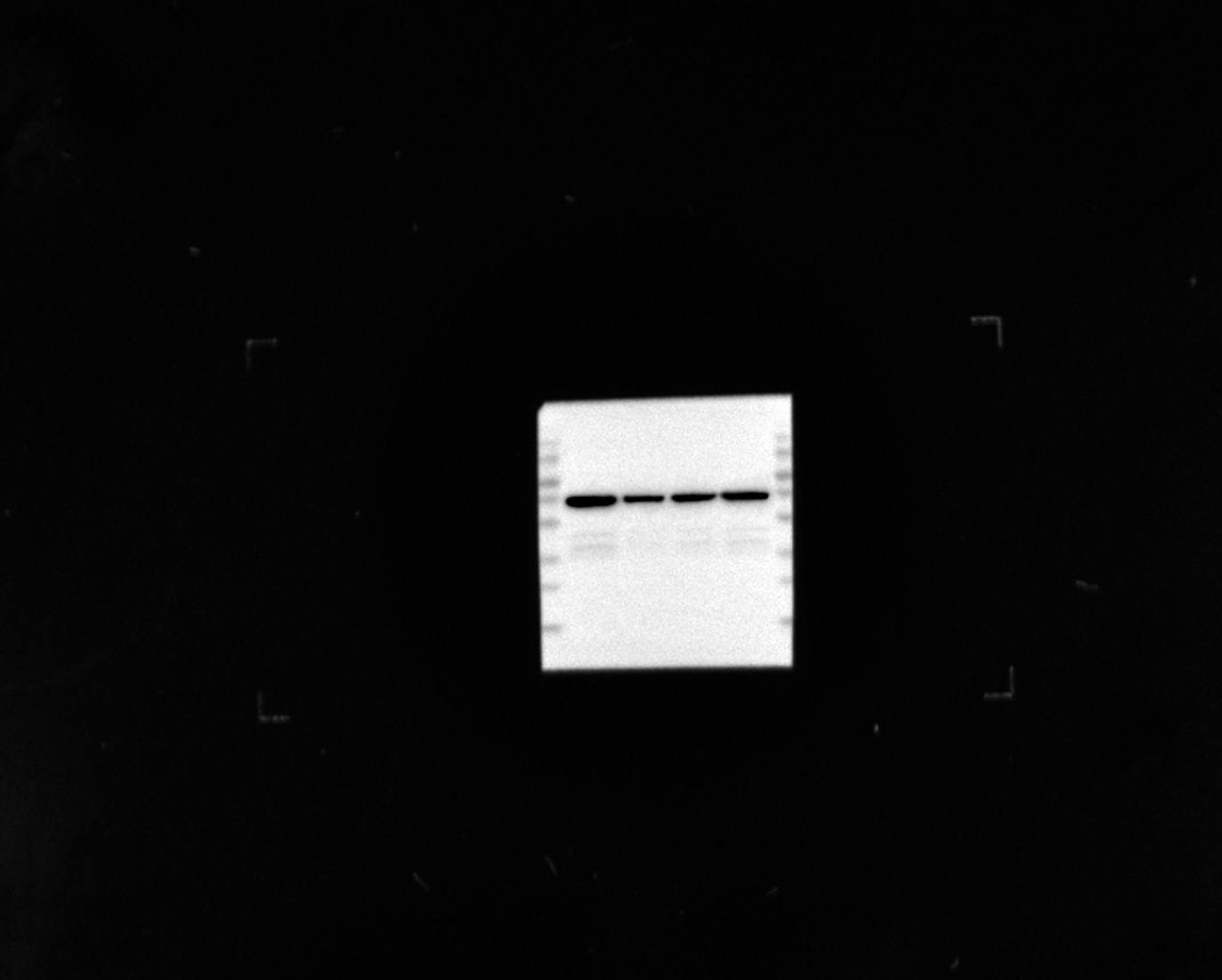

Supplement: Supplementary file 4 [file DataSheet2.zip › 原图2/TPH1/2副本.tif]

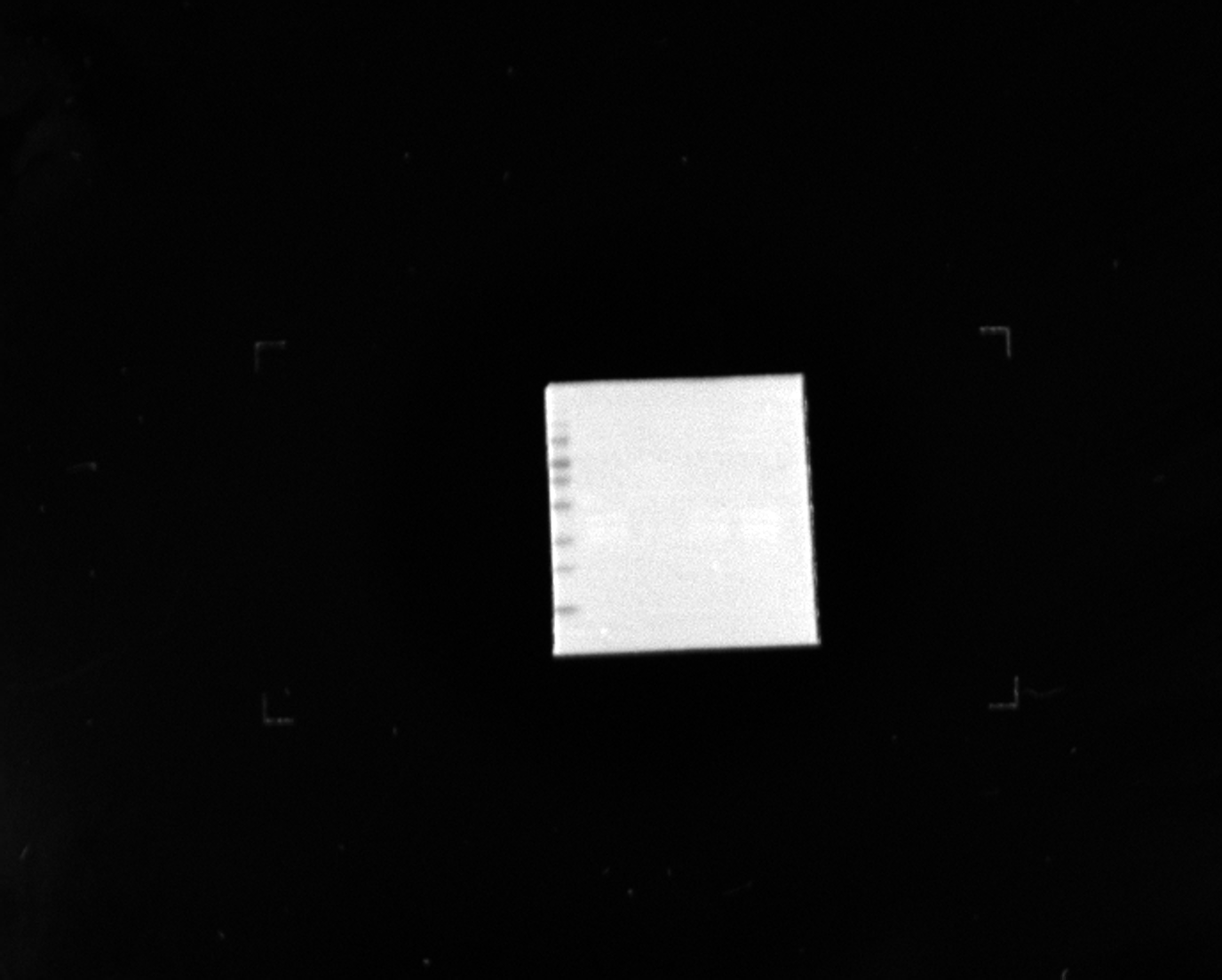

Supplement: Supplementary file 4 [file DataSheet2.zip › 原图2/TPH1/3-t.Tif]

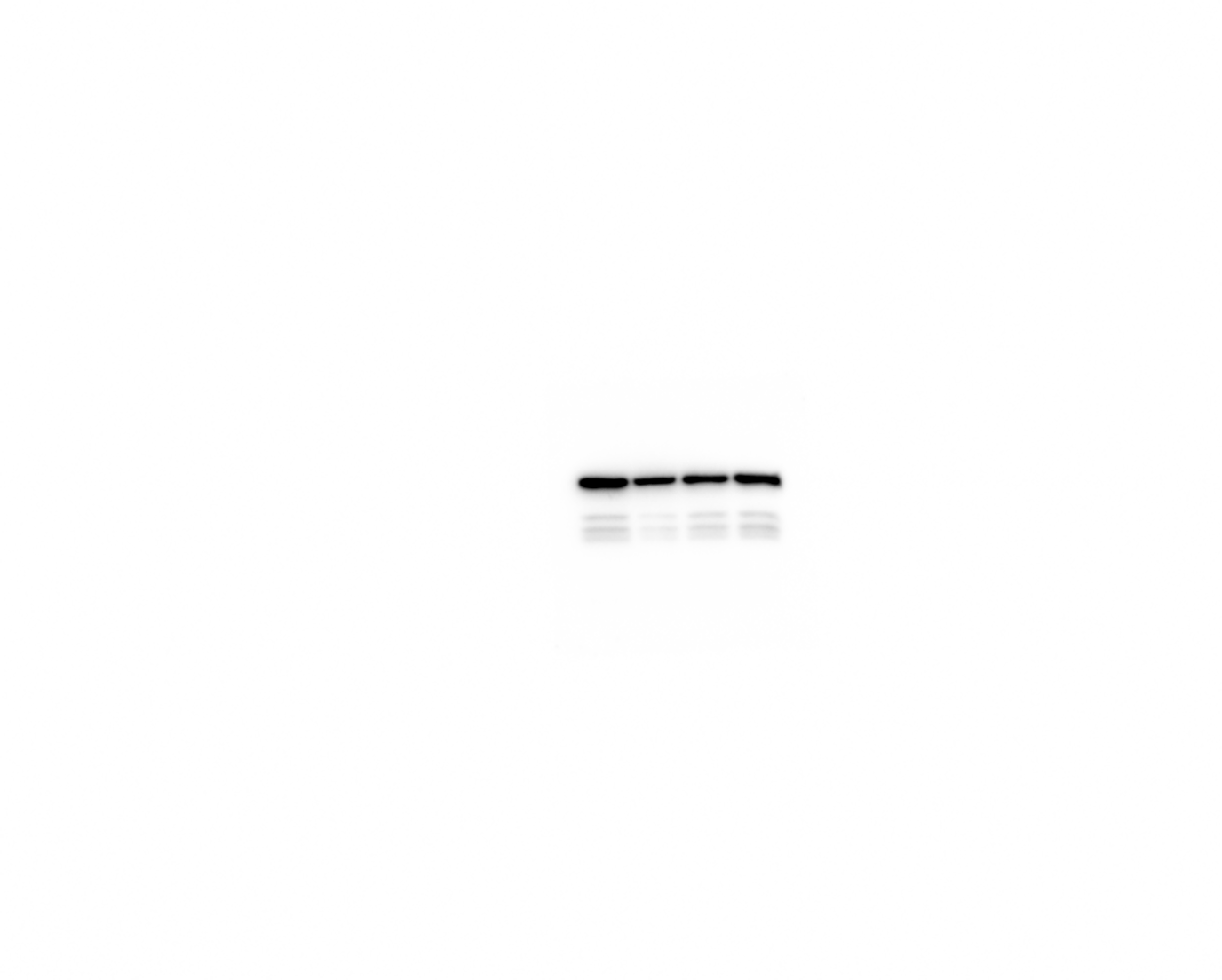

Supplement: Supplementary file 4 [file DataSheet2.zip › 原图2/TPH1/3.Tif]

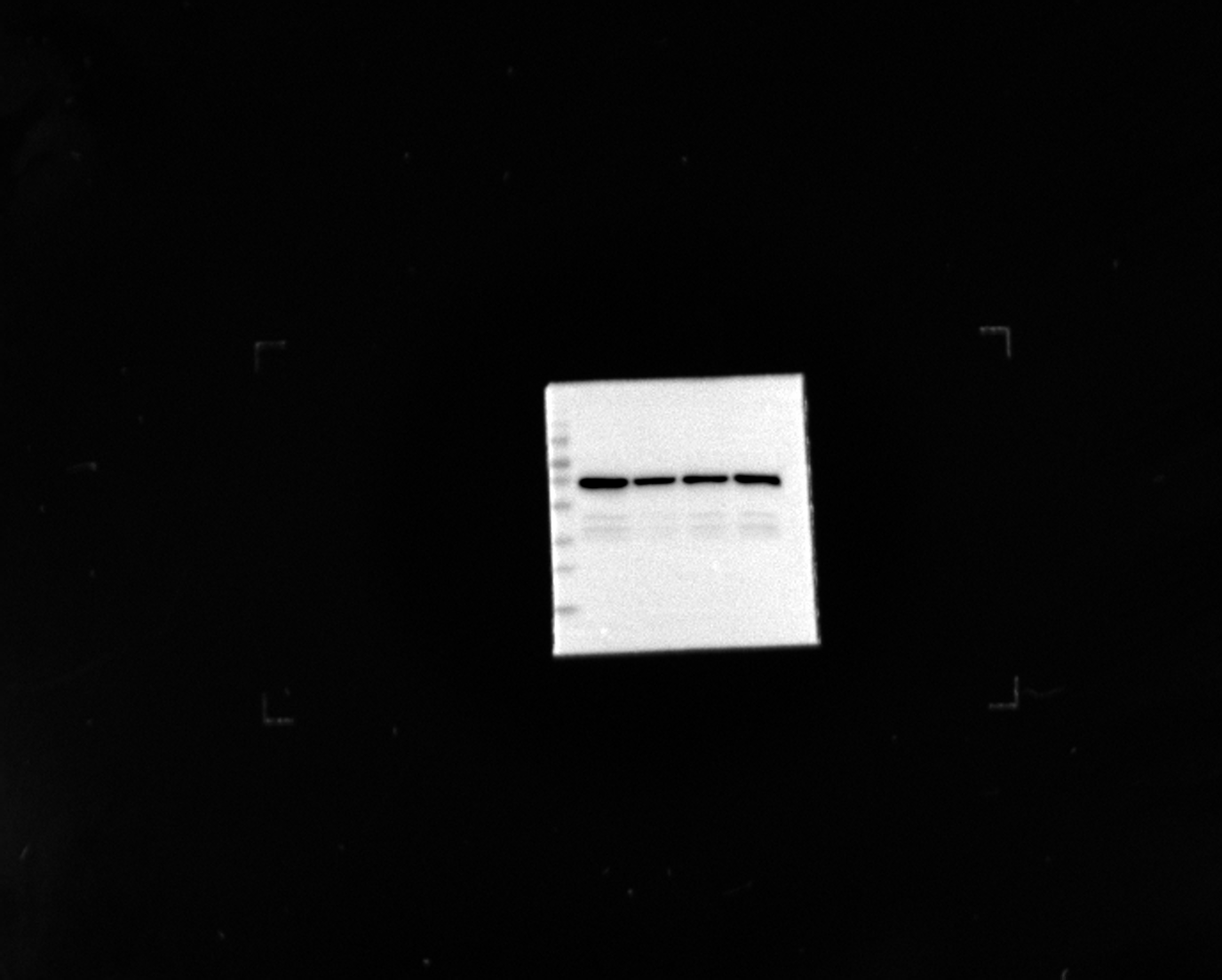

Supplement: Supplementary file 4 [file DataSheet2.zip › 原图2/TPH1/3副本.tif]

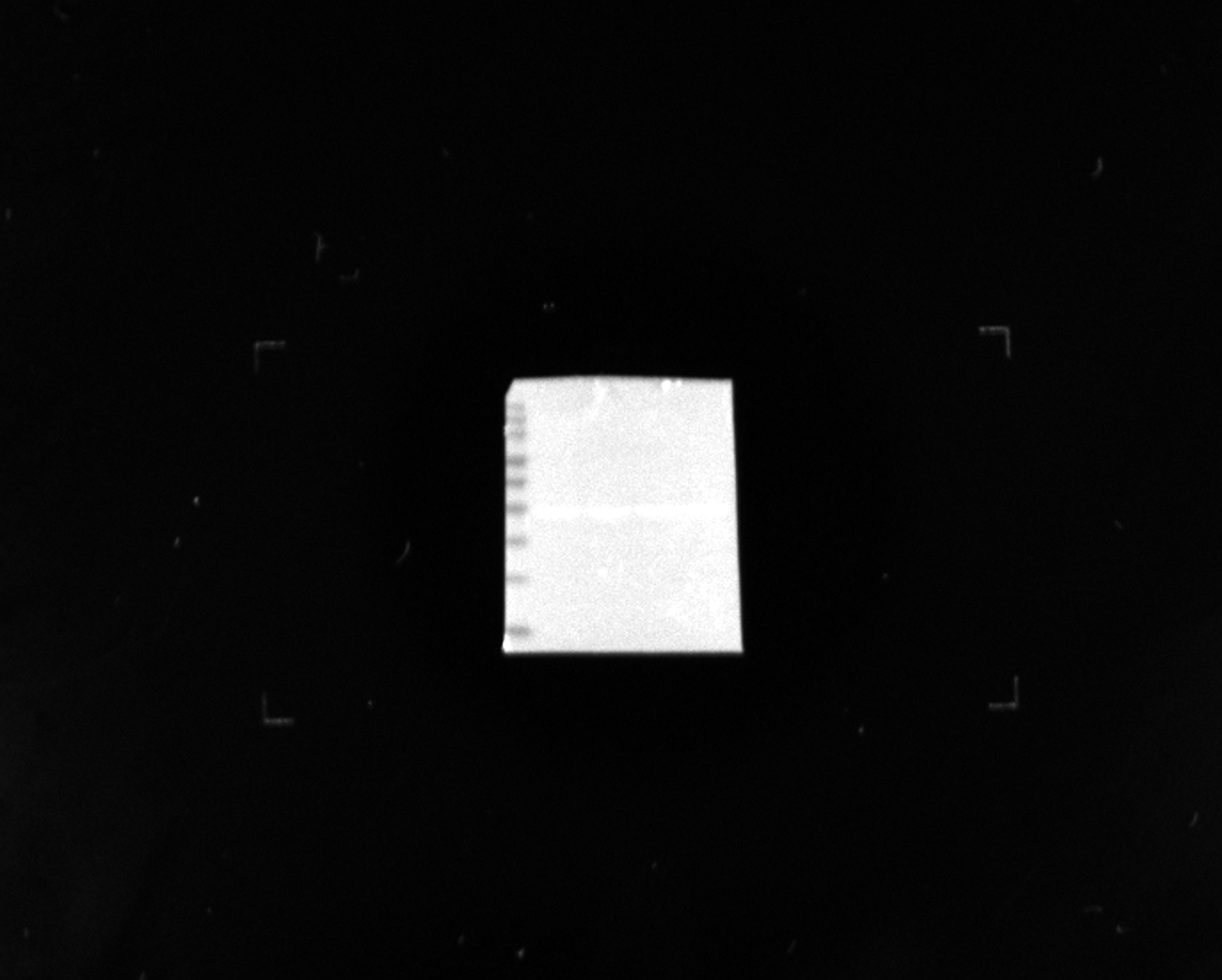

Supplement: Supplementary file 4 [file DataSheet2.zip › 原图2/β-actin/1-t.Tif]

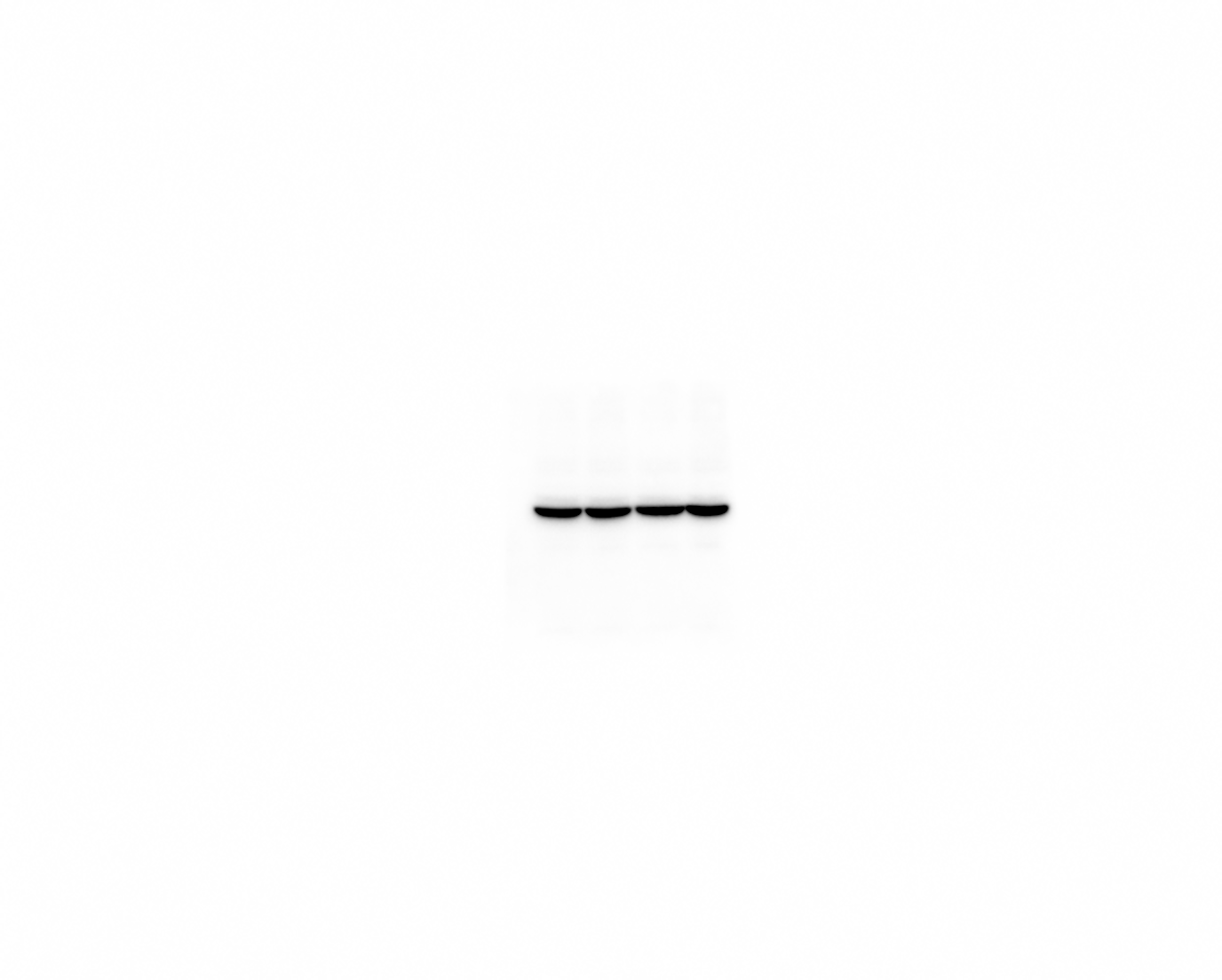

Supplement: Supplementary file 4 [file DataSheet2.zip › 原图2/β-actin/1.Tif]

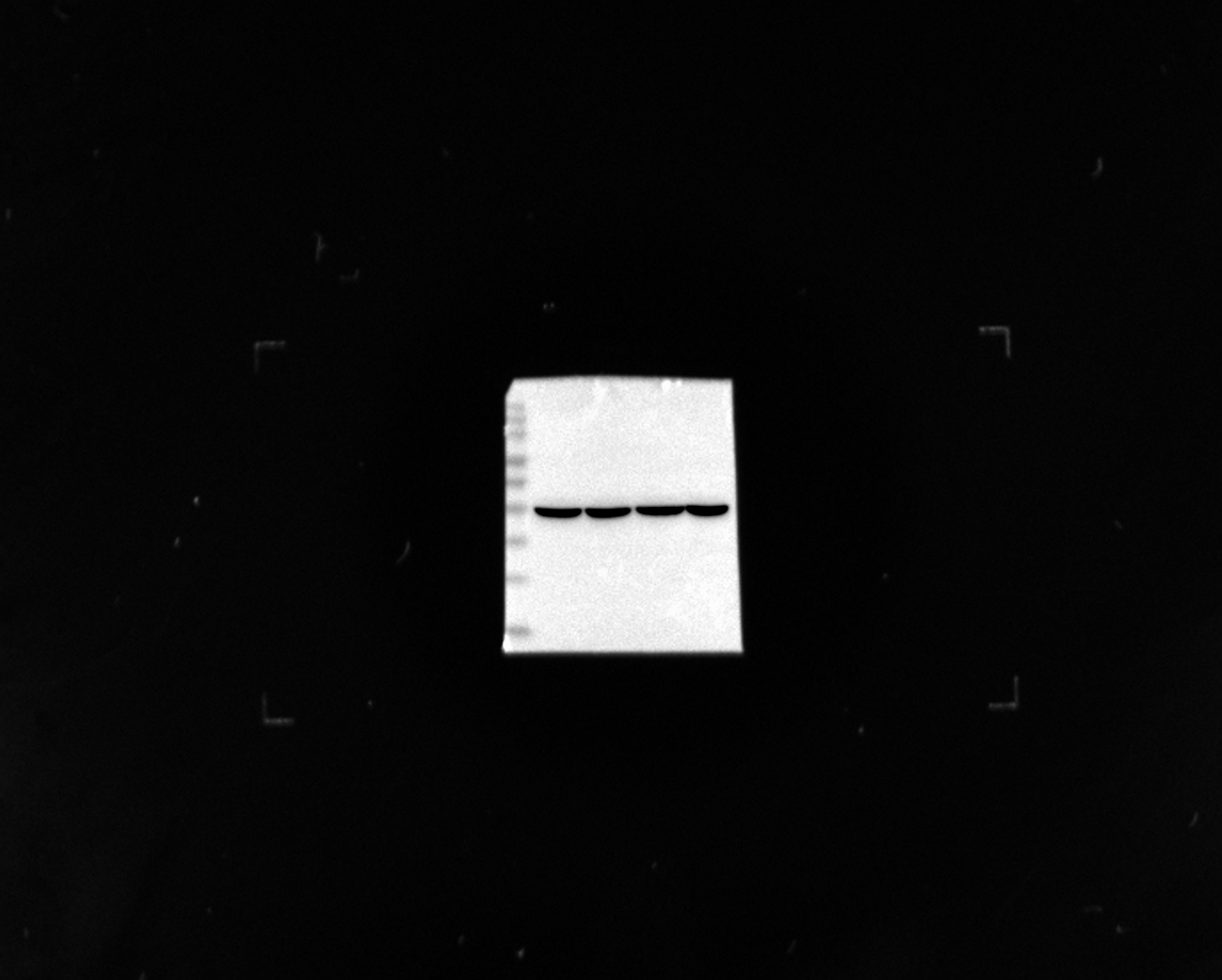

Supplement: Supplementary file 4 [file DataSheet2.zip › 原图2/β-actin/1副本.tif]

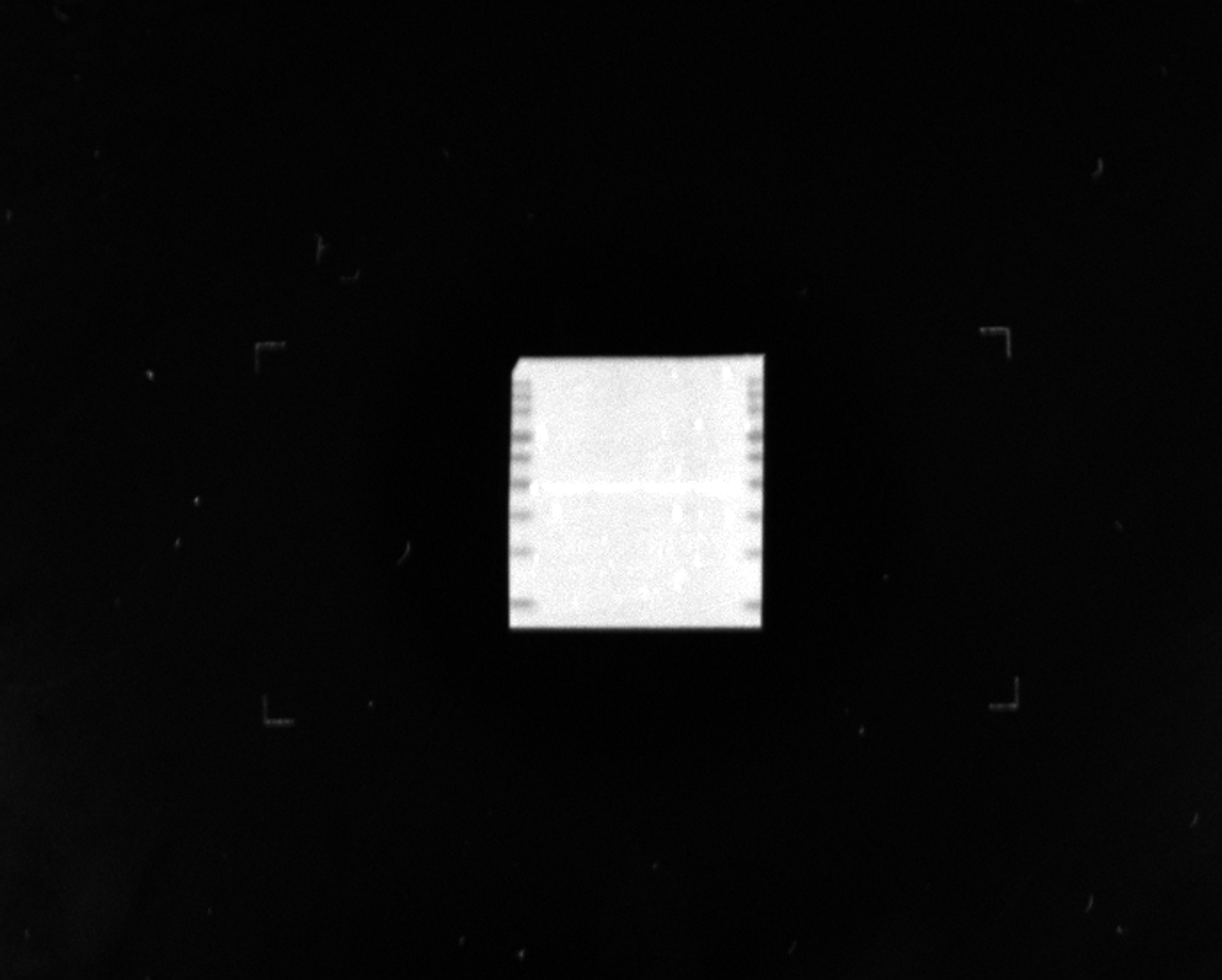

Supplement: Supplementary file 4 [file DataSheet2.zip › 原图2/β-actin/2-t.Tif]

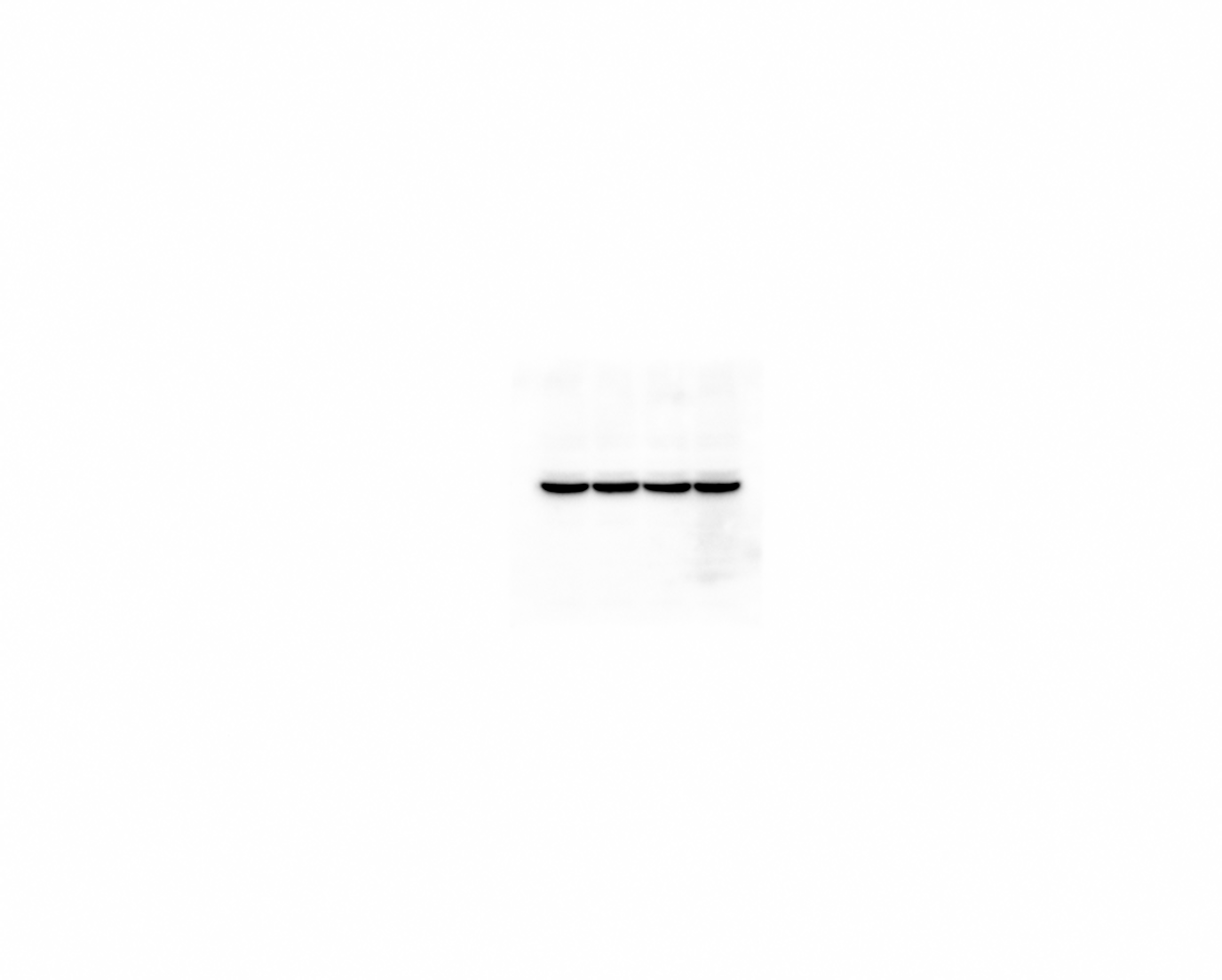

Supplement: Supplementary file 4 [file DataSheet2.zip › 原图2/β-actin/2.Tif]

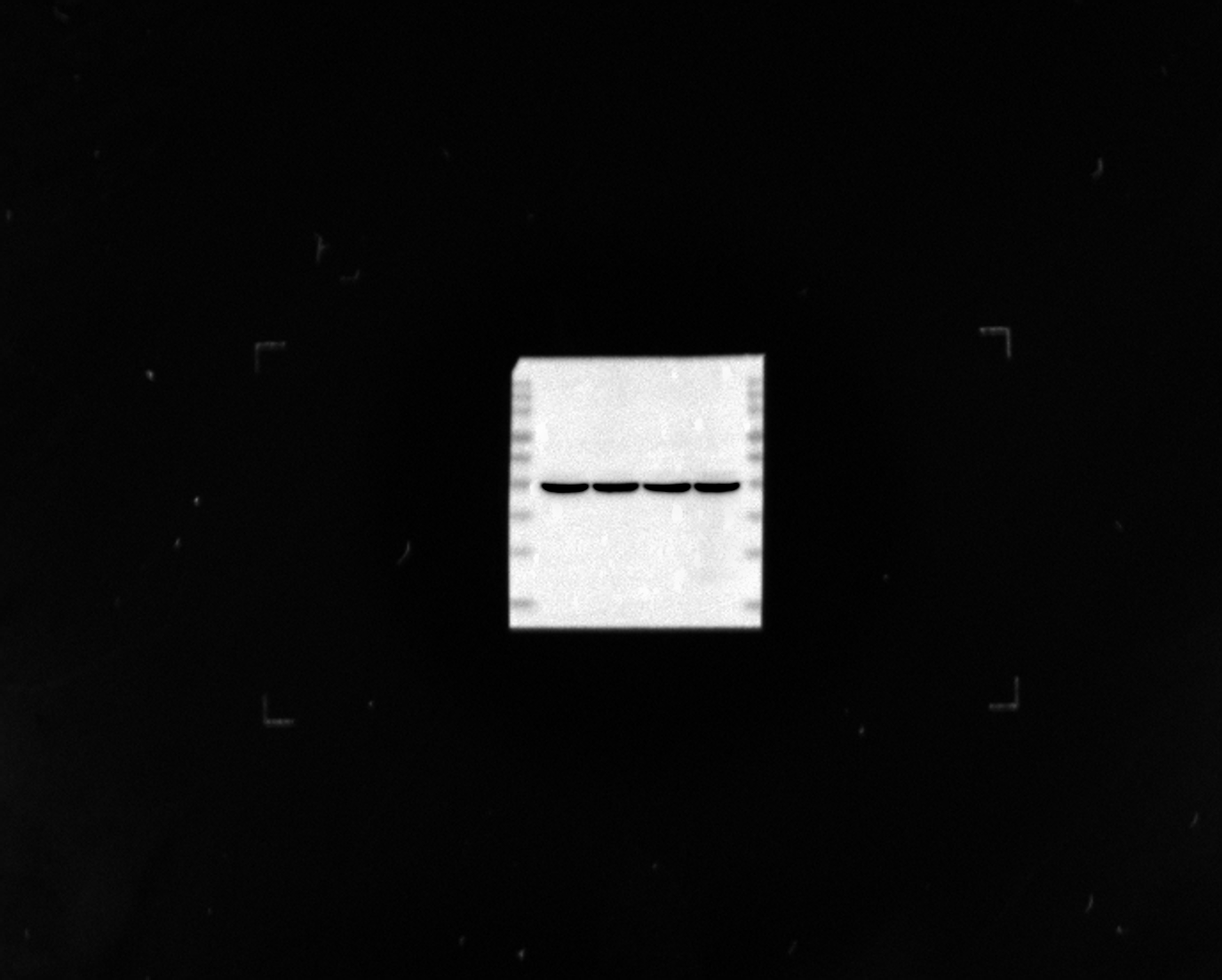

Supplement: Supplementary file 4 [file DataSheet2.zip › 原图2/β-actin/2副本.tif]

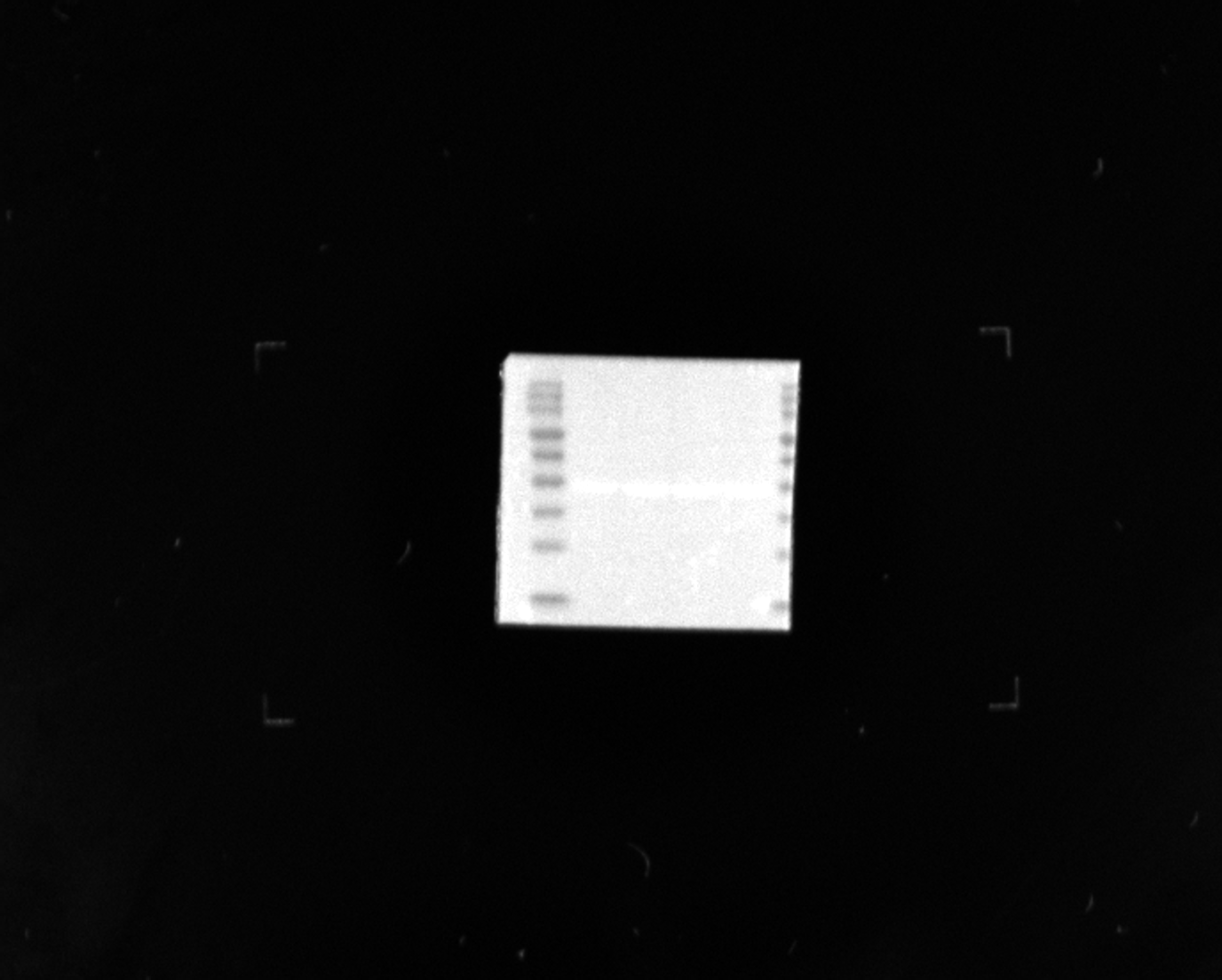

Supplement: Supplementary file 4 [file DataSheet2.zip › 原图2/β-actin/3-t.Tif]

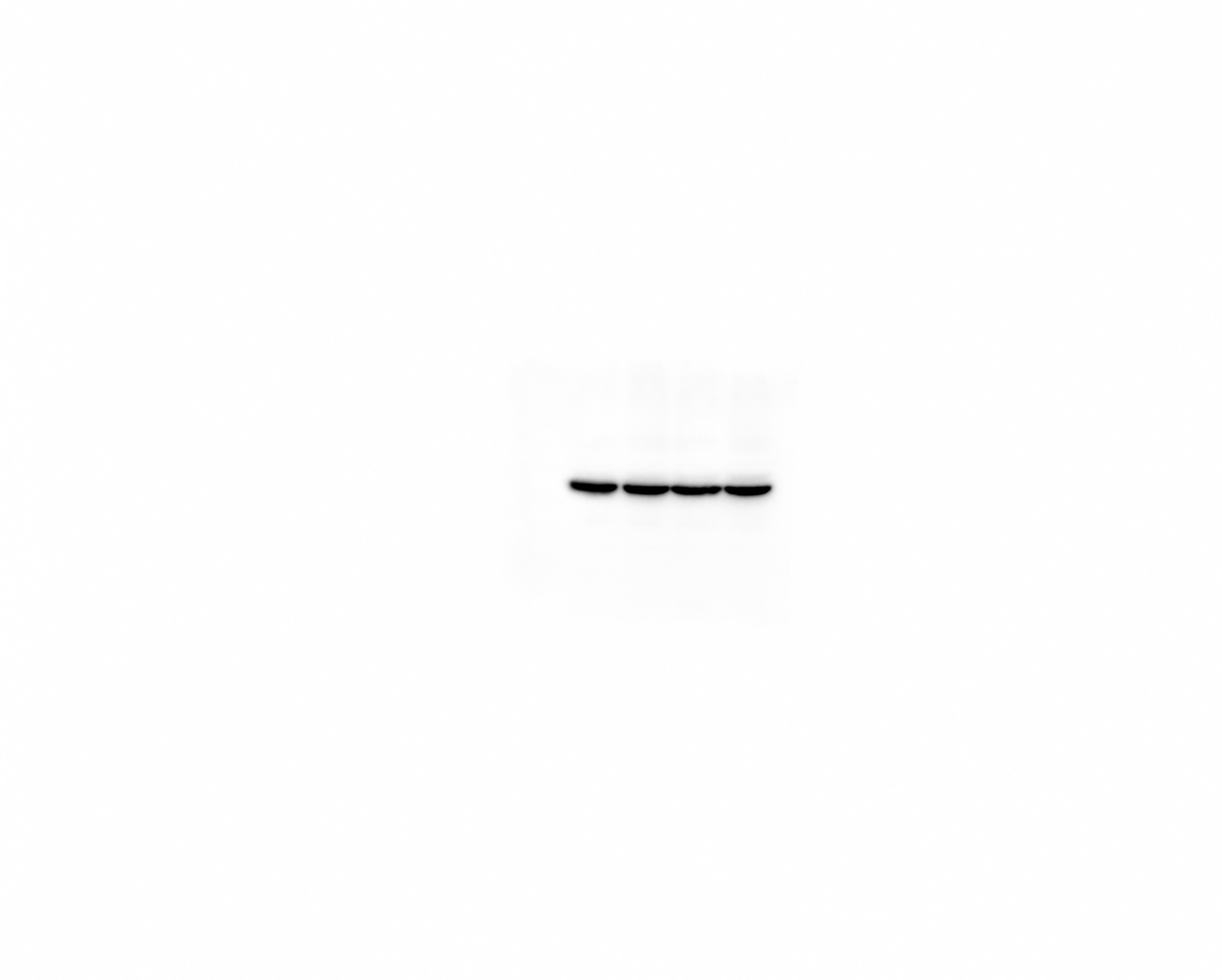

Supplement: Supplementary file 4 [file DataSheet2.zip › 原图2/β-actin/3.Tif]

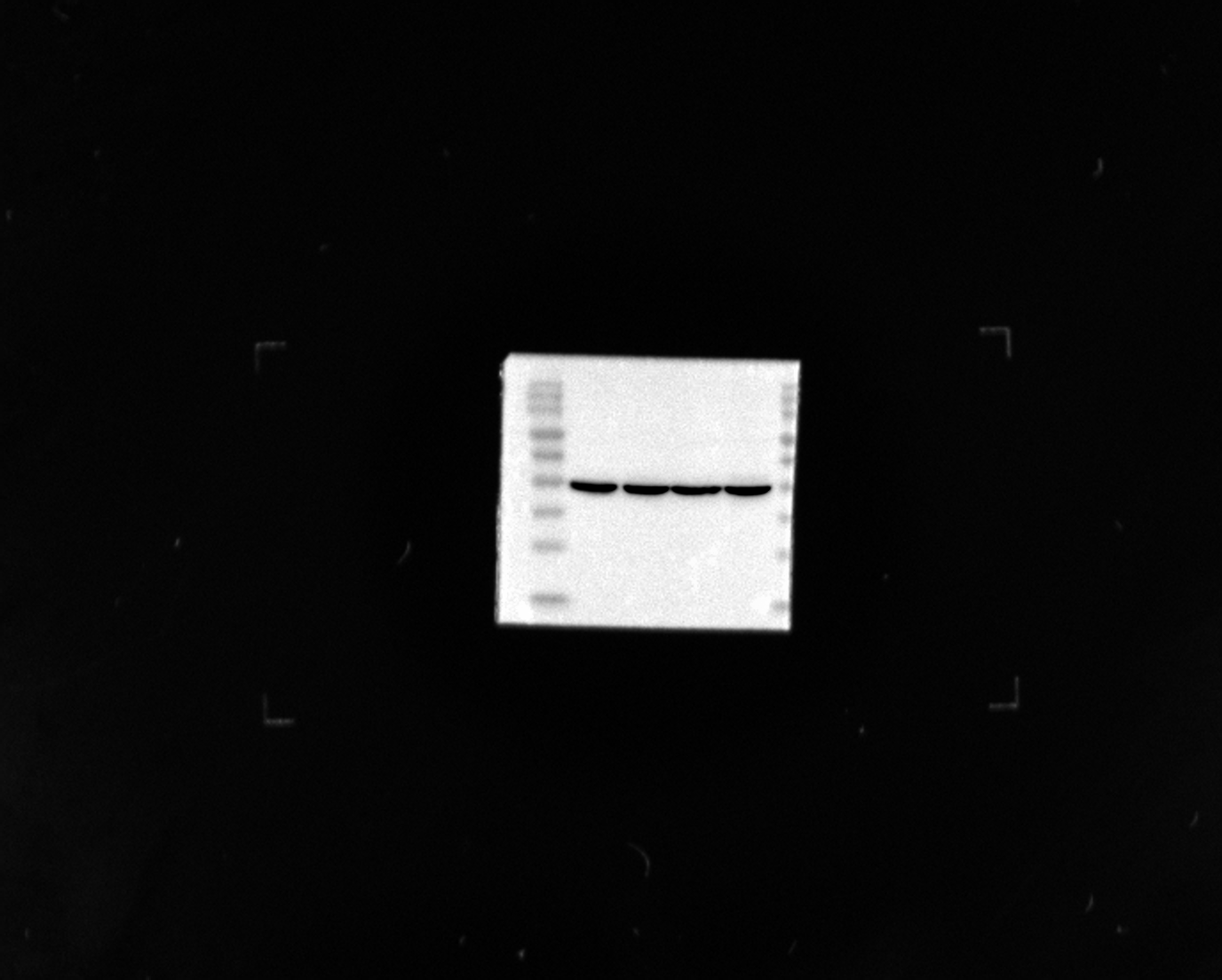

Supplement: Supplementary file 4 [file DataSheet2.zip › 原图2/β-actin/3副本.tif]
